# Gold-catalyzed stereoselective dearomatization/metal-free aerobic oxidation: access to 3-substituted indolines/oxindoles†
†Electronic supplementary information (ESI) available. CCDC 1551068, 1551070, 1572912 and 1572913. For ESI and crystallographic data in CIF or other electronic format see DOI: 10.1039/c7sc04086e


**DOI:** 10.1039/c7sc04086e

**Published:** 2017-11-06

**Authors:** Kai Liu, Guangyang Xu, Jiangtao Sun

**Affiliations:** a Jiangsu Key Laboratory of Advanced Catalytic Materials & Technology , School of Petrochemical Engineering , Changzhou University , Changzhou 213164 , P. R. China . Email: jtsun08@gmail.com ; Email: jtsun@cczu.edu.cn

## Abstract

An unprecedented dearomatization of indoles with diazoesters has been developed *via* cationic gold(i) catalysis. The functionalization selectively occurs at the C3-position to deliver methylene indole derivatives in good yields with excellent *Z*-selectivity, demonstrating unusual reactivity and selectivity compared with other noble metal catalysis. Importantly, simply followed by silica gel adsorption, an unprecedented metal-free aerobic oxidation occurs for indoles bearing N-electron donating substituents, providing a novel and efficient approach towards 3-substituted indolin-2-ones with a newly formed quaternary stereocenter in excellent stereoselectivity. Notably, these processes afford direct and selective access to a variety of valuable intermediates from abundant feedstock chemicals.

## Abstract 2

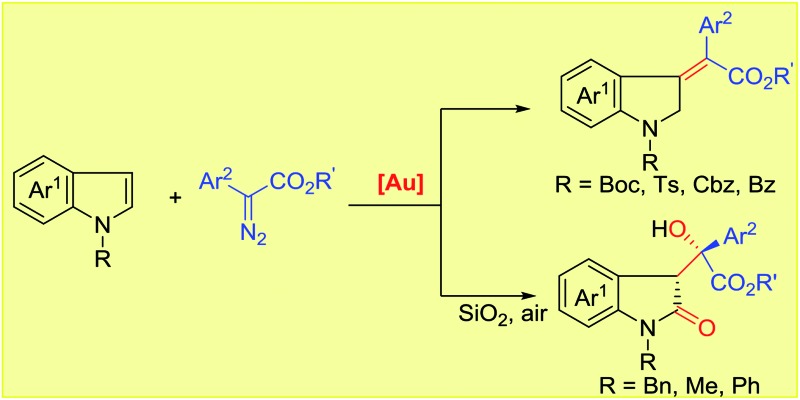
An unprecedented gold-catalyzed dearomatization and a metal-free aerobic oxidation have been developed.

## Introduction

**Scheme 1:**
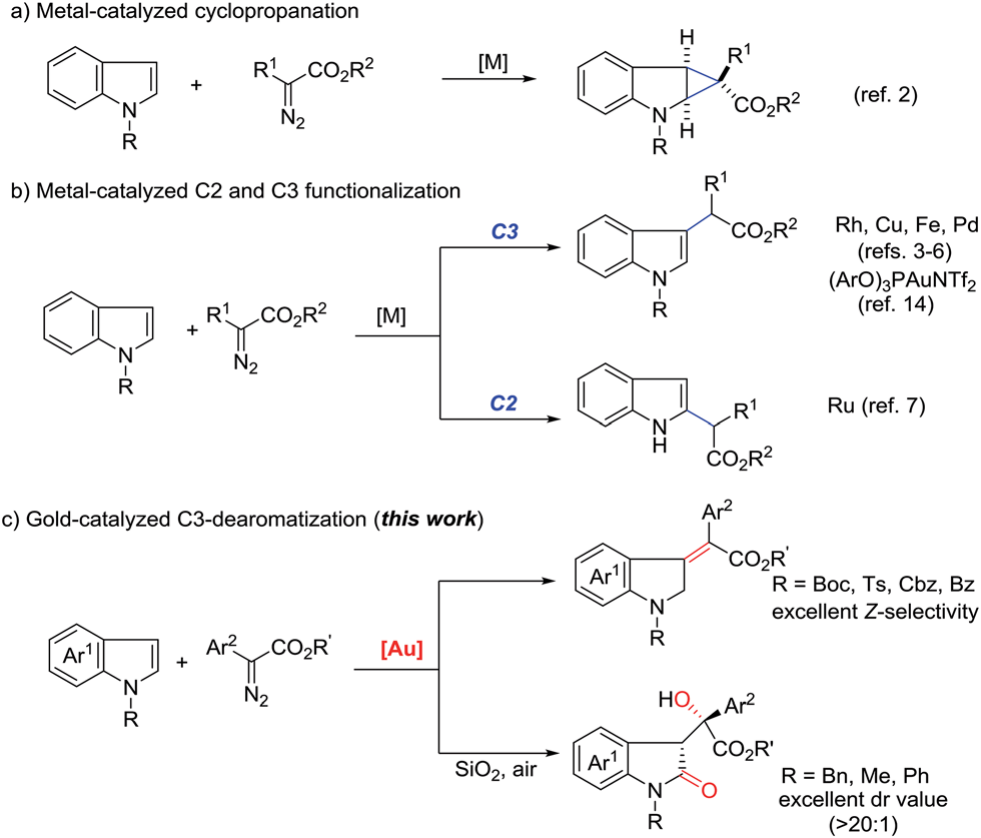
Functionalization of indoles by metal-carbene transfer from diazo compounds: previous reports and our discovery.

Transition-metal-catalyzed carbene transfer from diazo compounds represents a powerful tool in modern organic synthesis, allowing the rapid assembly of a range of valuable structures which cannot be easily achieved by other methodologies.^1^ Particularly, different metal-carbenes often exhibit distinct reactivity and selectivity toward the same reaction precursors, which increases molecular complexity. A good example is the reaction of diazoesters with 2,3-nonsubstituted indoles, which often leads to two principal products, namely cyclopropane derivatives (Scheme 1a)^2^ and formal C(sp^2^)–H insertion products (Scheme 1b).^3^–^7^ Indeed, for the addition reaction, the use of rhodium,^3^ copper,^4^ iron^5^ and palladium^6^ complexes selectively afforded C3-alkylation products. In contrast, C2-alkylation has been observed for 1H-indoles upon exposure to a ruthenium catalyst.^7^ Additionally, annulation^8^ and the N–H insertion reaction^9^ have also been reported.

On the other hand, recent literature disclosed that the reactivity and chemoselectivity of gold-carbene highly depended on the electronic and steric properties of the ancillary ligand to the gold center,^10^,^11^ as well as the choice of counterion.^12^ Variation of the gold catalysts has recently been shown to even allow the formation of gold(iii) intermediates from gold(i) precursors.^13^ In 2014, Shi and co-workers described a ligand-controlled gold-catalyzed addition of arenes to α-aryl diazoesters.^14^ They mentioned one example of indole C3-alkylation in the presence of an electron-deficient phosphite-gold catalyst, which exhibited similar chemo- and site-selectivity to rhodium and copper catalysis (Scheme 1b). However, we recently found that N-heterocyclic carbene (NHC) gold complexes displayed inverse chemoselectivity to phosphite gold catalysts even for the same substrates.15*b* Thus, we envisioned that, when exposed to different gold catalysts, the reaction of indoles with diazo compounds would probably result in distinct reaction pathways. In continuation with our interests in gold-carbene chemistry,^15^ as anticipated but not expected, herein we report the unprecedented stereoselective dearomatization of indoles with diazoesters under cationic gold(i) catalysis (Scheme 1c). Furthermore, when N-electron-donating substituted indoles are utilized, an unprecedented metal-free aerobic oxidation occurs after the initial dearomatization.

## Results and discussion

At the outset, we employed *N*-boc indole **1a**, *N*-benzyl indole **2a** and phenyl diazoacetate **3a** as model substrates to investigate the reaction (Table 1). The use of Ph_3_PAuCl/AgSbF_6_ (5 mol%) in dichloromethane at room temperature afforded C3-insertion product **5a** in 70% yield (entry 1), while JohnPhosAuCl/AgSbF_6_ gave 16% yield of **5a** (entry 2). When *t*-BuXPhosAuCl was used, 10% yield of **4a** was obtained although **5a** was still the major product (entry 3). Not surprisingly, the electron-deficient phosphite gold complex only gave **5a** as a single product (entry 4). Gratifyingly, the use of IPrAuCl/AgSbF_6_ provided **4a** in 70% yield (entry 5). We therefore started to survey other NHC gold complexes as well so as to try different counterions. To our delight, the yield of **4a** was improved to 79% by IPrAu(PhCN)SbF_6_ (entry 6), and was further increased to 81% by IPrAu(PhCN)BAr_F_^16^ (entry 7). A screen of the solvents revealed chloroform was the best one (entries 8 to 11), providing **4a** in 87% yield (entry 9), and no significant side products were detected. The structure of **4a** was confirmed with NMR spectroscopy and was further identified using X-ray analysis.^17^ Notably, when N-benzyl indole **2a** was subjected to this reaction, the corresponding 3-methyleneindoline was not obtained and 3-substituted indolin-2-one **6a** was obtained instead in 77% yield (entry 12, see ESI† for details), indicating that an aerobic oxidation occurred. Variation of the solvents did not improve the reaction (entries 13 to 15).

**Table 1:** Optimization of the reaction conditions^*a*^

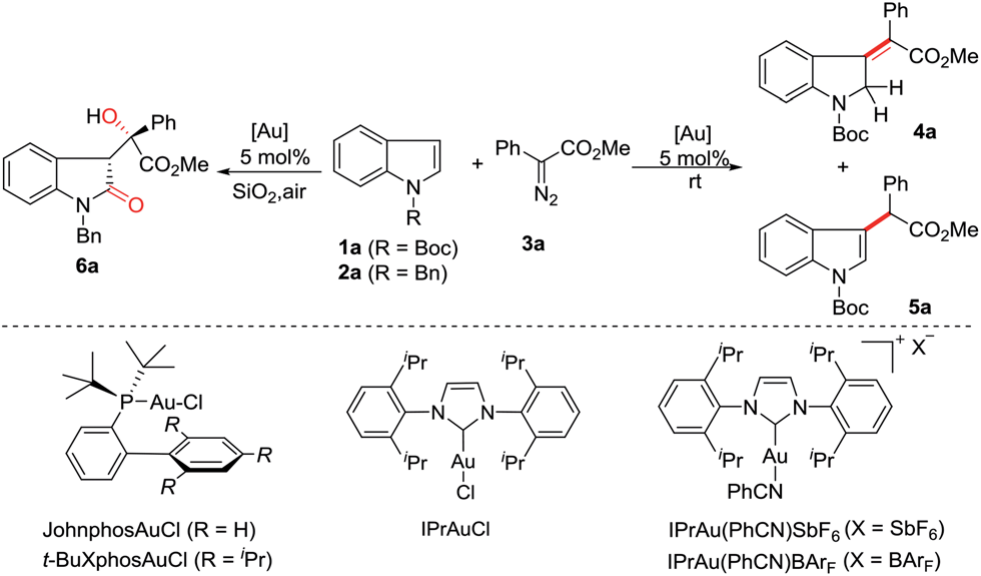					
Entry	Indole	Catalyst	Solvent	Yield^*b*^ (%)	
				**4a**(**5a**)	**6a**
1	**1a**	Ph_3_PAuCl/AgSbF_6_	CH_2_Cl_2_	0(70)	—
2	**1a**	JohnPhosAuCl/AgSbF_6_	CH_2_Cl_2_	0(16)	—
3	**1a**	*t*-BuXPhosAuCl/AgSbF_6_	CH_2_Cl_2_	10(61)	—
4	**1a**	(ArO)_3_PAuCl/AgSbF_6_	CH_2_Cl_2_	0(75)	—
5	**1a**	IPrAuCl/AgSbF_6_	CH_2_Cl_2_	70(13)	—
6	**1a**	IPrAu(PhCN)SbF_6_	CH_2_Cl_2_	79(10)	—
7	**1a**	IPrAu(PhCN)BAr_F_	CH_2_Cl_2_	81(8)	—
8	**1a**	IPrAu(PhCN)BAr_F_	DCE	81(9)	—
**9**	**1a**	**IPrAu(PhCN)BAr**_**F**_	**CHCl**_**3**_	**87(<5)**	**—**
10	**1a**	IPrAu(PhCN)BAr_F_	Toluene	70(15)	—
11	**1a**	IPrAu(PhCN)BAr_F_	THF	25(<5)	—
**12**	**2a**	**IPrAu(PhCN)BAr**_**F**_	**CHCl**_3_	**—**	**77**
13	**2a**	IPrAu(PhCN)BAr_F_	CH_2_Cl_2_	—	70
14	**2a**	IPrAu(PhCN)BAr_F_	DCE	—	65
15	**2a**	IPrAu(PhCN)BAr_F_	THF	—	50

^*a*^Reaction conditions: **1a** or **2a** (0.2 mmol) and **3a** (0.3 mmol) in 2 mL of the solvent were added to a solution of 5 mol% gold catalyst in 2 mL of the solvent *via* a syringe pump under argon for 2 h. The mixture was stirred at rt for another 2 h. For **6a**, silica gel (5 g) adsorption of crude products was performed and was kept in air for 12 h at rt.

^*b*^Isolated yields. (ArO) = (2,4-di-*tert*-butylphenyl).

**Scheme 2:**
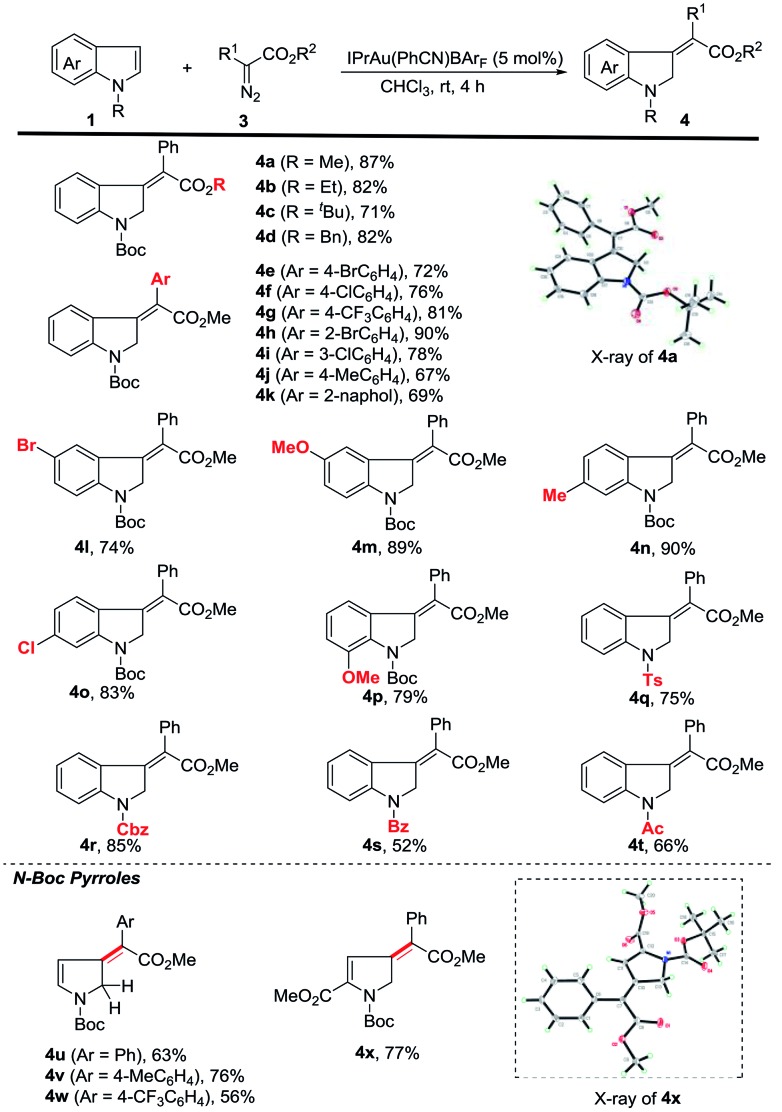
Substrate scope and reaction conditions: to a solution of 5 mol% of IPrAu(PhCN)BAr_F_ in CHCl_3_ (2 mL) a solution of **1** (0.2 mmol) and **3** (0.3 mmol) in 2 mL CHCl_3_ was added at rt for 2 h *via* a syringe pump under argon. The resulting mixture was stirred at rt for another 2 h. Isolated yields have been listed. 4 equiv. of the diazo substrate were used for the preparation of **4g**.

We then started to explore the substrate scope for the first dearomatization (Scheme 2). The reaction of indole **1a** with a variety of diazoesters was firstly examined. It was observed that methyl phenyl diazoacetate gave the corresponding product in better yield than other esters (**4a** to **4d**). Aromatic diazoesters with different substituents were employed, providing the corresponding 3-methyleneindolines in moderate to excellent yields (**4e** to **4k**). In general, electron-deficient aromatic diazoesters furnished the products (**4e** to **4i**) in higher yields than electron-rich substrates (**4j** to **4k**). Next, the scope of indoles was investigated. Gratifyingly, C5-, C6- and C7-substituted *N*-boc indoles bearing either electron-withdrawing or electron-donating groups were all tolerated, furnishing the corresponding indolines in moderate to excellent yields (**4l** to **4p**). Furthermore, different N-substituted indoles were also examined. The protecting groups such as tosyl (Ts), benzyloxycarbonyl (Cbz), benzoyl (Bz) and acetyl (Ac) were all amenable to the reaction and the corresponding products were obtained in acceptable yields (**4q** to **4t**). This protocol was also amenable to pyrroles. The desired dearomatization products were isolated in good yields (**4u** to **4x**). The structure of **4x** was further confirmed by single-crystal X-ray crystallography.^17^ It should be noted that all of the methylene derivatives were isolated in the single *Z*-configuration.

**Scheme 3:**
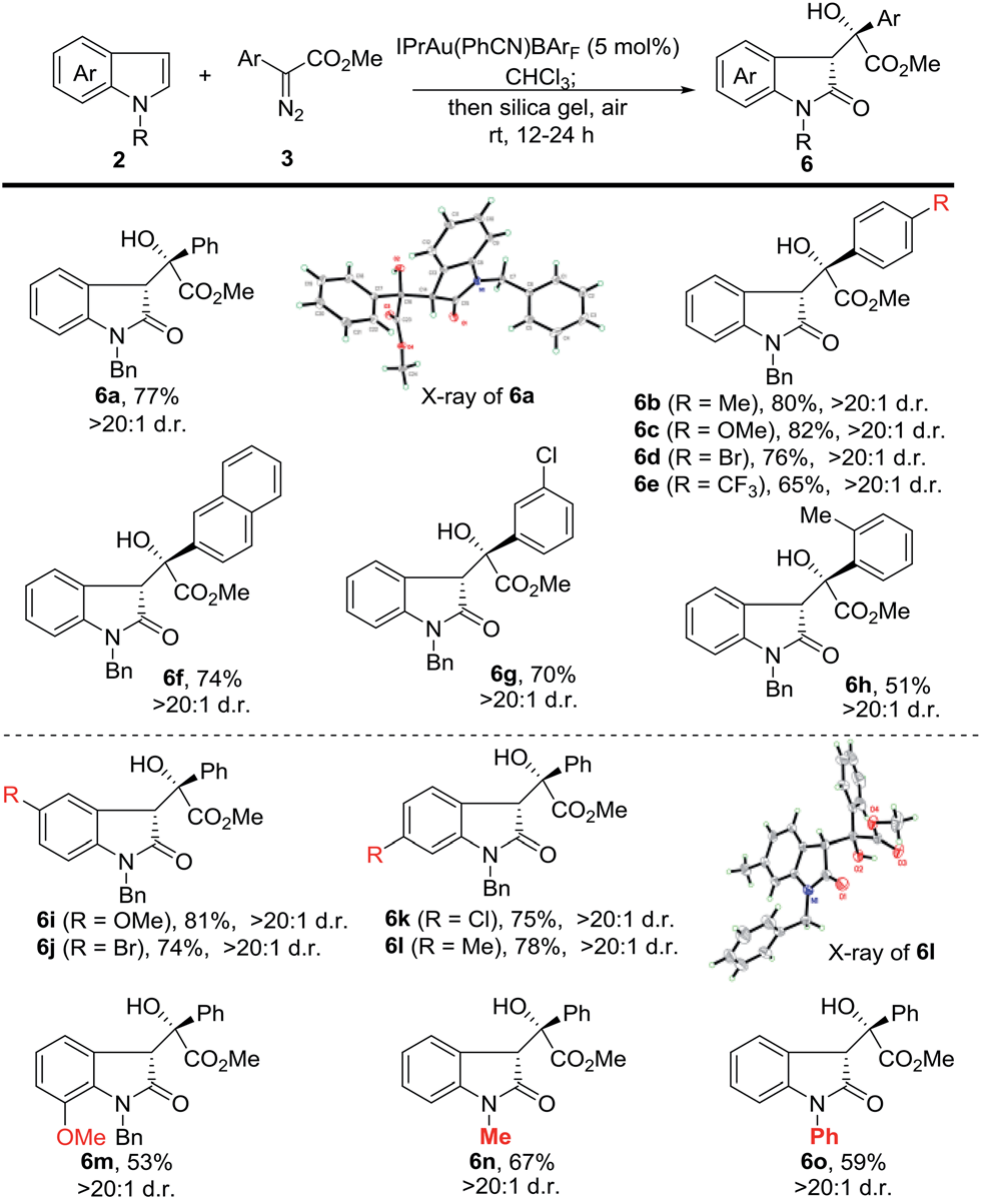
Tandem reaction of the dearomatization and aerobic oxidation. Reaction conditions: IPrAu(PhCN)BAr_F_ (5 mol%), **2** (0.2 mmol) and **3** (0.3 mmol) in 4 mL CHCl_3_ at rt for 4 h. Then SiO_2_ (5 g) adsorption in air at rt for 12 to 24 h. Isolated yields have been listed.

Next, we investigated the substrate scope of the tandem reaction towards the formation of 3-substituted indolin-2-ones (Scheme 3). Generally, the reaction of **2a** with aromatic diazoesters either bearing electron-donating or electron-withdrawing substituents proceeded smoothly to afford the corresponding products in moderate to good yields (**6a** to **6h**). A longer oxidation time (24 h) is needed for **6e** to **6h**. Afterwards, indoles bearing various substituents were examined. The use of *N*-benzyl 5-methoxy and 5-bromo indoles provided **6i** and **6j** in 81% and 74% yield, while 6-chloro and 6-methyl delivered the corresponding products in 75% and 78% yield, respectively. 7-Methoxy *N*-benzyl indole was also examined, and **6m** was isolated in 53% yield. Finally, *N*-methyl and *N*-phenyl indoles were tested and the corresponding products (**6n** and **6o**) were obtained in moderate yields. The structure of **6a** and **6l** was confirmed by X-ray analysis.

**Scheme 4:**
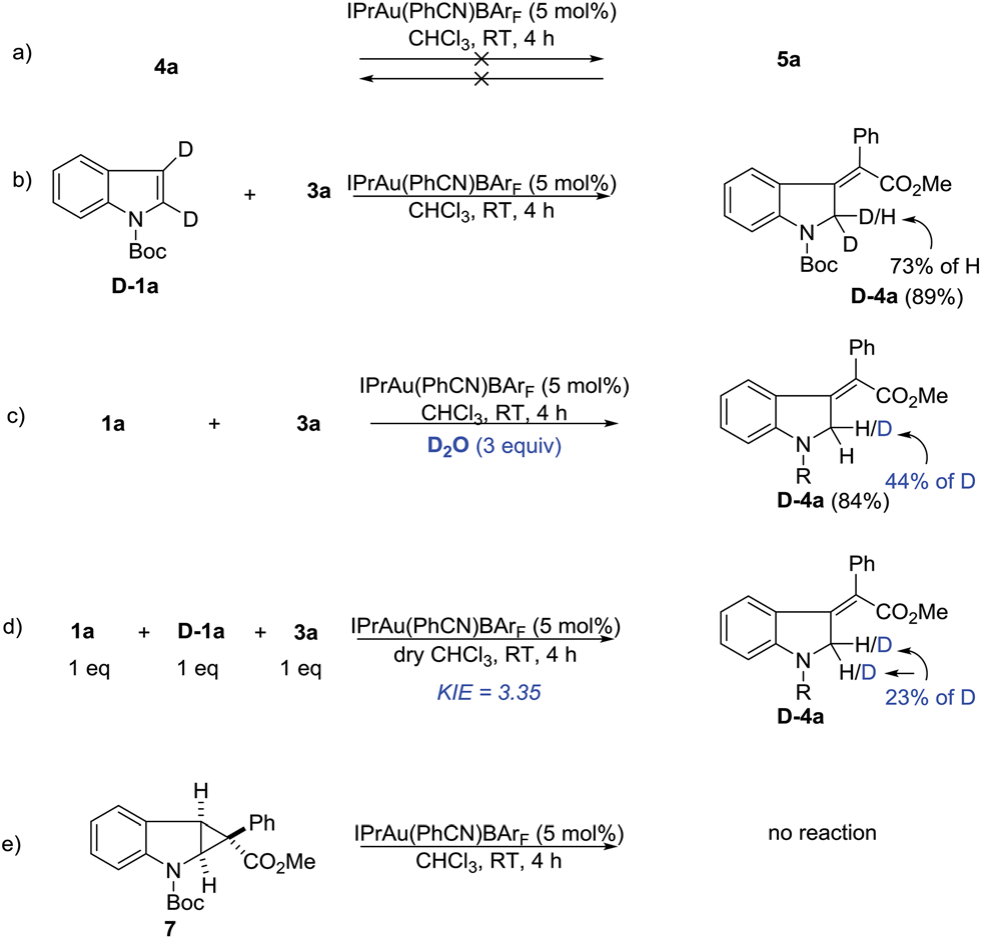
Mechanistic studies on the dearomatization reaction.

Deuterium labeling and control experiments were conducted to understand the reaction mechanism (Scheme 4). First, **4a** cannot be converted to **5a** under standard reaction conditions (Scheme 4a), which ruled out the possibility of preferential formation of **5** followed by isomerization and *vice versa*. Next, the reaction of **D-1a** with **3a** yielded **D-4a**. The high deuterium incorporation at the 2-position might indicate that a 1,2-hydrogen shift is likely involved in the reaction (Scheme 4b). Since there is significant loss of deuterium, we suspected that the reaction may be interfered with by adventitious water present in the reaction mixture. Thus, a control reaction with 3 equivalents of D_2_O was run. Indeed, significant deuterium incorporation into the product was observed (Scheme 4c), suggesting that there is D/H exchange with the adventitious proton source during the reaction’s progress. A study on the kinetic isotope effect (KIE) using intermolecular competition between **1a** and **D-1a** indicated a KIE value of 3.35 (Scheme 4d), which is consistent with the 1,2-hydrogen shift being the rate-determining step. Furthermore, compound **7** was separately prepared and subjected to standard reaction conditions. Unfortunately, no reaction was observed. The chemical incompetence of **7** rules out its possible role as an intermediate in this process (Scheme 4e).

**Scheme 5:**
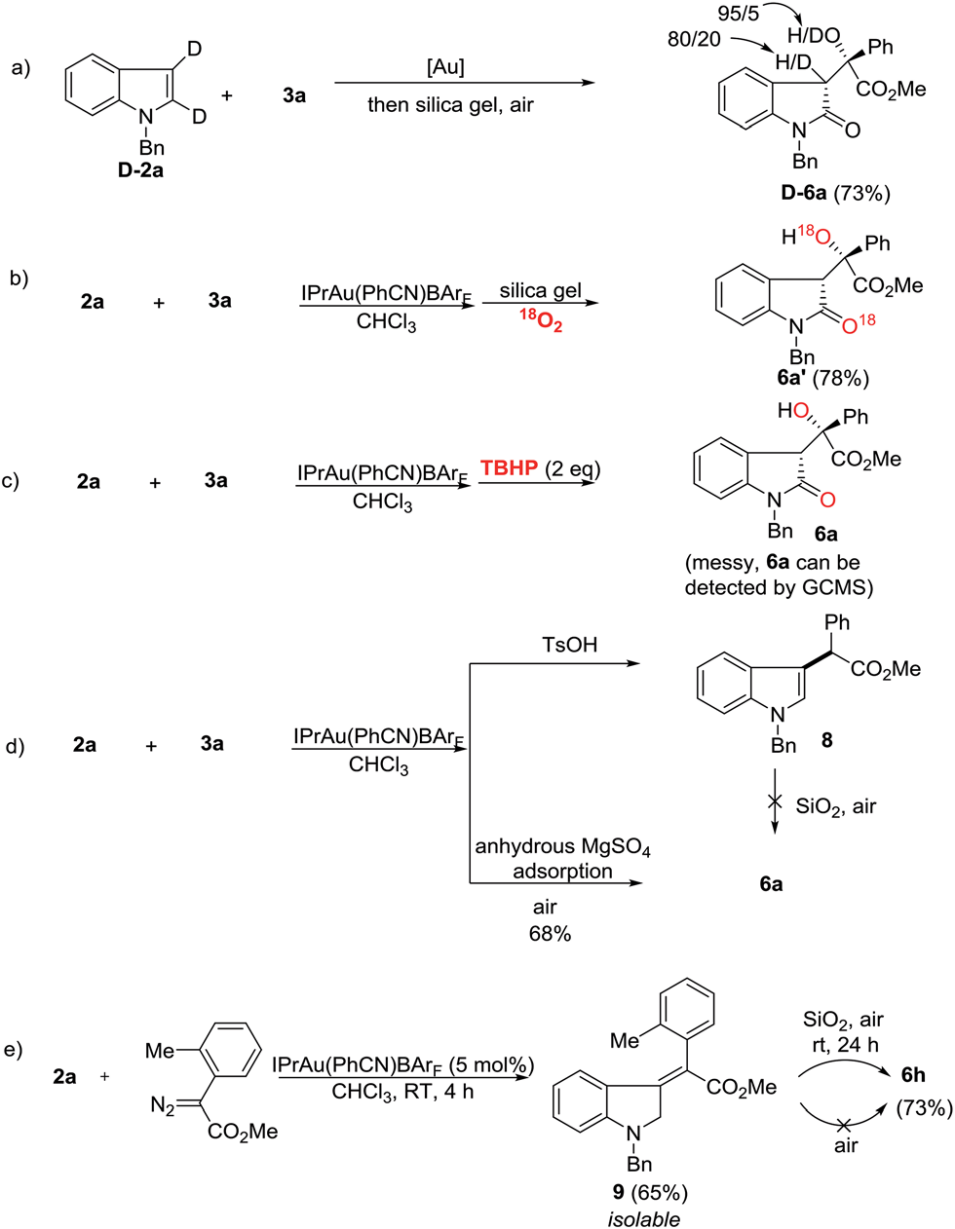
Mechanistic studies on the aerobic oxidation.

Next, mechanistic studies for the aerobic oxidation were carried out (Scheme 5). First, the reaction of **D-2a** with **3a** gave **D-6a** in 73% yield with a low ratio of deuterium labeling (Scheme 5a). Moreover, the ^18^O-labeled product **6a′** was obtained in 78% yield under an ^18^O_2_ atmosphere, indicating that the oxygen atom in **6** came from the air (Scheme 5b). Using TBHP (*tert*-butyl hydroperoxide) instead of air as an oxidant, the reaction was messy although **6a** could still be detected by GC/MS (Scheme 5c). To determine the role of the silica gel, *p*-toluene sulfonic acid was added and the C-3 alkylation product **8** was obtained in high yield (Scheme 5d). Moreover, by replacing the silica gel with anhydrous MgSO_4_, **6a** was isolated in 68% yield upon exposure to air for 12 h (Scheme 5d). Therefore, the acidic property of the silica gel may not be critically important for this transformation. Its role is presumably to facilitate the aerobic oxidation by increasing the contact surface of the olefin intermediate with oxygen. Just recently, López and co-workers reported the gold-catalyzed formal insertion of aryl diazoesters into ferrocene to generate functionalized metallocenes.^18^ They also found that the silica gel promoted the aerobic oxidation leading to tertiary-substituted ferrocenyl alcohols. In that case, they believed the aerobic oxidation might proceed through initial electron-transfer from iron to molecular oxygen, which triggered the radical sequence to give the target product. Thus, to determine the reactive intermediate for this oxidation process, the C3-alkylation product **8** was subjected to silica gel in air, however, no reaction occurred (Scheme 5d). Gratifyingly, although the reaction intermediates are extremely unstable, we successfully isolated intermediate **9** after carefully checking all of the reactions listed in Scheme 3. Furthermore, the treatment of **9** with silica gel in air provided **6h** in 73% yield (Scheme 5e). This result indicated that gold catalysts did not work during the aerobic oxidation. Without silica gel adsorption, **6h** cannot be obtained either. Different from López’s report, this aerobic oxidation does not involve metal participation.

**Scheme 6:**
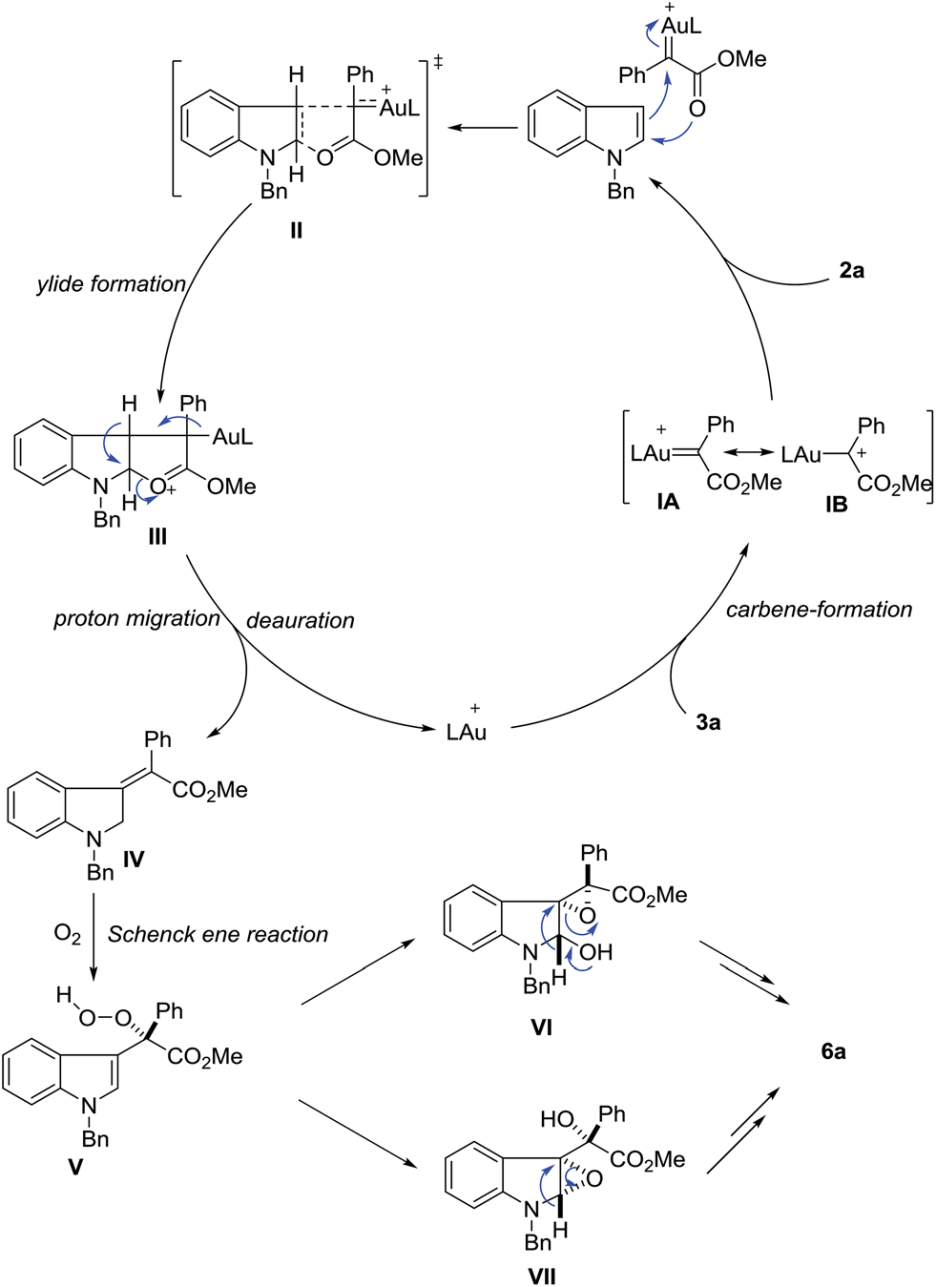
Proposed reaction mechanisms.

Until now, several reaction mechanisms have been proposed for indole functionalization with diazo compounds in the presence of rhodium, copper or other metal complexes.^2^–^8^ Clearly, this gold-catalyzed dearomatization is quite different from these known processes.^19^,^20^ Although the exact reaction mechanism is not clear at this moment, plausible ones have been proposed (Scheme 6). In view of the unique formation of *Z*-olefins, the carboxylate group may assist the olefin selectivity. Partially analogous to Fox’s description of rhodium-catalyzed C-3 alkylation of indoles with diazoesters3*d* and density functional theory (DFT) calculations reported by Xie *et al.*,3*f* the reaction of **3a** with the cationic gold catalyst first generates gold carbene species **IA** or **IB**, which is followed by nucleophilic attack with indole to produce ylide **III***via* transition state **II**. Then this ylide intermediate would undergo a 1,2-hydrogen shift to give the final dearomatization intermediate **IV**, together with catalyst regeneration. The assistance of the ester carbonyl group explains the *Z*-configuration of the observed product. Next, silica gel-assisted aerobic oxidation occurs. The reaction of **IV** with molecular oxygen generates intermediate **V***via* the Schenck ene reaction.^21^ Owing to the high oxidation ability of the peroxide motif and the electron-rich nature of the indole ring, subsequent internal epoxidation can rapidly take place *via* either **VI** or **VII**. Finally, semi-pinacol rearrangement of **VI**^22^ or rearrangement of the amino epoxide motif in **VII**^23^ leads to the observed amide **6**. Considering the high reactivity of peroxide **V**, the epoxidation step occurs on the same face of the indole plane once it is formed (C–C bond rotation is thus discouraged). In this scenario, the stereochemistry integrity determines the high diastereoselectivity observed in the final product.

## Conclusions

In summary, we have developed an unprecedented gold-catalyzed stereoselective dearomatization of indoles with diazoesters, providing 3-methyleneindolines in good to excellent yields with unique *Z*-configuration. Moreover, when N-donating substituent indoles were subjected to the reaction, a tandem reaction sequence occurred including the initial dearomatization and a sequential metal-free aerobic oxidation to produce 3-substituted indolin-2-ones. As a result, molecular oxygen has been successfully inlaid into the final structure. Notably, the use of the cationic gold(i) catalyst IPrAu(PhCN)BAr_F_ is crucial to the whole process.

## Conflicts of interest

There are no conflicts to declare.

## Supplementary Material

Supplementary information

Crystal structure data
