# Supplementary material for: Gold-catalyzed stereoselective dearomatization/metal-free aerobic oxidation: access to 3-substituted indolines/oxindoles
Source: Chem Sci. 2017 Nov 6;9(3):634–9. doi: 10.1039/c7sc04086e (PMC5868387; doi:10.1039/c7sc04086e)
Supplement: Supplementary file 1 [file SC-009-C7SC04086E-s001.pdf]

Electronic Supplementary Material (ESI) for Chemical Science.
This journal is © The Royal Society of Chemistry 2017

Gold-Catalyzed Stereoselective Dearomatization/Metal-Free Aerobic Oxidation: Access to 3-Substituted Indolines/Oxindoles

Kai Liu, Guangyang Xu and Jiangtao Sun*

Jiangsu Key Laboratory of Advanced Catalytic Materials & Technology, School of Petrochemical Engineering, Changzhou University

1 Gehu Road, 213164 Changzhou, China

E-mail: jtsun08@gmail.com or jtsun@cczu.edu.cn

Table of Contents

1. General information

2. Preparation of substrates

3. General procedure for Scheme 2

4. General procedure for Scheme 3

5. Mechanistic studies on the dearomatization reaction of Scheme 4

6. Mechanistic studies on the aerobic oxidation of Scheme 5

6. X-ray structure of **4a**, **4x**, **6a** and **6l**

7. References

8. ^{1}H NMR and ^{13}C NMR Spectra of compounds

General information

All of the reactions were carried out in flame-dried tubes under argon atmosphere. Solvents were dried prior to use. For column chromatography, 200-300 mesh silica gel was used. ^{1}H NMR were recorded on Bruker 300 MHz, 400 MHz or 500 MHz spectrometer and ^{13}C NMR were recorded on Bruker 75 MHz, 100 MHz or 125MHz spectrometer in $CDCl_3$ or acetone-d_6. HRMS were performed on Agilent 6540 Q-TOF mass spectrometer (ESI). Melting points were determined on a SGW X-4B melting point apparatus. The diazo compounds[1] and IPrAu(PhCN)BAr_F[2] were known compounds and prepared according to the literature procedures.

Preparation of substrates

The N-protected indoles were known compounds and prepared according to the literature procedures.[3]

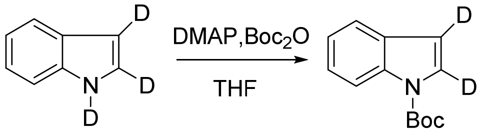

D-1a

To a solution of 1H-indole-1,2,3-d_3[4] (146 mg, 1.2 mmol) in dry THF (7 mL) was added DMAP (2 mg, 0.02 mmol) and Boc_2O (288 mg, 1.3 mmol) at 0 °C under an argon atmosphere. Then the reaction mixture was stirred at room temperature for 3 h under an argon atmosphere, after which it was concentrated and purified by column chromatography (silica gel, EtOAc/Petroleum ether = 1:100) to give **D-1a** (255 mg, 97%) as colorless oil. ^{1}H NMR (400 MHz, $CDCl_3$) δ 8.15 (d, J = 8.0 Hz, 1H), 7.56 (d, J = 8.0 Hz, 1H), 7.31 (t, J = 8.0 Hz, 1H), 7.22 (t, J = 8.0 Hz, 1H), 1.67 (s, 9H). ^{13}C NMR (100 MHz, $CDCl_3$) δ 149.84, 135.17, 130.52, 125.25, 124.20, 122.66, 120.94, 115.19, 107.18, 83.66, 28.24. HRMS (ESI) calcd. for $C_{13}H_{14}D_2NO_2$ $[M+H]^+$: 220.1301, found: 220.1303.

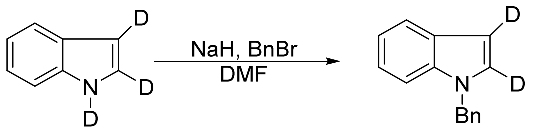

D-2a

To a suspension of 60% NaH (96 mg, 2.4 mmol) in dry DMF (5 mL) was added dropwise a solution of 1H-indole-1,2,3-d_3[4] (238 mg, 2 mmol) in dry DMF (2 mL) at 0 °C. The mixture was stirred for 30 min, then BnBr (410 mg, 2.4 mmol) was added dropwise. The reaction mixture was warmed to room temperature and stirred for 12 h. The reaction mixture was quenched with H_2O and extracted with ether; the combined organic layers were washed with brine, dried over anhydrous Na_2SO_4 and concentrated under vacuum; the residue was purified by column chromatography (silica gel, EtOAc/Petroleum ether = 1:30-1:15) to give **D-2a** as light yellow oil (313 mg, 75%). ^{1}H NMR (400 MHz, $CDCl_3$) δ 7.65 (d, *J* = 7.8 Hz, 1H), 7.32-7.23 (m, 4H), 7.17 (t, *J* = 7.4 Hz, 1H), 7.14-7.08 (m, 3H), 5.34 (s, 2H). ^{13}C NMR (100 MHz, $CDCl_3$) δ 137.61, 136.33, 128.82, 127.87, 127.65, 126.83, 121.73, 121.04, 121.01, 119.59, 109.76, 101.56, 50.10. HRMS (ESI) calcd. for $C_{15}H_{12}D_2N$ $[M+H]^+$: 210.1246, found: 210.1249.

General procedure for Scheme 2

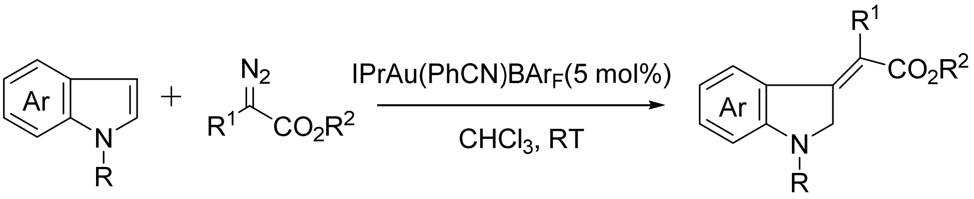

1 3 4

To a dry Schlenk tube was added IPrAu(PhCN)BAr$_F$ (5 mol%) and $CHCl_3$ (2 mL) under an argon atmosphere, then **1** (0.2 mmol, 1 eq.) and **3** (0.3 mmol, 1.5 eq.) in dry $CHCl_3$ (2 mL) was added via a syringe pump over 2 h under an argon atmosphere. After stirring for 2 h at rt, the reaction mixture was concentrated and purified by flash chromatography on silica gel (petroleum ether/ethyl acetate = 80:1) to give desired product **4**.

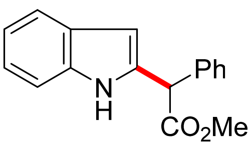

methyl 2-(1H-indol-2-yl)-2-phenylacetate (Sa)*[5]*:

This compound was prepared via general procedure as red oil (24 mg, yield: 46 %).

^{1}H NMR (400 MHz, $CDCl_3$) δ 8.74 (s, 1H), 7.55 (d, *J* = 8.0 Hz, 1H), 7.39-7.26 (m, 6H), 7.16 (t, *J* = 8.0 Hz, 1H), 7.08 (t, *J* = 8.0 Hz, 1H), 6.40 (s, 1H), 5.22 (s, 1H), 3.79 (s, 3H). ^{13}C NMR (125 MHz, $CDCl_3$) δ 172.38, 137.44, 136.28, 134.48, 128.86, 128.05, 127.97, 127.78, 121.98, 120.39, 119.92, 110.99, 102.27, 52.72, 50.60.

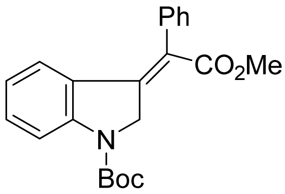

tert-butyl (Z)-3-(2-methoxy-2-oxo-1-phenylethylidene)indoline-1-carboxylate (4a):

This compound was prepared via general procedure as a white solid (64 mg, yield: 87%), mp: 189-191 °C.

^{1}H NMR (300 MHz, $CDCl_3$) δ 8.03 (s, 1H), 7.48-7.45 (m, 3H), 7.25-7.19 (m, 3H), 6.64-6.55 (m, 1H), 6.00 (d, *J* = 9.0 Hz, 1H), 5.14 (s, 2H), 3.71 (s, 3H), 1.61 (s, 9H). ^{13}C NMR (125 MHz, $CDCl_3$) δ 167.49, 151.40, 137.18, 132.05, 129.61, 129.24, 128.72,127.95, 127.35, 126.35, 121.88, 115.13, 81.52, 55.59, 51.96, 28.46. HRMS (ESI) calcd. for $C_{22}H_{24}NO_4$ $[M+H]^+$: 366.1700, found: 366.1702.

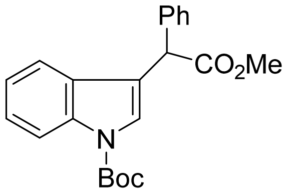

tert-butyl 3-(2-methoxy-2-oxo-1-phenylethyl)-1H-indole-1-carboxylate (5a):

This compound was prepared via general procedure using $Ph_3PAuCl/AgSbF_6$ instead of IPrAu(PhCN)BAr_F as catalyst and CH_2Cl_2 as solvent to afford as yellow oil (51 mg, yield: 70%). ^{1}H NMR (300 MHz, $CDCl_3$) δ 8.12 (d, *J* = 9.0 Hz, 1H), 7.60 (d, *J* = 3.0

Hz, 1H), 7.45-7.38 (m, 2H), 7.37-7.24 (m, 5H), 7.20-7.12 (m, 1H), 5.17 (s, 1H), 3.76 (s, 3H), 1.66 (s, 9H). ^{13}C NMR (75 MHz, $CDCl_3$) δ 172.54, 149.74, 137.27, 135.53, 129.49, 128.74, 128.47, 127.64, 124.60, 124.55, 122.64, 119.20, 118.04, 115.36, 83.85, 52.51, 48.69, 28.22. HRMS (ESI) calcd. for $C_{22}H_{24}O_4$ $[M+H]^+$: 366.1700, found: 366.1702.

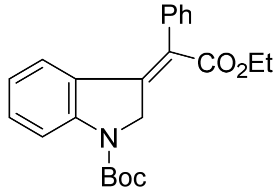

tert-butyl (Z)-3-(2-ethoxy-2-oxo-1-phenylethylidene)indoline-1-carboxylate (4b):

This compound was prepared via general procedure as a yellow solid (62 mg, yield: 82%), mp: 133-135 °C.

^{1}H NMR (400 MHz, $CDCl_3$) δ 8.03 (s, 1H), 7.51-7.39 (m, 3H), 7.25-7.18 (m, 3H), 6.59 (t, *J* = 8.0 Hz, 1H), 6.02 (d, *J* = 8.0 Hz, 1H), 5.13 (s, 2H), 4.20 (q, *J* = 8.0 Hz, 2H), 1.61 (s, 9H), 1.22 (t, *J* = 8.0 Hz, 3H). ^{13}C NMR (75 MHz, $CDCl_3$) δ 167.09, 151.39, 147.55, 137.29, 131.91, 129.60, 129.13, 127.83, 127.45, 126.35, 122.20, 121.87, 115.08, 81.45, 60.63, 55.56, 28.46, 14.29. HRMS (ESI) calcd. for $C_{23}H_{26}NO_4$ $[M+H]^+$: 380.1856, found: 380.1857.

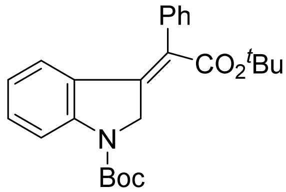

tert-butyl (Z)-3-(2-(tert-butoxy)-2-oxo-1-phenylethylidene)indoline-1-carboxylate (4c):

This compound was prepared via general procedure as a yellow solid (58 mg, yield: 71%), mp: 133-135 °C.

^{1}H NMR (400 MHz, $CDCl_3$) δ 8.02 (s, 1H), 7.49-7.37 (m, 3H), 7.20 (d, *J* = 8.0 Hz, 3H), 6.58 (t, *J* = 8.0 Hz, 1H), 6.05 (d, *J* =8.0 Hz, 1H), 5.07 (s, 2H), 1.60 (s, 9H), 1.49 (s, 9H). ^{13}C NMR (125 MHz, $CDCl_3$) δ 166.43, 151.40, 137.83, 131.56, 129.53,

129.00, 128.41, 127.64, 126.30, 124.29, 121.85, 114.97, 81.07, 55.51, 28.48, 28.27. HRMS (ESI) calcd. for $C_{25}H_{30}NO_4$ $[M+H]^+$: 408.2169, found: 408.2165.

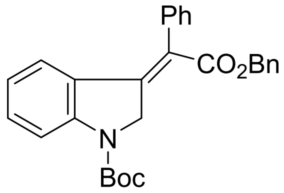

tert-butyl (Z)-3-(2-(benzyloxy)-2-oxo-1-phenylethylidene)indoline-1-carboxylate (4d):

This compound was prepared via general procedure as a yellow solid (72 mg, yield: 82%), mp: 90-92 °C.

^{1}H NMR (400 MHz, $CDCl_3$) δ 8.04 (s, 1H), 7.49-7.42 (m, 3H), 7.33-7.21 (m, 6H), 7.17 (d, *J* = 8.0 Hz, 2H), 6.60 (t, *J* = 8.0 Hz, 1H), 6.07 (d, *J* = 8.0 Hz, 1H), 5.20 (s, 2H), 5.14 (s, 2H), 1.59 (s, 9H). ^{13}C NMR (75 MHz, $CDCl_3$) δ 166.67, 151.37, 137.15, 136.43, 132.10, 129.63, 129.20, 129.13, 128.60, 128.49, 128.40, 127.91, 127.72, 127.22, 127.07, 126.41, 121.90, 115.13, 80.92, 65.89, 55.57, 28.46. HRMS (ESI) calcd. for $C_{28}H_{28}NO_4$ $[M+H]^+$: 442.2013, found: 442.2016.

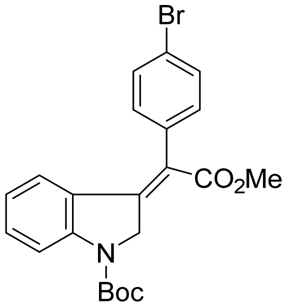

tert-butyl (Z)-3-(1-(4-bromophenyl)-2-methoxy-2-oxoethylidene)indoline-1-carboxylate (4e):

This compound was prepared via general procedure as a white solid (64 mg, yield: 72%), mp: 181-183 °C.

^{1}H NMR (400 MHz, $CDCl_3$) δ 8.06 (s, 1H), 7.60 (d, *J* = 8.0 Hz, 2H), 7.28 (d, *J* = 8.0 Hz, 1H), 7.11 (d, *J* = 8.0 Hz, 2H), 6.67 (t, *J* = 8.0 Hz, 1H), 6.13 (d, *J* = 8.0 Hz, 1H), 5.13 (s, 2H), 3.71 (s, 3H), 1.61 (s, 9H). ^{13}C NMR (75 MHz, $CDCl_3$) δ 167.04, 151.31,

136.12, 132.49, 132.39, 132.22, 131.53, 129.38, 126.20, 122.10, 122.02, 115.29, 81.74, 55.62, 52.02, 28.44. HRMS (ESI) calcd. for $C_{22}H_{23}BrNO_4$ $[M+H]^+$: 444.0805, found: 444.0806.

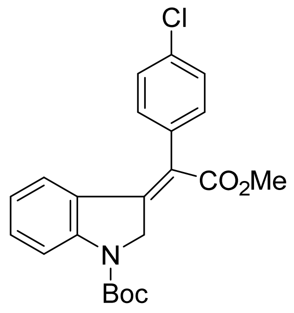

tert-butyl (Z)-3-(1-(4-chlorophenyl)-2-methoxy-2-oxoethylidene)indoline-1-carboxylate (4f):

This compound was prepared via general procedure as a yellow solid (61 mg, yield: 76%), mp: 180-181 °C.

^{1}H NMR (400 MHz, $CDCl_3$) δ 8.06 (s, 1H), 7.45 (d, *J* = 8.0 Hz, 2H), 7.27 (t, *J* = 8.0 Hz, 1H), 7.17 (d, *J* = 8.0 Hz, 2H), 6.66 (t, *J* = 8.0 Hz, 1H), 6.13 (d, *J* = 8.0 Hz, 1H), 5.13 (s, 2H), 3.71 (s, 3H), 1.61 (s, 9H). ^{13}C NMR (75 MHz, $CDCl_3$) δ 167.12, 151.32, 148.90, 135.63, 133.92, 132.37, 131.20, 129.54, 129.43, 127.00, 126.20, 121.99, 120.18, 115.28, 81.68, 55.62, 52.01, 28.44. HRMS (ESI) calcd. for $C_{22}H_{23}ClNO_4$ $[M+H]^+$: 400.1310, found: 400.1308.

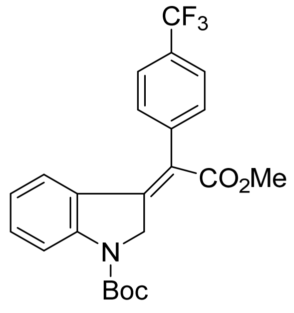

tert-butyl (Z)-3-(2-methoxy-2-oxo-1-(4-(trifluoromethyl)phenyl)ethylidene)indoline -1-carboxylate (4g):

This compound was prepared via general procedure as a white solid (70 mg, yield: 81%), mp: 202-204 °C.

^{1}H NMR (400 MHz, $CDCl_3$) δ 8.07 (s, 1H), 7.73 (d, *J* = 8.0 Hz, 2H), 7.37 (d, *J* = 8.0 Hz, 2H), 7.28 (d, *J* = 8.0 Hz, 1H), 6.63 (t, *J* = 8.0Hz, 1H), 6.00 (d, *J* = 8.0 Hz, 1H), 5.16 (s, 2H), 3.71 (s, 3H), 1.61 (s, 9H). ^{13}C NMR (75 MHz, $CDCl_3$) δ 166.84, 151.29, 141.10, 132.55, 130.30, 129.90, 126.25, 126.20, 126.15, 126.10, 126.02, 122.37, 122.03, 115.37, 81.80, 55.65, 52.04, 28.44. HRMS (ESI) calcd. for $C_{23}H_{23}F_3NO_4$ $[M+H]^+$: 434.1574, found: 434.1578.

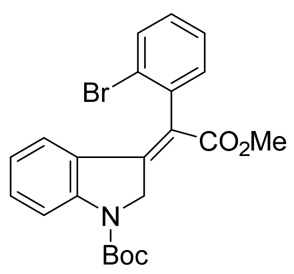

tert-butyl (Z)-3-(1-(2-bromophenyl)-2-methoxy-2-oxoethylidene)indoline-1-carboxylate (4h):

This compound was prepared via general procedure as a white solid (80 mg, yield: 90%), mp: 147-149 °C.

^{1}H NMR (400 MHz, $CDCl_3$) δ 8.07 (s, 1H), 7.72 (d, *J* = 8.0 Hz, 1H), 7.41 (t, *J* = 8.0 Hz, 1H), 7.35-7.22 (m, 3H), 6.64 (t, *J* = 7.3 Hz, 1H), 6.00 (d, *J* = 8.0 Hz, 1H), 5.28-5.10 (m, 2H), 3.72 (s, 3H), 1.61 (s, 9H). ^{13}C NMR (125 MHz, $CDCl_3$) δ 166.60, 151.32, 149.25, 147.90, 137.95, 133.22, 132.47, 131.34, 129.68, 128.28, 126.91, 125.65, 124.87, 122.21, 120.42, 115.29, 81.56, 55.51, 52.04, 28.45. HRMS (ESI) calcd. for $C_{22}H_{23}BrNO_4$ $[M+H]^+$: 444.0805, found: 444.0803.

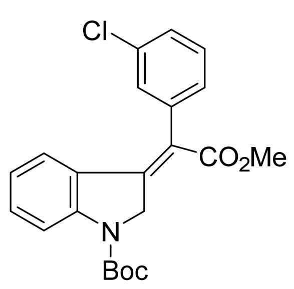

tert-butyl (Z)-3-(1-(3-chlorophenyl)-2-methoxy-2-oxoethylidene)indoline-1-carboxylate (4i):

This compound was prepared via general procedure as a yellow solid (62 mg, yield: 78%), mp: 160-162 °C.

^{1}H NMR (400 MHz, $CDCl_3$) δ 8.06 (s, 1H), 7.46-7.36 (m, 2H), 7.30-7.23 (m, 2H), 7.12 (d, J = 8.0 Hz, 1H), 6.65 (t, J = 8.0 Hz, 1H), 6.09 (d, J = 8.0 Hz, 1H), 5.14 (s, 2H), 3.71 (s, 3H), 1.61 (s, 9H). ^{13}C NMR (75 MHz, $CDCl_3$) δ 166.98, 151.31, 149.11, 147.80, 138.96, 134.91, 132.43, 130.50, 129.86, 128.17, 127.99, 126.89, 126.24, 122.04, 120.05, 115.29, 81.75, 55.59, 52.05, 28.44. HRMS (ESI) calcd. for $C_{22}H_{23}ClNO_4$ $[M+H]^+$: 400.1310, found: 400.1313.

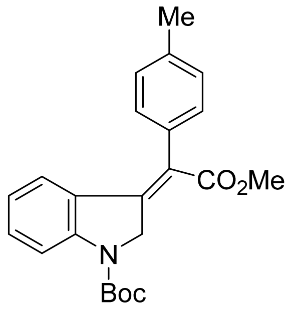

tert-butyl (Z)-3-(2-methoxy-2-oxo-1-(p-tolyl)ethylidene)indoline-1-carboxylate (4j):

This compound was prepared via general procedure as a white solid (54 mg, yield: 67%), mp: 149-151 °C.

^{1}H NMR (400 MHz, $CDCl_3$) δ 8.04 (s, 1H), 7.28-7.21 (m, 3H), 7.11 (d, J = 4.0 Hz, 2H), 6.62 (t, J = 8.0 Hz, 1H), 6.08 (d, J = 8.0 Hz, 1H), 5.13 (s, 2H), 3.71 (s, 3H), 2.45 (s, 3H), 1.60 (s, 9H). ^{13}C NMR (125 MHz, $CDCl_3$) δ 167.66, 151.42, 148.33, 147.68, 137.65, 134.09, 131.95, 130.00, 129.41, 127.47, 126.39, 121.86, 115.10, 81.47, 55.62, 51.96, 28.47, 21.47. HRMS (ESI) calcd. for $C_{23}H_{25}NNaO_4$ $[M+Na]^+$: 402.1676, found: 402.1677.

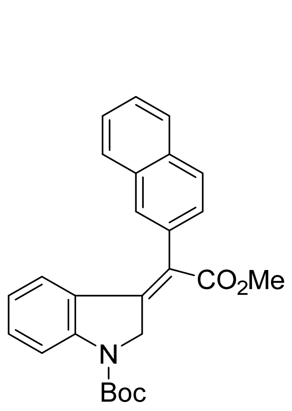

tert-butyl (Z)-3-(2-methoxy-1-(naphthalen-2-yl)-2-oxoethylidene)indoline-1-carboxylate (4k):

This compound was prepared via general procedure as a yellow solid (57 mg, yield: 69%), mp: 160-162 °C.

^{1}H NMR (400 MHz, $CDCl_3$) δ 8.04 (s, 1H), 7.97-7.90 (m, 2H), 7.83 (d, *J* = 8.0 Hz, 1H), 7.73 (s, 1H), 7.59-7.48 (m, 2H), 7.34 (d, *J* = 8.0 Hz, 1H), 7.20 (t, *J* = 8.0 Hz, 1H), 6.47 (t, *J* = 8.0 Hz, 1H), 6.03 (d, *J* = 8.0 Hz, 1H), 5.20 (s, 2H), 3.69 (s, 3H), 1.62 (s, 9H). ^{13}C NMR (75 MHz, $CDCl_3$) δ 167.58, 151.42, 148.67, 147.74, 134.54, 133.85, 132.85, 132.11, 128.95, 128.56, 128.21, 127.91, 127.76, 127.31, 126.40, 126.29, 126.20, 121.97, 121.44, 115.16, 81.62, 55.71, 51.98, 28.48. HRMS (ESI) calcd. for $C_{26}H_{26}NO_4$ $[M+H]^+$: 416.1856, found: 416.1858.

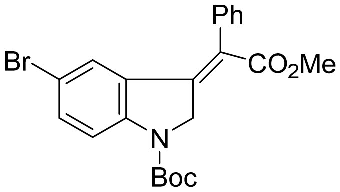

tert-butyl (Z)-5-bromo-3-(2-methoxy-2-oxo-1-phenylethylidene)indoline-1-carboxylate (4l):

This compound was prepared via general procedure as a yellow solid (66 mg, yield: 74%), mp: 206-208 °C.

^{1}H NMR (400 MHz, $CDCl_3$) δ 7.92 (s, 1H), 7.54-7.45 (m, 3H), 7.31 (d, *J* = 8.0 Hz, 1H), 7.20 (d, *J* = 8.0 Hz, 2H), 5.97 (s, 1H), 5.14 (s, 2H), 3.72 (s, 3H), 1.60 (s, 9H). ^{13}C NMR (125 MHz, $CDCl_3$) δ 167.18, 151.19, 146.55, 136.48, 134.49, 129.43, 129.32, 129.22, 129.12, 128.30, 128.18, 122.91, 116.45, 114.48, 81.93, 55.71, 52.08, 28.42. HRMS (ESI) calcd. for $C_{22}H_{23}BrNO_4$ $[M+H]^+$: 444.0805, found: 444.0804.

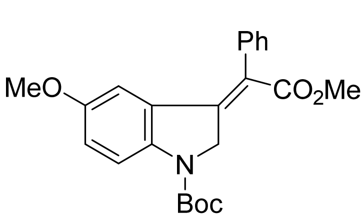

tert-butyl (Z)-5-methoxy-3-(2-methoxy-2-oxo-1-phenylethylidene)indoline-1-

carboxylate (4m):

This compound was prepared via general procedure as a yellow solid (70 mg, yield: 89%), mp: 167-169 °C.

^{1}H NMR (400 MHz, $CDCl_3$) δ 7.95 (s, 1H), 7.54-7.46 (m, 2H), 7.45-7.38 (m, 1H), 7.31-7.20 (m, 2H), 6.83 (d, *J* = 8.0 Hz, 1H), 5.53 (s, 1H), 5.14 (s, 2H), 3.72 (s, 3H), 3.27 (s, 3H), 1.60 (s, 9H). ^{13}C NMR (125 MHz, $CDCl_3$) δ 167.38, 154.44, 151.35, 148.68, 142.19, 137.17, 129.98, 129.21, 129.10, 127.93, 121.23, 120.33, 115.89, 109.05, 81.23, 55.86, 54.90, 51.95, 28.49. HRMS (ESI) calcd. for $C_{23}H_{26}NO_5$ $[M+H]^+$: 396.1805, found: 396.1803.

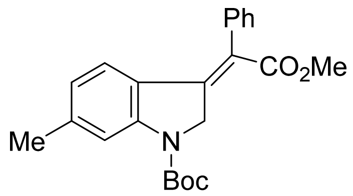

tert-butyl (Z)-3-(2-methoxy-2-oxo-1-phenylethylidene)-6-methylindoline-1-carboxylate (4n):

This compound was prepared via general procedure as a white solid (68 mg, yield: 90%), mp: 150-152 °C.

^{1}H NMR (400 MHz, $CDCl_3$) δ 7.90 (s, 1H), 7.49-7.40 (m, 3H), 7.22 (d, *J* = 4.0 Hz, 2H), 6.43 (d, *J* = 8.0 Hz, 1H), 5.86 (d, *J* = 8.0 Hz, 1H), 5.13 (s, 2H), 3.70 (s, 3H), 2.28 (s, 3H), 1.61 (s, 9H). ^{13}C NMR (75 MHz, $CDCl_3$) δ 167.58, 151.48, 148.53, 147.85, 143.19, 137.37, 129.70, 129.19, 127.85, 126.00, 124.92, 123.20, 120.42, 115.55, 81.51, 55.90, 51.90, 28.46, 22.03. HRMS (ESI) calcd. for $C_{23}H_{26}NO_4$ $[M+H]^+$: 380.1856, found: 380.1857.

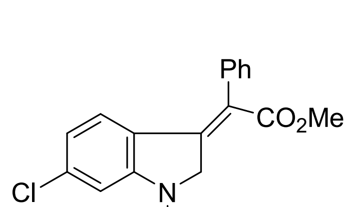

tert-butyl (Z)-6-chloro-3-(2-methoxy-2-oxo-1-phenylethylidene)indoline-1-carboxylate (4o):

This compound was prepared via general procedure as a white solid (66 mg, yield: 83%), mp: 178-180 °C.

^{1}H NMR (400 MHz, CDCl$_3$) δ 8.09 (s, 1H), 7.50-7.42 (m, 3H), 7.20 (d, *J* = 8.0 Hz, 2H), 6.56 (d, *J* = 8.0 Hz, 1H), 5.86 (d, *J* = 8.0 Hz, 1H), 5.15 (s, 2H), 3.71 (s, 3H), 1.61 (s, 9H). ^{13}C NMR (75 MHz, CDCl$_3$) δ 167.33, 151.17, 138.17, 136.83, 129.43, 129.35, 128.13, 126.93, 125.88, 122.28, 121.97, 115.36, 82.05, 55.93, 52.04, 28.39. HRMS (ESI) calcd. for $C_{22}H_{23}ClNO_4$ [M+H]$^+$: 400.1310, found: 400.1311.

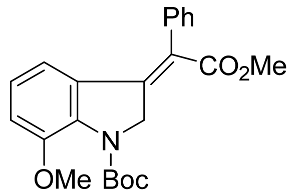

tert-butyl (Z)-7-methoxy-3-(2-methoxy-2-oxo-1-phenylethylidene)indoline-1-carboxylate (4p):

This compound was prepared via general procedure as a yellow solid (62 mg, yield: 79%), mp: 152-154 °C.

^{1}H NMR (400 MHz, CDCl$_3$) δ 7.49-7.39 (m, 3H), 7.25-7.20 (m, 2H), 6.82 (d, *J* = 8.0 Hz, 1H), 6.65 (t, *J* = 8.0 Hz, 1H), 5.64 (d, *J* = 8.0 Hz, 1H), 5.19 (s, 2H), 3.85 (s, 3H), 3.71 (s, 3H), 1.55 (s, 9H). ^{13}C NMR (75 MHz, CDCl$_3$) δ 167.47, 152.55, 149.55, 149.52, 137.38, 137.04, 131.31, 129.68, 129.07, 127.95, 124.16, 122.35, 118.44, 115.07, 81.15, 58.17, 56.03, 52.01, 28.23. HRMS (ESI) calcd. for $C_{23}H_{26}NO_5$ [M+H]$^+$: 396.1805, found: 396.1804.

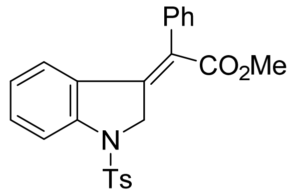

methyl (Z)-2-phenyl-2-(1-tosylindolin-3-ylidene)acetate (4q):

This compound was prepared via general procedure as a white solid (63 mg, yield: 75%), mp: 183-185 °C.

^{1}H NMR (400 MHz, CDCl$_3$) δ 7.83-7.72 (m, 3H), 7.46-7.38 (m, 3H), 7.30-7.21 (m,

3H), 7.17-7.08 (m, 2H), 6.62 (t, J = 8.0 Hz, 1H), 5.93 (d, J = 8.0 Hz, 1H), 5.12 (s, 2H), 3.69 (s, 3H), 2.38 (s, 3H). ^{13}C NMR (75 MHz, $CDCl_3$) δ 167.25, 147.01, 146.84, 144.49, 136.58, 134.04, 132.12, 129.91, 129.38, 129.24, 128.14, 127.82, 127.26, 126.74, 123.03, 122.73, 114.36, 56.77, 52.11, 21.61. HRMS (ESI) calcd. for $C_{24}H_{22}NO_4S$ [M+H]$^+$: 420.1264, found: 420.1263.

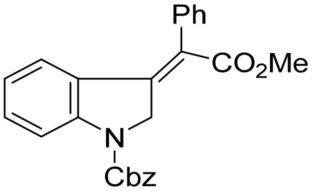

benzyl (Z)-3-(2-methoxy-2-oxo-1-phenylethylidene)indoline-1-carboxylate (4r):

This compound was prepared via general procedure as a yellow solid (68 mg, yield: 85%), mp: 135-137 °C.

^{1}H NMR (300 MHz, $CDCl_3$) δ 8.07 (s, 1H), 7.53-7.26 (m, 9H), 7.25-7.20 (m, 2H), 6.63 (t, J = 9.0 Hz, 1H), 6.01 (d, J = 9.0 Hz, 1H), 5.34 (s, 2H), 5.24 (s, 2H), 3.70 (s, 3H). ^{13}C NMR (75 MHz, $CDCl_3$) δ 167.45, 152.01, 147.98, 147.29, 136.96, 136.19, 132.15, 129.55, 129.29, 128.66, 128.33, 128.25, 128.05, 127.92, 127.40, 126.35, 122.42, 115.26, 67.32, 55.33, 52.03. HRMS (ESI) calcd. for $C_{25}H_{22}NO_4$ [M+H]$^+$: 400.1543, found: 400.1541.

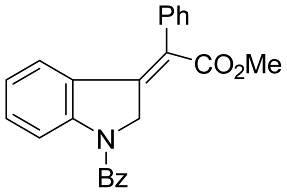

methyl (Z)-2-(1-benzoylindolin-3-ylidene)-2-phenylacetate (4s):

This compound was prepared via general procedure as a white solid (38 mg, yield: 52%), mp: 221-223 °C.

^{1}H NMR (400 MHz, $CDCl_3$) δ 8.21 (s, 1H), 7.63 (d, J = 4.0 Hz, 2H), 7.55-7.42 (m, 6H), 7.31-7.19 (m, 3H), 6.72 (t, J = 8.0 Hz, 1H), 6.07 (d, J = 8.0 Hz, 1H), 5.24 (s, 2H), 3.63 (s, 3H). ^{13}C NMR (75 MHz, $CDCl_3$) δ 168.97, 167.36, 147.70, 147.55, 136.76, 136.55, 131.96, 130.78, 129.49, 129.31, 128.83, 128.38, 128.13, 127.10, 126.32,

123.74, 122.12, 117.55, 58.02, 52.05. HRMS (ESI) calcd. for $C_{24}H_{20}NO_3$ $[M+H]^+$: 370.1438, found: 370.1439.

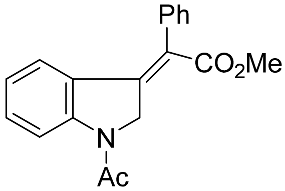

methyl (Z)-2-(1-acetylindolin-3-ylidene)-2-phenylacetate (4t):

This compound was prepared via general procedure as a white solid (41 mg, yield: 66%), mp: 193-195 °C.

^{1}H NMR (400 MHz, $CDCl_3$) δ 8.41 (d, *J* = 8.0 Hz, 1H), 7.53-7.43 (m, 3H), 7.29 (d, *J* = 8.0 Hz, 1H), 7.24 (d, *J* = 8.0 Hz, 2H), 6.69 (t, *J* = 8.0 Hz, 1H), 6.04 (d, *J* = 8.0 Hz, 1H), 5.26 (s, 2H), 3.72 (s, 3H), 2.40 (s, 3H). ^{13}C NMR (75 MHz, $CDCl_3$) δ 170.76, 168.46, 147.80, 147.53, 136.64, 132.33, 129.49, 129.33, 128.15, 127.61, 126.16, 123.33, 121.93, 117.18, 56.44, 52.10, 24.44. HRMS (ESI) calcd. for $C_{19}H_{18}NO_3$ $[M+H]^+$: 308.1281, found: 308.1285.

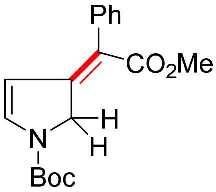

tert-butyl (Z)-3-(2-methoxy-2-oxo-1-phenylethylidene)-2,3-dihydro-1H-pyrrole-1-carboxylate (4u):

This compound was prepared via general procedure as a white solid (40 mg, yield: 63%), mp: 101-103 °C.

The compound is a mixture of conformers in $CDCl_3$. ^{1}H NMR (400 MHz, $CDCl_3$) δ 7.42-7.33 (m, 2.6H), 7.32-7.27 (m, 1H), 7.24-7.12 (m, 2.4H), 4.58 (s, 0.6H), 4.51 (s, 0.4H), 4.96 (s, 2H), 3.70 (s, 3H), 1.54 (s, 9H). ^{13}C NMR (100 MHz, $CDCl_3$) δ 167.44, 157.12, 150.35, 142.41, 137.02, 130.35, 128.11, 127.12, 115.97, 110.29, 82.04, 54.28, 51.58, 28.31. ^{1}H NMR (400 MHz, DMSO-d_6) δ 7.52-7.34 (m, 3H), 7.33-7.26 (m, 1H), 7.18 (d, *J* = 8.0 Hz, 2H), 5.45 (s, 1H), 4.85 (s, 2H), 3.60 (s, 3H), 1.49 (s, 9H). ^{13}C

NMR (100 MHz, DMSO-d_6) δ 166.87, 156.85, 155.87, 143.54, 137.28, 130.64, 128.53, 127.44, 115.94, 110.22, 81.93, 54.23, 51.76, 28.29. HRMS (ESI) calcd. for $C_{18}H_{22}NO_4$ $[M+H]^+$: 316.1543, found: 316.1541.

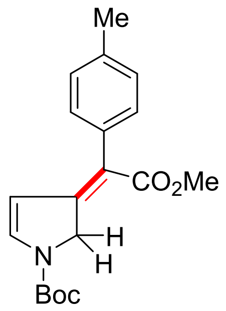

tert-butyl (Z)-3-(2-methoxy-2-oxo-1-(p-tolyl)ethylidene)-2,3-dihydro-1H-pyrrole-1-carboxylate (4v):

This compound was prepared via general procedure as a white solid (50 mg, yield: 76%), mp: 104-106 °C.

The compound is a mixture of conformers in $CDCl_3$. ^1H NMR (400 MHz, $CDCl_3$) δ 7.37 (s, 0.7H), 7.21-7.12 (m, 2.3H), 7.10 (d, J = 4.0 Hz, 2H), 5.59 (s, 0.7H), 5.53 (s, 0.3H), 4.94 (s, 2H), 3.70 (s, 3H), 2.37 (s, 3H), 1.54 (s, 9H). ^{13}C NMR (125 MHz, $CDCl_3$) δ 167.60, 156.92, 150.37, 142.16, 136.77, 133.99, 130.17, 128.85, 115.89, 110.42, 81.98, 54.25, 51.56, 28.30, 21.26. HRMS (ESI) calcd. for $C_{19}H_{24}NO_4$ $[M+H]^+$: 330.1700, found: 330.1701.

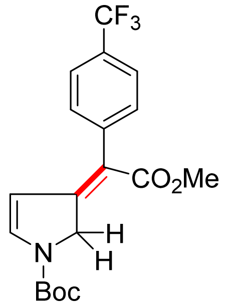

tert-butyl (Z)-3-(2-methoxy-2-oxo-1-(4-(trifluoromethyl)phenyl)ethylidene)-2,3-dihydro-1H-pyrrole-1-carboxylate (4w):

This compound was prepared via general procedure as a yellow solid (43 mg, yield: 56%), mp: 116-117 °C.

The compound is a mixture of conformers in $CDCl_3$.^{1}H NMR (400 MHz, $CDCl_3$) δ 7.62 (d, J = 8.0 Hz, 2H), 7.45 (s, 0.6H), 7.34 (d, J = 8.0 Hz, 2H), 7.22 (s, 0.3H), 5.54 (s, 0.7H), 5.48 (s, 0.3H), 4.98 (s, 2H), 3.71 (s, 3H), 1.55 (s, 9H). ^{13}C NMR (125 MHz, $CDCl_3$) δ 166.81, 158.04, 150.15, 143.52, 142.72, 140.88, 130.80, 129.30, 129.04, 127.47, 125.30, 125.13, 125.11, 125.08, 125.05, 123.14, 109.57, 82.31, 54.42, 51.65, 28.27. HRMS (ESI) calcd. for $C_{19}H_{21}F_3NO_4$ $[M+H]^+$: 384.1417, found: 384.1419.

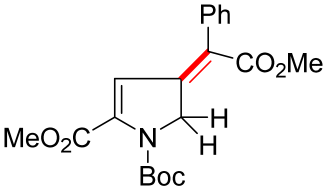

1-(tert-butyl) 2-methyl (Z)-4-(2-methoxy-2-oxo-1-phenylethylidene)-4,5-dihydro-1H -pyrrole-1,2-dicarboxylate (4x):

This compound was prepared via general procedure as a yellow solid (57 mg, yield: 77%), mp: 110-112 °C.

^{1}H NMR (400 MHz, $CDCl_3$) δ 7.41-7.29 (m, 3H), 7.19 (d, J = 8.0 Hz, 2H), 5.87 (s, 1H), 5.06 (s, 2H), 3.82 (s, 3H), 3.72 (s, 3H), 1.51 (s, 9H). ^{13}C NMR (125 MHz, $CDCl_3$) δ 166.99, 162.52, 152.93, 150.11, 145.50, 136.30, 130.17, 128.21, 127.54, 120.18, 115.03, 82.75, 55.72, 52.69, 51.83, 28.13. HRMS (ESI) calcd. for $C_{20}H_{24}NO_6$ $[M+H]^+$: 374.1598, found: 374.1599.

General procedure for Scheme 3

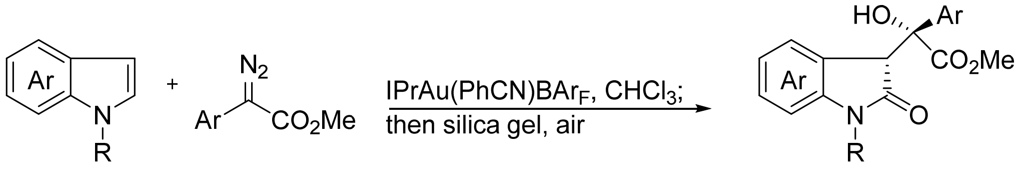

2 **3** **6**

To a dry Schlenk tube was added IPrAu(PhCN)BAr_F (5 mol%) and $CHCl_3$ (2 mL) under an argon atmosphere, then **2** (0.2 mmol, 1 eq.) and **3** (0.3 mmol, 1.5 eq.) in dry $CHCl_3$ (2 mL) was added via a syringe pump over 2 h under an argon atmosphere. The resulting solution was stirred at RT for another 2 h. The reaction mixture was

adsorbed on silica gel (5 g) by removing the solvent under vacuum; the silica gel powder was spread on a paper and exposed under air for 12-24 h. The yellow or orange color of the silica gel was disappeared. The crude product was eluted with EtOAc from the silica gel, concentrated and purified by column chromatography (silica gel, EtOAc/Petroleum ether = 1:10-1:5) to give compound **6**.

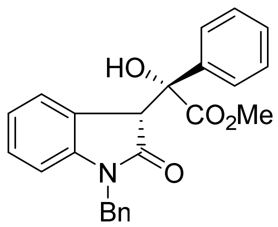

methyl 2-(1-benzyl-2-oxoindolin-3-yl)-2-hydroxy-2-phenylacetate (6a):

A white solid (60 mg, 77%), mp: 179-181 °C.

^{1}H NMR (400 MHz, $CDCl_3$) δ 7.77 (d, *J* = 7.4 Hz, 2H), 7.49-7.38 (m, 3H), 7.34-7.20 (m, 5H), 7.07 (t, *J* = 7.8 Hz, 1H), 6.66 (dd, *J* = 15.8, 7.8 Hz, 2H), 5.92 (d, *J* = 7.8 Hz, 1H), 5.03 (d, *J* = 15.8 Hz, 1H), 4.83 (d, *J* = 15.8 Hz, 1H), 4.62 (s, 1H), 4.13 (s, 1H), 4.00 (s, 3H). ^{13}C NMR (125 MHz, $CDCl_3$) δ 174.56, 174.24, 144.19, 138.91, 135.48, 128.77, 128.52, 128.47, 127.51, 127.08, 126.58, 124.82, 124.43, 122.14, 109.14, 78.73, 54.00, 53.69, 43.67. HRMS (ESI) calcd. for $C_{24}H_{22}NO_4$ $[M+H]^+$: 388.1543, found: 388.1549.

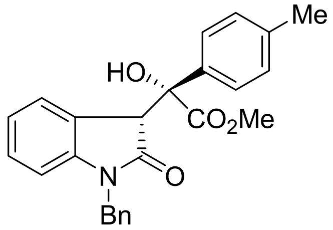

methyl 2-(1-benzyl-2-oxoindolin-3-yl)-2-hydroxy-2-(p-tolyl)acetate (6b):

A light yellow solid (64 mg, 80%), mp: 176-178 °C.

^{1}H NMR (300 MHz, $CDCl_3$) δ 7.63 (d, *J* = 8.3 Hz, 2H), 7.33-7.21 (m, 7H), 7.07 (t, *J* = 7.8 Hz, 1H), 6.73-6.60 (m, 2H), 6.00 (d, *J* = 7.8 Hz, 1H), 5.02 (d, *J* = 15.8 Hz, 1H), 4.83 (d, *J* = 15.8 Hz, 1H), 4.61 (s, 1H), 4.10 (s, 1H), 3.98 (s, 3H), 2.42 (s, 3H). ^{13}C NMR (125 MHz, $CDCl_3$) δ 174.64, 174.40, 144.17, 138.31, 135.91, 135.51, 129.24,

128.76, 128.42, 127.50, 127.08, 126.47, 124.90, 124.55, 122.13, 109.10, 78.68, 53.93, 53.60, 43.65, 21.17. HRMS (ESI) calcd. for $C_{25}H_{24}NO_4$ $[M+H]^+$: 402.1700, found: 402.1704.

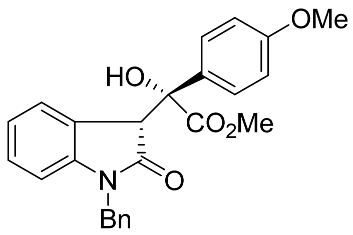

methyl 2-(1-benzyl-2-oxoindolin-3-yl)-2-hydroxy-2-(4-methoxyphenyl) acetate (6c):

A white solid (68 mg, 82%), mp: 98-99 °C.

^{1}H NMR (400 MHz, $CDCl_3$) δ 7.66 (d, *J* = 8.4 Hz, 2H), 7.35-7.20 (m, 5H), 7.08 (t, *J* = 7.7 Hz, 1H), 6.97 (d, *J* = 8.4 Hz, 2H), 6.70 (t, *J* = 7.7 Hz, 1H), 6.64 (d, *J* = 7.7 Hz, 1H), 6.02 (d, *J* = 7.7 Hz, 1H), 5.02 (d, *J* = 15.8 Hz, 1H), 4.83 (d, *J* = 15.8 Hz, 1H), 4.58 (s, 1H), 4.10 (s, 1H), 3.98 (s, 3H), 3.87 (s, 3H). ^{13}C NMR (100 MHz, $CDCl_3$) δ 174.61, 174.45, 159.70, 144.18, 135.50, 130.84, 128.78, 128.45, 127.85, 127.52, 127.09, 124.93, 124.56, 122.17, 113.83, 109.13, 78.46, 55.37, 53.95, 53.71, 43.65. HRMS (ESI) calcd. for $C_{25}H_{24}NO_5$ $[M+H]^+$: 418.1649, found: 418.1645.

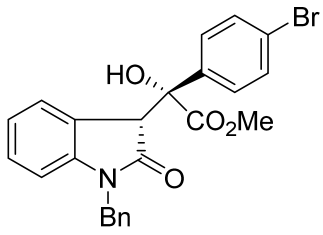

methyl (2-(1-benzyl-2-oxoindolin-3-yl)-2-(4-bromophenyl)-2-hydroxyacetate (6d):

A white solid (71 mg, 76%), mp: 147-149 °C.

^{1}H NMR (300 MHz, $CDCl_3$) δ 7.64 (d, *J* = 8.8 Hz, 2H), 7.57 (d, *J* = 8.8 Hz, 2H), 7.34-7.21 (m, 5H), 7.10 (t, *J* = 7.8 Hz, 1H), 6.73 (t, *J* = 7.8 Hz, 1H), 6.65 (d, *J* = 7.8 Hz, 1H), 6.02 (d, *J* = 7.8 Hz, 1H), 5.02 (d, *J* = 15.8 Hz, 1H), 4.82 (d, *J* = 15.8 Hz, 1H), 4.56 (s, 1H), 4.17 (s, 1H), 4.00 (s, 3H). ^{13}C NMR (125 MHz, $CDCl_3$) δ 174.28, 173.75, 144.18, 138.05, 135.36, 131.68, 128.79, 128.66, 128.43, 127.57, 127.06, 124.74, 124.11, 122.88, 122.28, 109.28, 78.50, 54.16, 53.50, 43.69. HRMS (ESI) calcd. for

$C_{24}H_{21}BrNO_4$ $[M+H]^+$: 466.0648, found: 466.0651.

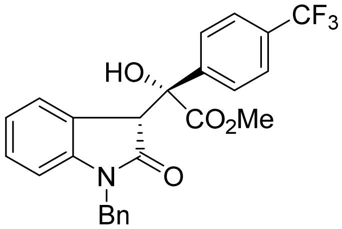

methyl 2-(1-benzyl-2-oxoindolin-3-yl)-2-hydroxy-2-(4-(trifluoromethyl)phenyl)acetate (6e):

A white solid (59 mg, 65%), mp: 156-158 °C.

^{1}H NMR (300 MHz, $CDCl_3$) δ 7.91 (d, J = 8.3 Hz, 2H), 7.72 (d, J = 8.3 Hz, 2H), 7.34-7.21 (m, 5H), 7.10 (t, J = 7.8 Hz, 1H), 6.69 (dd, J = 16.3, 7.8 Hz, 2H), 5.92 (d, J = 7.8 Hz, 1H), 5.03 (d, J = 15.8 Hz, 1H), 4.83 (d, J = 15.8 Hz, 1H), 4.61 (s, 1H), 4.25 (s, 1H), 4.01 (s, 3H). ^{13}C NMR (125 MHz, $CDCl_3$) δ 174.17, 173.53, 144.20, 142.96, 135.33, 130.95, 130.69, 128.81, 128.75, 127.61, 127.16, 127.07, 125.51, 125.48, 124.55, 123.94, 122.31, 109.36, 78.65, 54.29, 53.55, 43.73. HRMS (ESI) calcd. for $C_{25}H_{21}F_3NO_4$ $[M+H]^+$: 456.1417, found: 456.1421.

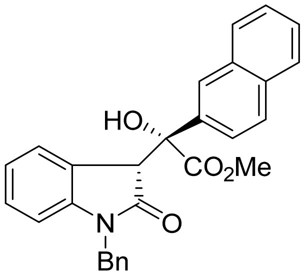

methyl 2-(1-benzyl-2-oxoindolin-3-yl)-2-hydroxy-2-(naphthalen-2-yl) acetate (6f):

A white solid (65 mg, 74%), mp: 162-164 °C.

^{1}H NMR (300 MHz, $CDCl_3$) δ 8.22 (s, 1H), 7.97-7.81 (m, 4H), 7.59-7.47 (m, 2H), 7.34-7.18 (m, 5H), 7.04 (t, J = 7.7 Hz, 1H), 6.69-6.51 (m, 2H), 5.92 (d, J = 7.7 Hz, 1H), 5.06 (d, J = 15.8 Hz, 1H), 4.83 (d, J = 15.8 Hz, 1H), 4.76 (s, 1H), 4.32 (s, 1H), 4.00 (s, 3H). ^{13}C NMR (125 MHz, $CDCl_3$) δ 174.64, 174.23, 144.18, 136.15, 135.48, 133.11, 133.05, 128.80, 128.71, 128.51, 128.32, 127.63, 127.54, 127.08, 126.75, 126.57, 126.19, 124.90, 124.38, 124.19, 122.25, 109.18, 79.02, 54.08, 53.38, 43.71.

HRMS (ESI) calcd. for $C_{28}H_{24}NO_4$ $[M+H]^+$: 438.1700, found: 438.1704.

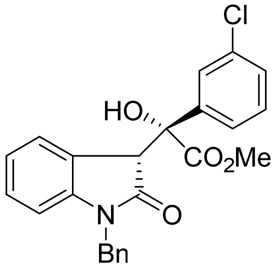

methyl 2-(1-benzyl-2-oxoindolin-3-yl)-2-(3-chlorophenyl)-2-hydroxyacetate (6g):

A light yellow solid (59 mg, 70%), mp: 110-112 °C.

^{1}H NMR (300 MHz, $CDCl_3$) δ 7.77 (d, *J* = 1.3 Hz, 1H), 7.70-7.63 (m, 1H), 7.45-7.36 (m, 2H), 7.33-7.21 (m, 5H), 7.10 (t, *J* = 7.8 Hz, 1H), 6.72 (t, *J* = 7.6 Hz, 1H), 6.65 (d, *J* = 7.8 Hz, 1H), 5.98 (d, *J* = 7.6 Hz, 1H), 5.03 (d, *J* = 15.9 Hz, 1H), 4.82 (d, *J* = 15.9 Hz, 1H), 4.57 (s, 1H), 4.17 (s, 1H), 4.02 (s, 3H). ^{13}C NMR (125 MHz, $CDCl_3$) δ 174.22, 173.69, 144.18, 141.06, 135.36, 134.77, 129.77, 128.80, 128.77, 128.67, 127.56, 127.04, 126.92, 124.88, 124.71, 124.04, 122.27, 109.29, 78.37, 54.24, 53.59, 43.69. HRMS (ESI) calcd. for $C_{24}H_{21}ClNO_4$ $[M+H]^+$: 422.1154, found: 422.1158.

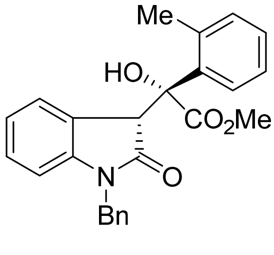

methyl 2-(1-benzyl-2-oxoindolin-3-yl)-2-hydroxy-2-(o-tolyl)acetate (6h):

A white solid (41 mg, 51%), mp: 129-130 °C.

^{1}H NMR (400 MHz, $CDCl_3$) δ 7.73 (d, *J* = 8.3 Hz, 1H), 7.38-7.18 (m, 8H), 7.11 (t, *J* = 7.8 Hz, 1H), 6.76 (t, *J* = 7.5 Hz, 1H), 6.67 (d, *J* = 7.8 Hz, 1H), 6.55 (d, *J* = 7.5 Hz, 1H), 5.00 (d, *J* = 15.8 Hz, 1H), 4.89 (s, 1H), 4.87 (d, *J* = 15.8 Hz, 1H), 4.18 (s, 1H), 3.92 (s, 3H), 2.58 (s, 3H). ^{13}C NMR (125 MHz, $CDCl_3$) δ 174.87, 174.18, 144.25, 137.54, 136.41, 135.54, 133.29, 128.78, 128.62, 128.51, 128.47, 127.53, 127.13, 126.06, 125.05, 124.89, 122.40, 109.14, 80.58, 53.83, 52.30, 43.70, 22.59. HRMS (ESI) calcd. for $C_{25}H_{24}NO_4$ $[M+H]^+$: 402.1700, found: 402.1697.

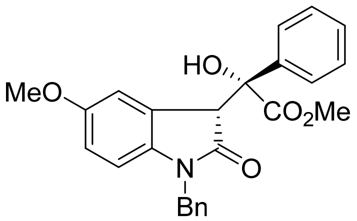

methyl 2-(1-benzyl-5-methoxy-2-oxoindolin-3-yl)-2-hydroxy-2-phenyl acetate (6i):

A white solid (67 mg, 81%), mp: 148-149 °C.

^{1}H NMR (300 MHz, $CDCl_3$) δ 7.78 (d, *J* = 6.6 Hz, 2H), 7.51-7.40 (m, 3H), 7.32-7.23 (m, 5H), 6.59 (dd, *J* = 8.5, 2.4 Hz, 1H), 6.51 (d, *J* = 8.5 Hz, 1H), 5.49 (d, *J* = 2.4 Hz, 1H), 5.01 (d, *J* = 15.8 Hz, 1H), 4.80 (d, *J* = 15.8 Hz, 1H), 4.59 (s, 1H), 4.15 (s, 1H), 4.00 (s, 3H), 3.39 (s, 3H). ^{13}C NMR (125 MHz, $CDCl_3$) δ 174.17, 155.20, 138.87, 137.60, 135.56, 128.77, 128.56, 127.50, 127.08, 126.67, 125.58, 113.38, 111.86, 109.45, 78.70, 55.27, 54.11, 54.03, 43.73. HRMS (ESI) calcd. for $C_{25}H_{24}NO_5$ $[M+H]^+$: 418.1649, found: 418.1652.

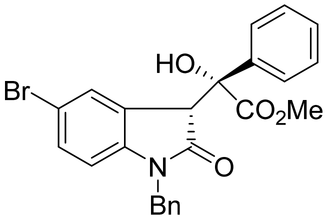

methyl 2-(1-benzyl-5-bromo-2-oxoindolin-3-yl)-2-hydroxy-2-phenylacetate (6j):

A white solid (69 mg, 74%), mp: 148-150 °C.

^{1}H NMR (400 MHz, $CDCl_3$) δ 7.75 (d, *J* = 7.4 Hz, 2H), 7.52-7.42 (m, 3H), 7.33-7.23 (m, 5H), 7.19 (d, *J* = 8.3 Hz, 1H), 6.49 (d, *J* = 8.3 Hz, 1H), 5.91 (s, 1H), 5.02 (d, *J* = 15.8 Hz, 1H), 4.79 (d, *J* = 15.8 Hz, 1H), 4.59 (s, 1H), 4.13 (s, 1H), 4.01 (s, 3H). ^{13}C NMR (125 MHz, $CDCl_3$) δ 173.93, 173.89, 143.20, 138.34, 134.98, 131.25, 128.86, 128.83, 128.68, 128.19, 127.69, 127.01, 126.44, 126.42, 114.93, 110.45, 78.64, 54.09, 53.79, 43.73. HRMS (ESI) calcd. for $C_{24}H_{21}BrNO_4$ $[M+H]^+$: 466.0648, found: 466.0651.

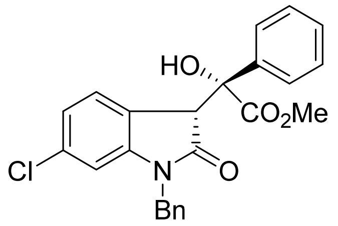

methyl 2-(1-benzyl-6-chloro-2-oxoindolin-3-yl)-2-hydroxy-2-phenylacetate (6k):

A light yellow solid (63 mg, 75%), mp: 181-183 °C.

^{1}H NMR (400 MHz, $CDCl_3$) δ 7.74 (d, *J* = 7.3 Hz, 2H), 7.49-7.39 (m, 3H), 7.36-7.23 (m, 5H), 6.63 (d, *J* = 7.9 Hz, 2H), 5.78 (d, *J* = 7.9 Hz, 1H), 4.99 (d, *J* = 15.9 Hz, 1H), 4.79 (d, *J* = 15.9 Hz, 1H), 4.57 (s, 1H), 4.12 (s, 1H), 3.99 (s, 3H). ^{13}C NMR (125 MHz, $CDCl_3$) δ 174.49, 173.97, 145.42, 138.60, 134.93, 134.37, 128.92, 128.68, 128.64, 127.75, 127.03, 126.49, 125.68, 122.80, 122.08, 109.72, 78.62, 54.08, 53.39, 43.77. HRMS (ESI) calcd. for $C_{24}H_{21}ClNO_4$ $[M+H]^+$: 422.1154, found: 422.1158.

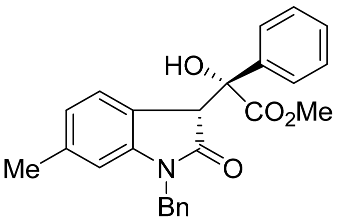

methyl 2-(1-benzyl-6-methyl-2-oxoindolin-3-yl)-2-hydroxy-2-phenylacetate (6l):

A light yellow solid (63 mg, 78%), mp: 196-198 °C.

^{1}H NMR (400 MHz, $CDCl_3$) δ 7.76 (d, *J* = 7.2 Hz, 2H), 7.53-7.37 (m, 3H), 7.35-7.18 (m, 5H), 6.48 (d, *J* = 7.5 Hz, 2H), 5.79 (d, *J* = 7.5 Hz, 1H), 5.00 (d, *J* = 15.9 Hz, 1H), 4.81 (d, *J* = 15.9 Hz, 1H), 4.58 (s, 1H), 4.11 (s, 1H), 3.98 (s, 3H), 2.17 (s, 3H). ^{13}C NMR (100 MHz, $CDCl_3$) δ 174.92, 174.32, 144.31, 139.01, 138.69, 135.64, 128.79, 128.52, 128.48, 127.47, 127.01, 126.61, 124.53, 122.83, 121.40, 110.00, 78.74, 54.00, 53.53, 43.60, 21.76. HRMS (ESI) calcd. for $C_{25}H_{24}NO_4$ $[M+H]^+$: 402.1700, found: 402.1697.

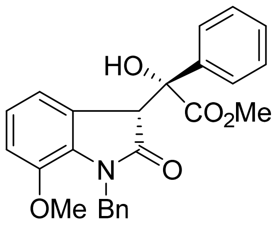

methyl 2-(1-benzyl-7-methoxy-2-oxoindolin-3-yl)-2-hydroxy-2-phenyl acetate (6m):

A white solid (44 mg, 53%), mp: 130-132 °C.

^{1}H NMR (400 MHz, $CDCl_3$) δ 7.76 (d, *J* = 7.3 Hz, 2H), 7.49-7.36 (m, 3H), 7.33-7.13 (m, 5H), 6.71 (d, *J* = 7.9 Hz, 1H), 6.62 (t, *J* = 7.9 Hz, 1H), 5.55 (d, *J* = 7.9 Hz, 1H), 5.27 (d, *J* = 15.2 Hz, 1H), 5.11 (d, *J* = 15.2 Hz, 1H), 4.58 (s, 1H), 4.13 (s, 1H), 3.97 (s, 3H), 3.57 (s, 3H). ^{13}C NMR (100 MHz, $CDCl_3$) δ 174.77, 174.31, 144.92, 138.97, 138.26, 132.42, 128.50, 128.46, 128.32, 126.92, 126.81, 126.65, 125.99, 122.72, 117.59, 112.90, 78.84, 55.73, 54.01, 53.83, 45.76. HRMS (ESI) calcd. for $C_{25}H_{24}NO_5$ $[M+H]^+$: 418.1649, found: 418.1652.

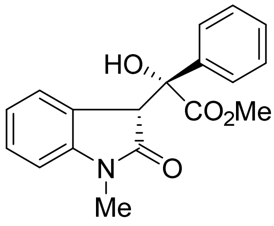

methyl 2-hydroxy-2-(1-methyl-2-oxoindolin-3-yl)-2-phenylacetate (6n):

A white solid (42 mg, 67%), mp: 156-157 °C.

^{1}H NMR (300 MHz, $CDCl_3$) δ 7.80-7.69 (m, 2H), 7.48-7.39 (m, 3H), 7.21 (t, *J* = 7.8 Hz, 1H), 6.80 (d, *J* = 7.8 Hz, 1H), 6.71 (td, *J* = 7.8, 0.9 Hz, 1H), 5.89 (d, *J* = 7.6 Hz, 1H), 4.52 (s, 1H), 4.03 (s, 1H), 3.98 (s, 3H), 3.21 (s, 3H). ^{13}C NMR (125 MHz, $CDCl_3$) δ 174.41, 174.25, 145.03, 138.91, 128.59, 128.52, 128.47, 126.55, 124.72, 124.40, 122.15, 108.09, 78.51, 53.95, 53.73, 26.18. HRMS (ESI) calcd. for $C_{18}H_{18}NO_4$ $[M+H]^+$: 312.1230, found: 312.1234.

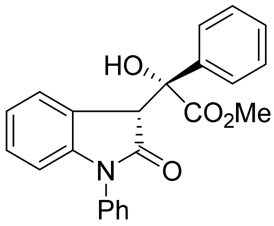

methyl 2-hydroxy-2-(2-oxo-1-phenylindolin-3-yl)-2-phenylacetate (6o):

A white solid (44 mg, 59%), mp: 175-177 °C.

^{1}H NMR (400 MHz, $CDCl_3$) δ 7.79 (d, *J* = 7.3 Hz, 2H), 7.55-7.36 (m, 8H), 7.13 (t, *J* = 7.7 Hz, 1H), 6.74 (t, *J* = 8.3 Hz, 2H), 5.97 (d, *J* = 7.7 Hz, 1H), 4.70 (s, 1H), 4.16 (s, 1H), 3.96 (s, 3H). ^{13}C NMR (125 MHz, $CDCl_3$) δ 174.09, 174.01, 145.19, 138.84, 134.25, 129.63, 128.56, 128.53, 128.47, 128.21, 126.77, 126.59, 125.02, 124.27, 122.53, 109.38, 78.84, 53.96, 53.94. HRMS (ESI) calcd. for $C_{23}H_{20}NO_4$ $[M+H]^+$: 374.1387, found: 374.1385.

Mechanistic studies on the dearomatization reaction of Scheme 4

1. Scheme 4a

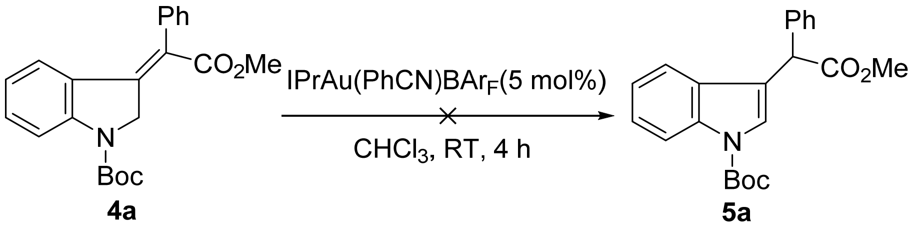

To a dry Schlenk tube was added IPrAu(PhCN)BAr$_F$ (16 mg, 0.01 mmol), **4a** (73 mg, 0.2 mmol) and $CHCl_3$ (4 mL) under an argon atmosphere. After stirring for 4 h at RT, the reaction mixture was concentrated and purified by flash chromatography on silica gel (petroleum ether/ethyl acetate = 60:1) to recover compound **4a**

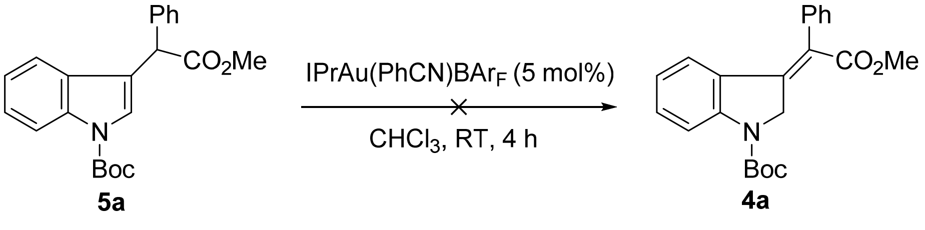

To a dry Schlenk tube was added IPrAu(PhCN)BAr$_F$ (16 mg, 0.01 mmol) and $CHCl_3$

(2 mL) under an argon atmosphere, then **5a** (73 mg, 0.2 mmol) in 2 mL dry $CHCl_3$ was added under an argon atmosphere. After stirring for 4 h at RT, the reaction mixture was concentrated and purified by flash chromatography on silica gel (petroleum ether/ethyl acetate = 60:1) to recover compound **5a**.

2. Scheme 4b

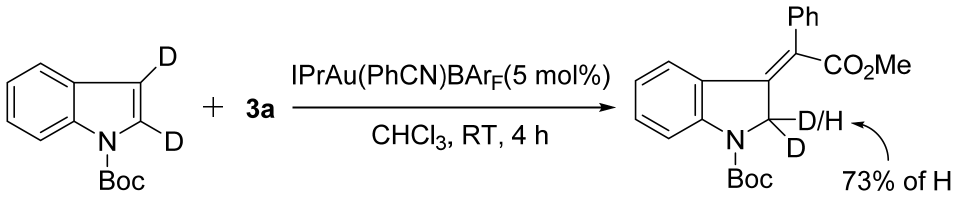

D-1a **D-4a**

To a dry Schlenk tube was added IPrAu(PhCN)BAr_F (16 mg, 0.01 mmol) and $CHCl_3$ (2 mL) under an argon atmosphere, then **D-1a** (44 mg, 0.2 mmol) and **3a** (53 mg, 0.3 mmol) in 2 mL dry $CHCl_3$ was added via a syringe pump over 2 h under an argon atmosphere. After stirring for 2 h at RT, the reaction mixture was concentrated and purified by flash chromatography on silica gel (petroleum ether/ethyl acetate = 80:1) to give product **D-4a** (65 mg, yield: 89%) as a white solid, mp: 190-192 °C.

^{1}H NMR (400 MHz, $CDCl_3$) δ 8.04 (s, 1H), 7.50-7.42 (m, 3H), 7.23 (d, J = 8.0 Hz, 3H), 6.59 (t, J = 8.0 Hz, 1H), 6.00 (d, J = 8.0 Hz, 1H), 5.12 (s, 0.73H), 3.71 (s, 3H), 1.61 (s, 9H). ^{13}C NMR (100 MHz, $CDCl_3$) δ 167.51, 151.43, 148.28, 147.77, 137.18, 132.07, 129.61, 129.26, 127.98, 127.39, 126.36, 121.90, 115.14, 99.99, 81.53, 55.56, 52.00, 28.48. HRMS (ESI) calcd. for $C_{22}H_{22}D_2NO_4$ $[M+H]^+$: 368.1825, found: 368.1823.

3. Scheme 4c

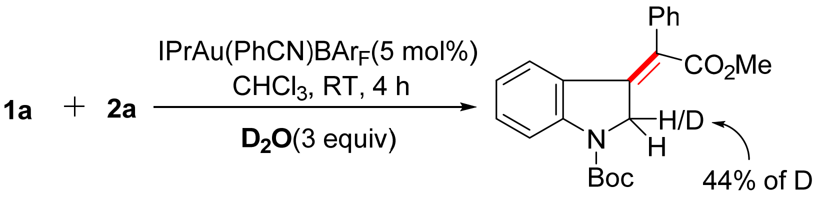

D-4a

To a dry Schlenk tube was added IPrAu(PhCN)BAr_F (16 mg, 0.01 mmol), D_2O (12

mg, 0.6 mmol) and $CHCl_3$ (2 mL) under an argon atmosphere, then **1a** (43 mg, 0.2 mmol) and **2a** (53 mg, 0.3 mmol) in 2 mL dry $CHCl_3$ was added via a syringe pump over 2 h under an argon atmosphere. After stirring for 2 h at RT, the reaction mixture was concentrated and purified by flash chromatography on silica gel (petroleum ether/ethyl acetate = 80:1) to give product **D-4a** as a white solid (61 mg, yield: 84%).

^{1}H NMR (300 MHz, $CDCl_3$) δ 8.04 (s, 1H), 7.55-7.41 (m, 3H), 7.26-7.18 (m, 3H), 6.66-6.53 (m, 1H), 5.99 (d, J = 9.0 Hz, 1H), 5.17-5.11 (m, 1.56H), 3.71 (s, 3H), 1.61 (s, 9H). ^{13}C NMR (100 MHz, $CDCl_3$) δ 167.51, 151.42, 137.18, 132.08, 129.61, 129.27, 127.98, 127.32, 126.37, 121.90, 115.14, 81.57, 55.61, 52.00, 28.48. HRMS (ESI) calcd. for $C_{22}H_{23}DNO_4$ $[M+H]^+$: 367.1763, found: 367.1764.

4. Scheme 4d

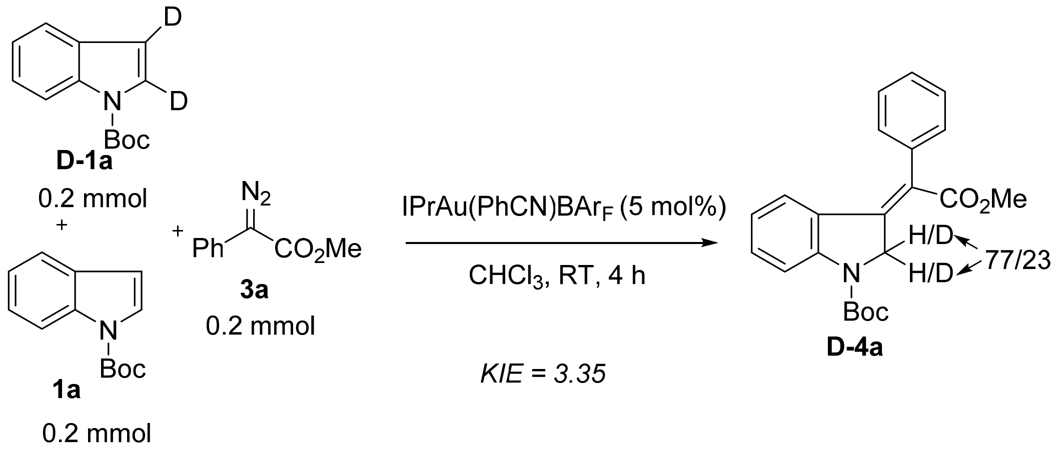

To a dry Schlenk tube was added IPrAu(PhCN)BAr$_F$ (16 mg, 0.01 mmol) and $CHCl_3$ (2 mL) under an argon atmosphere, then **1a** (43.4 mg, 0.2 mmol), **D-1a** (43.8 mg, 0.2 mmol) and **3a** (35.2 mg, 0.3 mmol) in 2 mL dry $CHCl_3$ was added via a syringe pump over 2 h under an argon atmosphere. After stirring for 2 h at RT, the reaction mixture was concentrated and purified by flash chromatography on silica gel (petroleum ether/ethyl acetate = 80:1) to give product **D-4a** (59 mg, yield: 80%) as a white solid. The ^{1}H NMR analysis showed that the KIE value was 3.35.

^{1}H NMR (300 MHz, $CDCl_3$) δ 8.03 (s, 1H), 7.52-7.40 (m, 3H), 7.29-7.16 (m, 3H), 6.68-6.53 (m, 1H), 6.00 (d, J = 7.9 Hz, 1H), 5.17-5.08 (m, 1.54H), 3.71 (s, 3H), 1.61 (s, 9H). ^{13}C NMR (100 MHz, $CDCl_3$) δ 167.52, 151.42, 137.19, 132.08, 129.62,

129.26, 128.92, 127.98, 127.20, 126.36, 121.90, 121.57, 115.15, 81.53, 77.39, 77.07, 76.75, 55.61, 51.99, 28.48.

5. Scheme 4e

Preparation of compound **7**: (**7** was prepared according to the similar literature procedure.)[6]

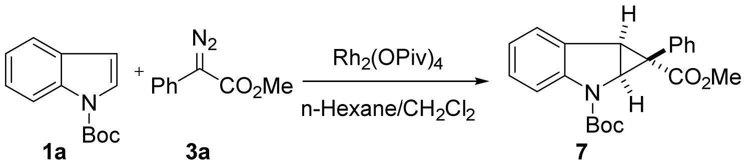

1a **3a** **7**

To a tube was added **1a** (217 mg, 1 mmol), $Rh_2(OPiv)_4$ (3.05 mg, 0.005 mmol) and n-Hexane (5 mL) under argon atmosphere, then **3a** (176 mg, 1 mmol) in n-Hexane/CH_2Cl_2 (3.5 mL/1.5 mL) was added via a syringe pump over 1.5 h. After stirred at rt for 1 h, the reaction mixture was concentrated and purified by flash chromatography on silica gel (CH_2Cl_2/petroleum ether = 1:4-1:1) to give **7** (102 mg, yield: 28%) as a white solid. ^{1}H NMR (400 MHz, DMSO-d_6) δ 7.56-7.48 (m, 1H), 7.34-6.87 (m, 8H), 4.79 (d, J = 6.8 Hz, 1H), 3.78 (d, J = 6.8 Hz, 1H), 3.59 (s, 3H), 1.56 (d, J = 25.5 Hz, 9H). ^{13}C NMR (75 MHz, DMSO-d_6) δ 173.02, 151.41, 132.32, 130.84, 128.91, 128.04, 127.63, 126.61, 126.31, 122.79, 114.26, 114.13, 81.90, 53.02, 50.38, 35.25, 31.64, 28.33.

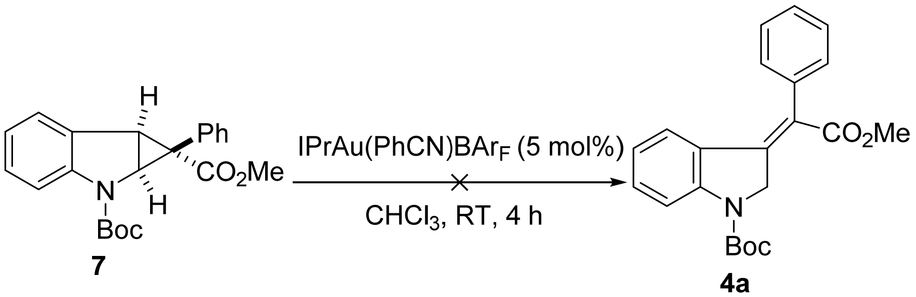

7 **4a**

To a dry Schlenk tube was added IPrAu(PhCN)BAr$_F$ (16 mg, 0.01 mmol) and $CHCl_3$ (2 mL), then **7** (73 mg, 0.2 mmol) in $CHCl_3$ (2 mL) under an argon atmosphere. The reaction solution was stirred for 4 h at RT. The reaction mixture was concentrated and purified by flash chromatography on silica gel (petroleum ether/ethyl acetate = 15:1)

to recover **7**.

Mechanistic studies on the aerobic oxidation of Scheme 5

1. Scheme 5a

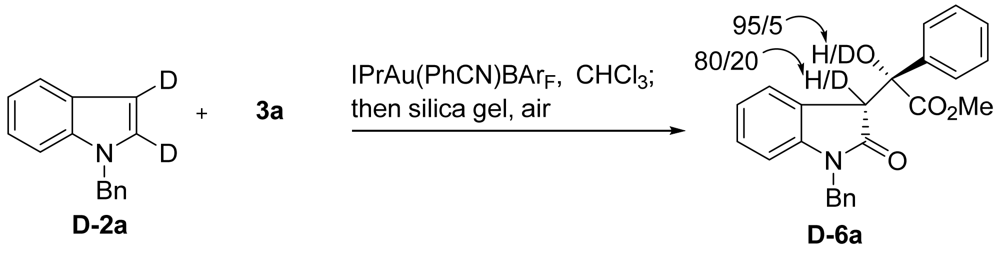

To a dry Schlenk tube was added IPrAu(PhCN)BAr_F (16 mg, 0.01 mmol) and $CHCl_3$ (2 mL) under an argon atmosphere, then **D-2a** (42 mg, 0.2 mmol) and **3a** (53 mg, 0.3 mmol) in 2 mL dry $CHCl_3$ was added via a syringe pump over 2 h under an argon atmosphere, then the resulting solution was stirred at RT for 2 h. The mixture was adsorbed on silica gel (5 g) by removing the solvent under vacuum; the silica gel powder was spread on a paper and exposed under air for 12 h. The yellow color of silica gel was disappeared. The crude product was eluted with EtOAc from the silica gel, concentrated and purified by column chromatography (silica gel, EtOAc/Petroleum ether = 1:10-1:5) to give compound **D-6a** (57 mg, 73%) as a white solid, mp: 179-180 °C.

^{1}H NMR (400 MHz, $CDCl_3$) δ 7.77 (d, *J* = 7.4 Hz, 2H), 7.49-7.40 (m, 3H), 7.34-7.20 (m, 5H), 7.07 (t, *J* = 7.7 Hz, 1H), 6.66 (dd, *J* = 15.8, 7.8 Hz, 2H), 5.92 (d, *J* = 7.4 Hz, 1H), 5.02 (d, *J* = 15.8 Hz, 1H), 4.83 (d, *J* = 15.8 Hz, 1H), 4.62 (s, 0.8H), 4.14 (s, 0.95H), 4.00 (s, 3H). ^{13}C NMR (100 MHz, $CDCl_3$) δ 174.58, 174.27, 144.19, 138.91, 135.49, 128.80, 128.55, 128.50, 127.53, 127.09, 126.60, 124.83, 124.44, 122.17, 109.17, 78.74, 54.04, 53.71, 43.68. HRMS (ESI) calcd. for $C_{24}H_{20}D_2NO_4$ $[M+H]^+$: 390.1669, found: 390.1666.

2. Scheme 5b

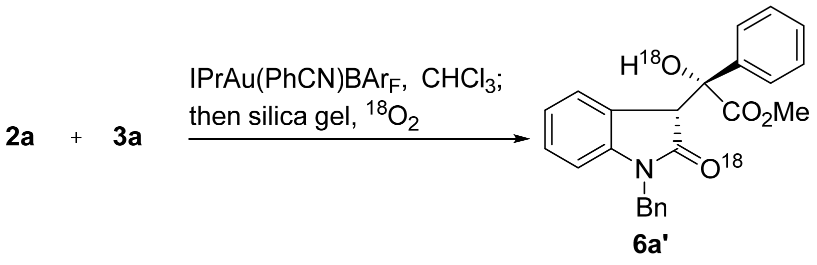

To a dry Schlenk tube was added IPrAu(PhCN)BAr_F (16 mg, 0.01 mmol) and $CHCl_3$ (2 mL) under an argon atmosphere, then **2a** (41.4 mg, 0.2 mmol) and **3a** (53 mg, 0.3 mmol) in 2 mL dry $CHCl_3$ was added via a syringe pump over 2 h under an argon atmosphere, then the resulting solution was stirred at RT for 2 h. To the reaction solution was added silica gel (5 g) and removed the solvent under vacuum, then the silica gel powder was stirred in a flask under a $^{18}O_2$ balloon for 12 h and the color of silica gel was disappeared. The crude product was eluted with EtOAc from the silica gel, concentrated and purified by column chromatography (silica gel, EtOAc/Petroleum ether = 1:10-1:5) to give compound **6a’** as a white solid (61 mg, 78%), mp: 179-180 °C.

^{1}H NMR (400 MHz, $CDCl_3$) δ 7.77 (d, *J* = 7.2 Hz, 2H), 7.49-7.38 (m, 3H), 7.34-7.20 (m, 5H), 7.07 (t, *J* = 7.8 Hz, 1H), 6.66 (dd, *J* = 15.7, 7.8 Hz, 2H), 5.92 (d, *J* = 7.8 Hz, 1H), 5.02 (d, *J* = 15.8 Hz, 1H), 4.83 (d, *J* = 15.8 Hz, 1H), 4.62 (s, 1H), 4.13 (s, 1H), 3.99 (s, 3H). ^{13}C NMR (100 MHz, $CDCl_3$) δ 174.57, 174.26, 144.18, 138.90, 135.48, 128.79, 128.54, 128.49, 127.53, 127.08, 126.59, 124.82, 124.43, 122.16, 109.16, 78.71, 54.03, 53.70, 43.68. HRMS (ESI) calcd. for $C_{24}H_{22}NO_2{}^{18}O_2$ $[M+H]^+$: 392.1628, found: 392.1634.

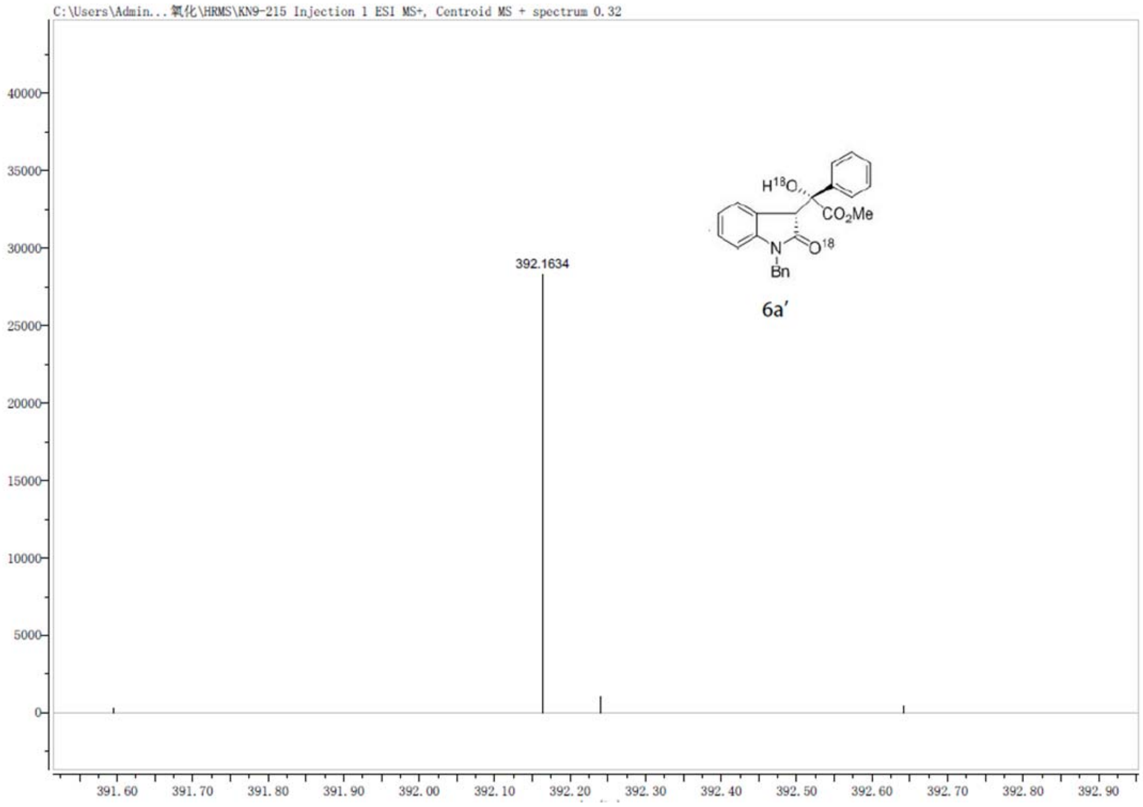

3. Scheme 5c

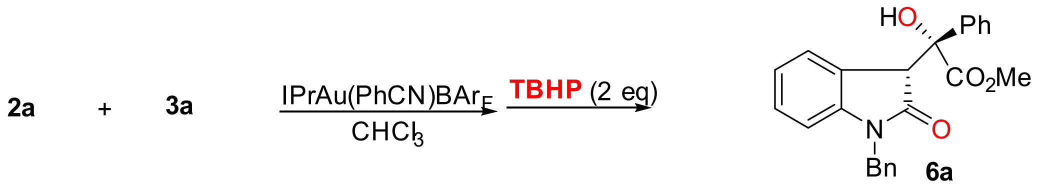

(messy, **6a** can be detected by GCMS)

To a dry Schlenk tube was added IPrAu(PhCN)BAr$_F$ (16 mg, 0.01 mmol) and $CHCl_3$ (2 mL) under an argon atmosphere, then **2a** (41 mg, 0.2 mmol) and **3a** (53 mg, 0.3 mmol) in 2 mL dry $CHCl_3$ was added via a syringe pump over 2 h under an argon atmosphere, then the resulting solution was stirred at RT for 2 h. To the reaction solution was added 70% TBHP (51 mg, 0.4 mmol), the solution was stirred at RT for 12 h. TLC analysis showed the reaction was messy, but **6a** can be detected by GCMS.

4. Scheme 5d

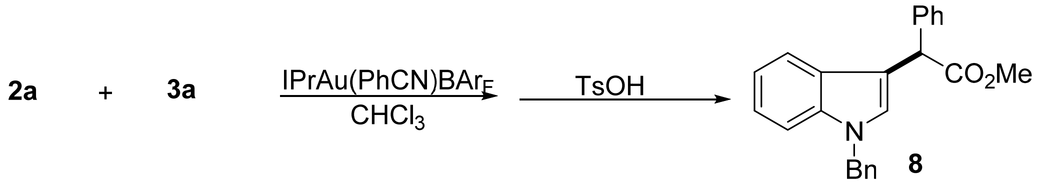

To a dry Schlenk tube was added IPrAu(PhCN)BAr$_F$ (16 mg, 0.01 mmol) and $CHCl_3$ (2 mL) under an argon atmosphere, then **2a** (41 mg, 0.2 mmol) and **3a** (53 mg, 0.3 mmol) in 2 mL dry $CHCl_3$ was added via a syringe pump over 2 h under an argon atmosphere, then the resulting solution was stirred at RT for 2 h. To the reaction solution was added TsOH (0.1 eq), then removed the solvent under vacuum, the crude residue was exposed under air for 1 h. The crude residue was purified by column chromatography (silica gel, EtOAc/Petroleum ether = 1:30-1:15) to give **8**[7] (50 mg, 70%) as a white solid.

^{1}H NMR (300 MHz, $CDCl_3$) δ 7.47-7.39 (m, 3H), 7.34-7.25 (m, 5H), 7.24-7.01 (m, 7H), 5.29 (s, 2H), 5.28 (s, 1H), 3.74 (s, 3H). ^{13}C NMR (75 MHz, $CDCl_3$) δ 173.47, 138.66, 137.48, 136.70, 128.79, 128.59, 128.45, 127.61, 127.45, 127.35, 127.29, 126.70, 122.09, 119.53, 119.24, 112.66, 109.93, 52.35, 50.18, 48.89.

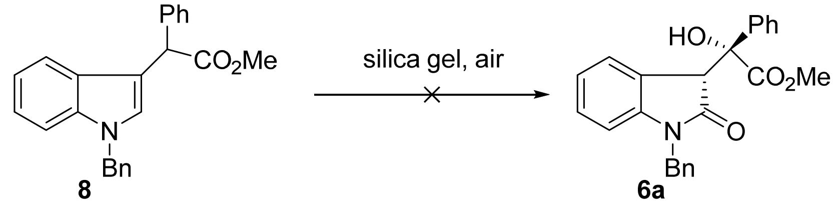

To a solution of **8** (71 mg, 0.2 mmol) in $CHCl_3$ (4 mL) was added silica gel (5 g), then the solvent was removed under vacuum to obtain the silica gel powder which was spread on a paper and exposed under air for 12 h. **8** was not converted to **6a**.

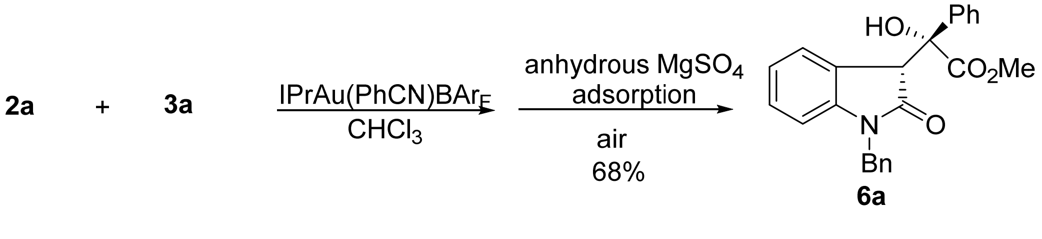

To a dry Schlenk tube was added IPrAu(PhCN)BAr$_F$ (16 mg, 0.01 mmol) and $CHCl_3$ (2 mL) under an argon atmosphere, then **2a** (41 mg, 0.2 mmol) and **3a** (53 mg, 0.3 mmol) in 2 mL dry $CHCl_3$ was added via a syringe pump over 2 h under an argon atmosphere, then the resulting solution was stirred at RT for 2 h. The mixture was adsorbed on anhydrous $MgSO_4$ (5 g) by removing the solvent under vacuum; the powder was spread on a paper and exposed under air for 12 h. The crude product was eluted with EtOAc from $MgSO_4$, concentrated and purified by column chromatography (silica gel, EtOAc/Petroleum ether = 1:10-1:5) to give compound **6a** (53 mg, 68%).

5. Scheme 5e

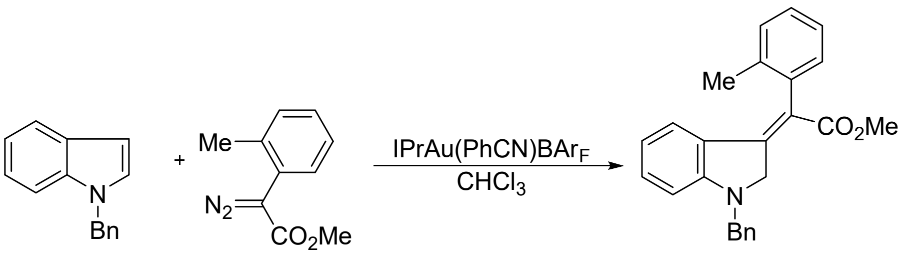

To a dry Schlenk tube was added IPrAu(PhCN)BAr$_F$ (16 mg, 0.01 mmol) and $CHCl_3$ (2 mL) under an argon atmosphere, then **2a** (41.4 mg, 0.2 mmol) and methyl 2-diazo-2-(o-tolyl)acetate (57 mg, 0.3 mmol) in 2 mL dry $CHCl_3$ was added via a syringe pump over 2 h under an argon atmosphere, then the resulting solution was stirred at RT for 2 h. The reaction solution was concentrated; the crude residue was purified by column chromatography (silica gel, EtOAc/Petroleum ether = 1:30-1:15) to give **9** as yellow oil (48 mg, 65%). [Note: (1) The silica gel was pretreated with TEA. (2) The product was unstable.]

^{1}H NMR (400 MHz, Acetone-d_6) δ 7.38-7.23 (m, 8H), 7.15-7.08 (m, 2H), 6.78 (d, J = 8.0 Hz, 1H), 6.23 (t, J = 8.0 Hz, 1H), 5.84 (d, J = 8.0 Hz, 1H), 4.75 (s, 2H), 4.69-4.58 (m, 2H), 3.56 (s, 3H), 2.09 (s, 3H). ^{13}C NMR (100 MHz, Acetone) δ 166.75, 156.32, 151.80, 138.17, 137.47, 136.93, 132.57, 130.23, 129.85, 128.65, 127.80, 127.61, 127.30, 126.62, 126.13, 124.56, 118.21, 116.56, 107.57, 59.07, 50.75, 50.19, 18.72. HRMS (ESI) calcd. for $C_{25}H_{24}NO_2$ $[M+H]^+$: 370.1802, found: 370.1805.

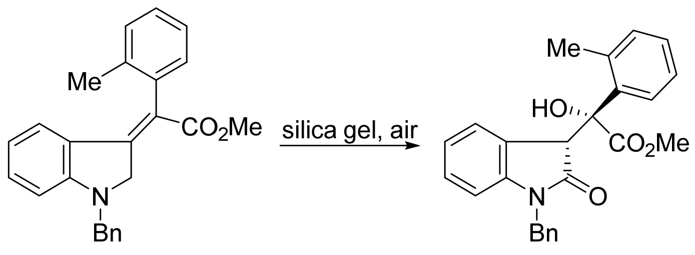

To a solution of **9** (37 mg, 0.1 mmol) in $CHCl_3$ (4 mL) was added silica gel (5 g), then the solvent was removed under vacuum to obtain the silica gel powder which was spread on a paper and exposed under air for 24 h. The yellow color of silica gel was almost disappeared. The crude product was eluted with EtOAc from the silica gel, concentrated and purified by column chromatography (silica gel, EtOAc/Petroleum ether = 1:10-1:5) to give **6h** (28 mg, 73%).

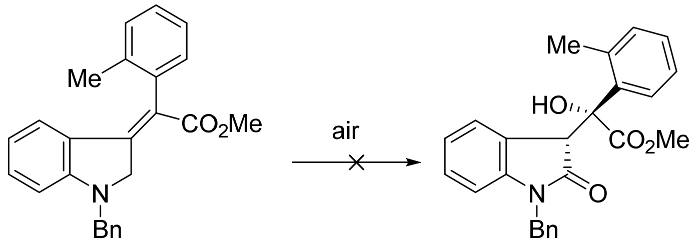

9 was exposed under air for 24 h. TLC analysis showed **6h** was not generated.

X-ray structure of 4a, 4x, 6a and 6l

The crystal structures have been deposited at the Cambridge Crystallographic Data Centre (CCDC 1551068, **4a**), (CCDC 1551070, **4x**), (CCDC 1572912, **6a**) and (CCDC 1572913, **6l**). The data can be obtained free of charge via the internet at www.ccdc.cam.ac.uk/data_request/cif.

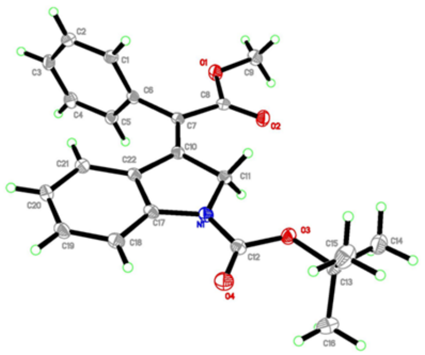

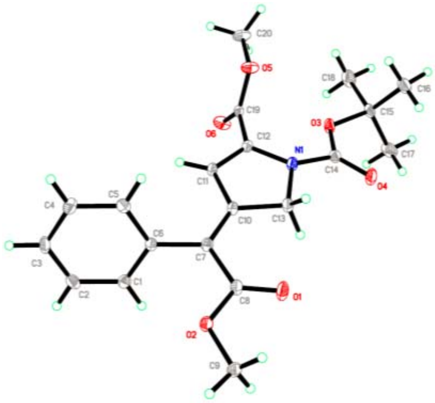

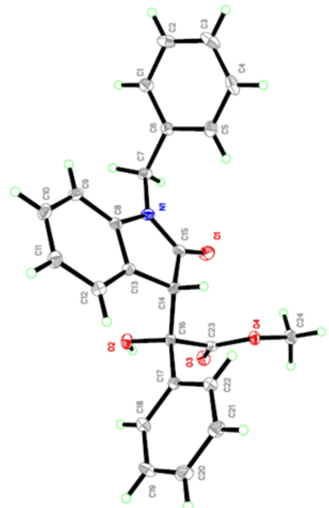

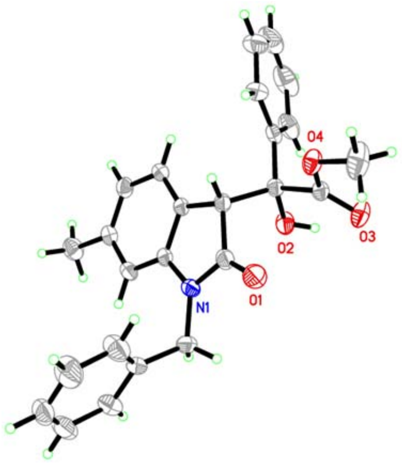

References

[1] (a) Davies, H. M. L.; Hansen, T.; Churchill, M. R. *J. Am. Chem. Soc.* **2000**, *122*, 3063. (b) Sambasivan, R.; Ball, Z. T. *J. Am. Chem. Soc.* **2010**, *132*, 9289. (c) Qu, Z.; Shi, W.; Wang, J. *J. Org. Chem.* **2001**, *66*, 8139.

[2] Huguet, N.; Leboeuf, D.; Echavarren, A. M. *Chem. Eur. J.* **2013**, *19*, 6581.

[3] (a) Xu, S.; Huang, X.; Hong, X.; Xu, B.; *Org. Lett.* **2012**, *14*, 4614. (b) Willis, M. C.; Brace, G. N.; Findlay, T. J. K.; Holmes, I. P. *Adv. Synth. Catal.* **2006**, *348*, 851. (c) Lang, R.; Wu, J.; Shi, L.; Xia, C.; Li, F. *Chem. Commun.* **2011**, *47*, 12553. (d) Xu, X.-H.; Liu, G.-K.; Azuma, A.; Tokunaga, E.; Shibata, N. *Org. Lett.* **2011**, *13*, 4854. (e) Salvi, L.; Davis, N. R.; Ali, S. Z.; Buchwald, S. L. *Org. Lett.* **2012**, *14*, 170. (f) Choy, P. Y.; Lau, C. P.; Kwong, F. Y. *J. Org. Chem.* **2011**, *76*, 80. (g) Malmgren, J.; Nagendiran, A.; Tai, C.-W.; Bäckvall, J.-E.; Olofsson B. *Chem. Eur. J.* **2014**, *20*, 13531. (h) Pan, S.; Ryu, N.; Shibata T. *J. Am. Chem. Soc.* **2012**, *134*, 17474. (i) Williams, C. W.; Shenje, R.; France, S. *J. Org. Chem.* **2016**, *81*, 8253. (j) Ackermann, L.; Lygin, A. V. *Org. Lett.* **2011**, *13*, 3332. (k) Witulski, B.; Buschmann, N.; Bergsträßer, U. *Tetrahedron* **2000**, *56*, 8473. (l) Kirchberg, S.; Fröhlich, R.; Studer, A. *Angew. Chem. Int. Ed.* **2009**, *48*, 4235.

[4] Ibaceta-Lizana, J. S. L.; Jackson, A. H.; Prasitpan, N.; Shannon, P. V. R. *J. Chem. Soc. Perkin Trans. 2* **1987**, 1221.

[5] Chan, W.-W.; Yeung, S.-H.; Zhou, Z.; Chan, A. S. C.; Yu, W.-Y. *Org. Lett.*

2010, *12*, 604.

[6] Lehner, V.; Davies, H. M. L.; Reiser, O. *Org. Lett.* **2017**, *19*, 4722.

[7] Cai, Y.; Zhu, S.-F.; Wang, G.-P.; Zhou, Q.-L. *Adv. Synth. Catal.* **2011**, *353*, 2939.

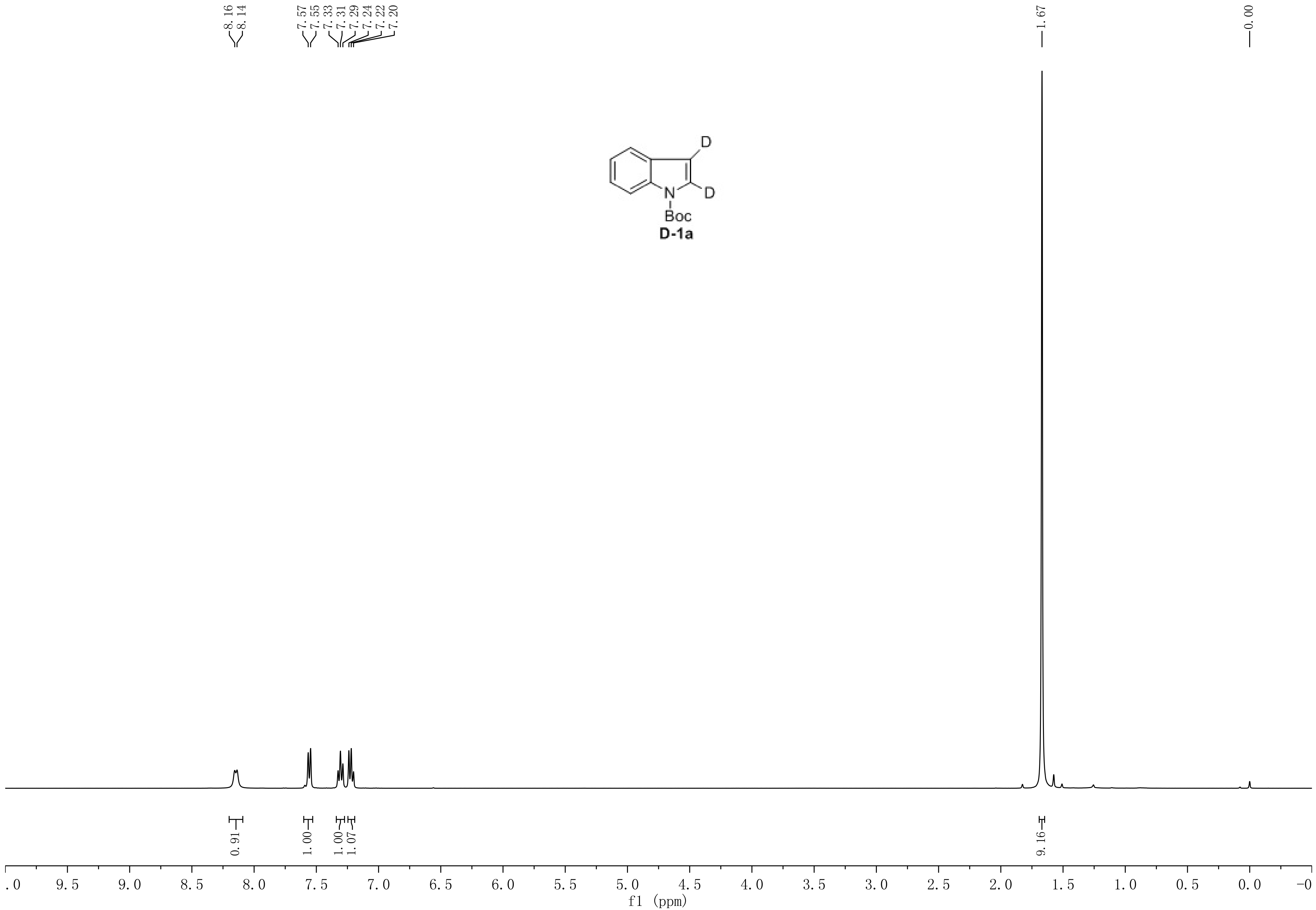

D-1a

8.16
8.14
7.57
7.55
7.33
7.31
7.29
7.24
7.22
7.20
1.67
0.00
0.91
1.00
1.00
1.07
9.16
f1 (ppm)

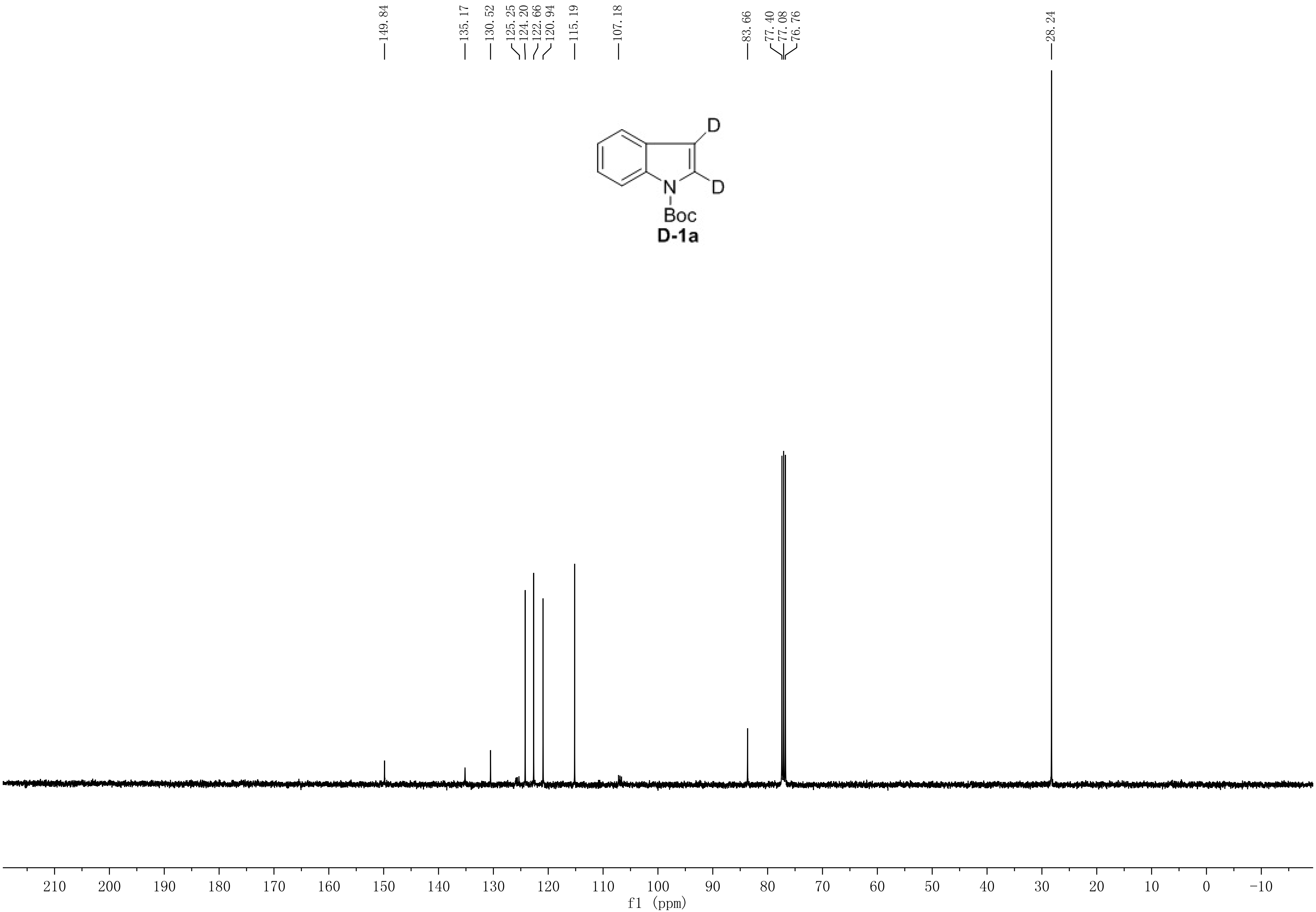

149.84
135.17
130.52
125.25
124.20
122.66
120.94
115.19
107.18
83.66
77.40
77.08
76.76
28.24
D
D
N
Boc
D-1a
210
200
190
180
170
160
150
140
130
120
110
100
90
80
70
60
50
40
30
20
10
0
−10
f1 (ppm)

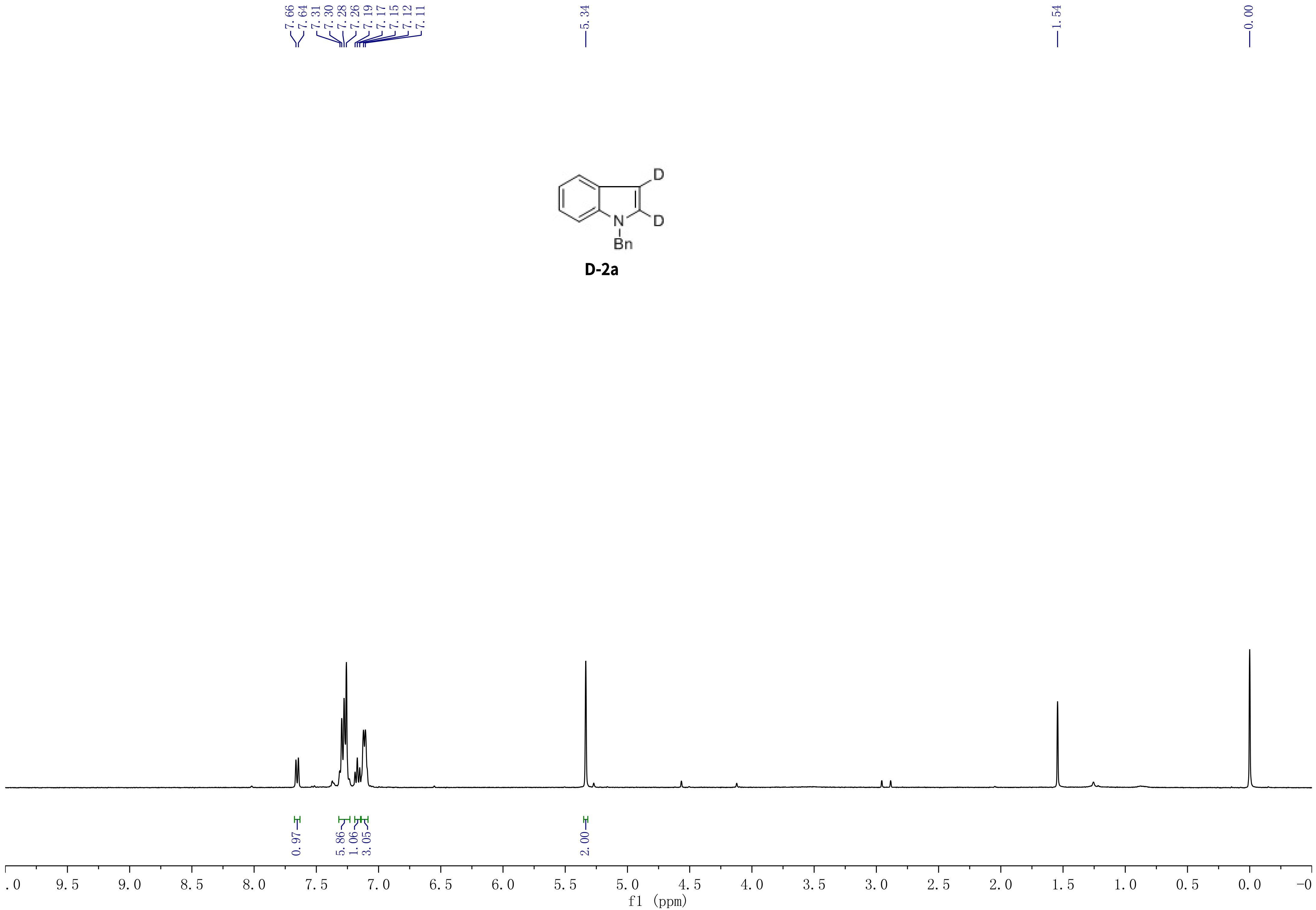
7.66
7.64
7.31
7.30
7.28
7.26
7.19
7.17
7.15
7.12
7.11
5.34
1.54
0.00
D
D
N
Bn
D-2a
0.97
5.86
1.06
3.05
2.00
f1 (ppm)

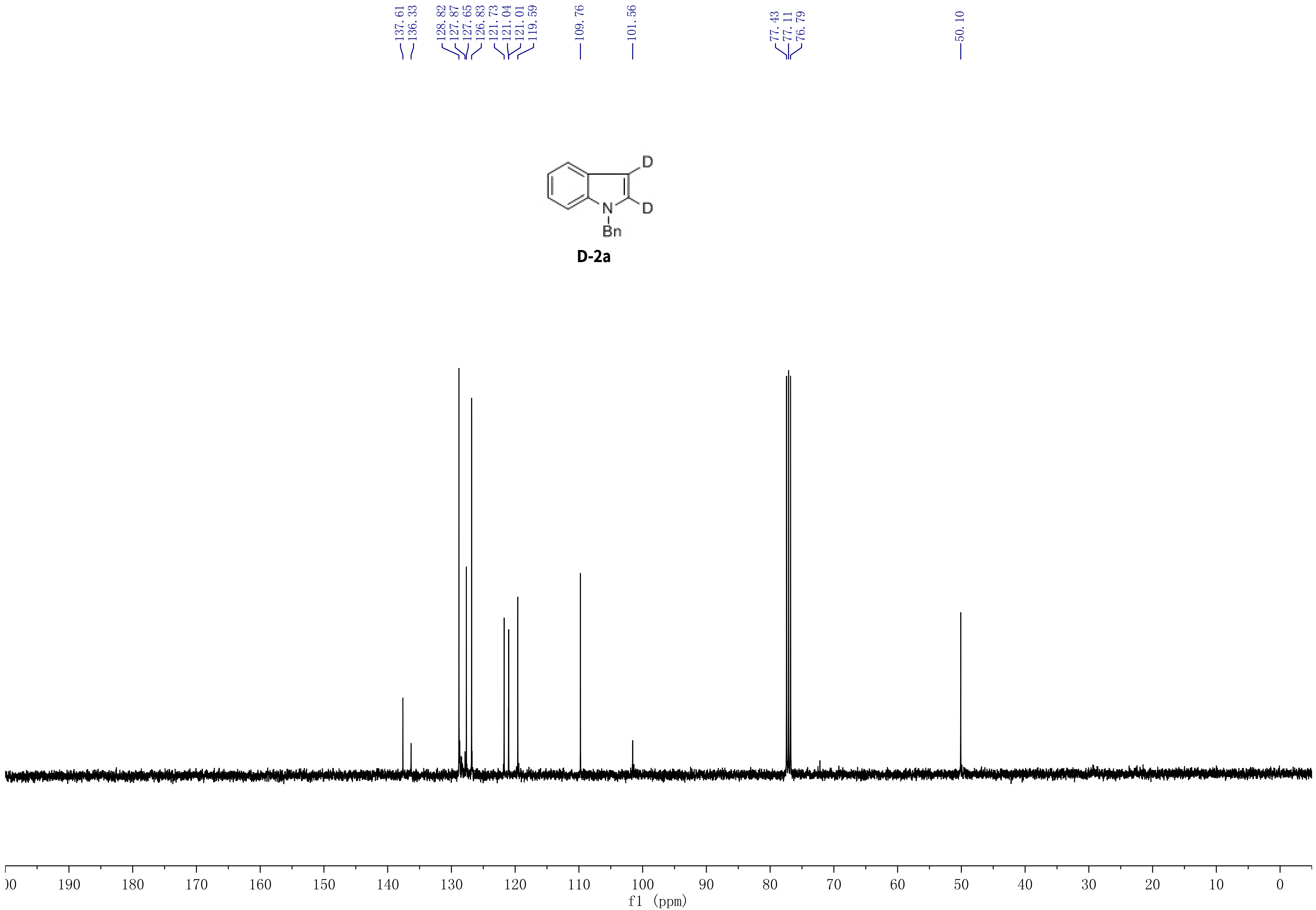
137.61
136.33
128.82
127.87
127.65
126.83
121.73
121.04
121.01
119.59
109.76
101.56
77.43
77.11
76.79
50.10
D
D
N
Bn
D-2a
190
180
170
160
150
140
130
120
110
100
90
80
70
60
50
40
30
20
10
0
f1 (ppm)

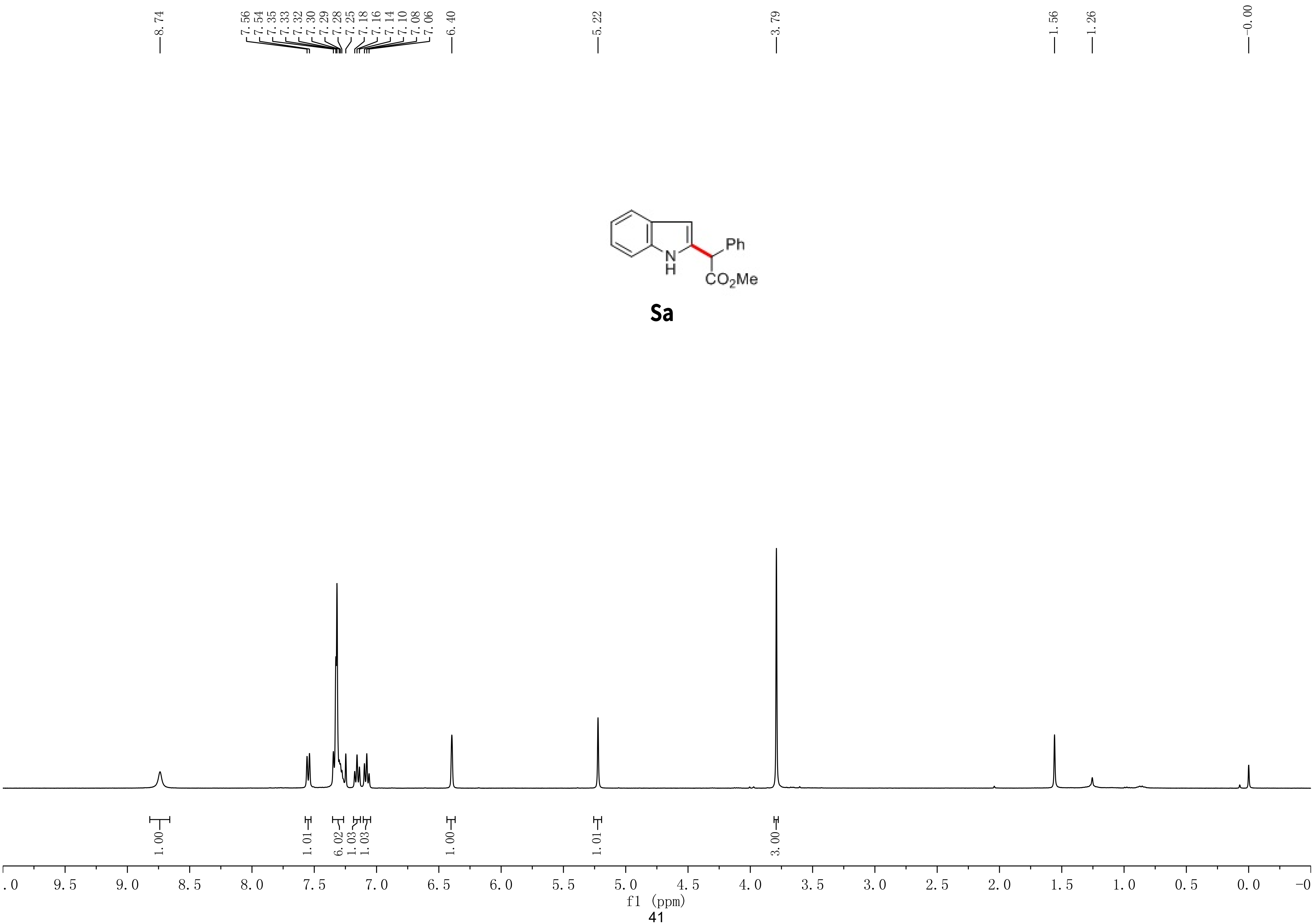
8.74
7.56 7.54 7.35 7.33 7.32 7.30 7.29 7.28 7.25 7.18 7.16 7.14 7.10 7.08 7.06
6.40
5.22
3.79
1.56
1.26
-0.00
Ph
N
H
CO2Me
1.00
1.01
6.02
1.03
1.03
1.00
1.01
3.00
f1 (ppm)

Sa

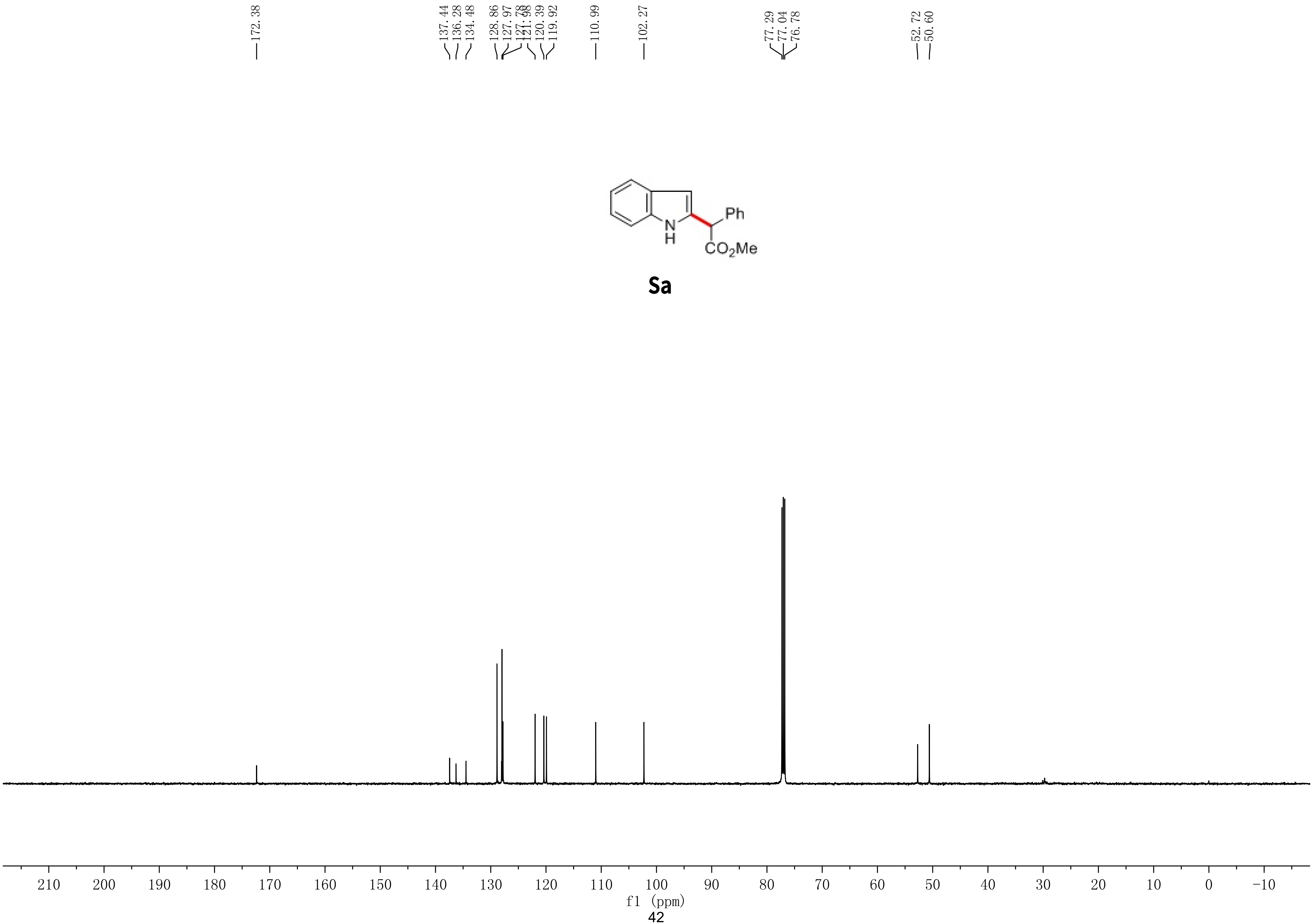
172.38
137.44
136.28
134.48
128.86
127.97
127.78
121.98
120.39
119.92
110.99
102.27
77.29
77.04
76.78
52.72
50.60
Ph
N
H
CO2Me
Sa
210 200 190 180 170 160 150 140 130 120 110 100 90 80 70 60 50 40 30 20 10 0 −10
f1 (ppm)

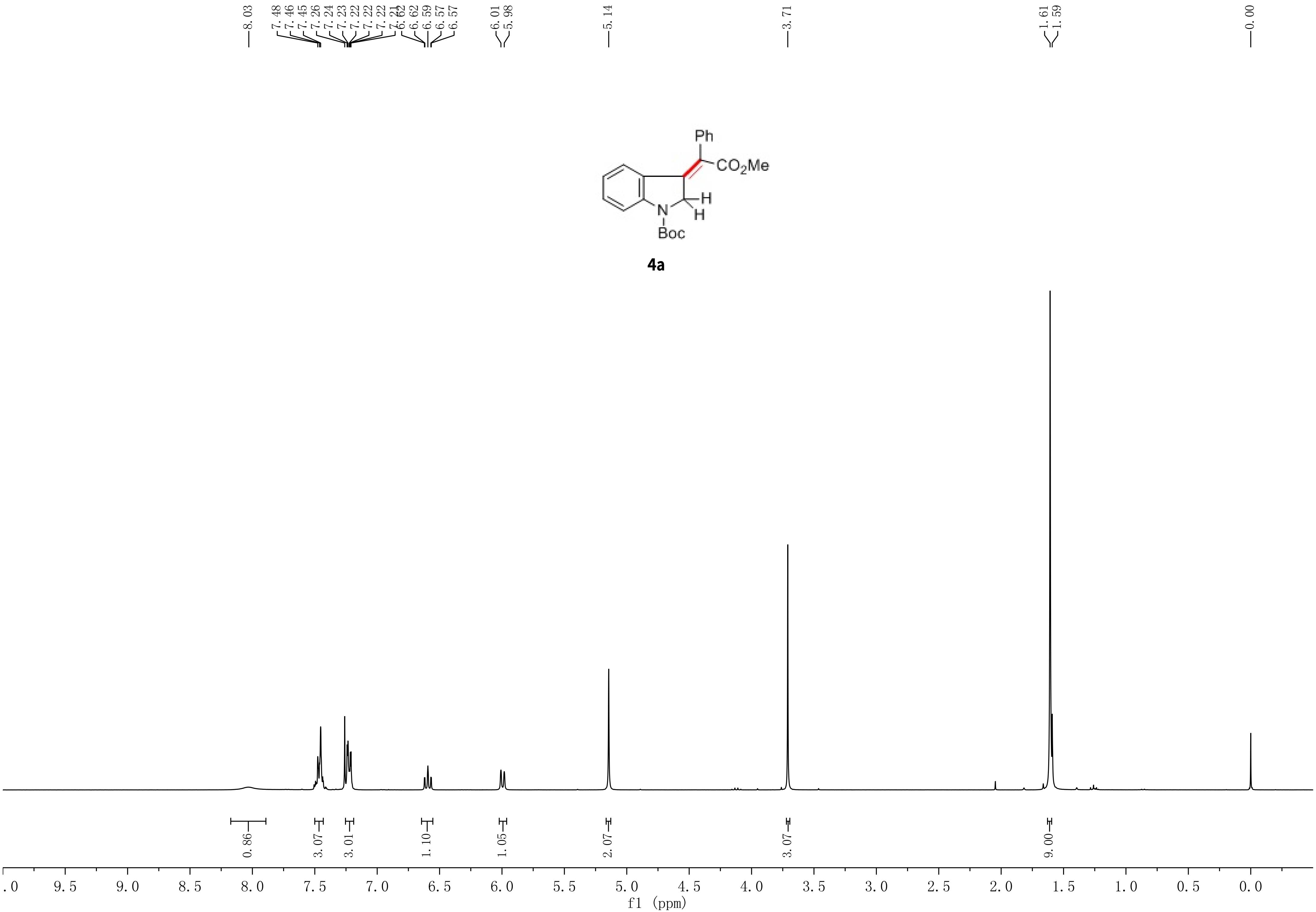
8.03
7.48
7.46
7.45
7.26
7.24
7.23
7.22
7.22
7.22
7.21
6.62
6.62
6.59
6.57
6.57
6.01
5.98
5.14
3.71
1.61
1.59
0.00
Ph
CO2Me
H
H
N
Boc
4a
0.86
3.07
3.01
1.10
1.05
2.07
3.07
9.00
.0
9.5
9.0
8.5
8.0
7.5
7.0
6.5
6.0
5.5
5.0
4.5
4.0
3.5
3.0
2.5
2.0
1.5
1.0
0.5
0.0
f1 (ppm)

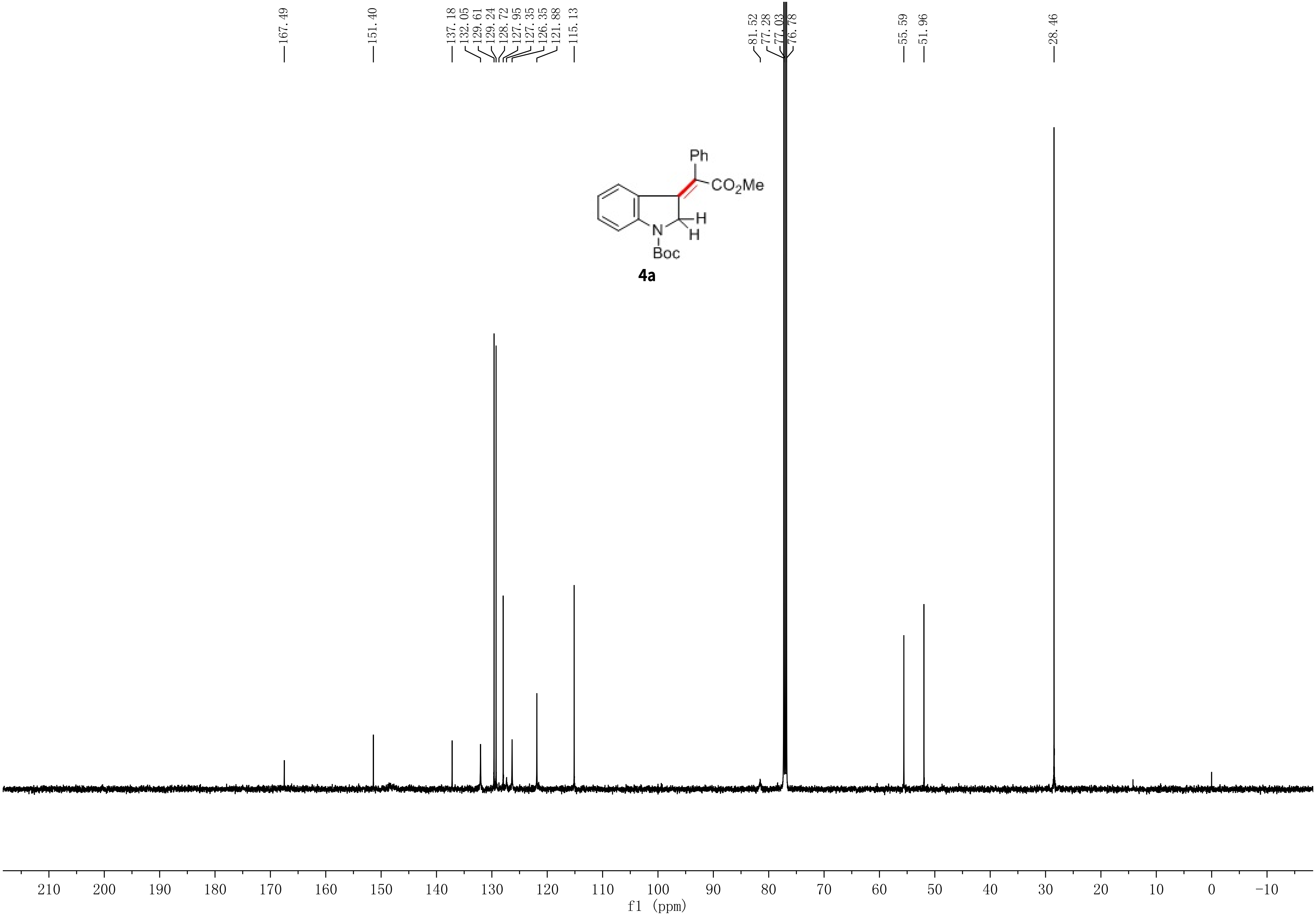
167.49
151.40
137.18
132.05
129.61
129.24
128.72
127.95
127.35
126.35
121.88
115.13
81.52
77.28
77.03
76.78
55.59
51.96
28.46
Ph
CO2Me
H
H
N
Boc
4a
210 200 190 180 170 160 150 140 130 120 110 100 90 80 70 60 50 40 30 20 10 0 −10
f1 (ppm)

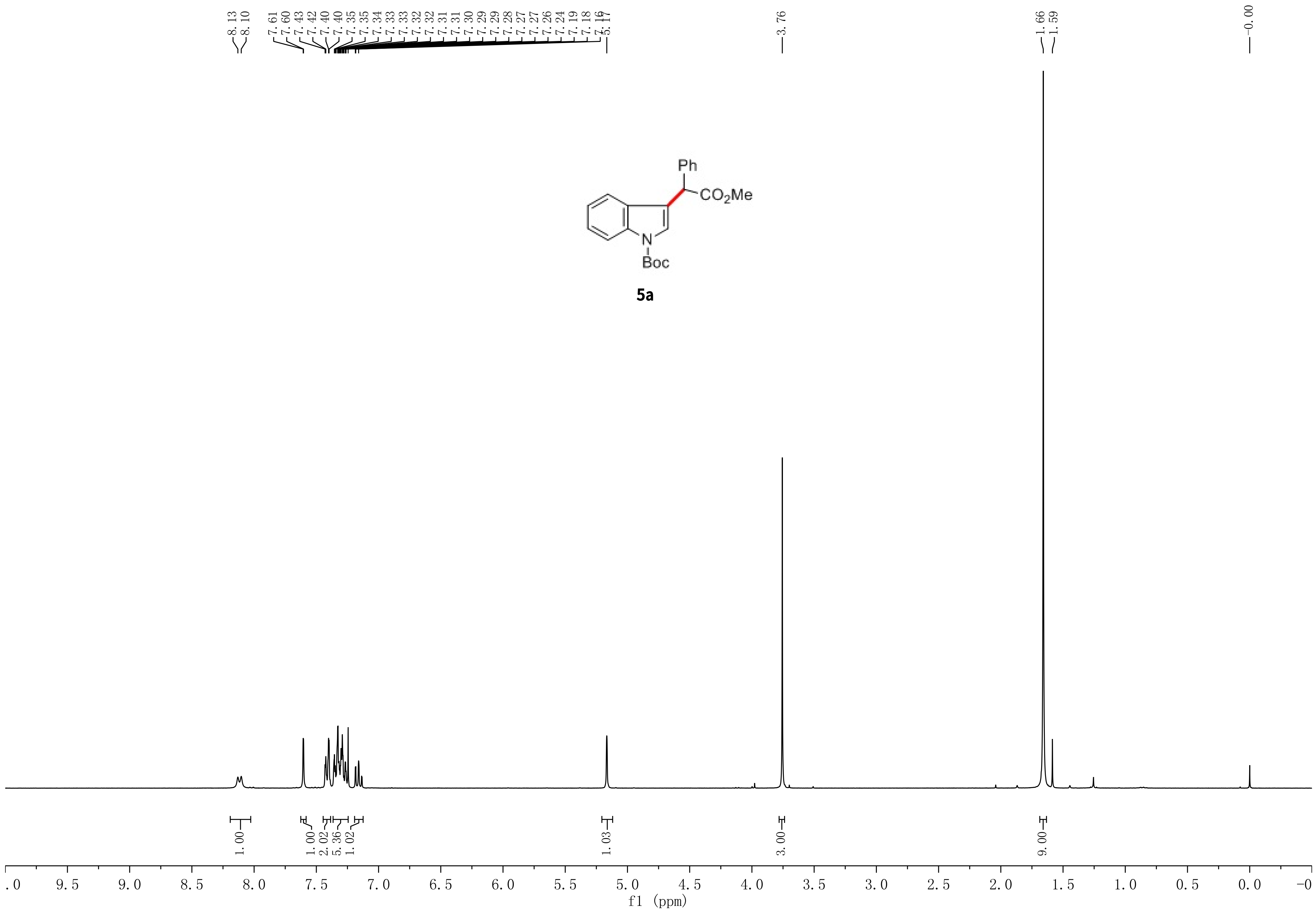
8.13
8.10
7.61
7.60
7.43
7.42
7.40
7.40
7.35
7.35
7.34
7.33
7.33
7.32
7.32
7.31
7.31
7.30
7.29
7.29
7.28
7.27
7.27
7.26
7.24
7.19
7.18
7.16
5.17
3.76
1.66
1.59
0.00
Ph
CO2Me
N
Boc
5a
1.00
1.00
2.02
5.36
1.02
1.03
3.00
9.00
f1 (ppm)

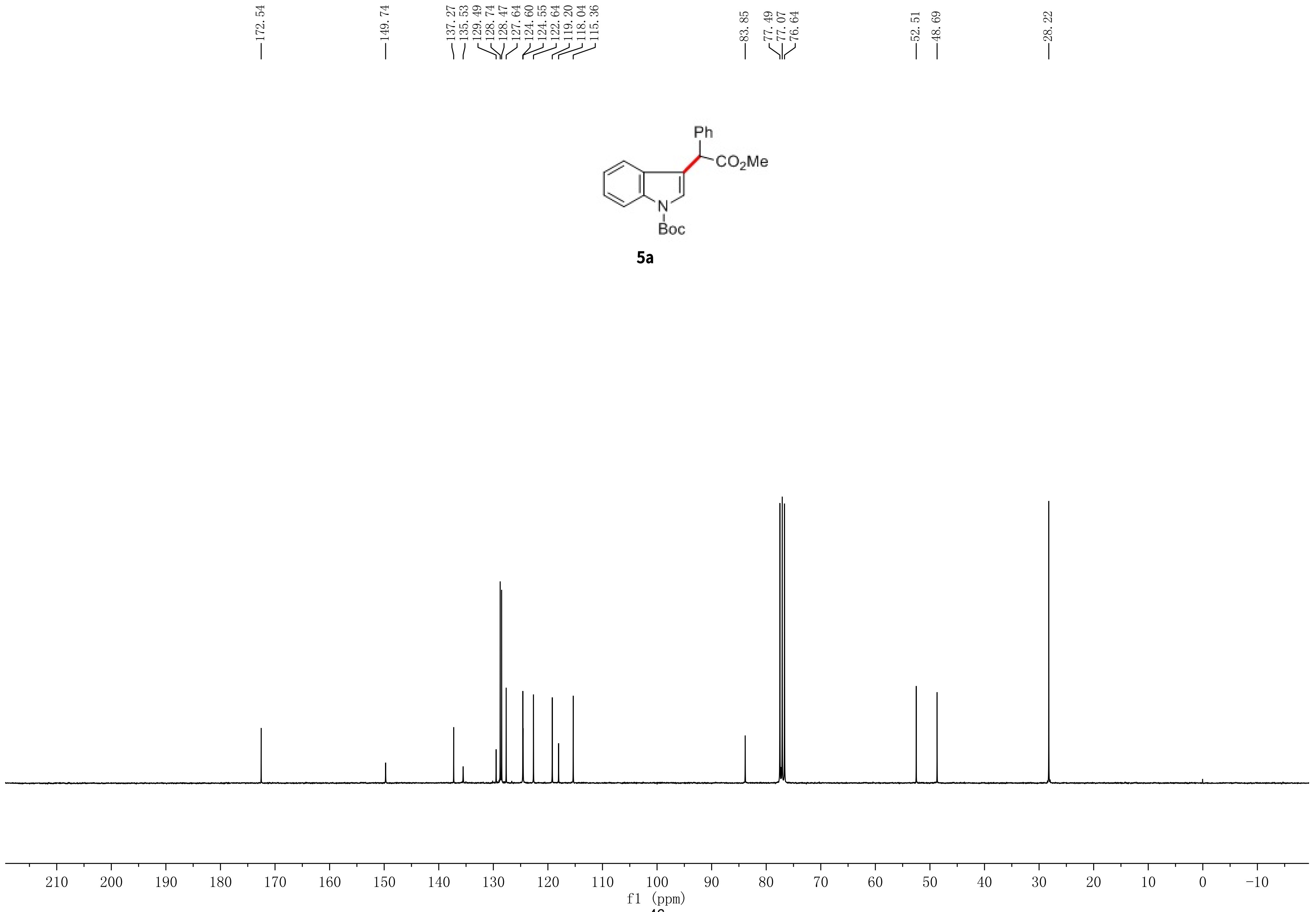
172.54
149.74
137.27
135.53
129.49
128.74
128.47
127.64
124.60
124.55
122.64
119.20
118.04
115.36
83.85
77.49
77.07
76.64
52.51
48.69
28.22
Ph
CO2Me
N
Boc
5a
210 200 190 180 170 160 150 140 130 120 110 100 90 80 70 60 50 40 30 20 10 0 −10
f1 (ppm)

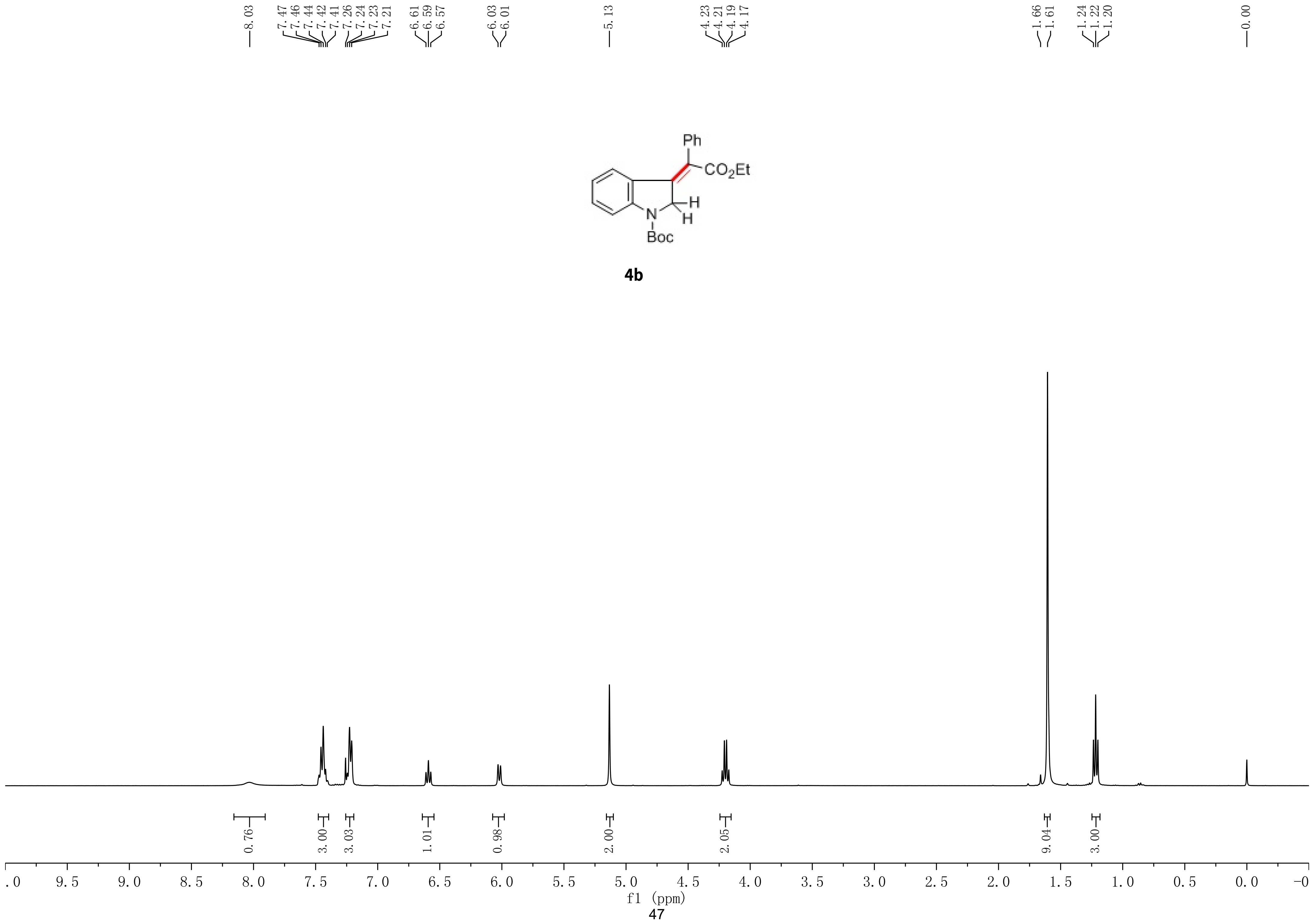
8.03
7.47 7.46 7.44 7.42 7.41 7.26 7.24 7.23 7.21
6.61 6.59 6.57
6.03 6.01
5.13
4.23 4.21 4.19 4.17
1.66 1.61
1.24 1.22 1.20
0.00
Ph
CO_2Et
H
H
N
Boc
4b
0.76
3.00
3.03
1.01
0.98
2.00
2.05
9.04
3.00
.0 9.5 9.0 8.5 8.0 7.5 7.0 6.5 6.0 5.5 5.0 4.5 4.0 3.5 3.0 2.5 2.0 1.5 1.0 0.5 0.0 -0
f1 (ppm)

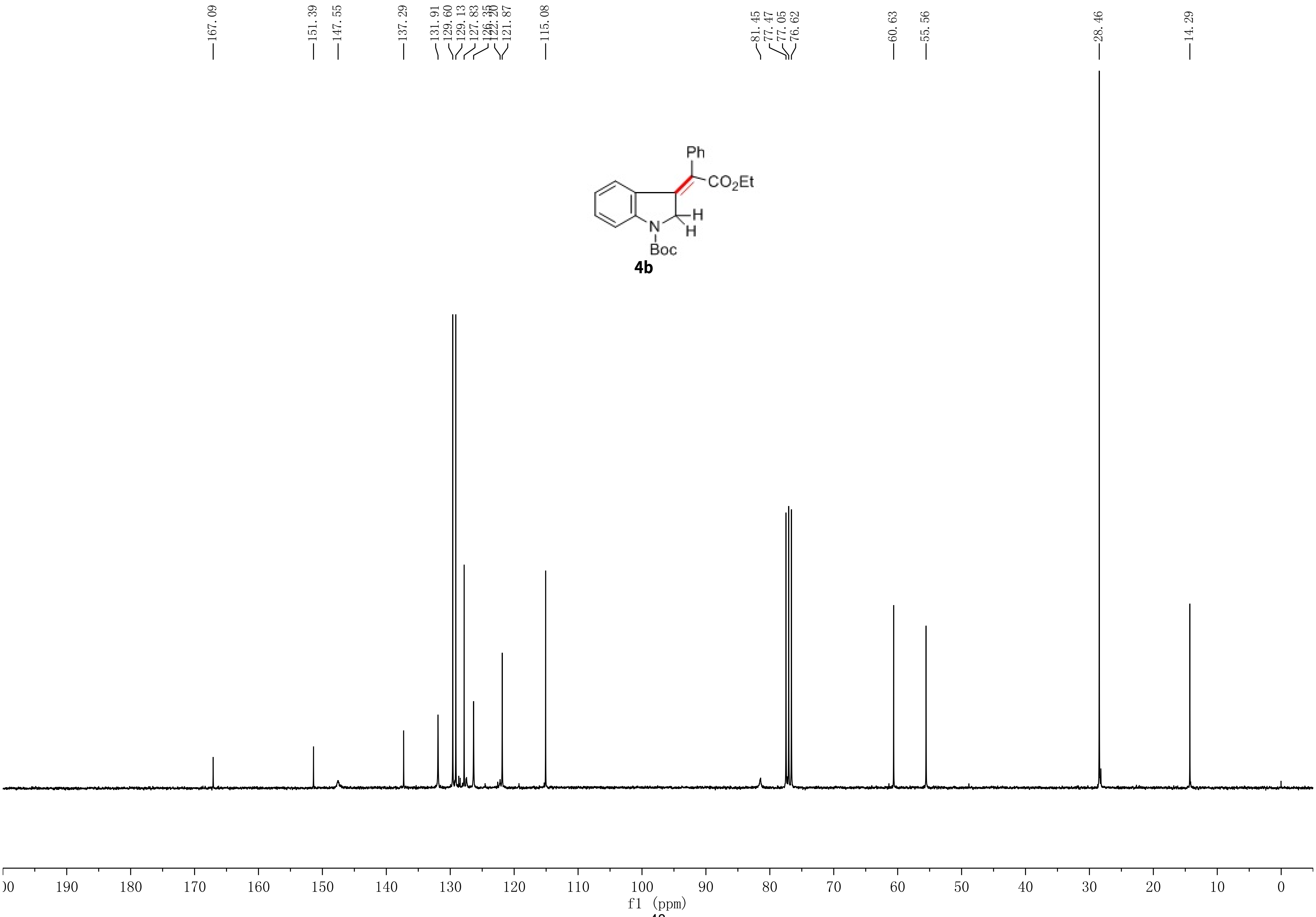
167.09
151.39
147.55
137.29
131.91
129.60
129.13
127.83
122.20
121.87
115.08
81.45
77.47
77.05
76.62
60.63
55.56
28.46
14.29
Ph
CO2Et
H
H
N
Boc
4b
190
180
170
160
150
140
130
120
110
100
90
80
70
60
50
40
30
20
10
0
f1 (ppm)

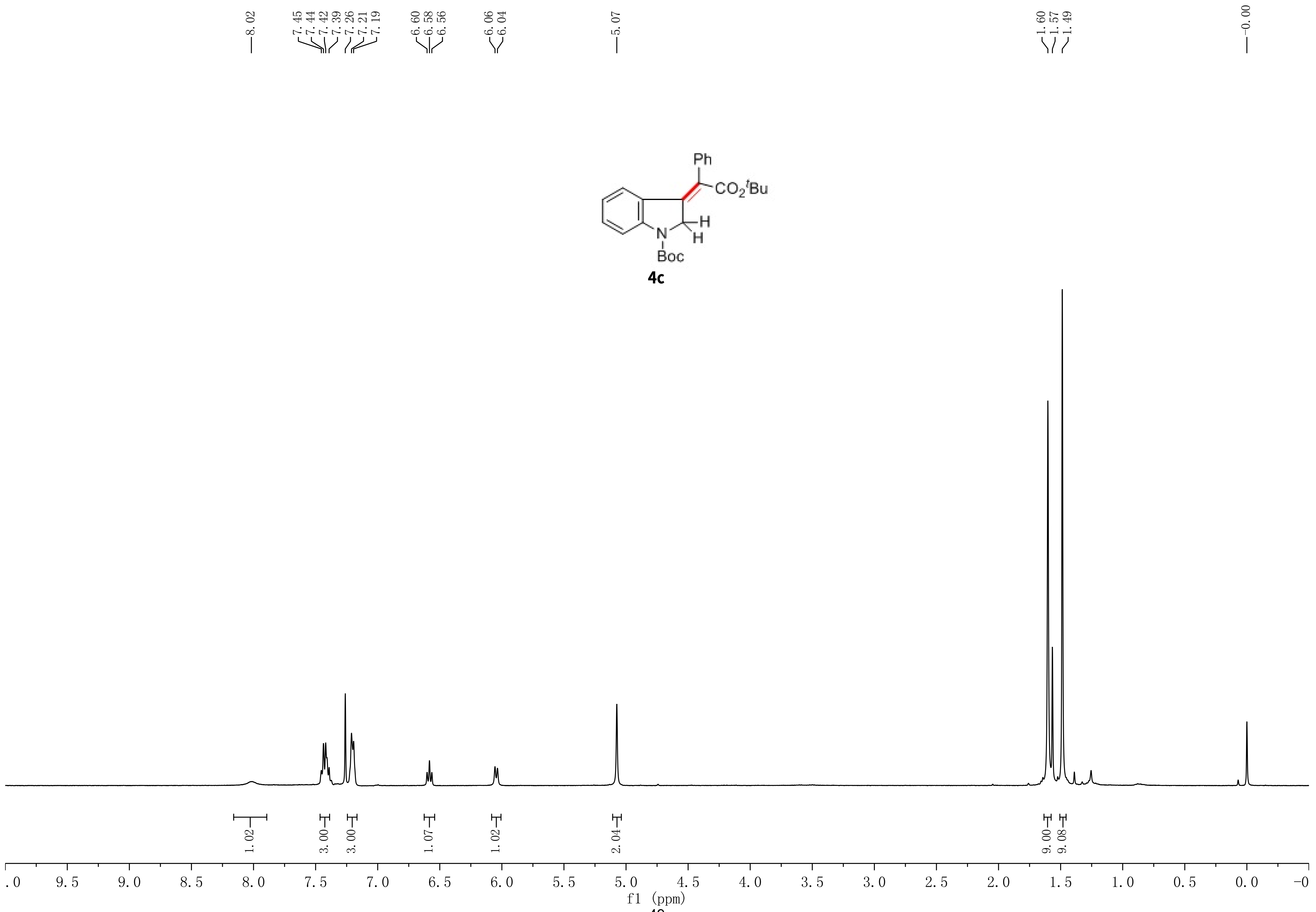
8.02
7.45
7.44
7.42
7.39
7.26
7.21
7.19
6.60
6.58
6.56
6.06
6.04
5.07
1.60
1.57
1.49
0.00
Ph
CO2tBu
H
H
N
Boc
4c
1.02
3.00
3.00
1.07
1.02
2.04
9.00
9.08
.0
9.5
9.0
8.5
8.0
7.5
7.0
6.5
6.0
5.5
5.0
4.5
4.0
3.5
3.0
2.5
2.0
1.5
1.0
0.5
0.0
-0
f1 (ppm)

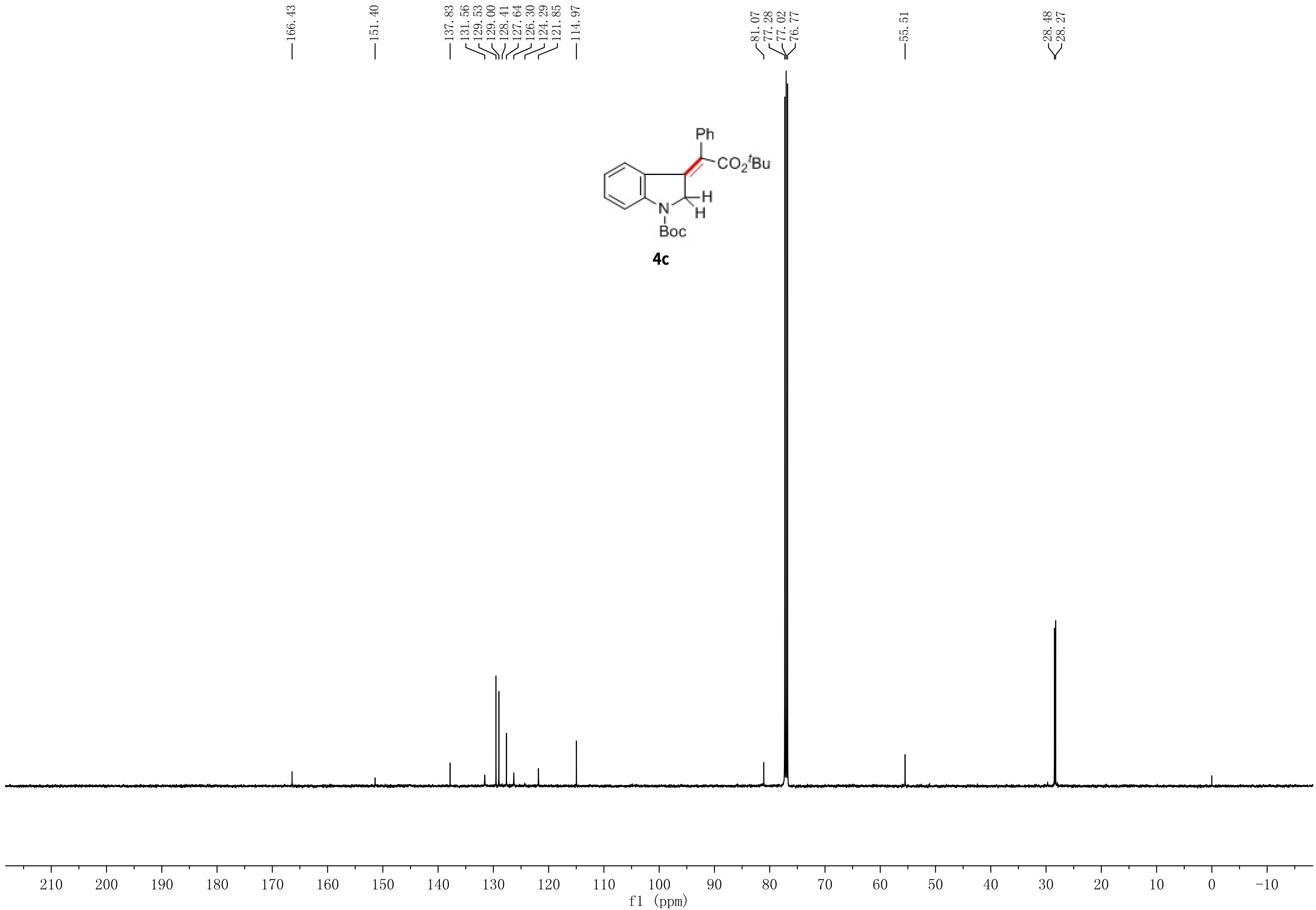
166.43
151.40
137.83
131.56
129.53
129.00
128.41
127.64
126.30
124.29
121.85
114.97
81.07
77.28
77.02
76.77
55.51
28.48
28.27
Ph
CO2tBu
H
H
N
Boc
4c
210 200 190 180 170 160 150 140 130 120 110 100 90 80 70 60 50 40 30 20 10 0 −10
f1 (ppm)

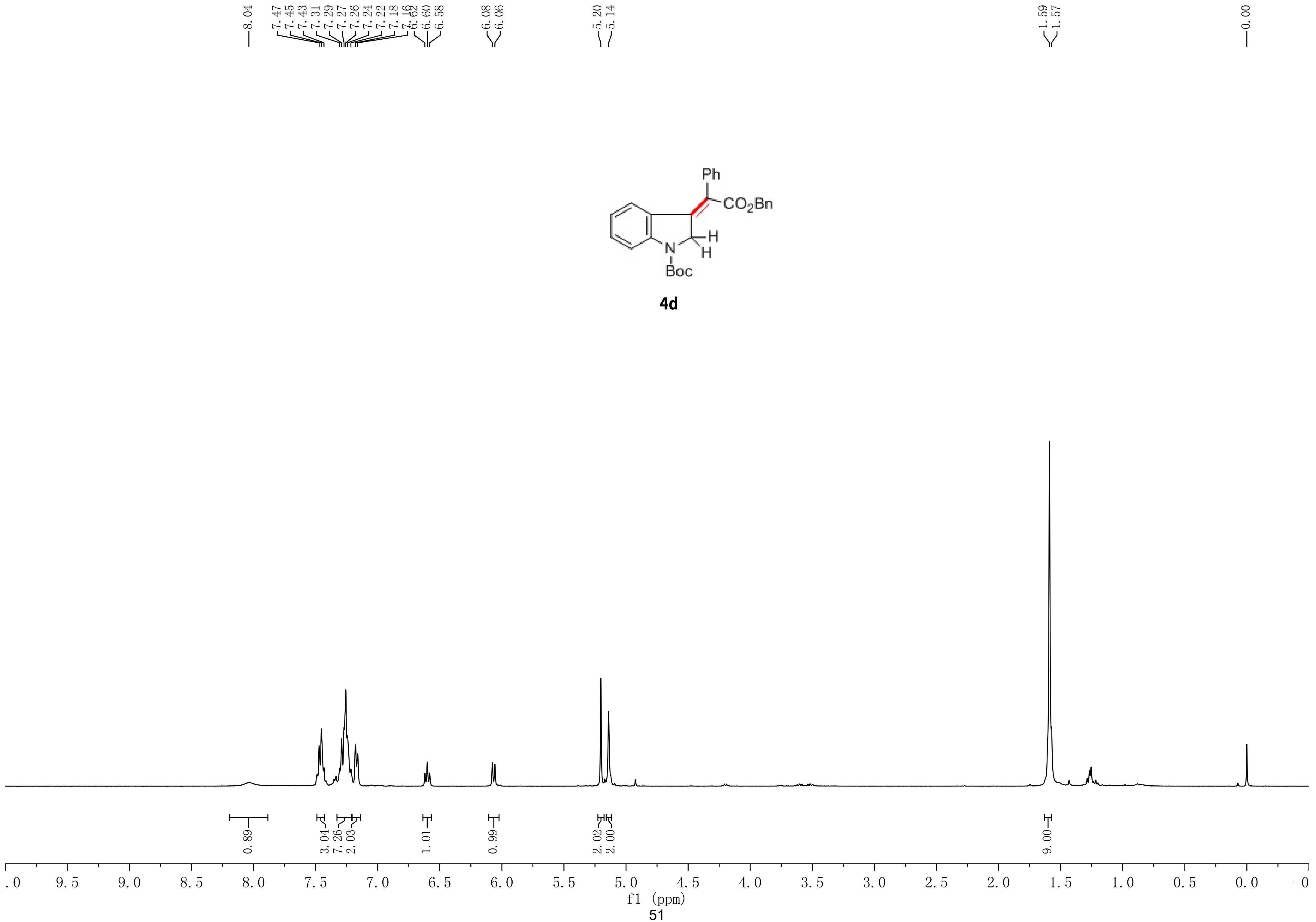
8.04
7.47
7.45
7.43
7.31
7.29
7.27
7.26
7.24
7.22
7.18
7.16
6.62
6.60
6.58
6.08
6.06
5.20
5.14
1.59
1.57
0.00
Ph
CO2Bn
H
H
N
Boc
4d
0.89
3.04
7.26
2.03
1.01
0.99
2.02
2.00
9.00
9.5
9.0
8.5
8.0
7.5
7.0
6.5
6.0
5.5
5.0
4.5
4.0
3.5
3.0
2.5
2.0
1.5
1.0
0.5
0.0
f1 (ppm)

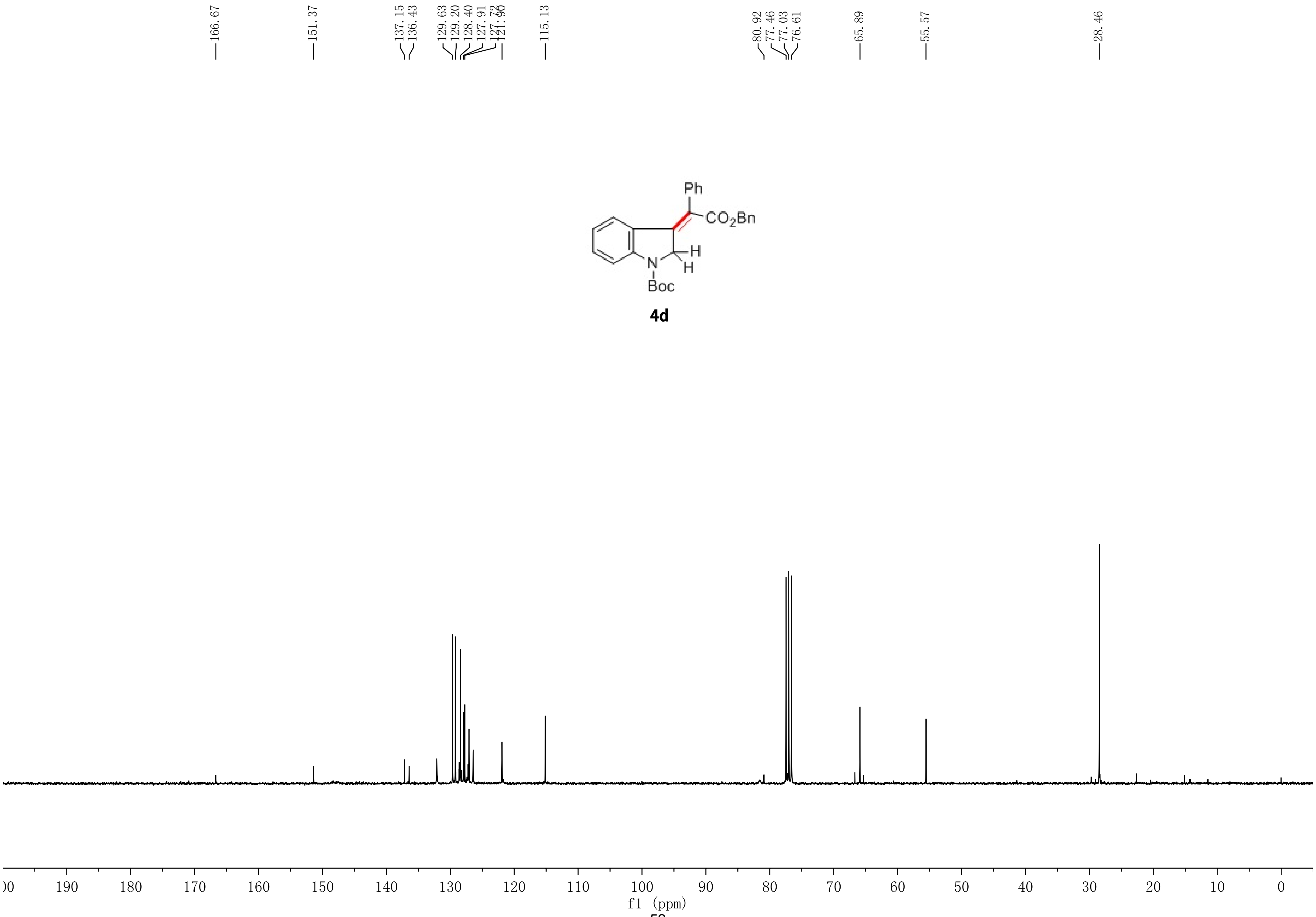
166.67
151.37
137.15
136.43
129.63
129.20
128.40
127.91
121.90
115.13
80.92
77.46
77.03
76.61
65.89
55.57
28.46
Ph
CO2Bn
H
H
N
Boc
4d
190 180 170 160 150 140 130 120 110 100 90 80 70 60 50 40 30 20 10 0
f1 (ppm)

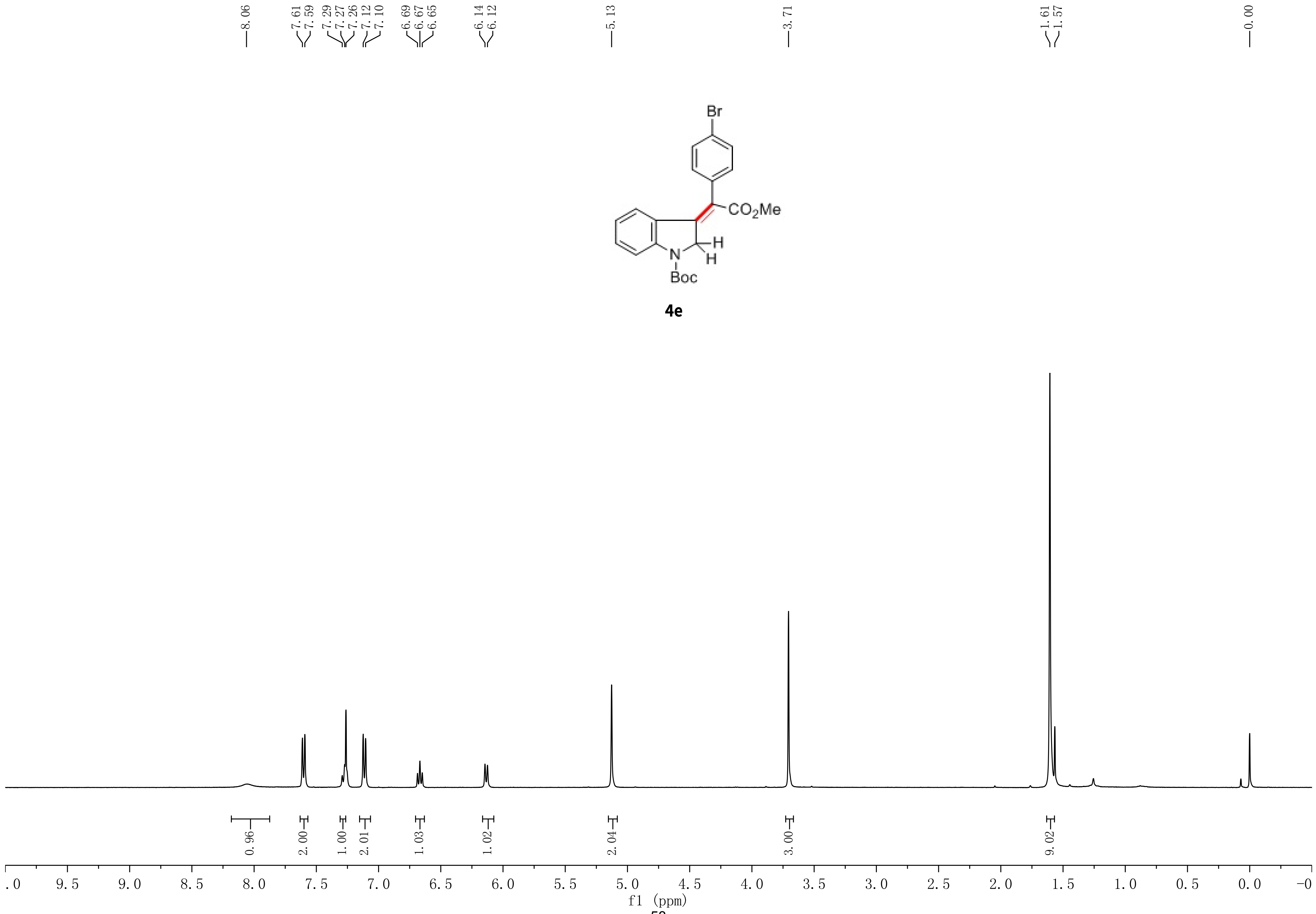
8.06
7.61
7.59
7.29
7.27
7.26
7.12
7.10
6.69
6.67
6.65
6.14
6.12
5.13
3.71
1.61
1.57
0.00
Br
CO2Me
N
Boc
H
H
4e
0.96
2.00
1.00
2.01
1.03
1.02
2.04
3.00
9.02
.0
9.5
9.0
8.5
8.0
7.5
7.0
6.5
6.0
5.5
5.0
4.5
4.0
3.5
3.0
2.5
2.0
1.5
1.0
0.5
0.0
-0
f1 (ppm)

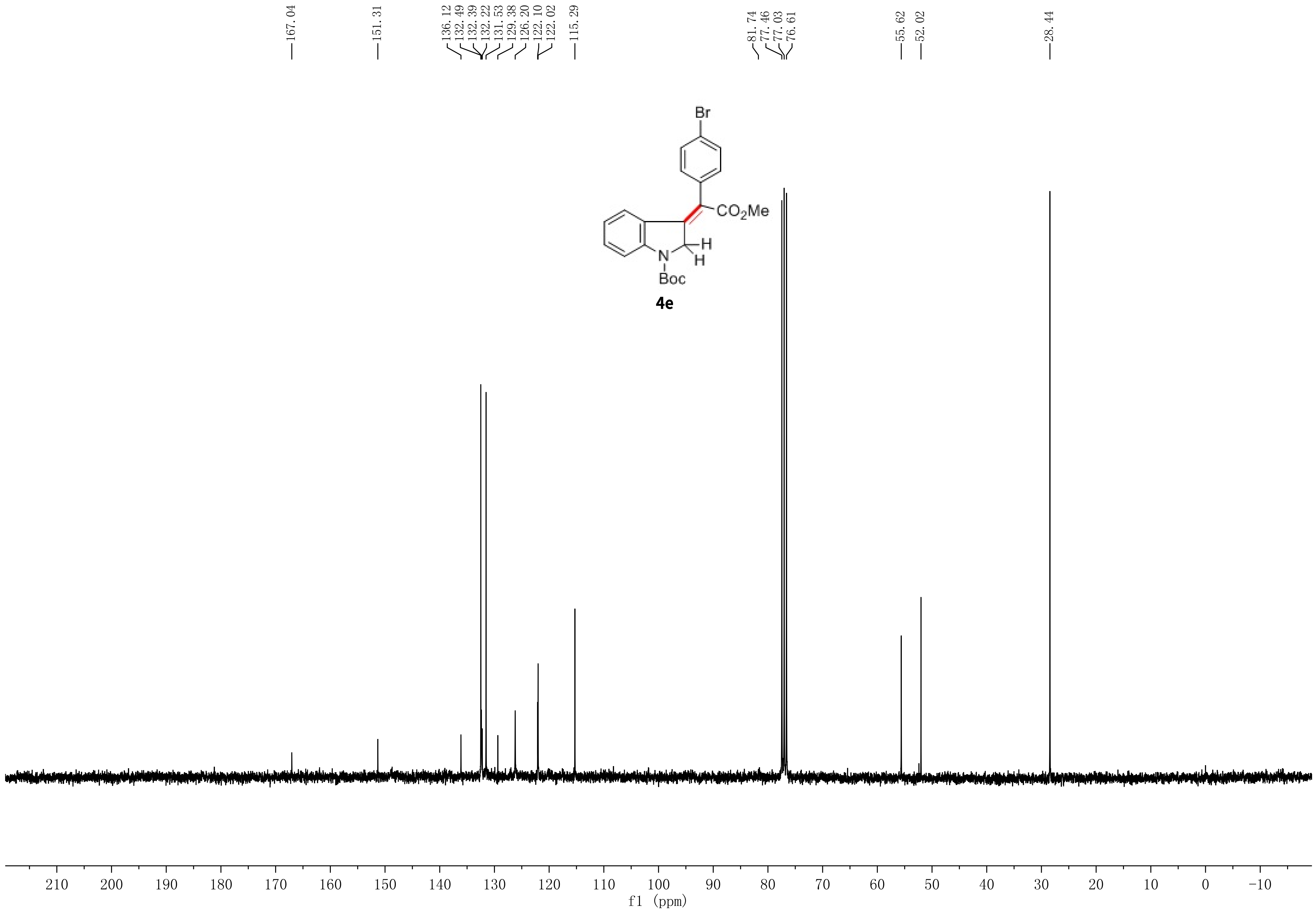
167.04
151.31
136.12
132.49
132.39
132.22
131.53
129.38
126.20
122.10
122.02
115.29
81.74
77.46
77.03
76.61
55.62
52.02
28.44
Br
CO2Me
H
H
N
Boc
4e
210 200 190 180 170 160 150 140 130 120 110 100 90 80 70 60 50 40 30 20 10 0 -10
f1 (ppm)

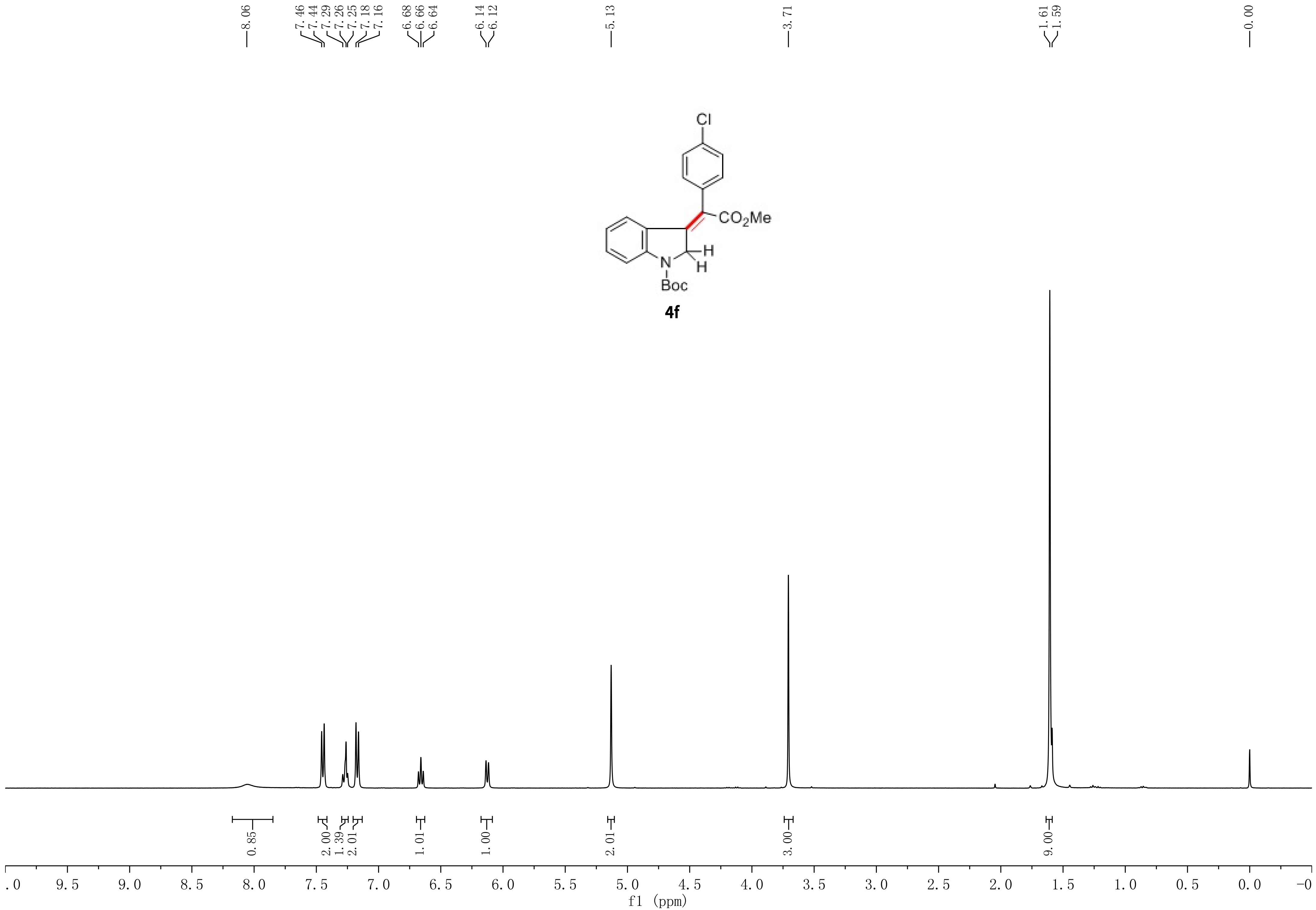

8.06
7.46
7.44
7.29
7.26
7.25
7.18
7.16
6.68
6.66
6.64
6.14
6.12
5.13
3.71
1.61
1.59
0.00
Cl
CO2Me
H
H
N
Boc
4f
0.85
2.00
1.39
2.01
1.01
1.00
2.01
3.00
9.00
f1 (ppm)

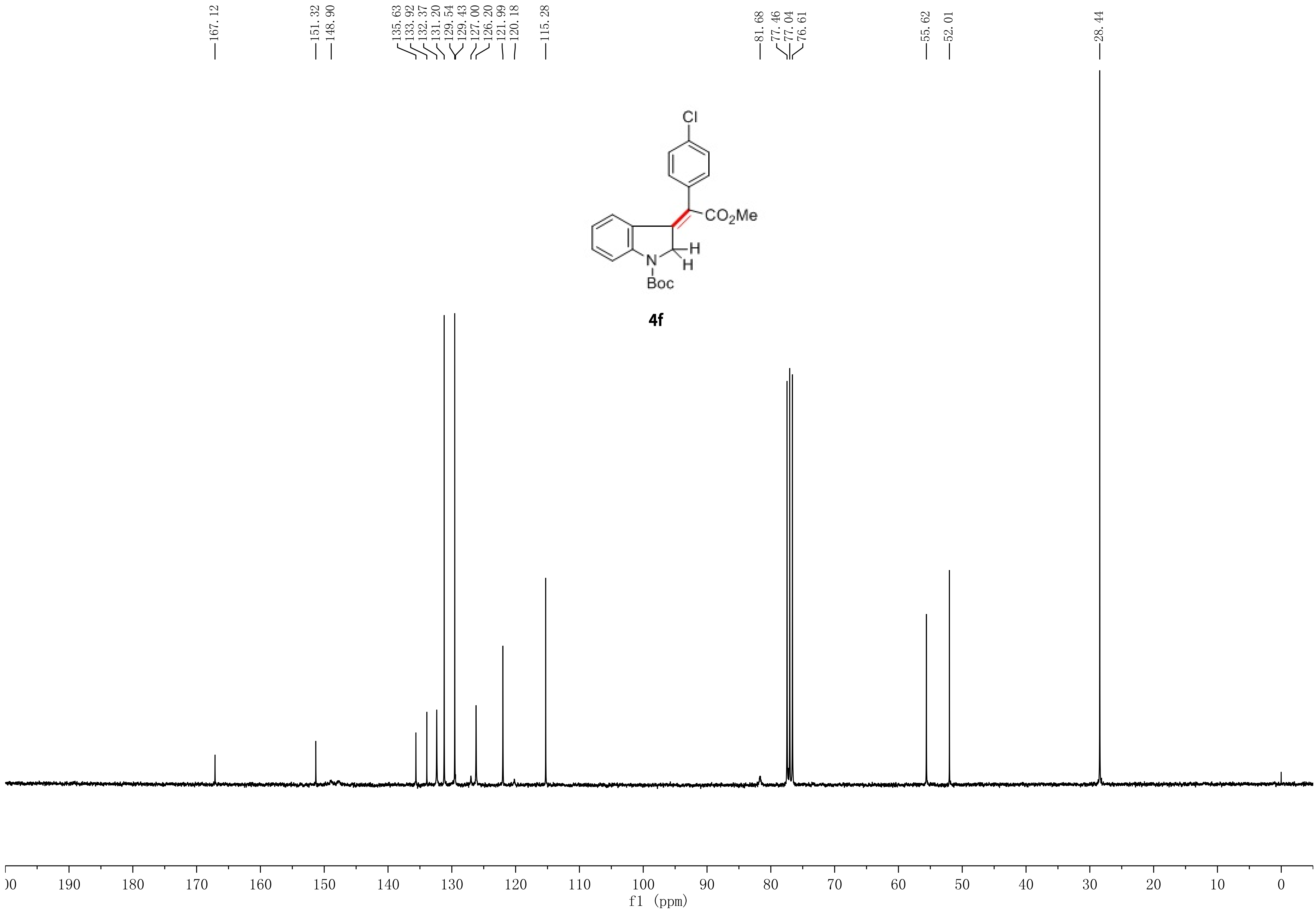

167.12
151.32
148.90
135.63
133.92
132.37
131.20
129.54
129.43
127.00
126.20
121.99
120.18
115.28
81.68
77.46
77.04
76.61
55.62
52.01
28.44
Cl
CO2Me
N
Boc
H
H
4f
190
180
170
160
150
140
130
120
110
100
90
80
70
60
50
40
30
20
10
0
f1 (ppm)

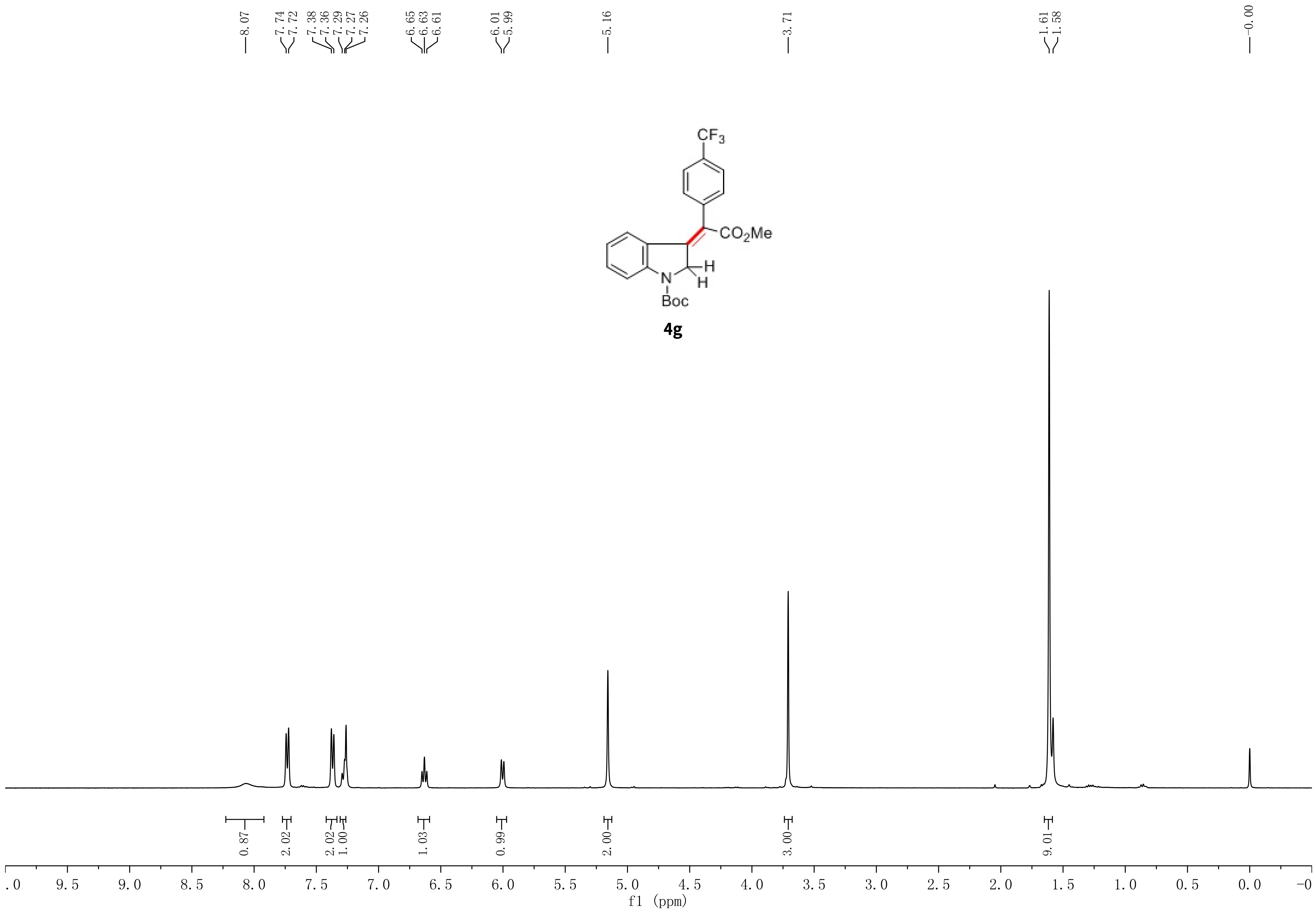
8.07
7.74
7.72
7.38
7.36
7.29
7.27
7.26
6.65
6.63
6.61
6.01
5.99
5.16
3.71
1.61
1.58
0.00
CF3
CO2Me
H
H
N
Boc
4g
0.87
2.02
2.02
1.00
1.03
0.99
2.00
3.00
9.01
f1 (ppm)

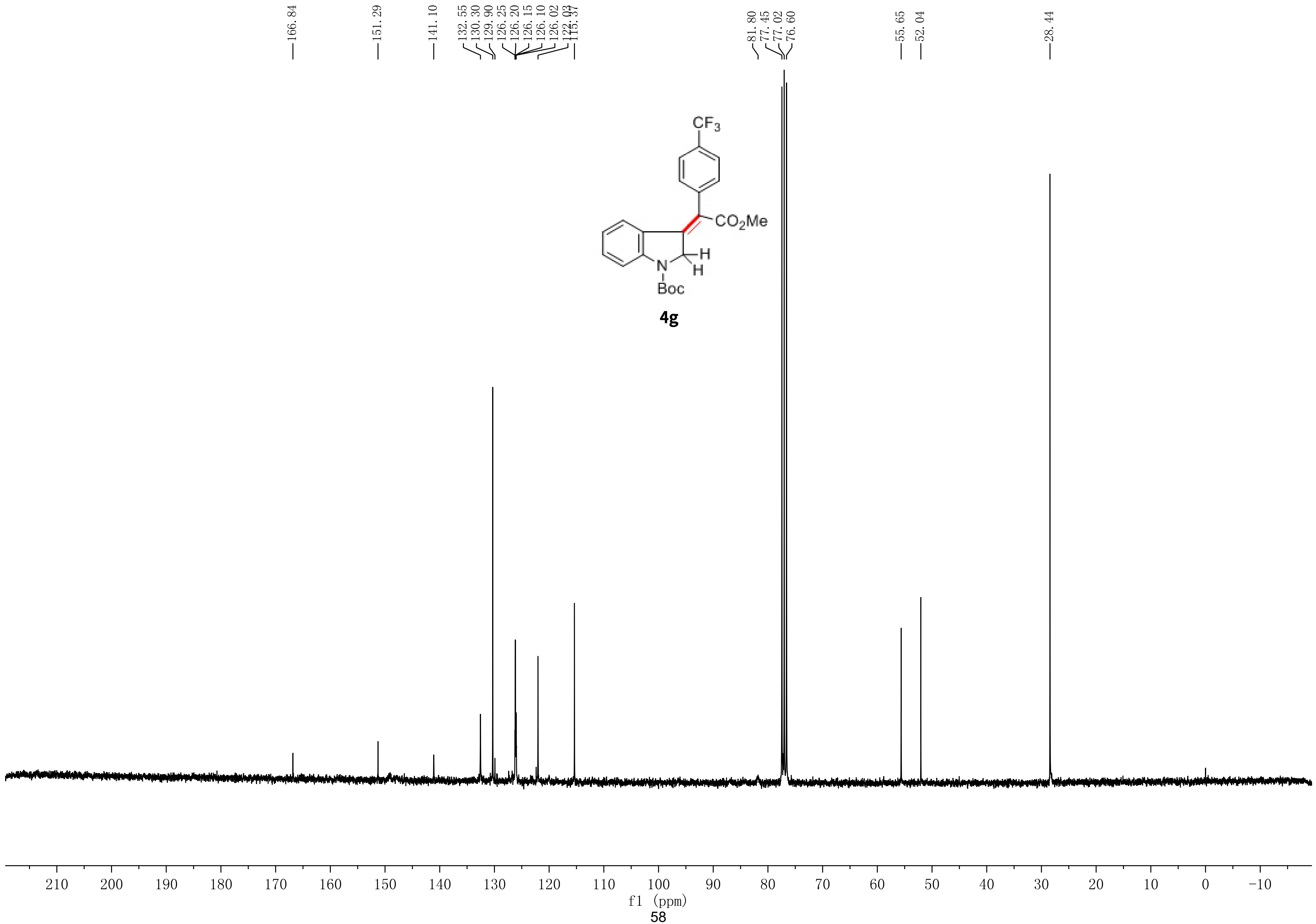
166.84
151.29
141.10
132.55
130.30
129.90
126.25
126.20
126.15
126.10
126.02
81.80
77.45
77.02
76.60
55.65
52.04
28.44
CF3
CO2Me
N
H
H
Boc
4g
210 200 190 180 170 160 150 140 130 120 110 100 90 80 70 60 50 40 30 20 10 0 −10
f1 (ppm)

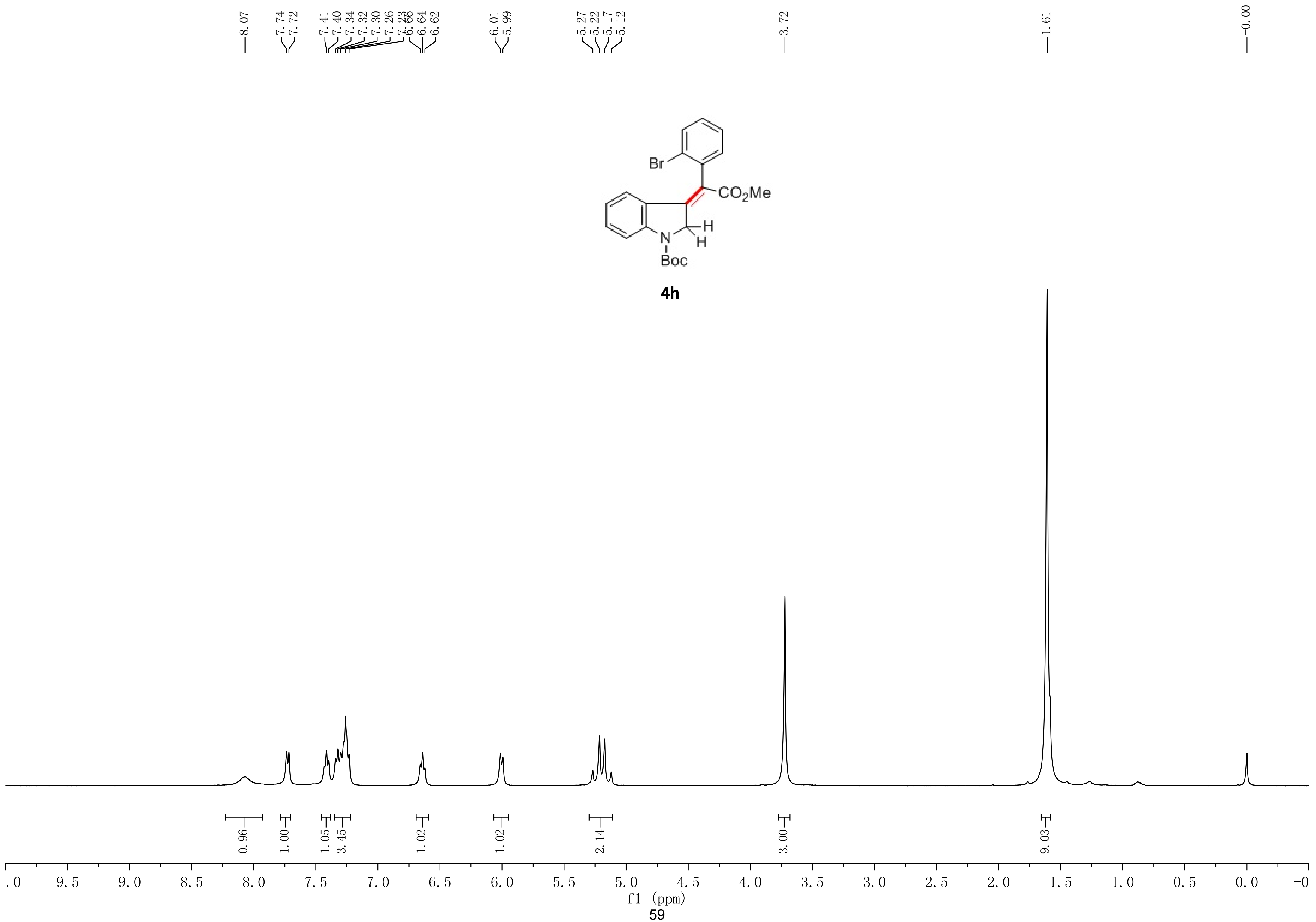
8.07
7.74
7.72
7.41
7.40
7.34
7.32
7.30
7.26
6.64
6.62
6.01
5.99
5.27
5.22
5.17
5.12
3.72
1.61
-0.00
Br
CO_2Me
H
H
N
Boc
0.96
1.00
1.05
3.45
1.02
1.02
2.14
3.00
9.03
9.5
9.0
8.5
8.0
7.5
7.0
6.5
6.0
5.5
5.0
4.5
4.0
3.5
3.0
2.5
2.0
1.5
1.0
0.5
0.0
f1 (ppm)

4h

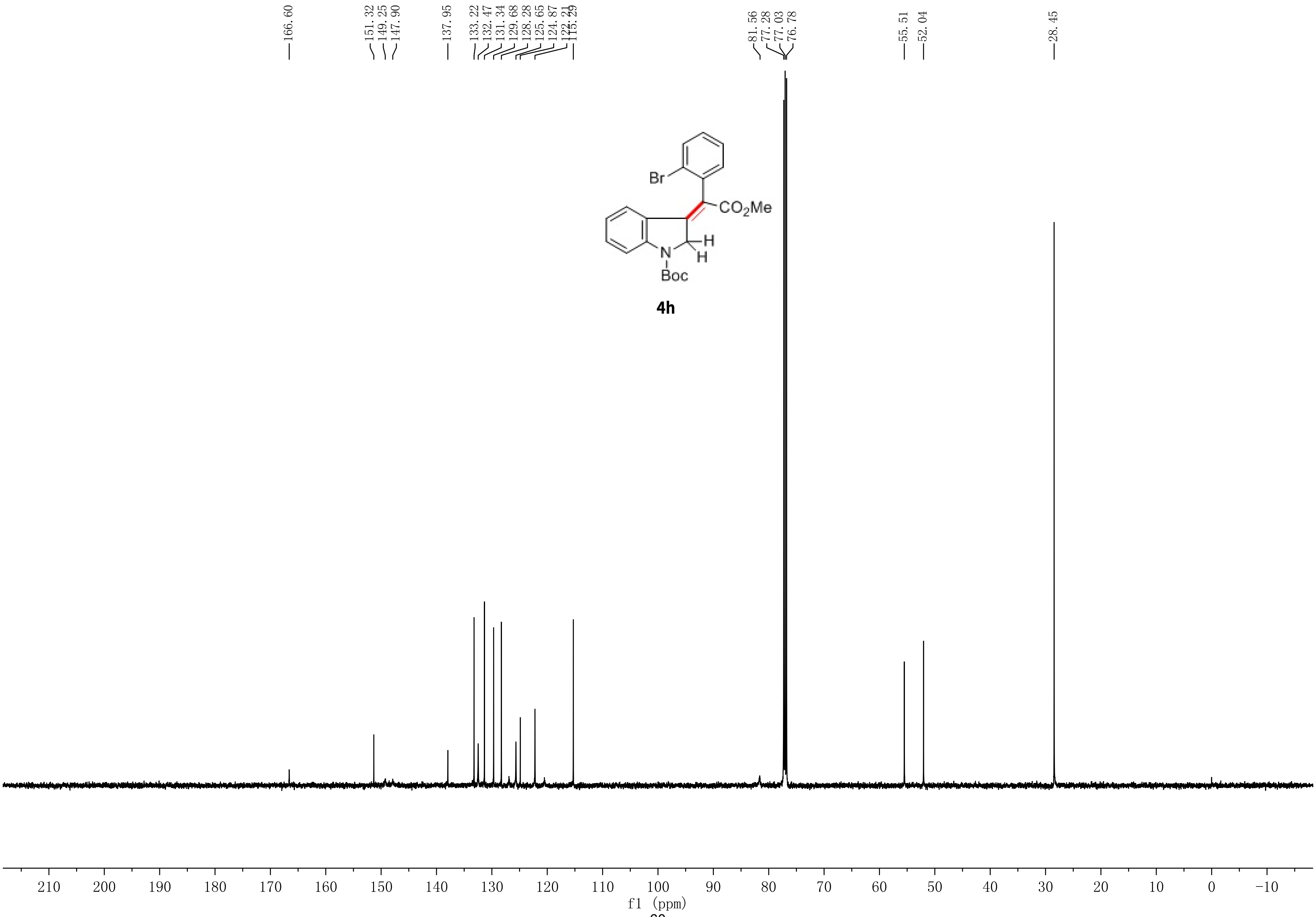
166.60
151.32
149.25
147.90
137.95
133.22
132.47
131.34
129.68
128.28
125.65
124.87
122.21
115.29
81.56
77.28
77.03
76.78
55.51
52.04
28.45
Br
CO_2Me
H
H
N
Boc
4h
210 200 190 180 170 160 150 140 130 120 110 100 90 80 70 60 50 40 30 20 10 0 -10
f1 (ppm)

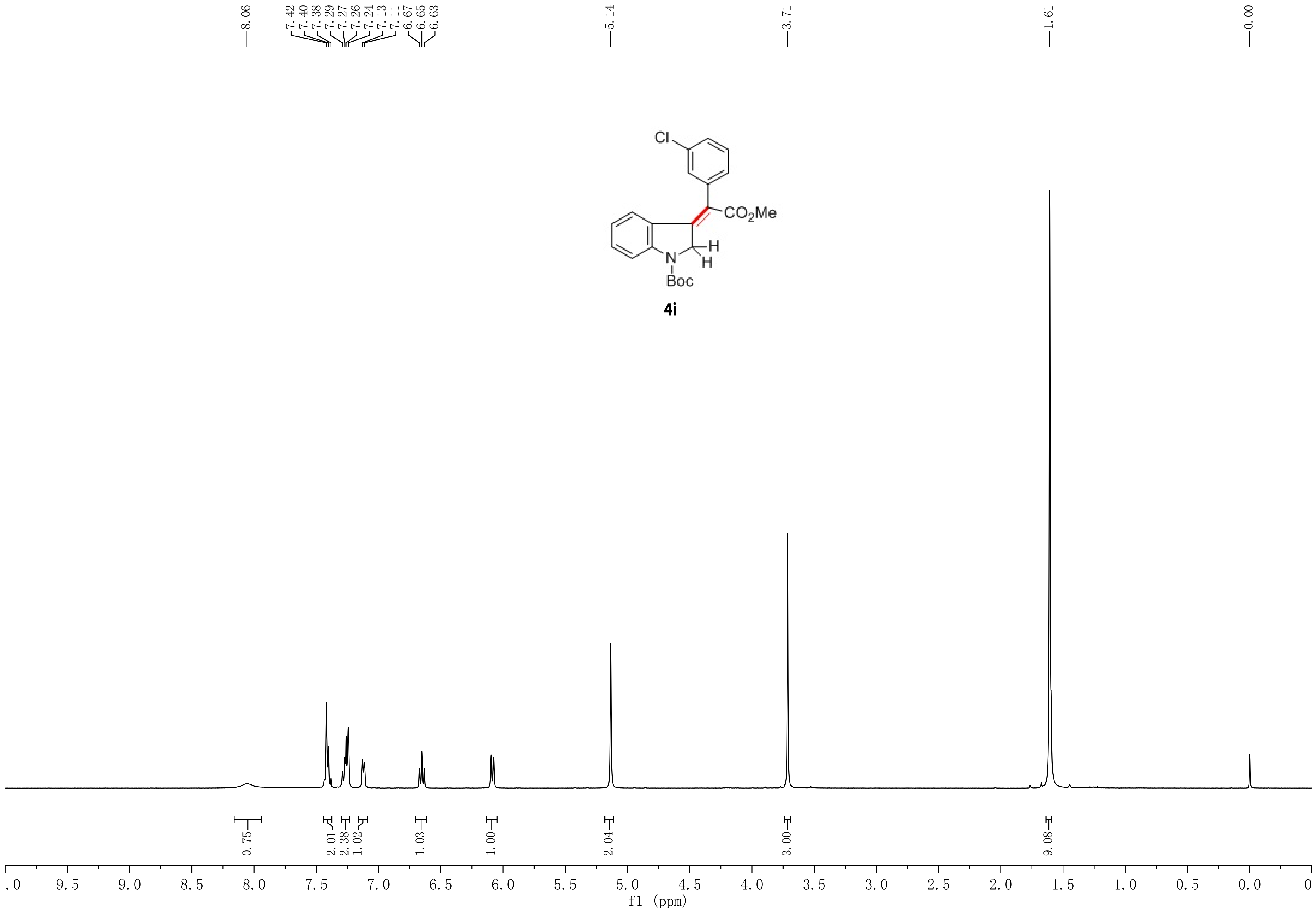
8.06
7.42
7.40
7.38
7.29
7.27
7.26
7.24
7.13
7.11
6.67
6.65
6.63
5.14
3.71
1.61
0.00
Cl
CO2Me
H
H
N
Boc
4i
0.75
2.01
2.38
1.02
1.03
1.00
2.04
3.00
9.08
f1 (ppm)

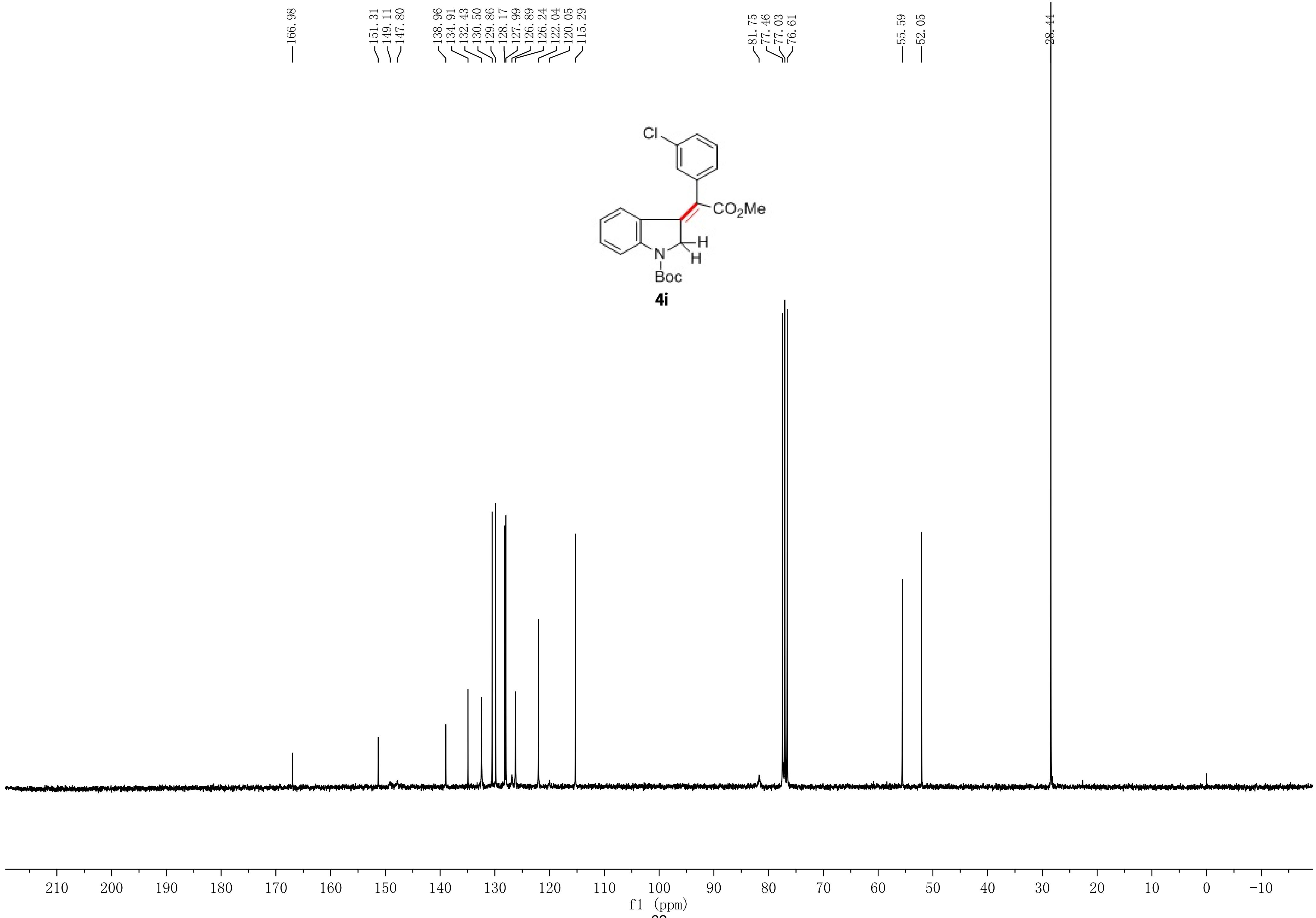
166.98
151.31
149.11
147.80
138.96
134.91
132.43
130.50
129.86
128.17
127.99
126.89
126.24
122.04
120.05
115.29
81.75
77.46
77.03
76.61
55.59
52.05
28.44
Cl
CO2Me
H
H
N
Boc
4i
210 200 190 180 170 160 150 140 130 120 110 100 90 80 70 60 50 40 30 20 10 0 -10
f1 (ppm)

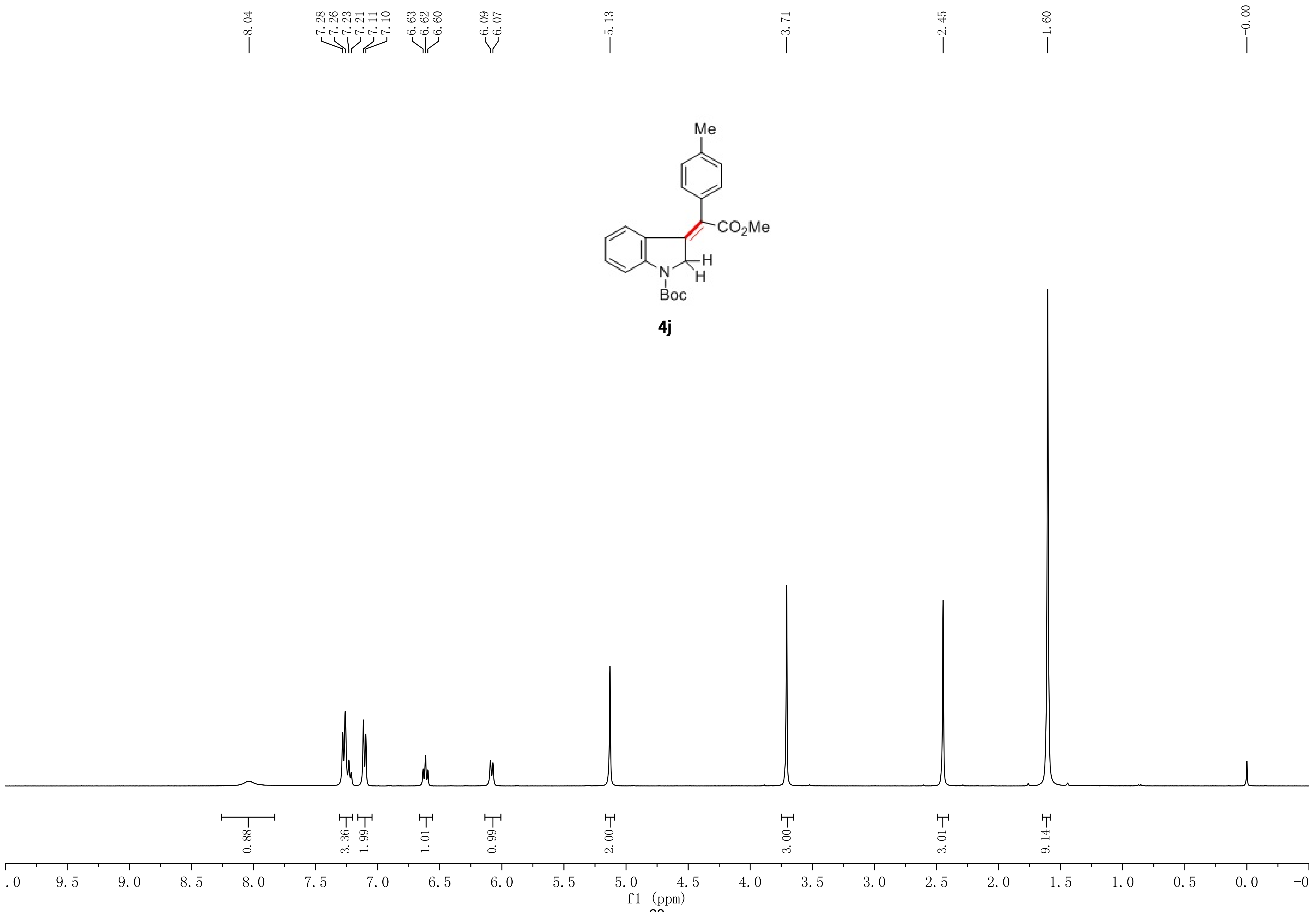
8.04
7.28
7.26
7.23
7.21
7.11
7.10
6.63
6.62
6.60
6.09
6.07
5.13
3.71
2.45
1.60
-0.00
Me
CO2Me
H
H
N
Boc
4j
0.88
3.36
1.99
1.01
0.99
2.00
3.00
3.01
9.14
.0
9.5
9.0
8.5
8.0
7.5
7.0
6.5
6.0
5.5
5.0
4.5
4.0
3.5
3.0
2.5
2.0
1.5
1.0
0.5
0.0
-0
f1 (ppm)

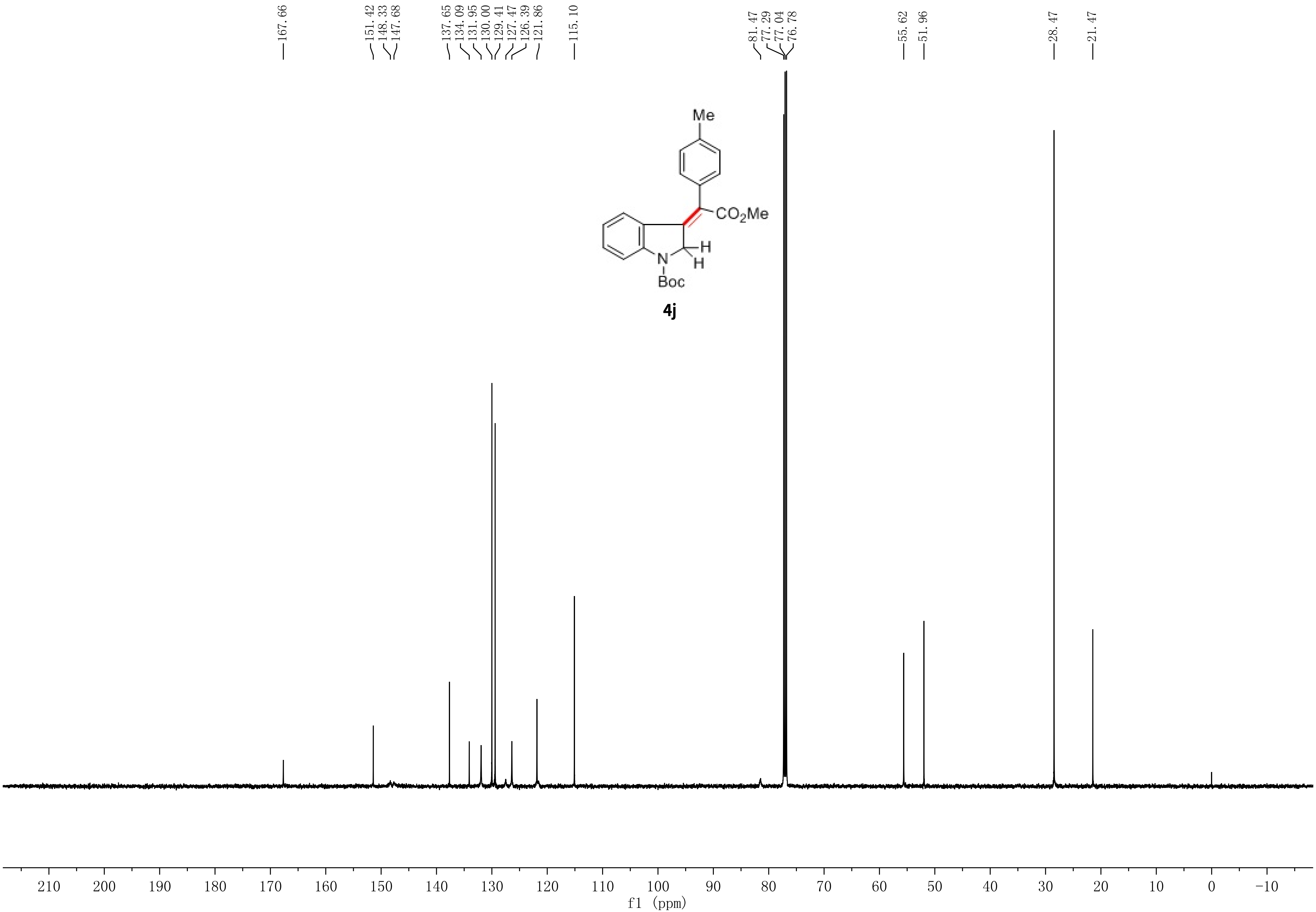
167.66
151.42
148.33
147.68
137.65
134.09
131.95
130.00
129.41
127.47
126.39
121.86
115.10
81.47
77.29
77.04
76.78
55.62
51.96
28.47
21.47
Me
CO_2Me
H
H
N
Boc
4j
210 200 190 180 170 160 150 140 130 120 110 100 90 80 70 60 50 40 30 20 10 0 −10
f1 (ppm)

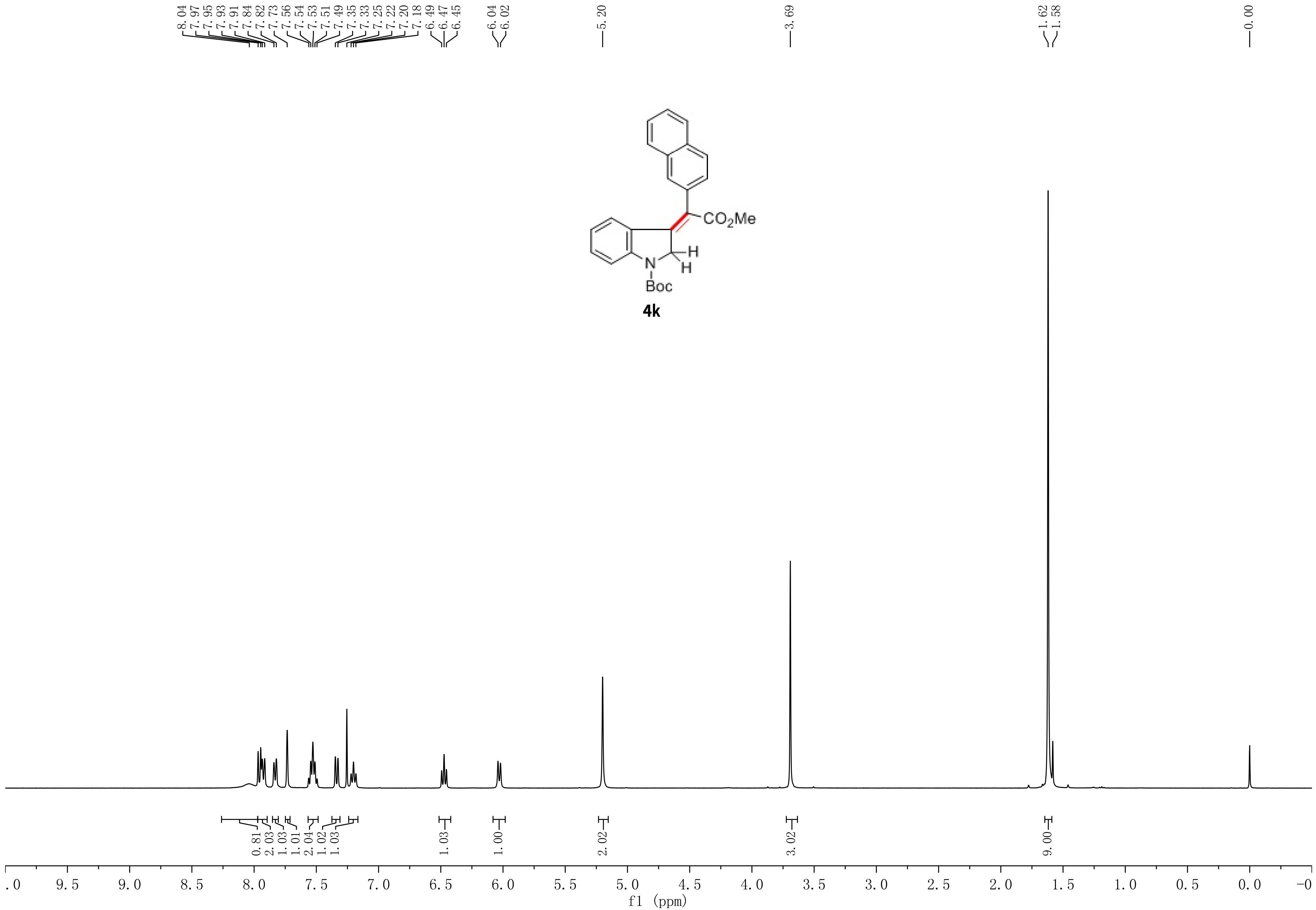
8.04
7.97
7.95
7.93
7.91
7.84
7.82
7.73
7.56
7.54
7.53
7.51
7.49
7.35
7.33
7.25
7.22
7.20
7.18
6.49
6.47
6.45
6.04
6.02
5.20
3.69
1.62
1.58
0.00
CO_2Me
H
H
N
Boc
4k
0.81
2.03
1.03
1.01
2.04
1.02
1.03
1.03
1.00
2.02
3.02
9.00
f1 (ppm)

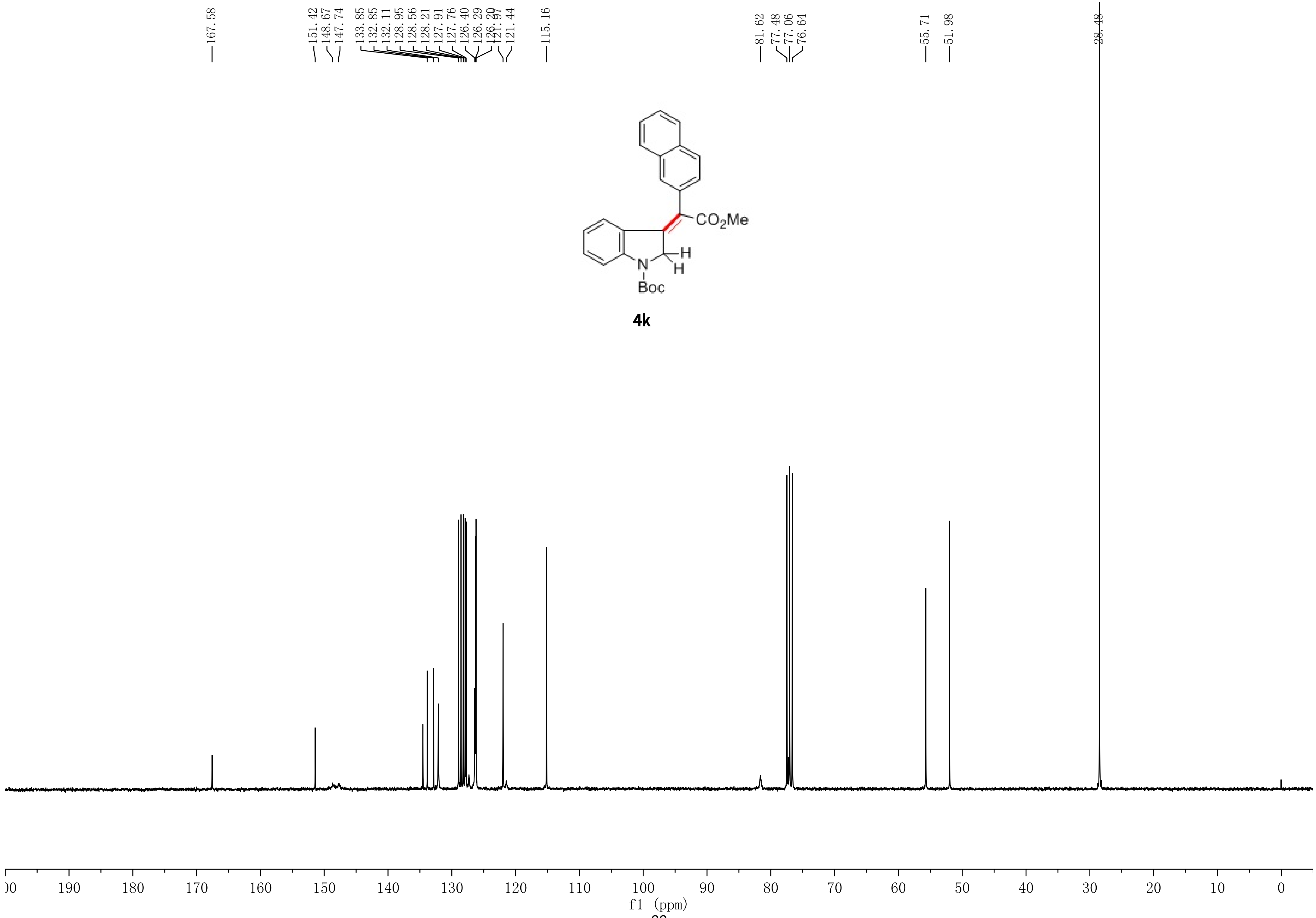
167.58
151.42
148.67
147.74
133.85
132.85
132.11
128.95
128.56
128.21
127.91
127.76
126.40
126.29
126.20
121.97
121.44
115.16
81.62
77.48
77.06
76.64
55.71
51.98
28.48
CO2Me
H
H
N
Boc
4k
190
180
170
160
150
140
130
120
110
100
90
80
70
60
50
40
30
20
10
0
f1 (ppm)

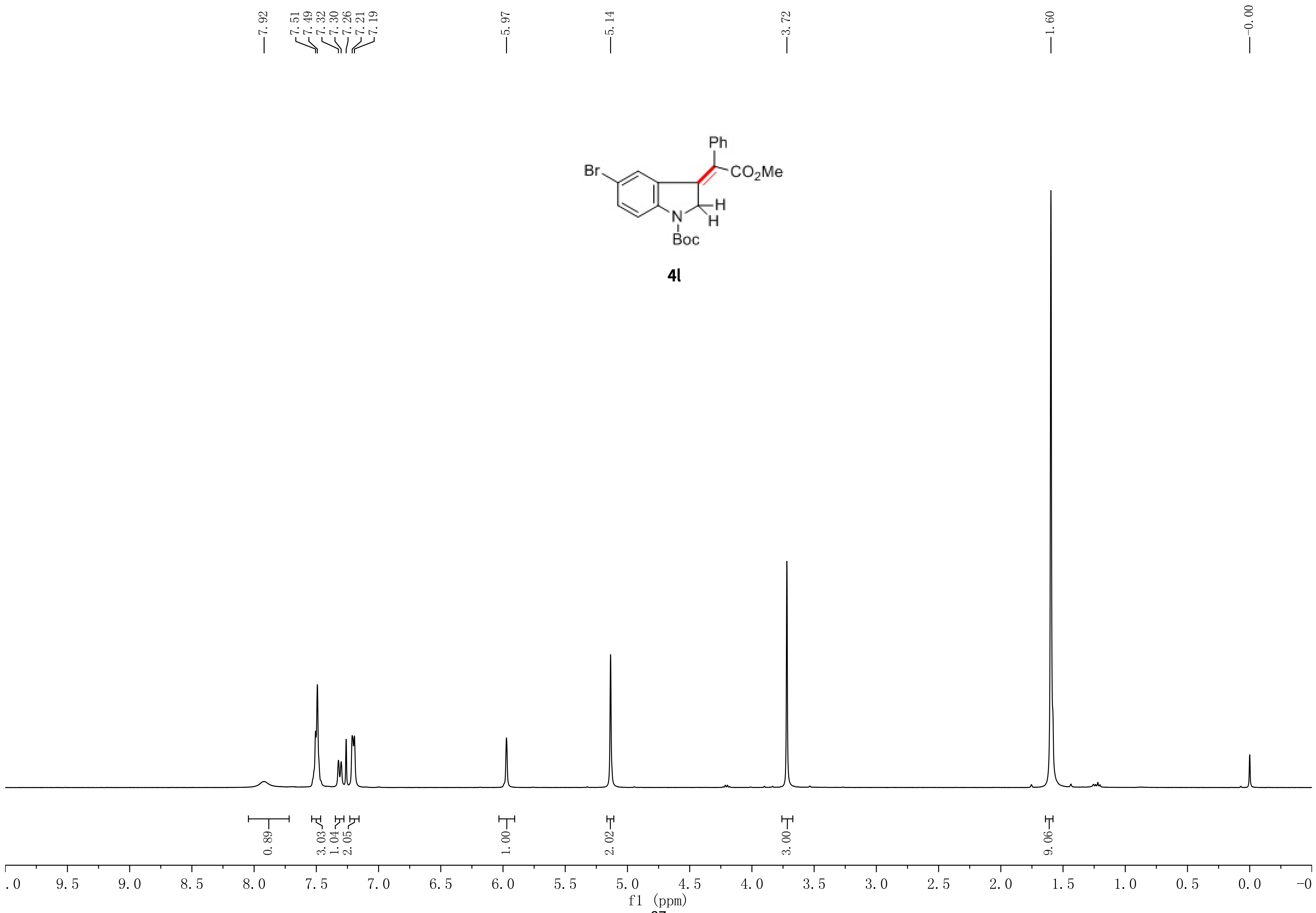
7.92
7.51
7.49
7.32
7.30
7.26
7.21
7.19
5.97
5.14
3.72
1.60
-0.00
Ph
Br
CO2Me
H
N
H
Boc
4l
0.89
3.03
1.04
2.05
1.00
2.02
3.00
9.06
f1 (ppm)

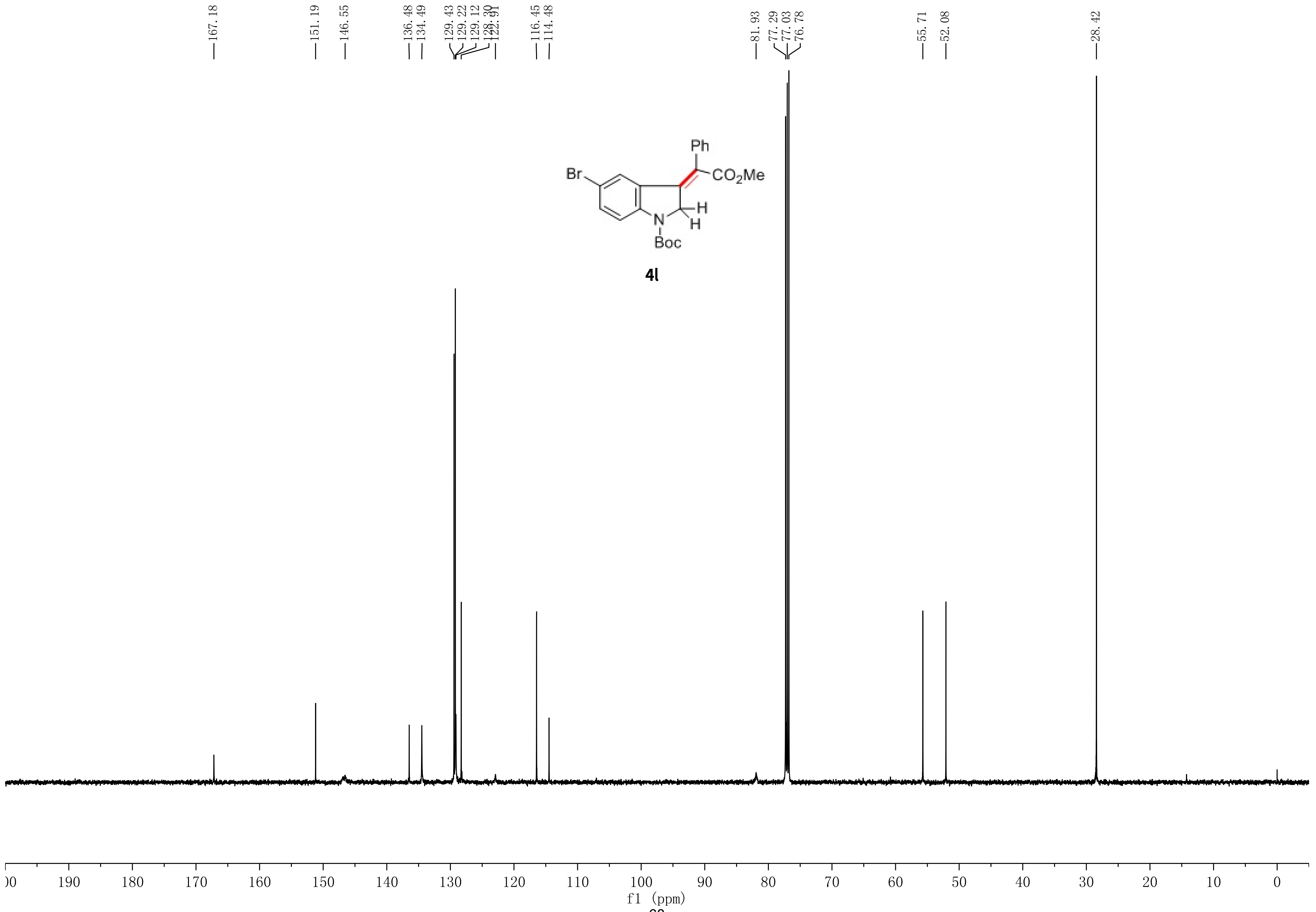
167.18
151.19
146.55
136.48
134.49
129.43
129.22
129.12
128.30
122.91
116.45
114.48
81.93
77.29
77.03
76.78
55.71
52.08
28.42
Ph
Br
CO2Me
H
H
N
Boc
4l
190 180 170 160 150 140 130 120 110 100 90 80 70 60 50 40 30 20 10 0
f1 (ppm)

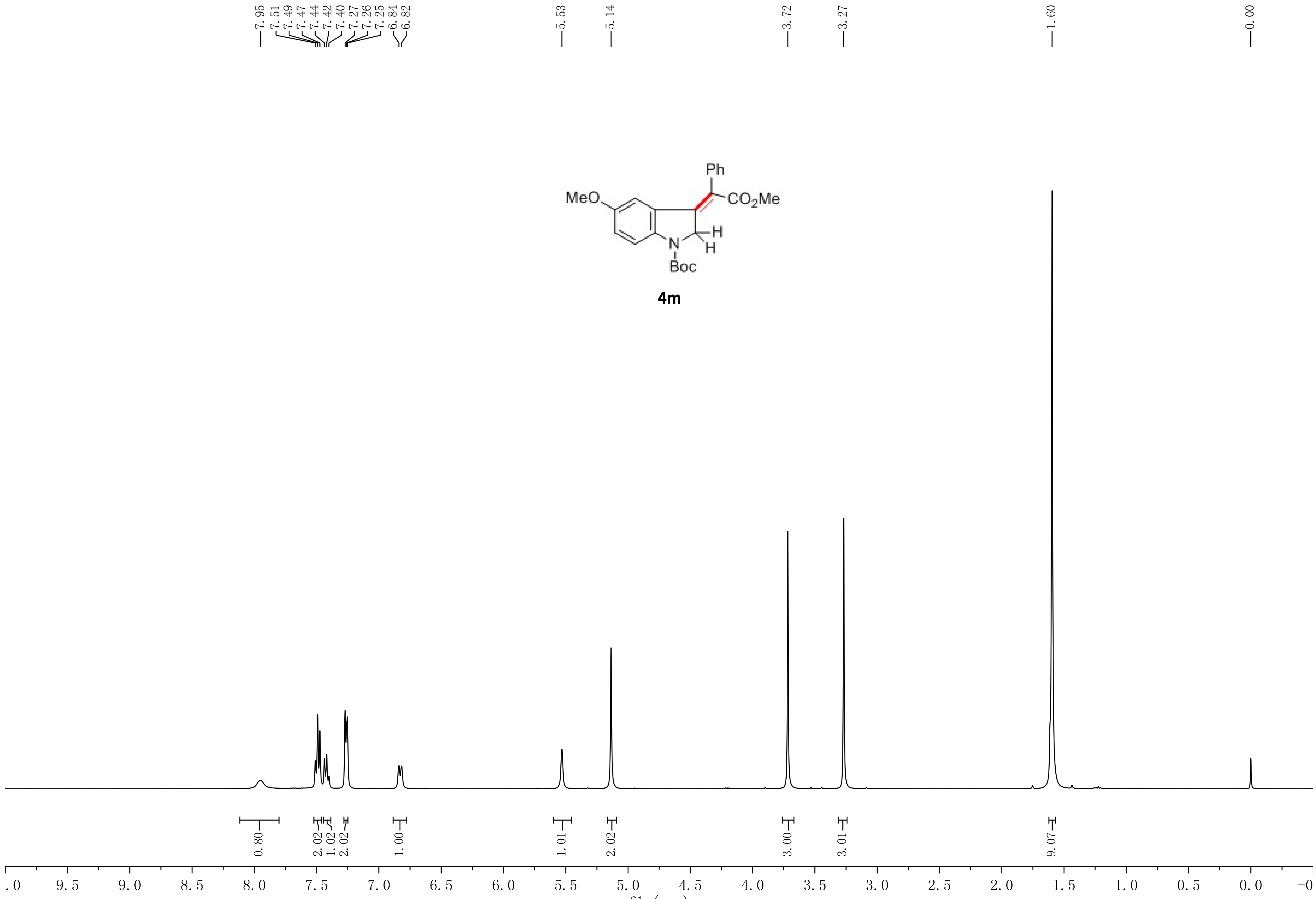
7.95
7.51
7.49
7.47
7.44
7.42
7.40
7.27
7.26
7.25
6.84
6.82
5.53
5.14
3.72
3.27
1.60
0.00
MeO
Ph
CO2Me
H
H
N
Boc
4m
0.80
2.02
1.02
2.02
1.00
1.01
2.02
3.00
3.01
9.07
9.5
9.0
8.5
8.0
7.5
7.0
6.5
6.0
5.5
5.0
4.5
4.0
3.5
3.0
2.5
2.0
1.5
1.0
0.5
0.0
f1 (ppm)

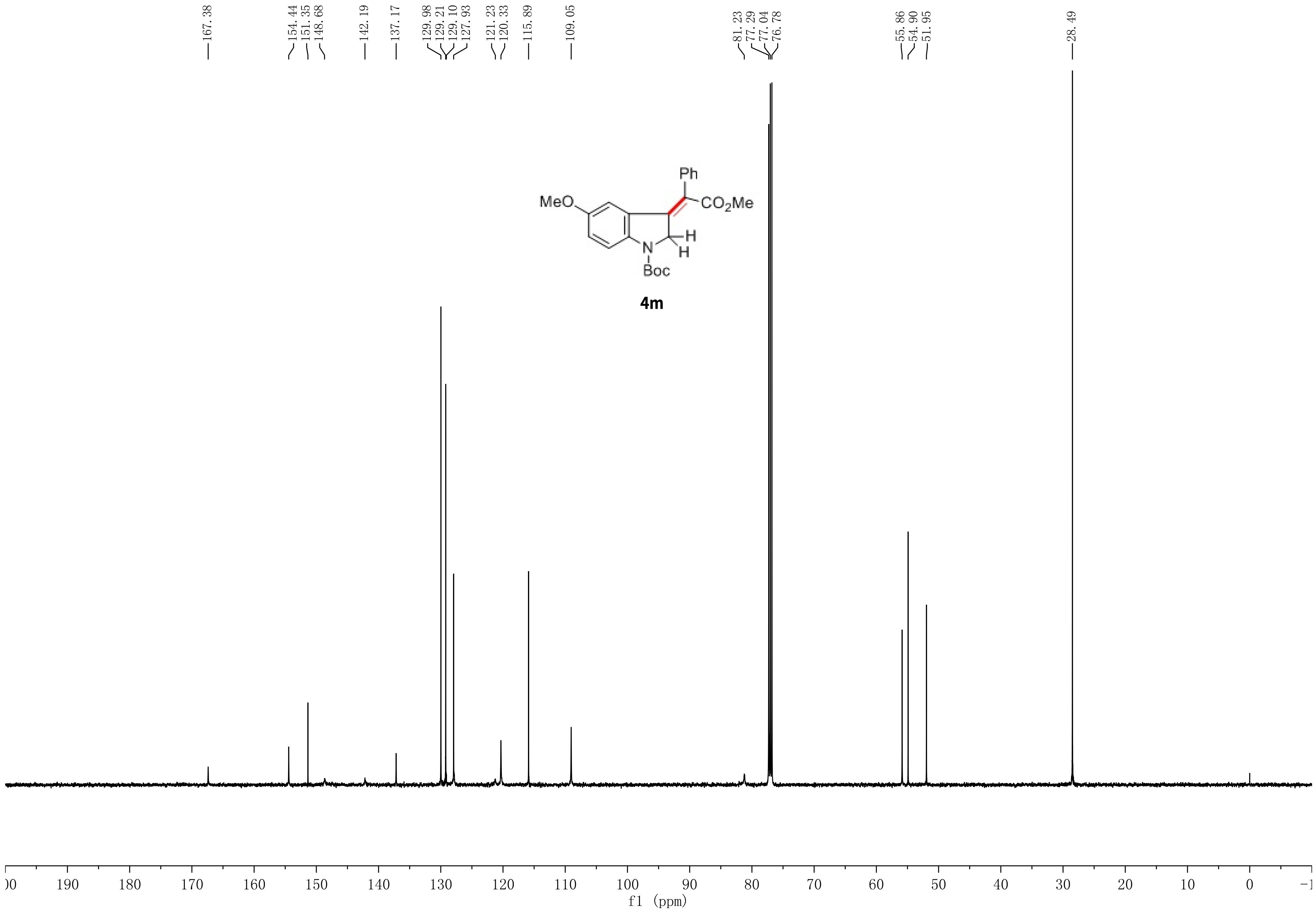
167.38
154.44
151.35
148.68
142.19
137.17
129.98
129.21
129.10
127.93
121.23
120.33
115.89
109.05
81.23
77.29
77.04
76.78
55.86
54.90
51.95
28.49
Ph
MeO
CO2Me
H
H
N
Boc
4m
190
180
170
160
150
140
130
120
110
100
90
80
70
60
50
40
30
20
10
0
f1 (ppm)

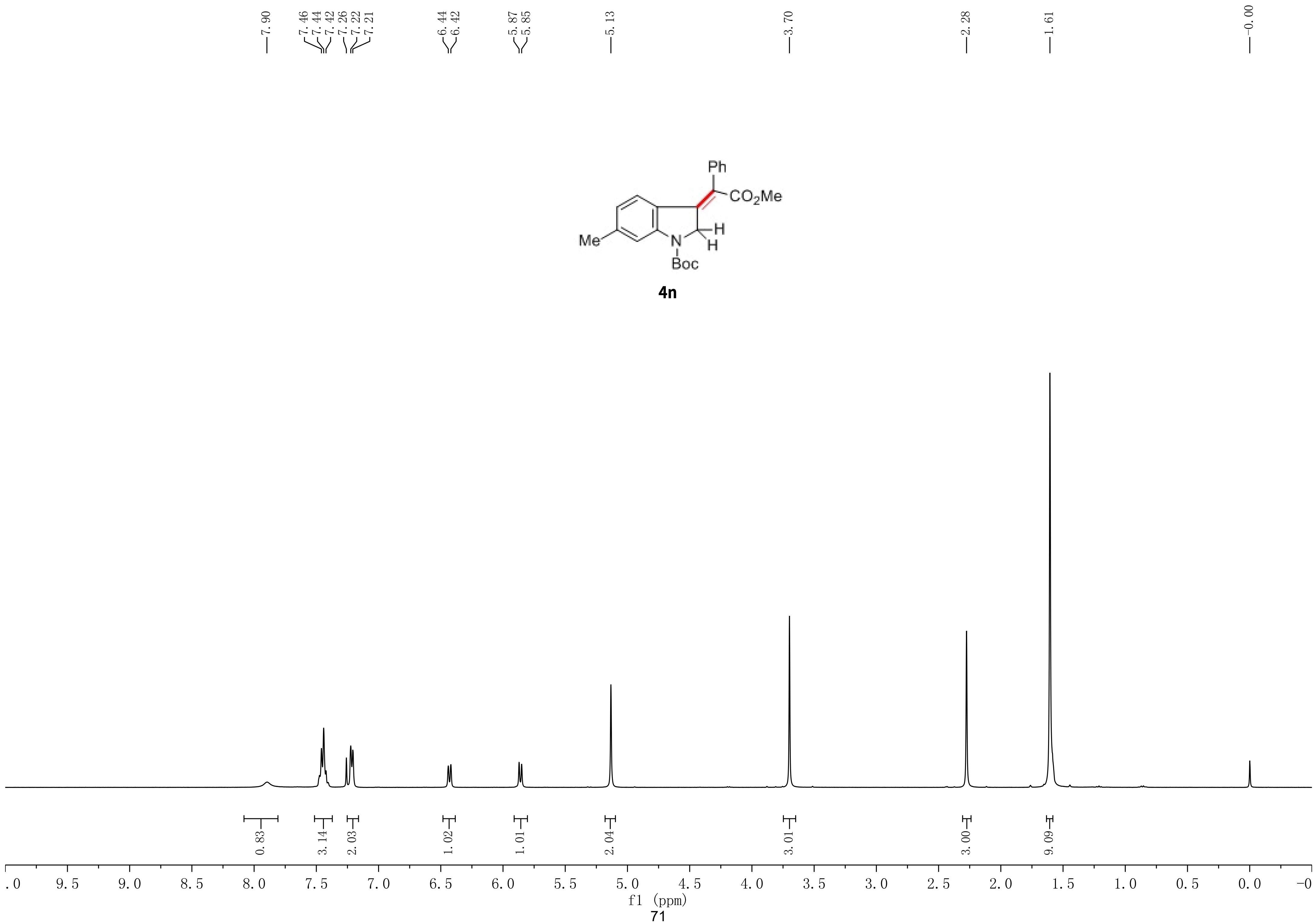
7.90
7.46 7.44 7.42 7.26 7.22 7.21
6.44 6.42
5.87 5.85
5.13
3.70
2.28
1.61
0.00
Ph
CO2Me
Me
N
Boc
H
H
4n
0.83
3.14
2.03
1.02
1.01
2.04
3.01
3.00
9.09
9.5 9.0 8.5 8.0 7.5 7.0 6.5 6.0 5.5 5.0 4.5 4.0 3.5 3.0 2.5 2.0 1.5 1.0 0.5 0.0
f1 (ppm)

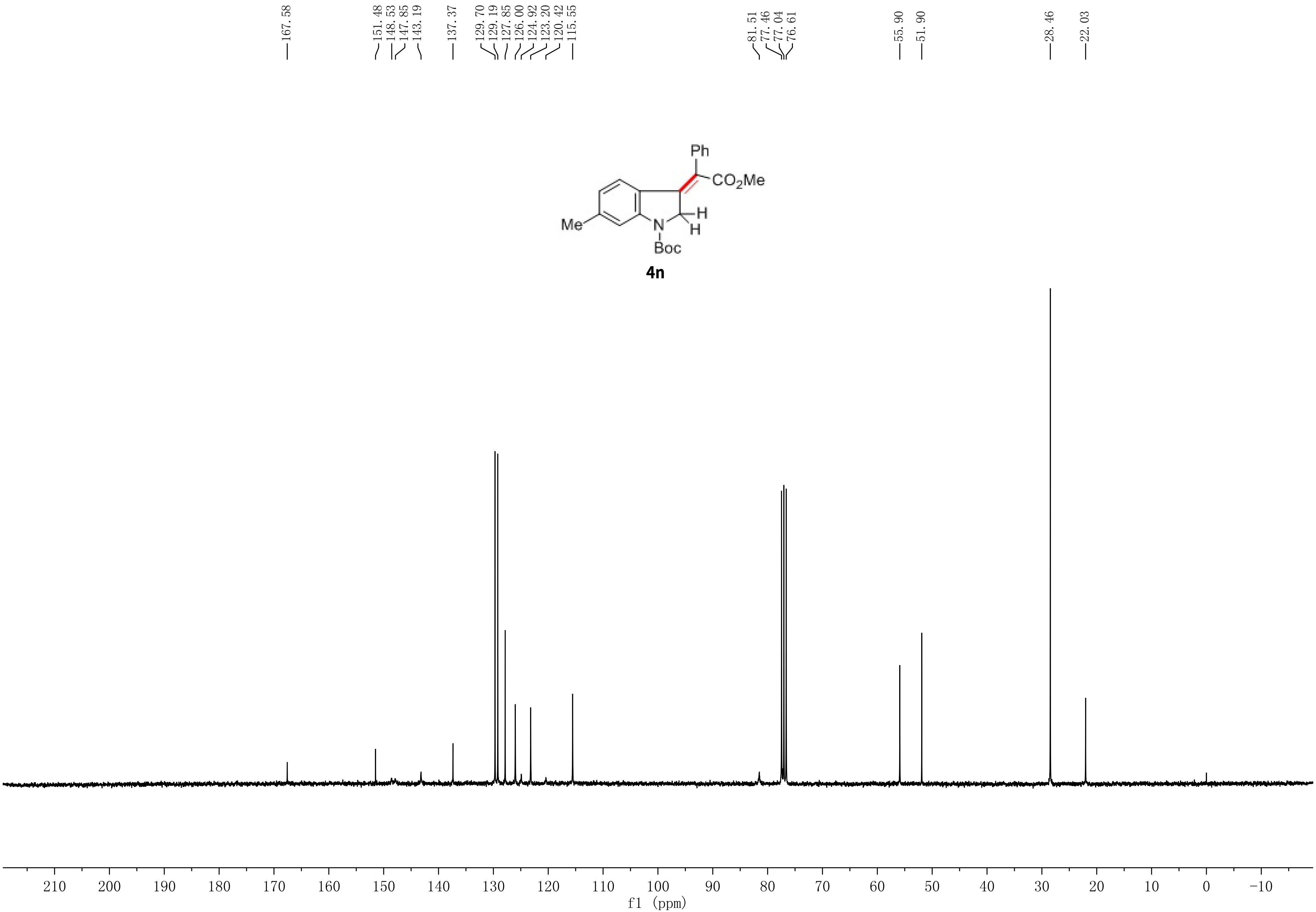
167.58
151.48
148.53
147.85
143.19
137.37
129.70
129.19
127.85
126.00
124.92
123.20
120.42
115.55
81.51
77.46
77.04
76.61
55.90
51.90
28.46
22.03
Ph
CO2Me
Me
N
H
H
Boc
4n
210 200 190 180 170 160 150 140 130 120 110 100 90 80 70 60 50 40 30 20 10 0 -10
f1 (ppm)

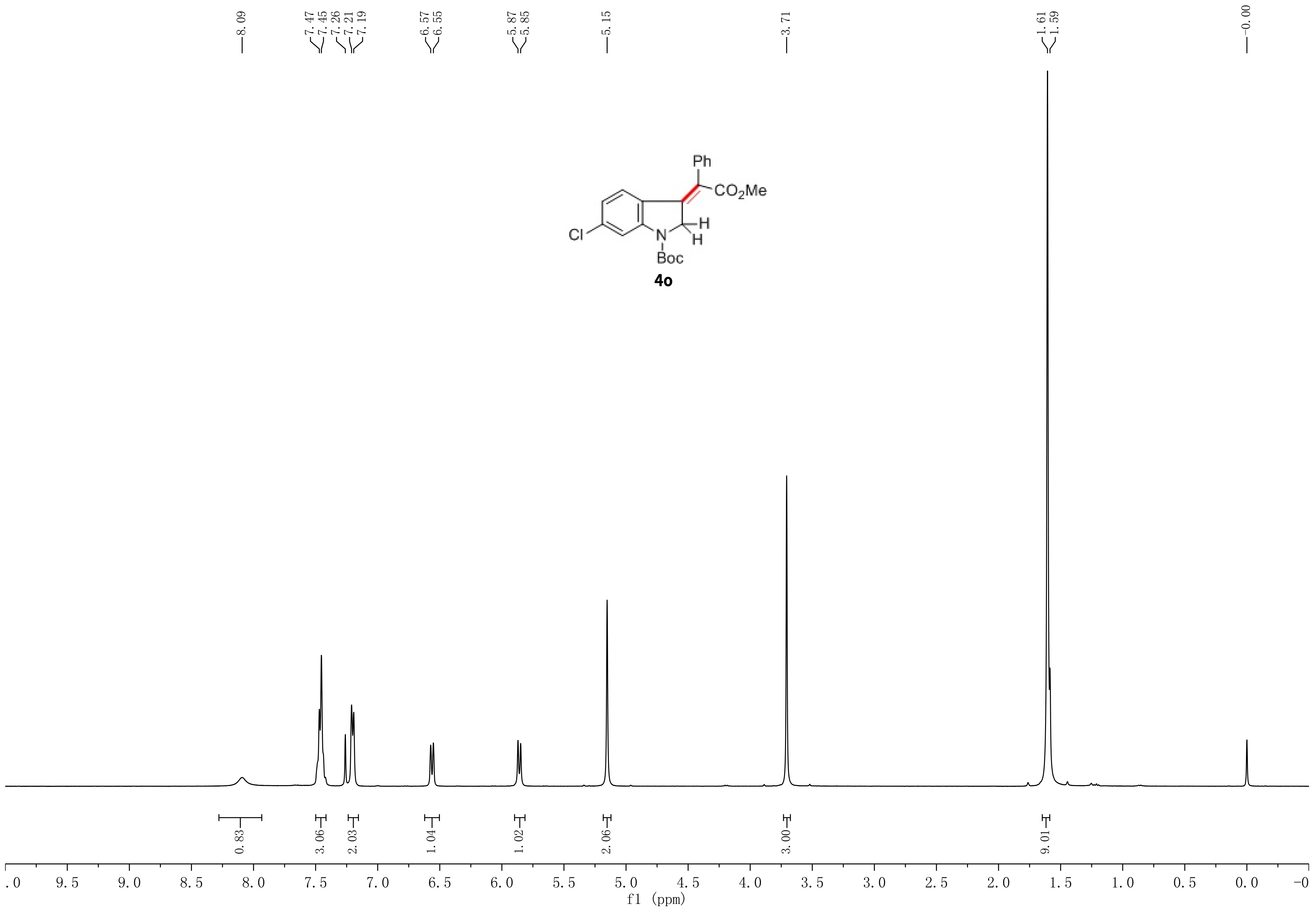
8.09
7.47
7.45
7.26
7.21
7.19
6.57
6.55
5.87
5.85
5.15
3.71
1.61
1.59
-0.00
Ph
CO2Me
Cl
N
H
H
Boc
4o
0.83
3.06
2.03
1.04
1.02
2.06
3.00
9.01
f1 (ppm)

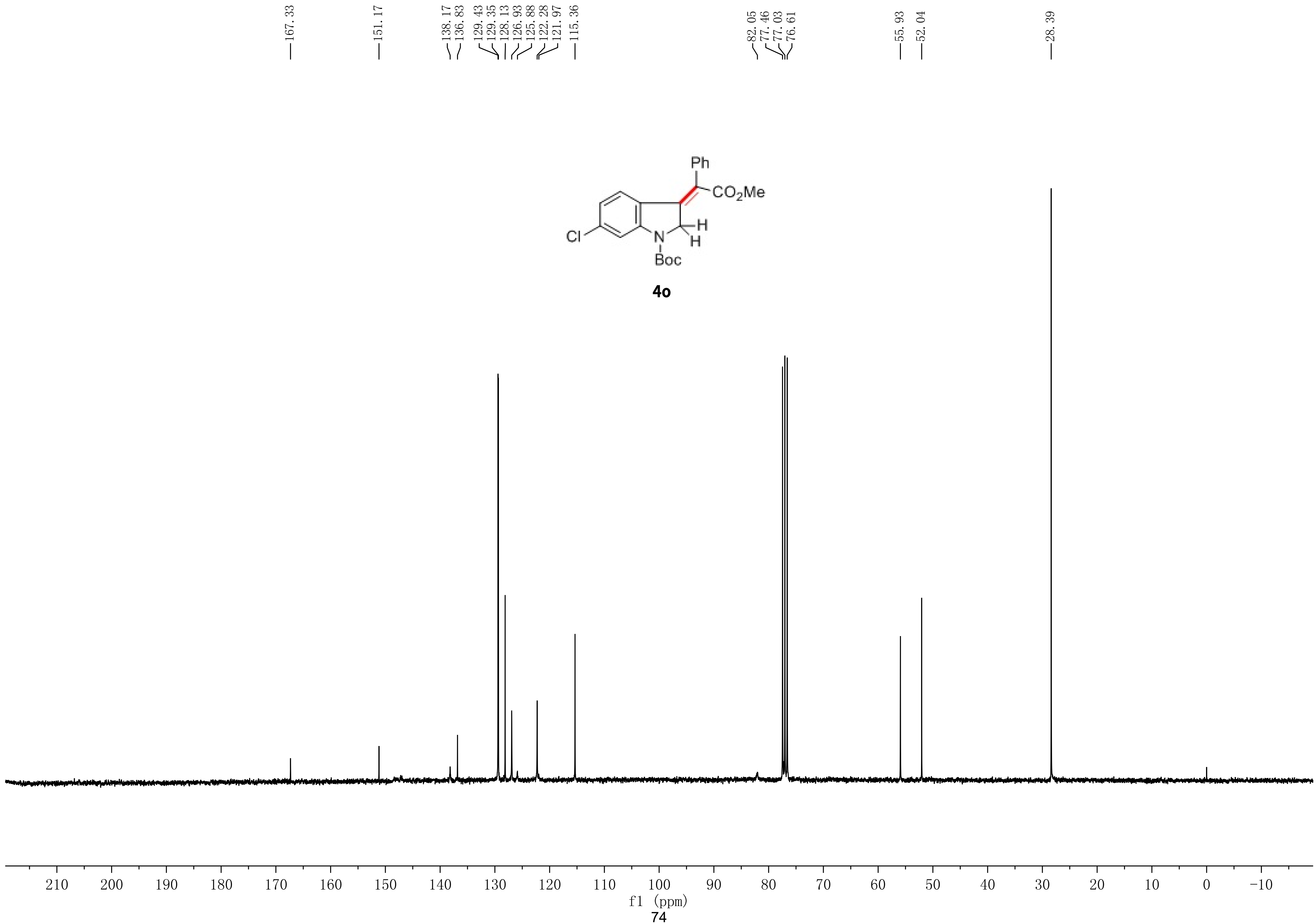
167.33
151.17
138.17
136.83
129.43
129.35
128.13
126.93
125.88
122.28
121.97
115.36
82.05
77.46
77.03
76.61
55.93
52.04
28.39
Ph
CO2Me
H
H
Cl
N
Boc
4o
210
200
190
180
170
160
150
140
130
120
110
100
90
80
70
60
50
40
30
20
10
0
−10
f1 (ppm)

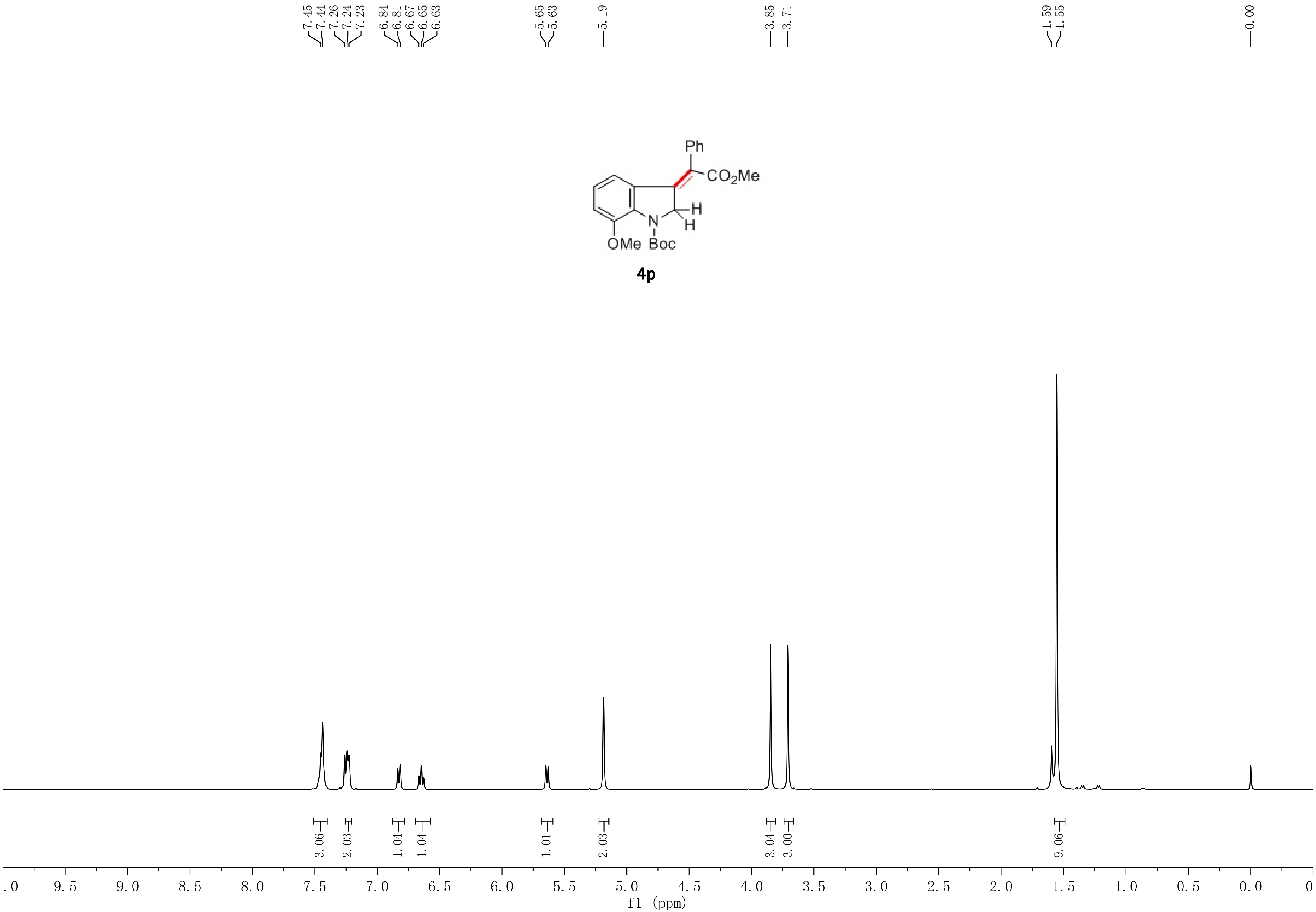
7.45
7.44
7.26
7.24
7.23
6.84
6.81
6.67
6.65
6.63
5.65
5.63
5.19
3.85
3.71
1.59
1.55
0.00
Ph
CO2Me
H
H
N
OMe
Boc
4p
3.06
2.03
1.04
1.04
1.01
2.03
3.04
3.00
9.06
f1 (ppm)

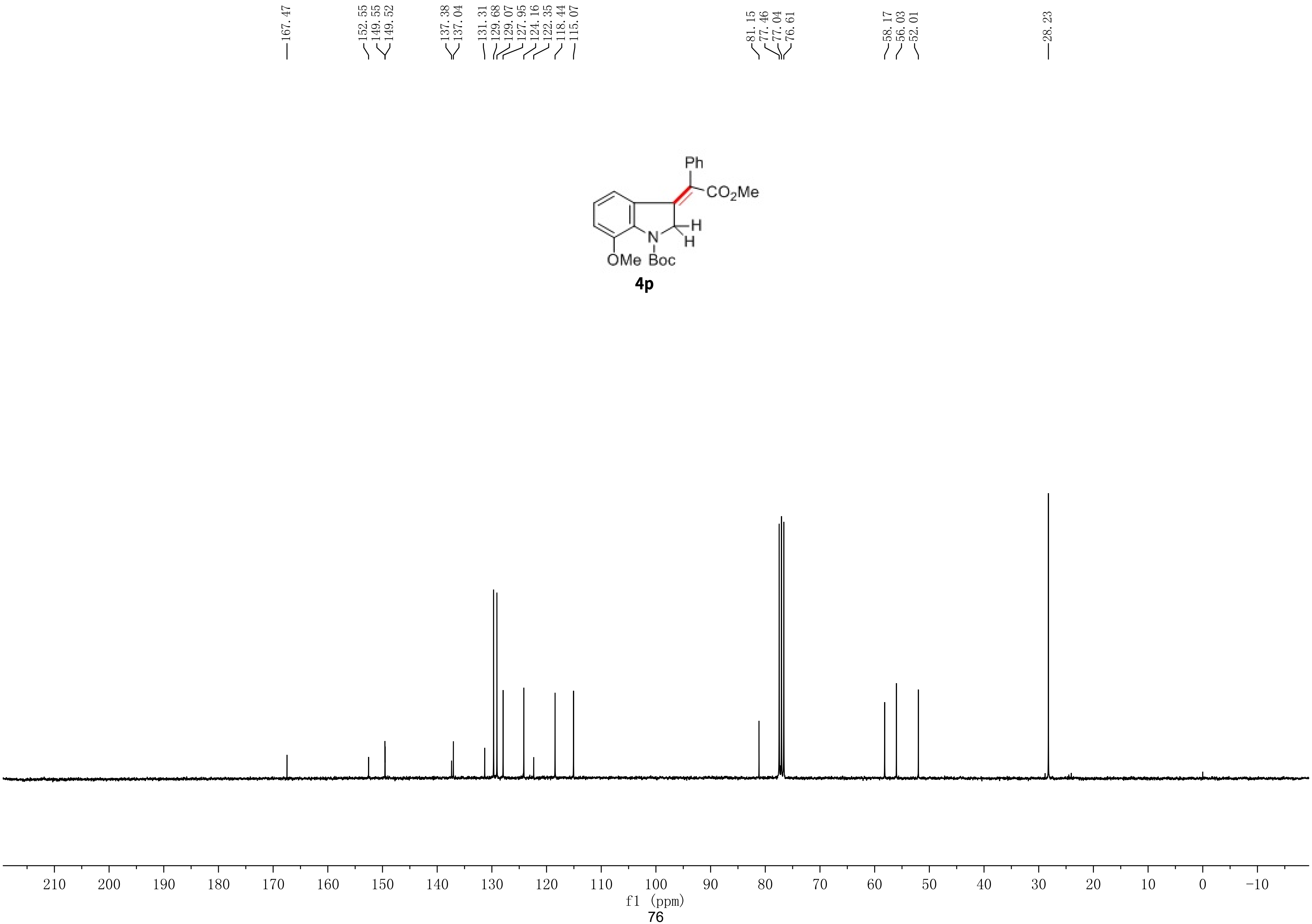

167.47
152.55
149.55
149.52
137.38
137.04
131.31
129.68
129.07
127.95
124.16
122.35
118.44
115.07
81.15
77.46
77.04
76.61
58.17
56.03
52.01
28.23
Ph
CO_2Me
H
H
N
OMe
Boc
4p
210 200 190 180 170 160 150 140 130 120 110 100 90 80 70 60 50 40 30 20 10 0 -10
f1 (ppm)

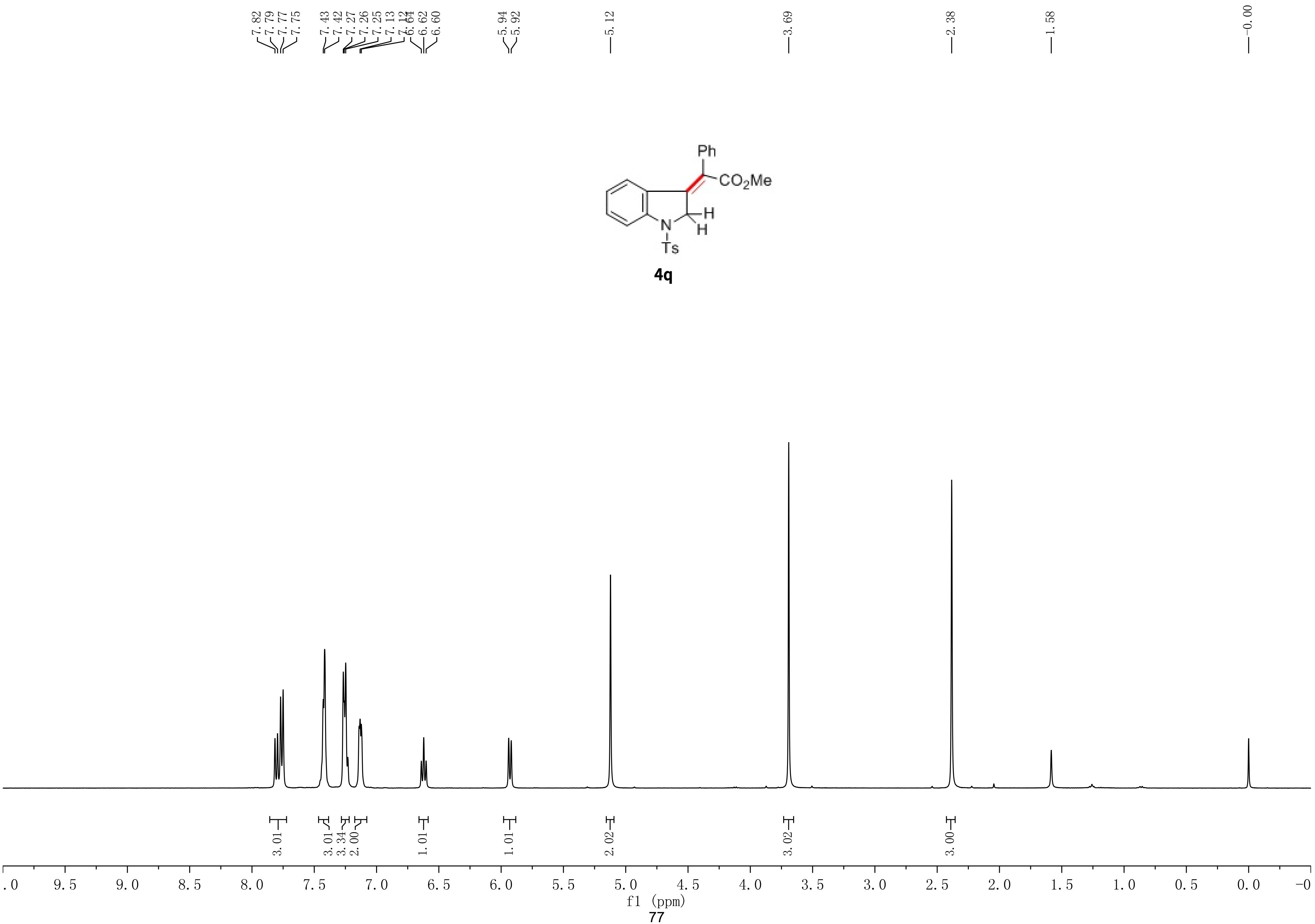
7.82
7.79
7.77
7.75
7.43
7.42
7.27
7.26
7.25
7.13
7.12
6.64
6.62
6.60
5.94
5.92
5.12
3.69
2.38
1.58
-0.00
Ph
CO2Me
H
H
N
Ts
4q
3.01
3.01
3.34
2.00
1.01
1.01
2.02
3.02
3.00
f1 (ppm)

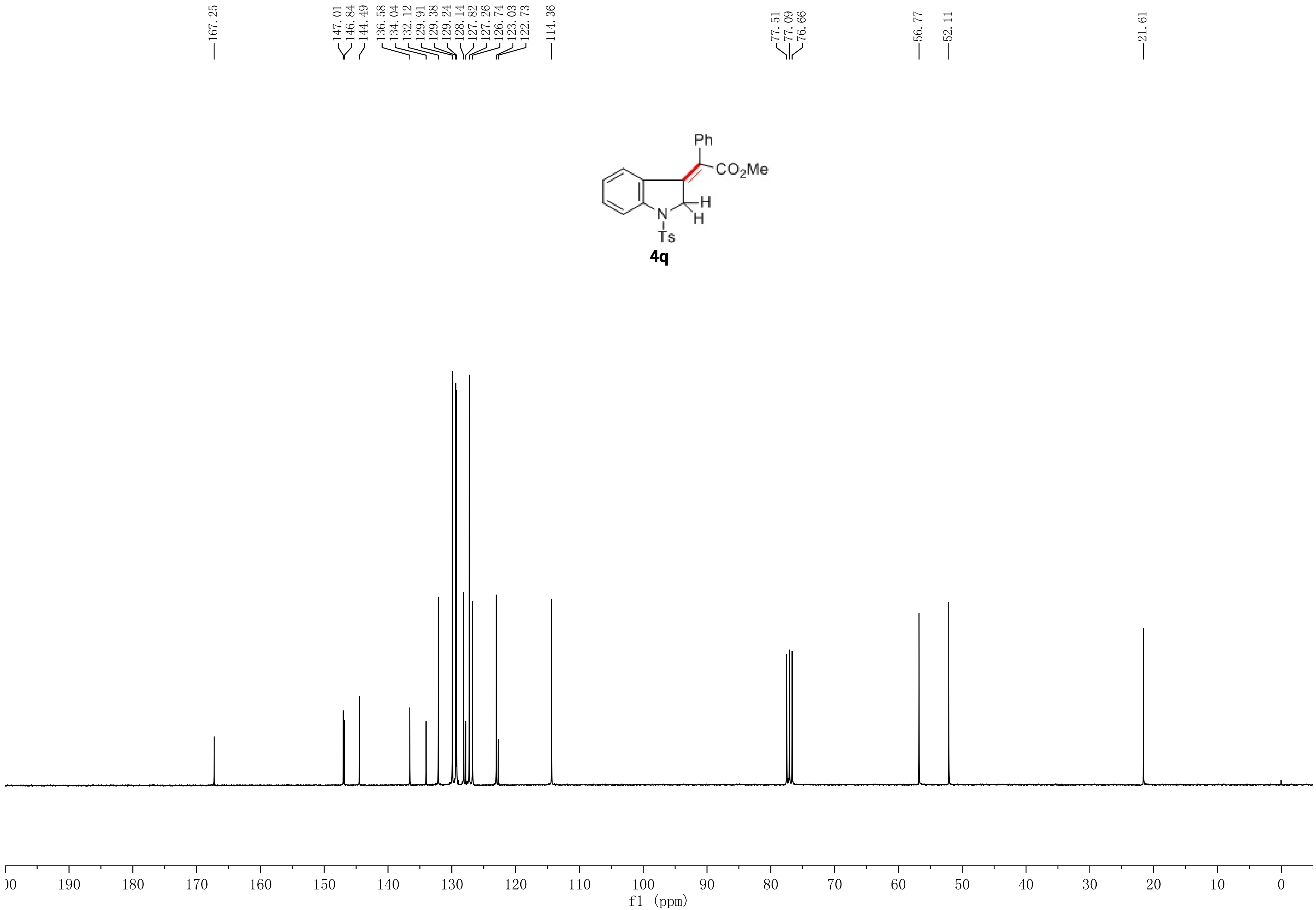
167.25
147.01
146.84
144.49
136.58
134.04
132.12
129.91
129.38
129.24
128.14
127.82
127.26
126.74
123.03
122.73
114.36
77.51
77.09
76.66
56.77
52.11
21.61
Ph
CO2Me
H
H
N
Ts
4q
f1 (ppm)

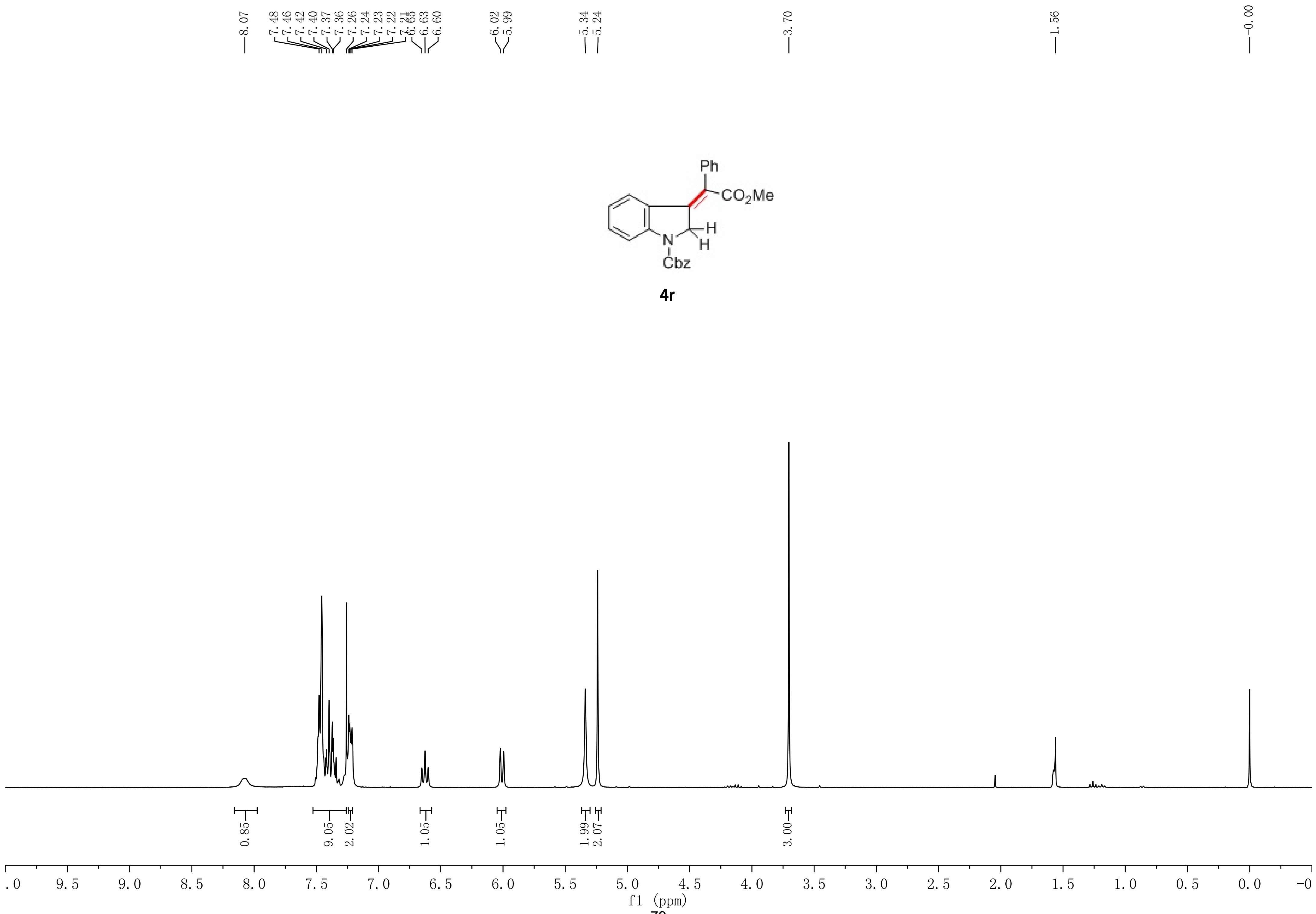
8.07
7.48
7.46
7.42
7.40
7.37
7.36
7.26
7.24
7.23
7.22
7.21
6.65
6.63
6.60
6.02
5.99
5.34
5.24
3.70
1.56
-0.00
Ph
CO2Me
H
H
N
Cbz
4r
0.85
9.05
2.02
1.05
1.05
1.99
2.07
3.00
f1 (ppm)

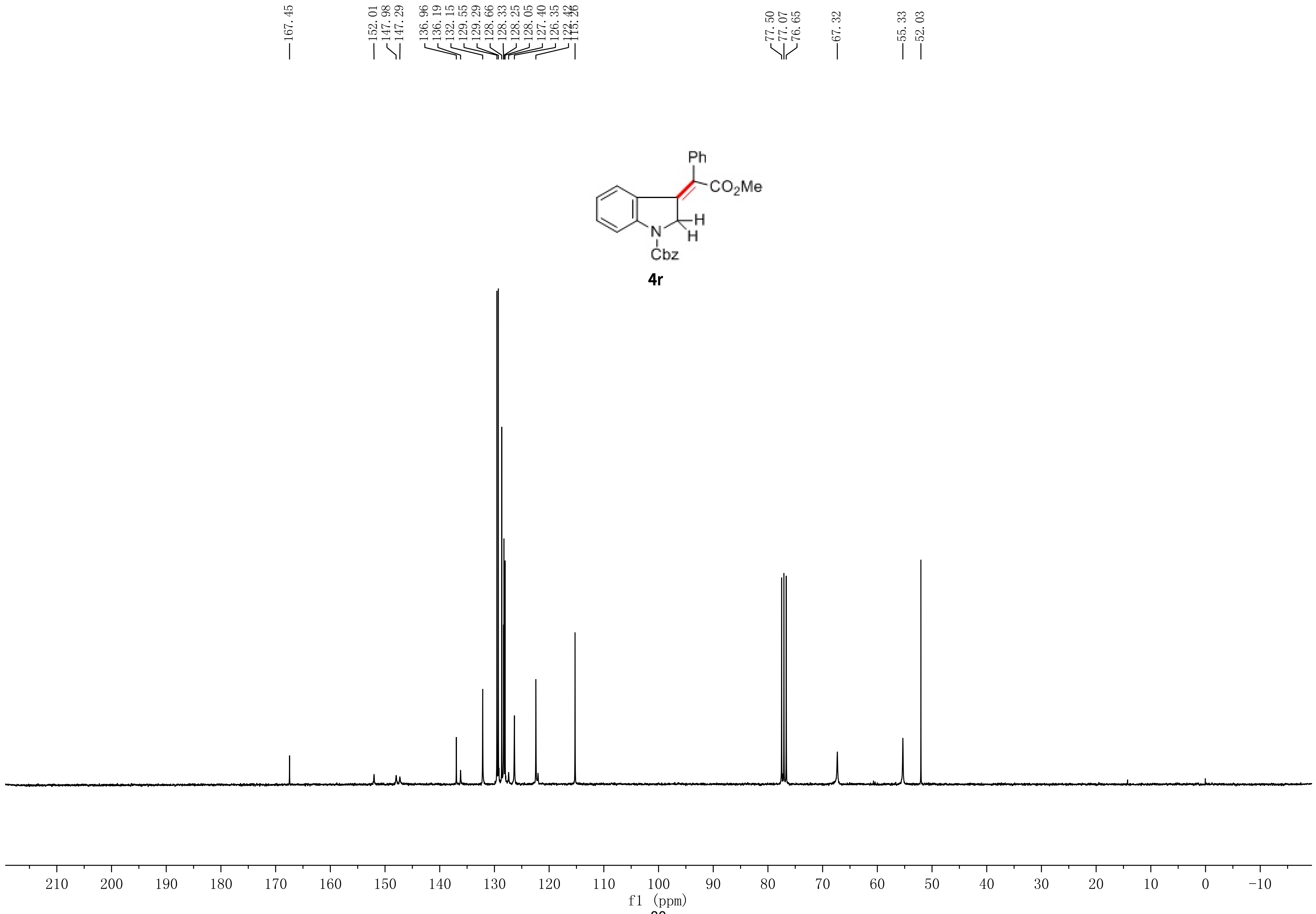
167.45
152.01
147.98
147.29
136.96
136.19
132.15
129.55
129.29
128.66
128.33
128.25
128.05
127.40
126.35
122.42
115.26
77.50
77.07
76.65
67.32
55.33
52.03
Ph
CO2Me
H
H
N
Cbz
4r
210 200 190 180 170 160 150 140 130 120 110 100 90 80 70 60 50 40 30 20 10 0 −10
f1 (ppm)

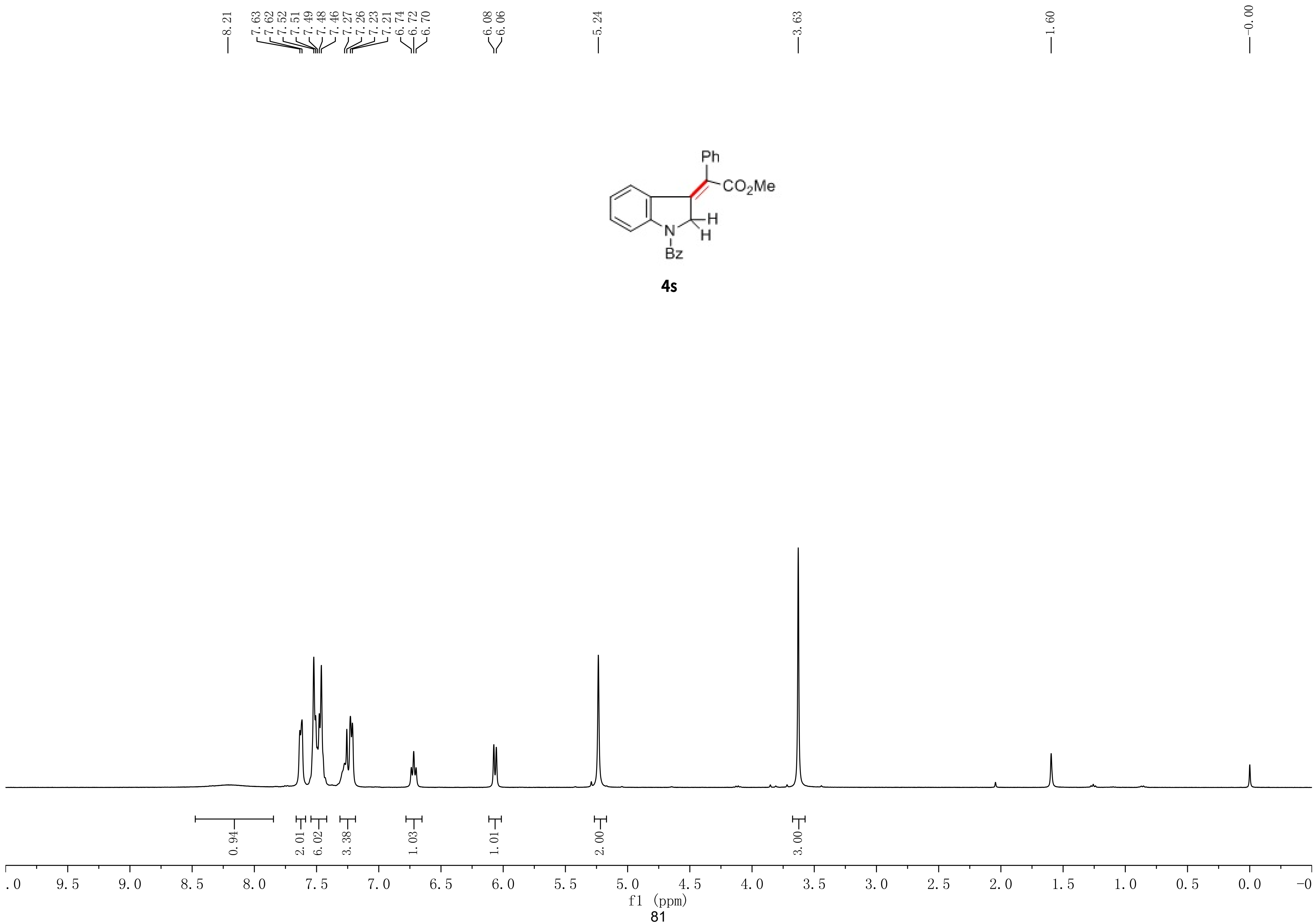
Ph
CO2Me
H
H
N
Bz

4s

8.21
7.63
7.62
7.52
7.51
7.49
7.48
7.46
7.27
7.26
7.23
7.21
6.74
6.72
6.70
6.08
6.06
5.24
3.63
1.60
-0.00
0.94
2.01
6.02
3.38
1.03
1.01
2.00
3.00
.0
9.5
9.0
8.5
8.0
7.5
7.0
6.5
6.0
5.5
5.0
4.5
4.0
3.5
3.0
2.5
2.0
1.5
1.0
0.5
0.0
-0
f1 (ppm)

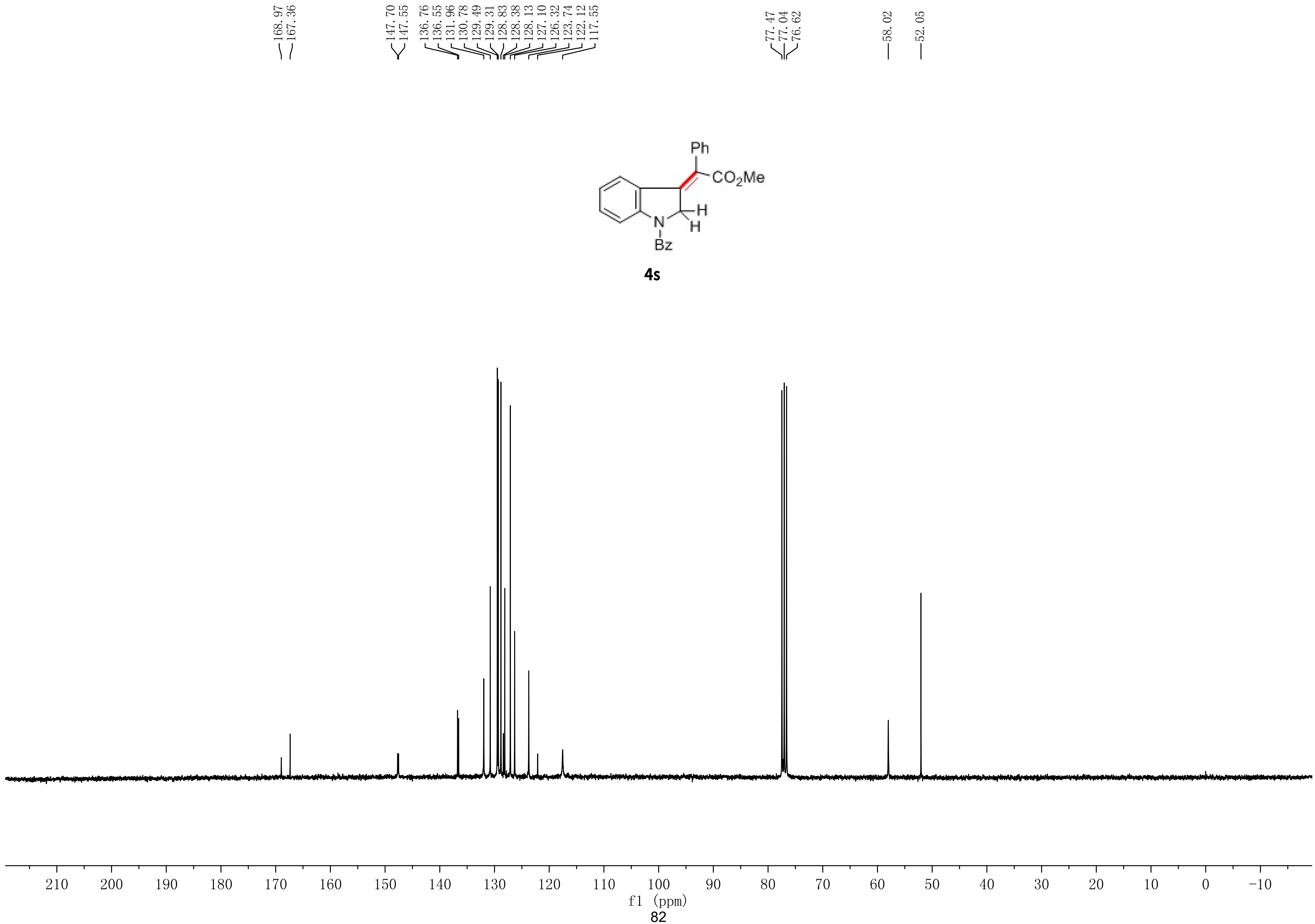
168.97
167.36
147.70
147.55
136.76
136.55
131.96
130.78
129.49
129.31
128.83
128.38
128.13
127.10
126.32
123.74
122.12
117.55
77.47
77.04
76.62
58.02
52.05
Ph
CO2Me
H
H
N
Bz
4s
210
200
190
180
170
160
150
140
130
120
110
100
90
80
70
60
50
40
30
20
10
0
-10
f1 (ppm)

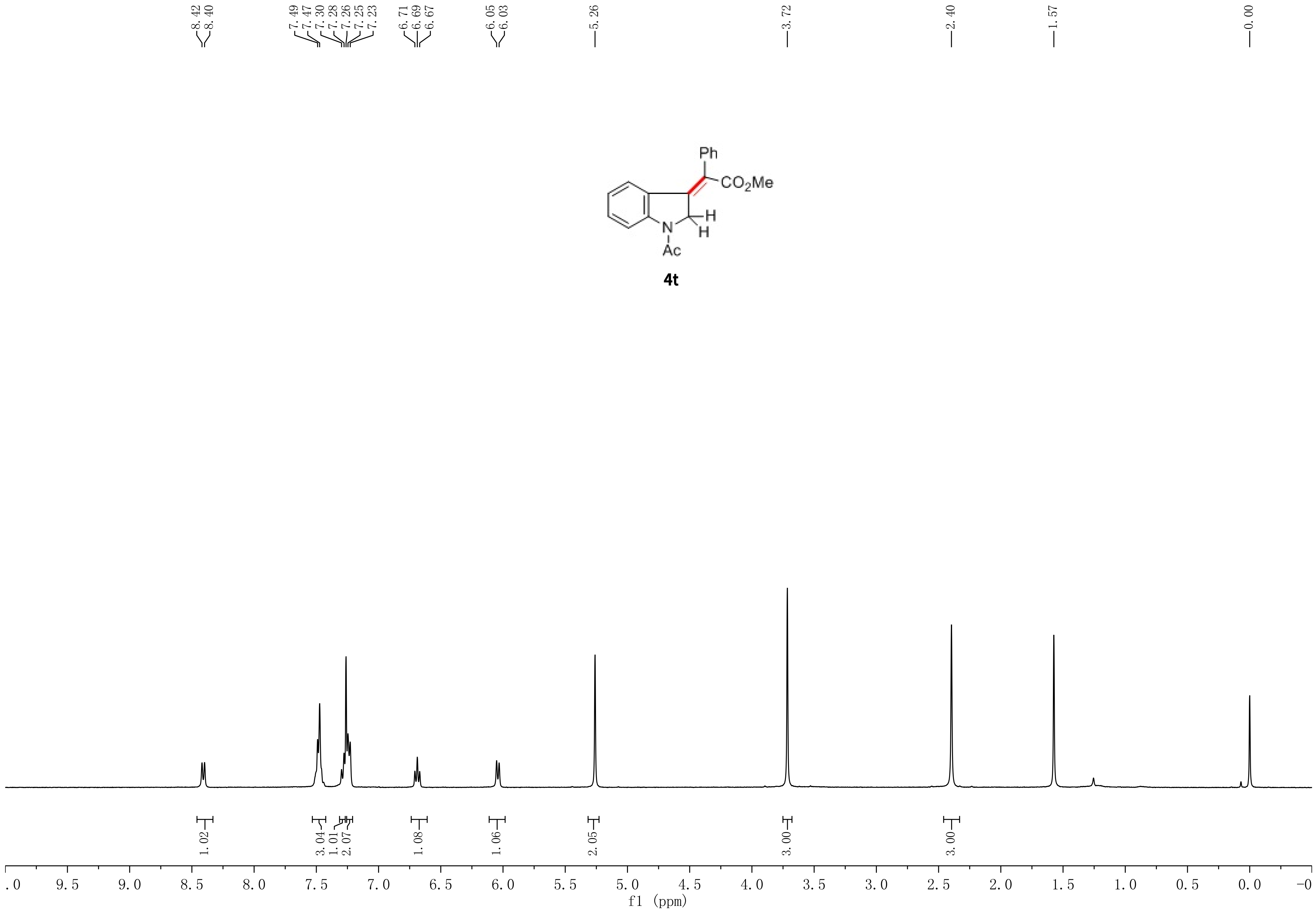
8.42
8.40
7.49
7.47
7.30
7.28
7.26
7.25
7.23
6.71
6.69
6.67
6.05
6.03
5.26
3.72
2.40
1.57
0.00
Ph
CO_2Me
H
H
N
Ac
4t
1.02
3.04
1.01
2.07
1.08
1.06
2.05
3.00
3.00
f1 (ppm)

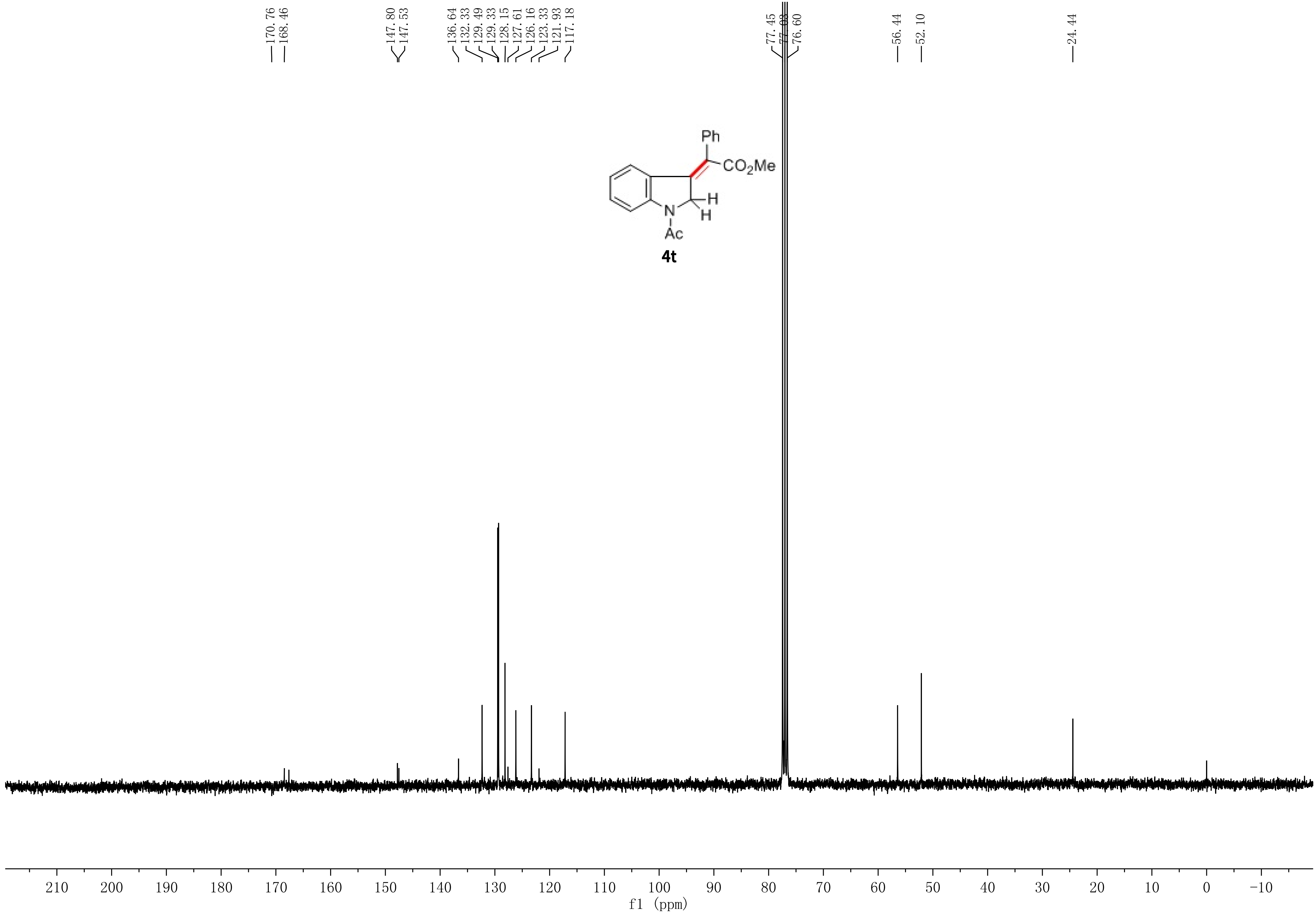
170.76
168.46
147.80
147.53
136.64
132.33
129.49
129.33
128.15
127.61
126.16
123.33
121.93
117.18
77.45
77.03
76.60
56.44
52.10
24.44
Ph
CO2Me
H
H
N
Ac
4t
210 200 190 180 170 160 150 140 130 120 110 100 90 80 70 60 50 40 30 20 10 0 -10
f1 (ppm)

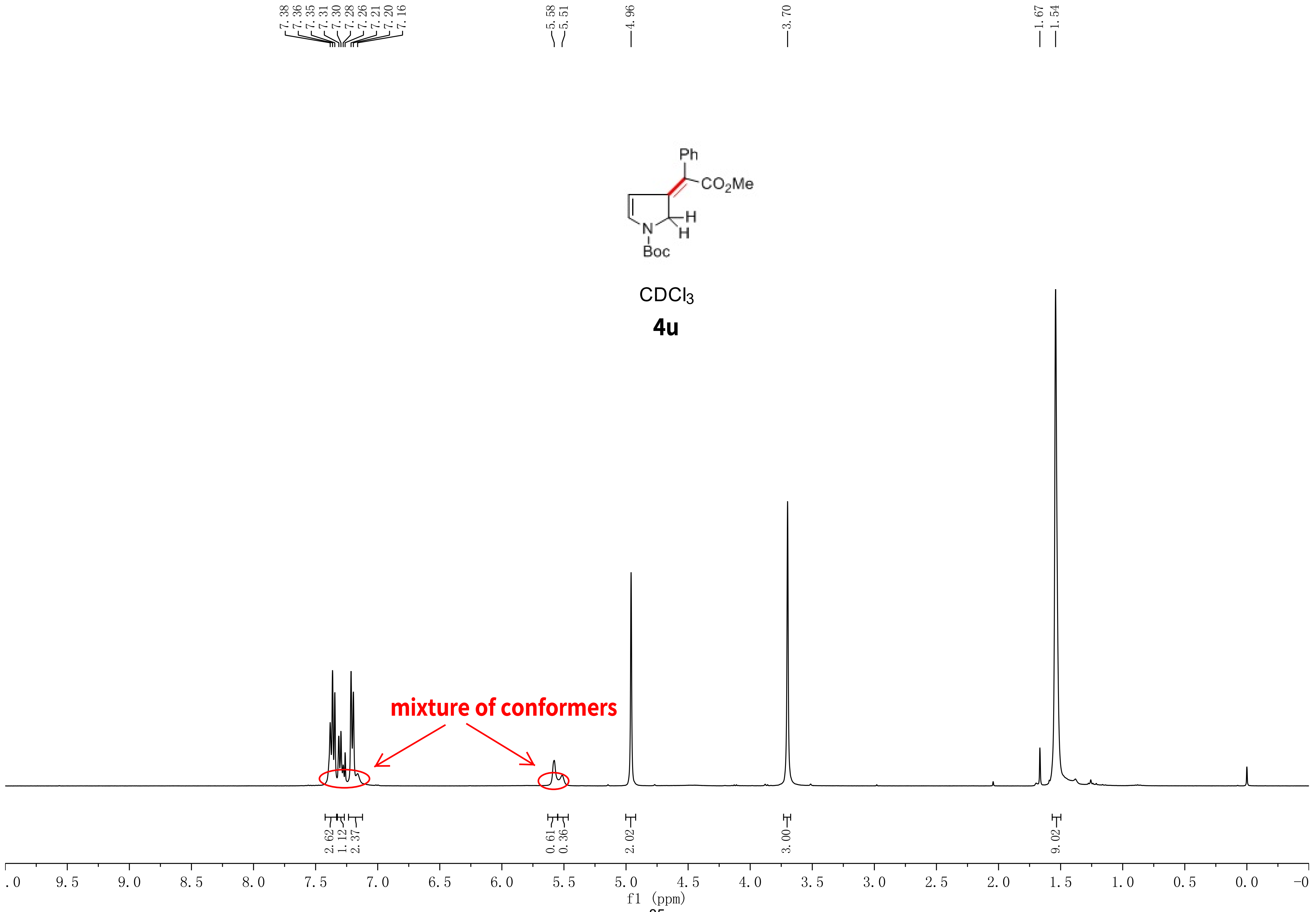
7.38
7.36
7.35
7.31
7.30
7.28
7.26
7.21
7.20
7.16
5.58
5.51
4.96
3.70
1.67
1.54
Ph
CO2Me
H
H
N
Boc
CDCl3
4u
mixture of conformers
2.62
1.12
2.37
0.61
0.36
2.02
3.00
9.02
.0
9.5
9.0
8.5
8.0
7.5
7.0
6.5
6.0
5.5
5.0
4.5
4.0
3.5
3.0
2.5
2.0
1.5
1.0
0.5
0.0
-0
f1 (ppm)

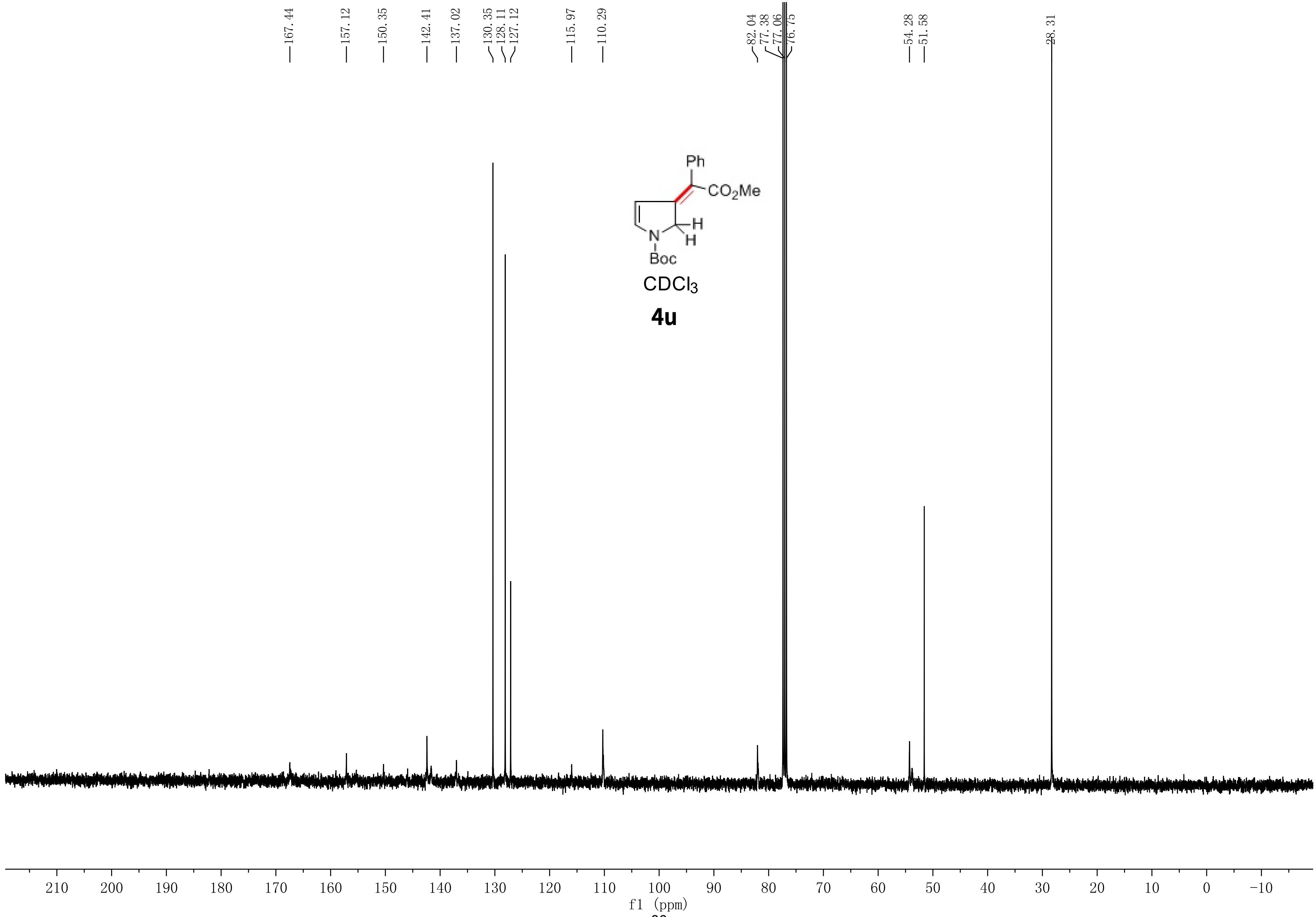
167.44
157.12
150.35
142.41
137.02
130.35
128.11
127.12
115.97
110.29
82.04
77.38
77.06
76.75
54.28
51.58
28.31
Ph
CO2Me
H
H
N
Boc
CDCl3
4u
210 200 190 180 170 160 150 140 130 120 110 100 90 80 70 60 50 40 30 20 10 0 -10
f1 (ppm)

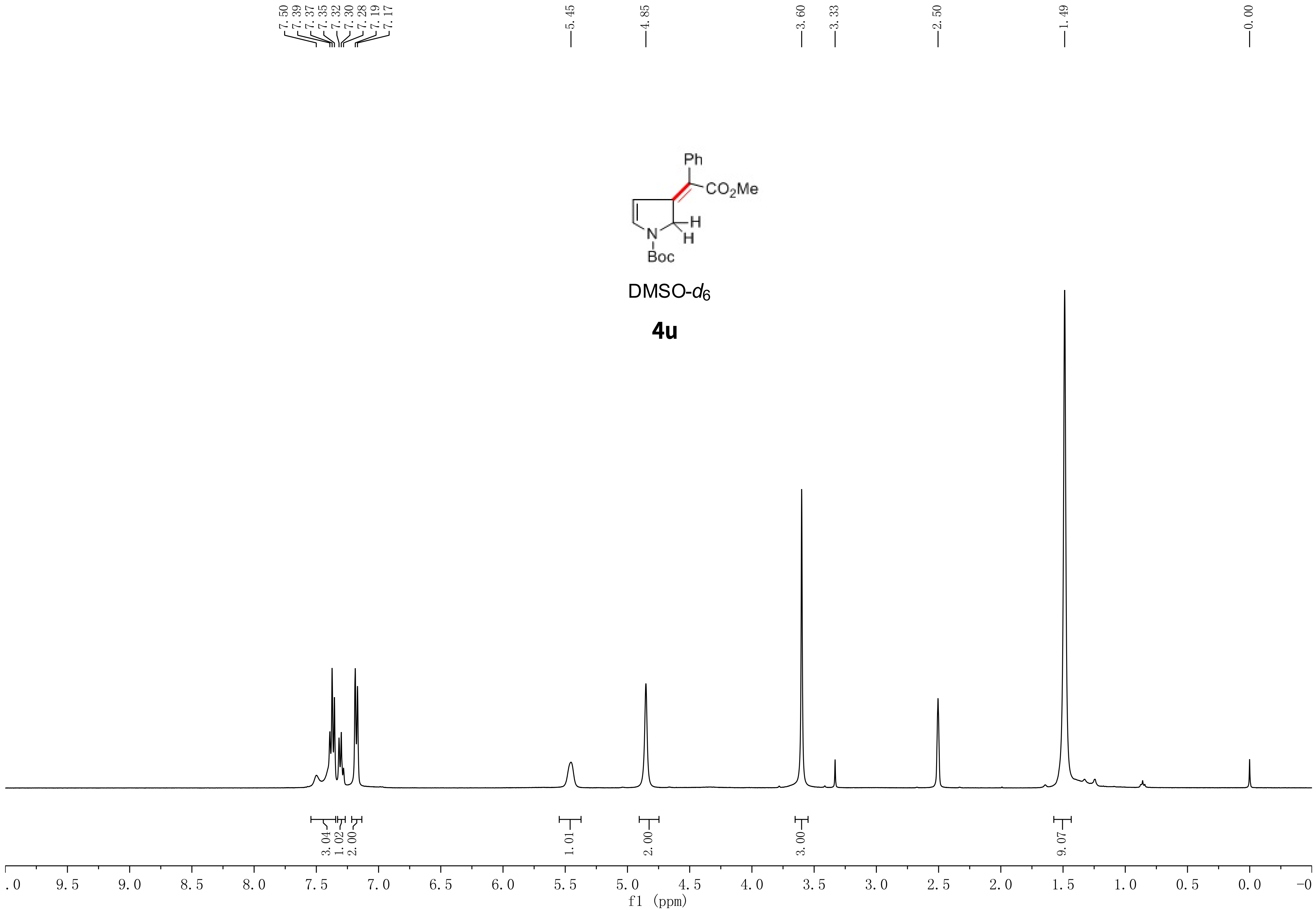
7.50
7.39
7.37
7.35
7.32
7.30
7.28
7.19
7.17
5.45
4.85
3.60
3.33
2.50
1.49
0.00
Ph
CO_2Me
H
H
N
Boc
DMSO-d_6
4u
3.04
1.02
2.00
1.01
2.00
3.00
9.07
f1 (ppm)

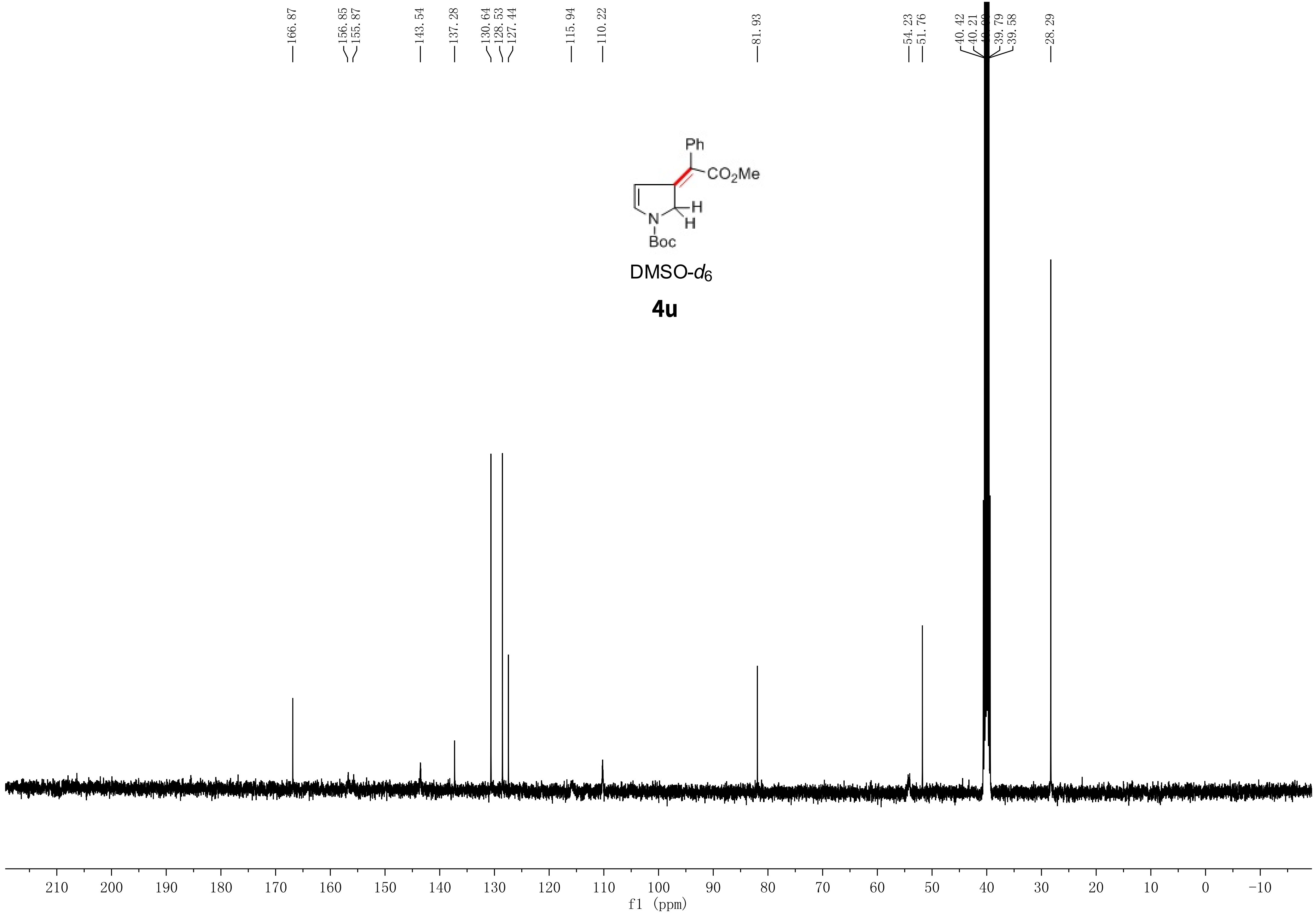
166.87
156.85
155.87
143.54
137.28
130.64
128.53
127.44
115.94
110.22
81.93
54.23
51.76
40.42
40.21
39.79
39.58
28.29
Ph
CO2Me
N
H
H
Boc
DMSO-d6
4u
210 200 190 180 170 160 150 140 130 120 110 100 90 80 70 60 50 40 30 20 10 0 -10
f1 (ppm)

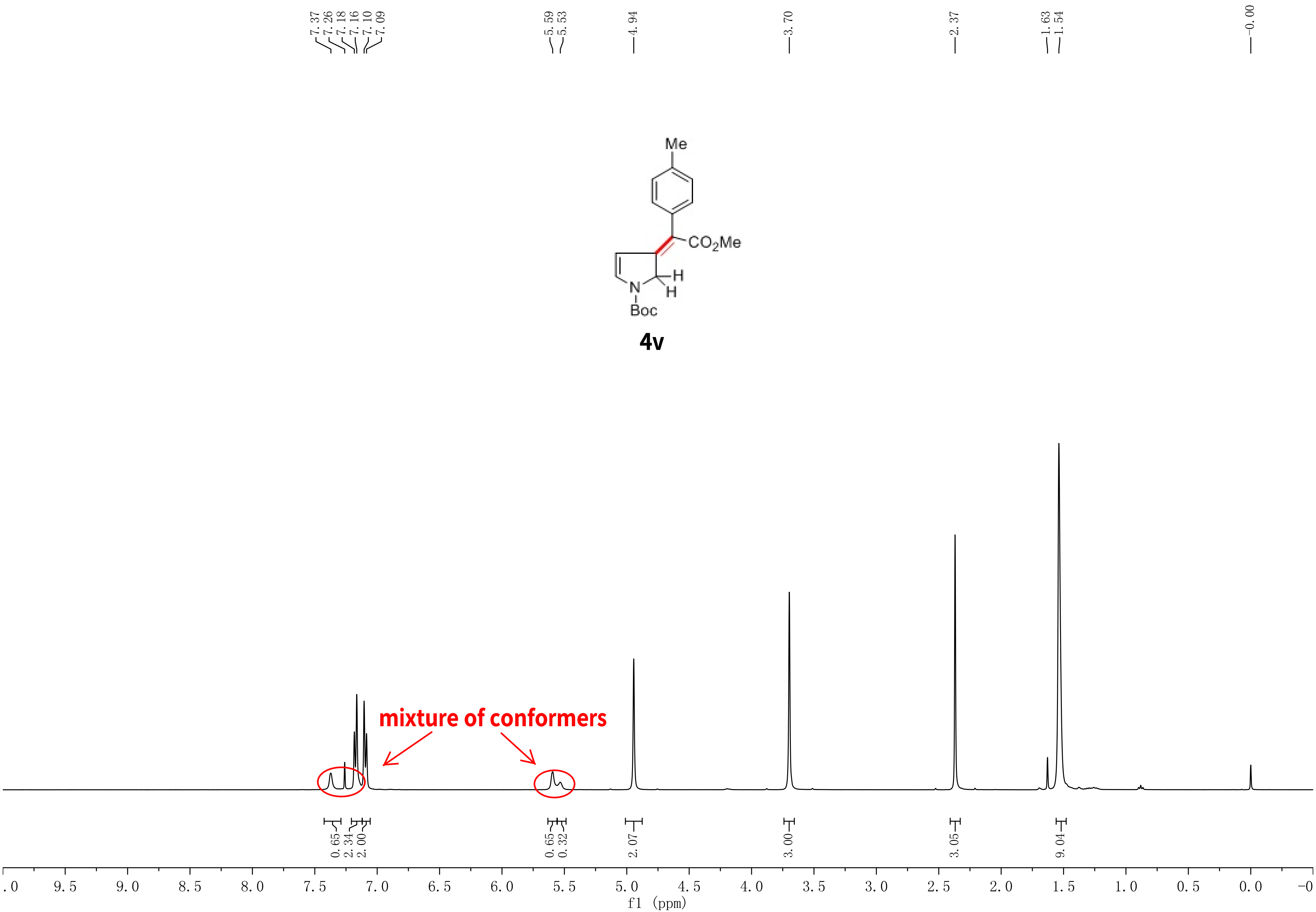
7.37
7.26
7.18
7.16
7.10
7.09
5.59
5.53
4.94
3.70
2.37
1.63
1.54
-0.00
Me
CO2Me
N
Boc
H
H
4v
mixture of conformers
0.65
2.34
2.00
0.65
0.32
2.07
3.00
3.05
9.04
f1 (ppm)

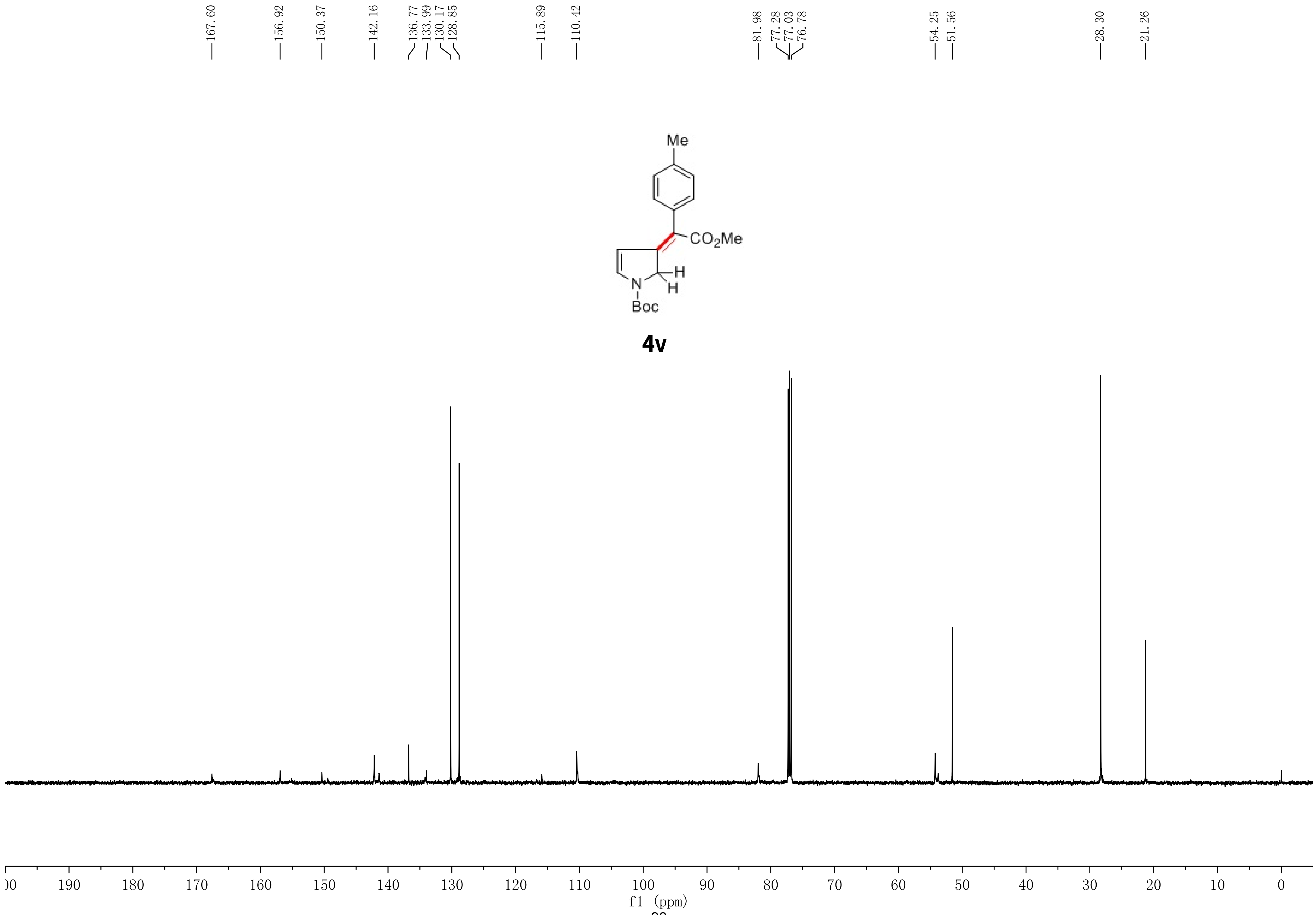

167.60
156.92
150.37
142.16
136.77
133.99
130.17
128.85
115.89
110.42
81.98
77.28
77.03
76.78
54.25
51.56
28.30
21.26
Me
CO2Me
H
H
N
Boc
4v
190
180
170
160
150
140
130
120
110
100
90
80
70
60
50
40
30
20
10
0
f1 (ppm)

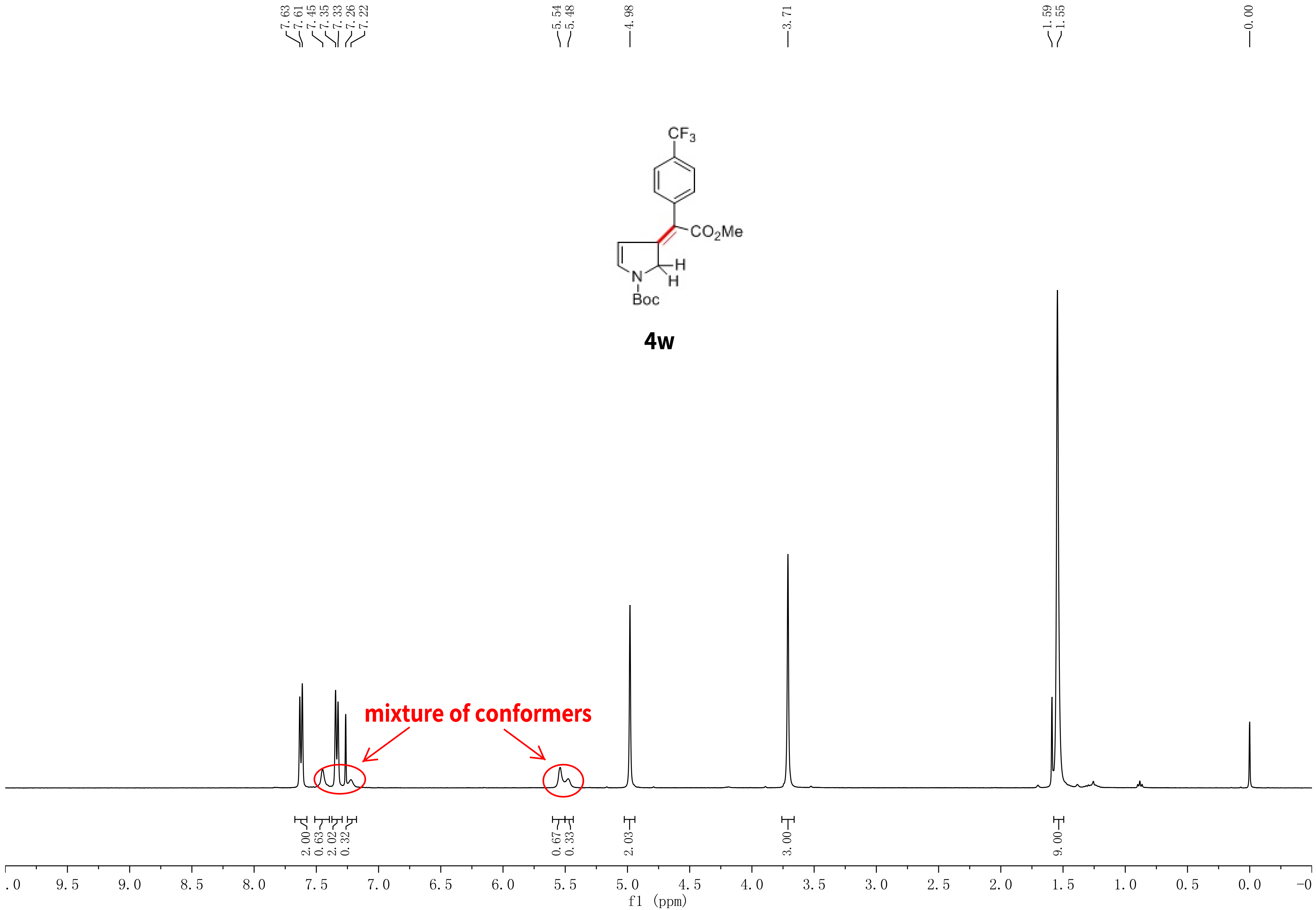
7.63
7.61
7.45
7.35
7.33
7.26
7.22
5.54
5.48
4.98
3.71
1.59
1.55
0.00
CF3
CO2Me
H
H
N
Boc
4w
mixture of conformers
2.00
0.63
2.02
0.32
0.67
0.33
2.03
3.00
9.00
f1 (ppm)

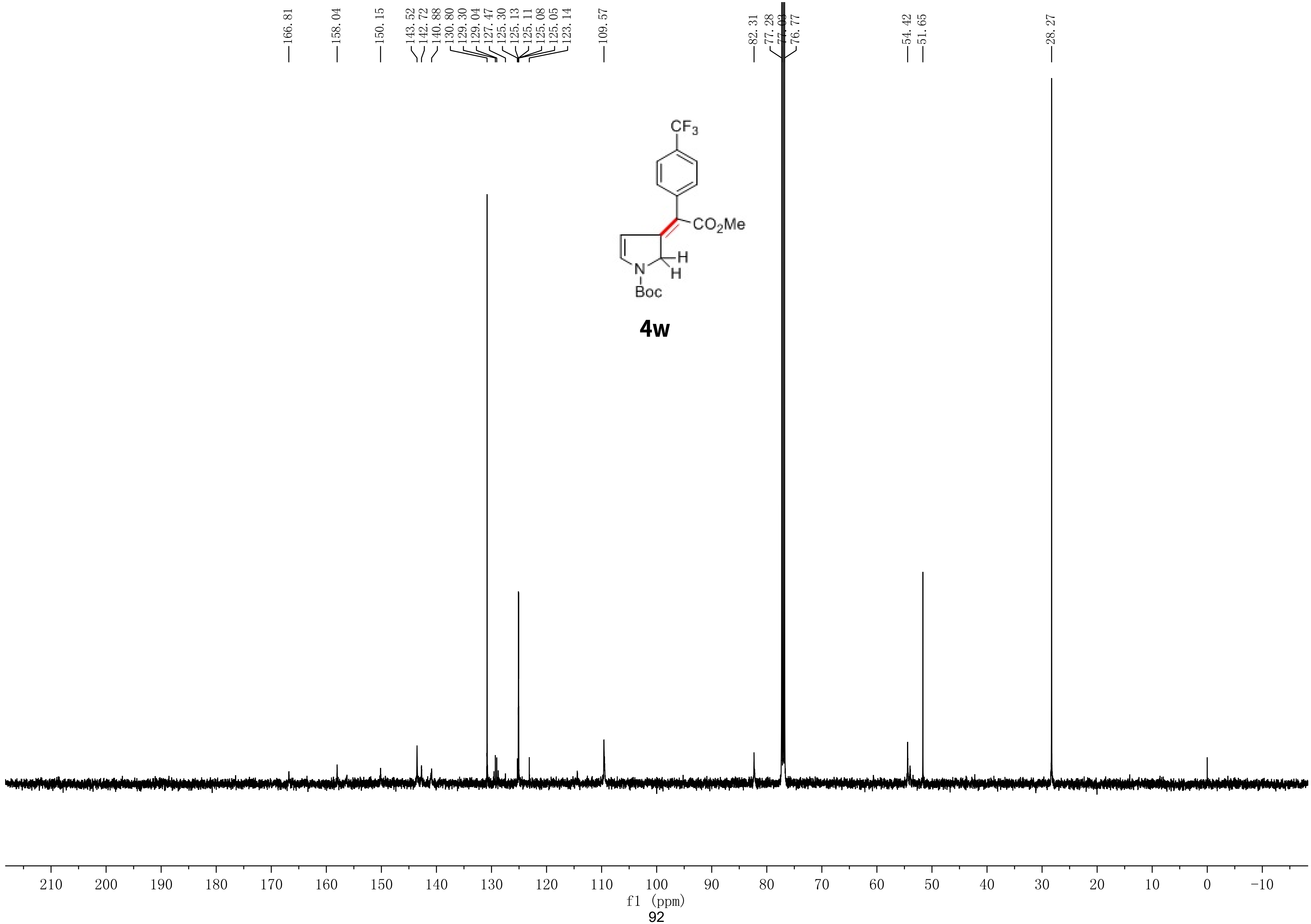
166.81
158.04
150.15
143.52
142.72
140.88
130.80
129.30
129.04
127.47
125.30
125.13
125.11
125.08
125.05
123.14
109.57
82.31
77.28
76.77
54.42
51.65
28.27
CF3
CO2Me
H
H
N
Boc
4w
210 200 190 180 170 160 150 140 130 120 110 100 90 80 70 60 50 40 30 20 10 0 −10
f1 (ppm)

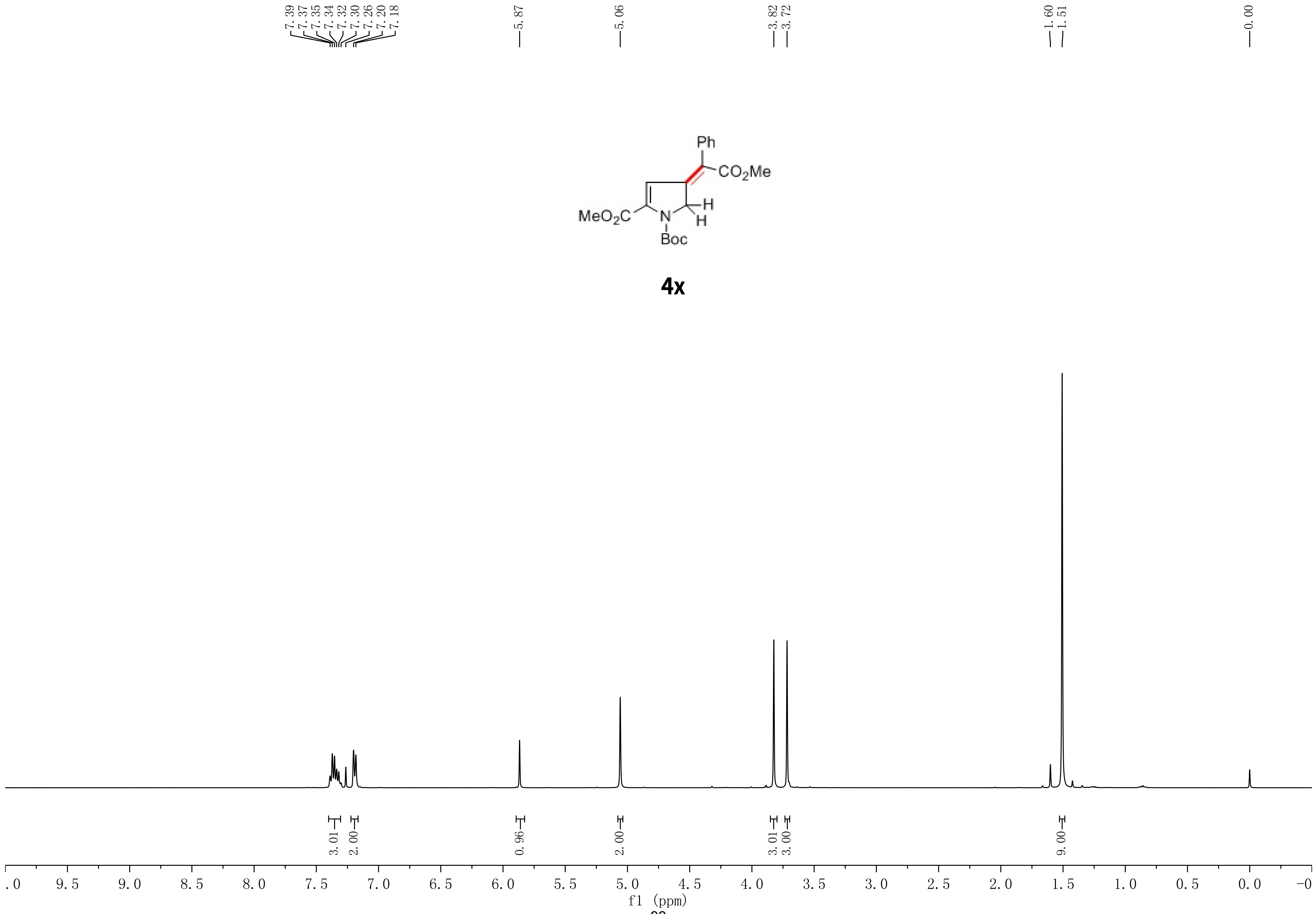
7.39
7.37
7.35
7.34
7.32
7.30
7.26
7.20
7.18
5.87
5.06
3.82
3.72
1.60
1.51
0.00
Ph
CO_2Me
MeO_2C
N
H
H
Boc
4x
3.01
2.00
0.96
2.00
3.01
3.00
9.00
f1 (ppm)

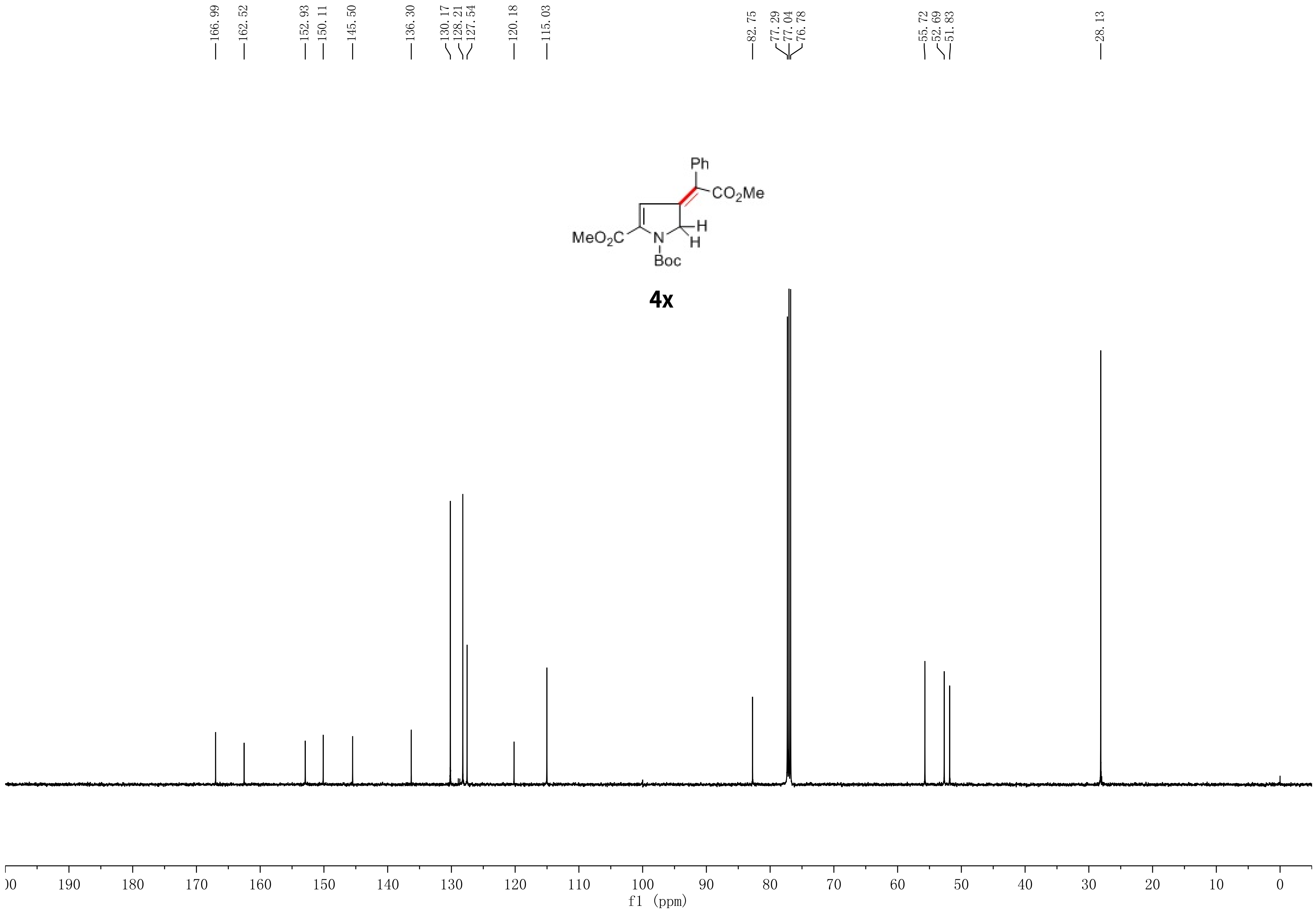
166.99
162.52
152.93
150.11
145.50
136.30
130.17
128.21
127.54
120.18
115.03
82.75
77.29
77.04
76.78
55.72
52.69
51.83
28.13
Ph
CO2Me
MeO2C
N
Boc
H
H
4x
190
180
170
160
150
140
130
120
110
100
90
80
70
60
50
40
30
20
10
0
f1 (ppm)

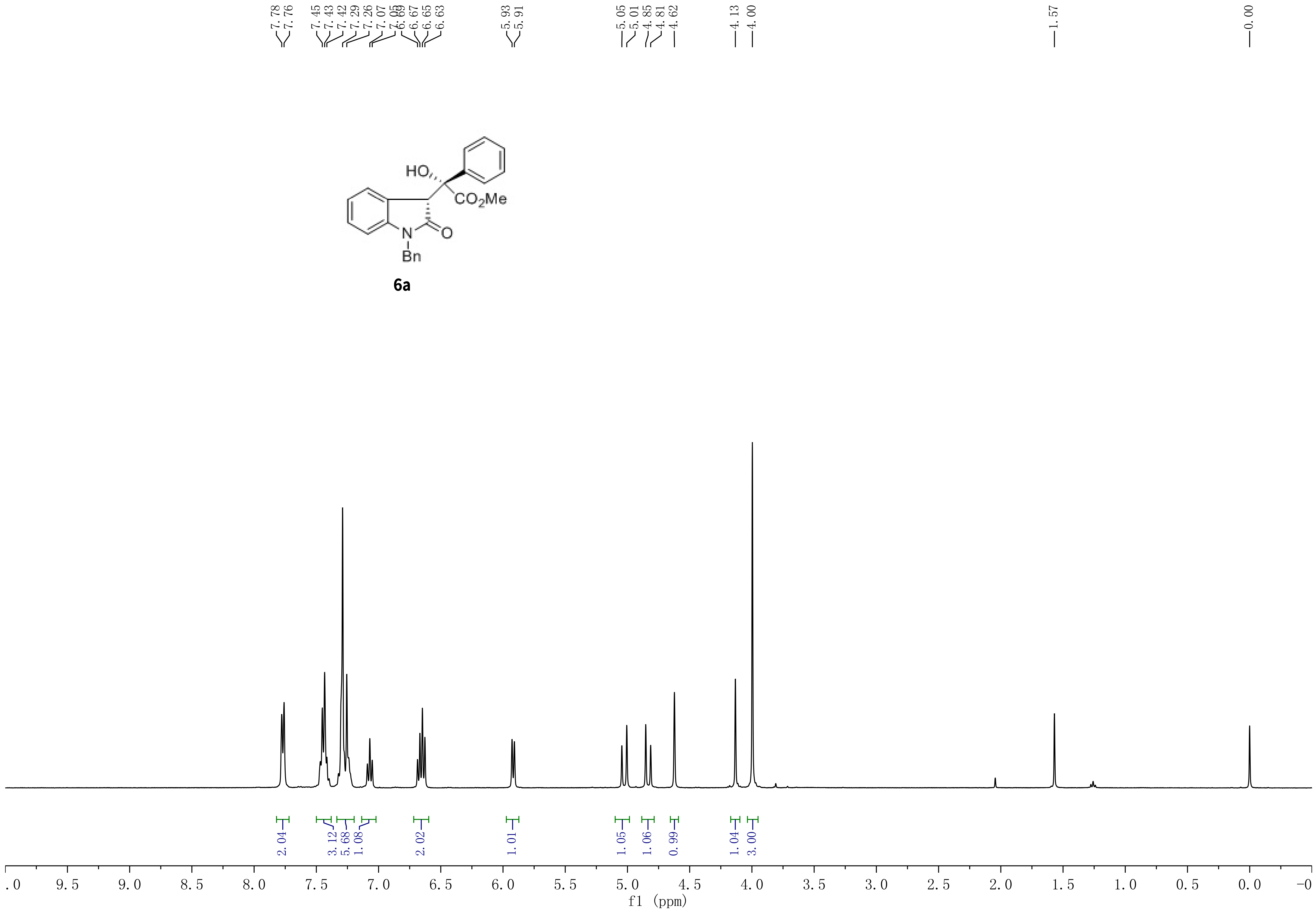
7.78
7.76
7.45
7.43
7.42
7.29
7.26
7.07
6.67
6.65
6.63
5.93
5.91
5.05
5.01
4.85
4.81
4.62
4.13
4.00
1.57
0.00
HO
CO_2Me
N
O
Bn
6a
2.04
3.12
5.68
1.08
2.02
1.01
1.05
1.06
0.99
1.04
3.00
9.5
9.0
8.5
8.0
7.5
7.0
6.5
6.0
5.5
5.0
4.5
4.0
3.5
3.0
2.5
2.0
1.5
1.0
0.5
0.0
f1 (ppm)

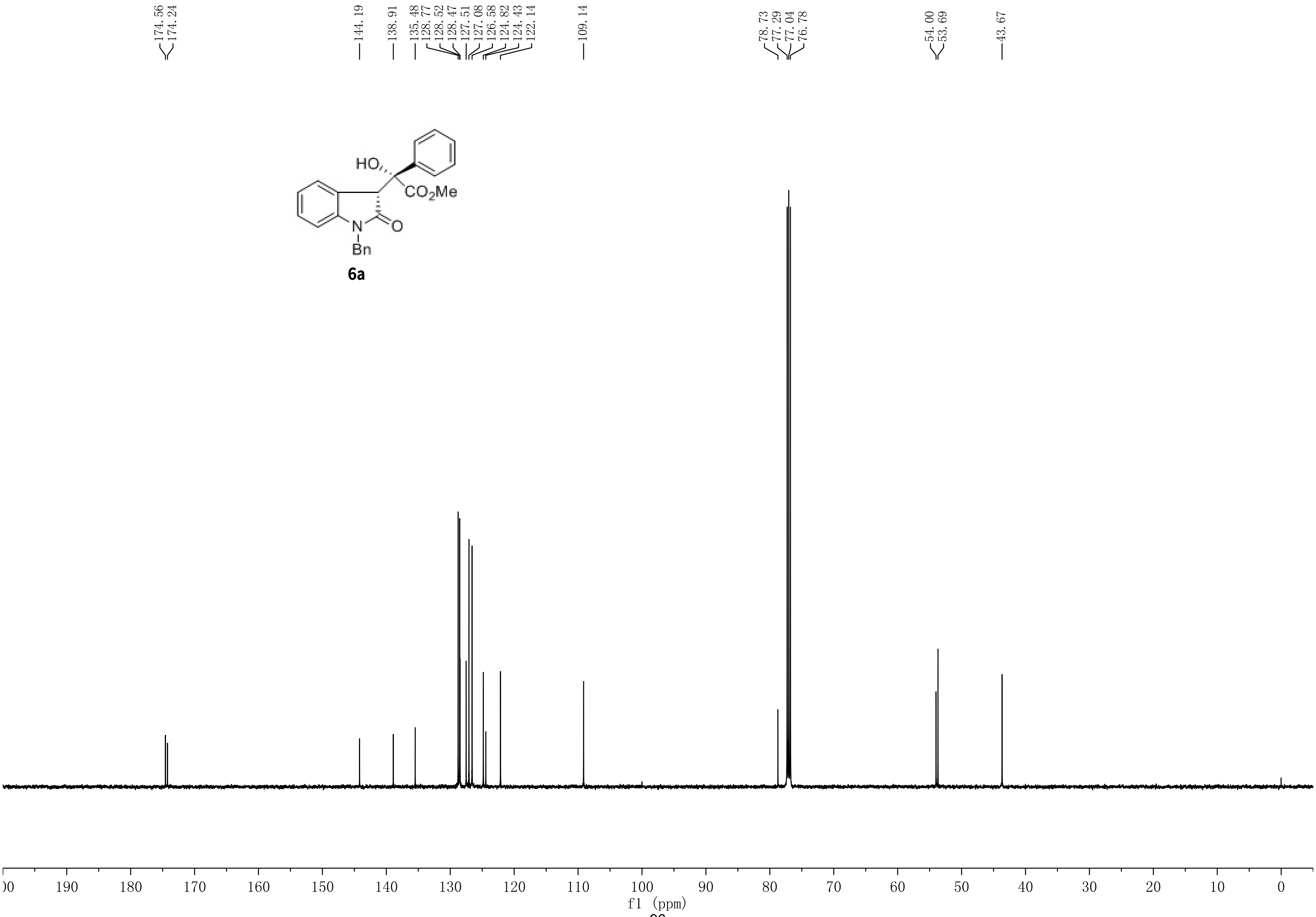
174.56
174.24
144.19
138.91
135.48
128.77
128.52
128.47
127.51
127.08
126.58
124.82
124.43
122.14
109.14
78.73
77.29
77.04
76.78
54.00
53.69
43.67
HO
CO2Me
N
O
Bn
6a
)0
190
180
170
160
150
140
130
120
110
100
90
80
70
60
50
40
30
20
10
0
f1 (ppm)

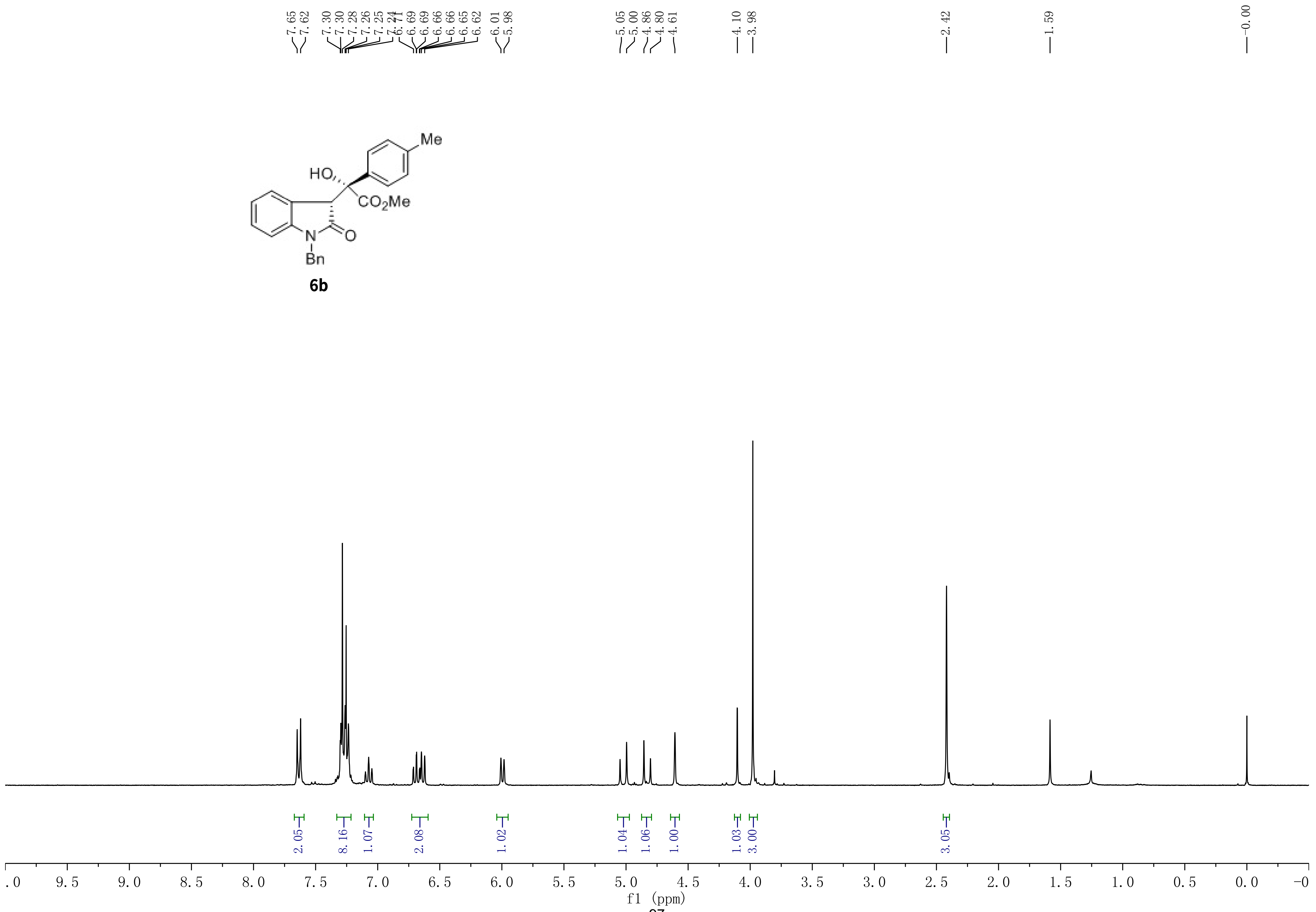
7.65 7.62 7.30 7.30 7.28 7.26 7.25 7.24 6.71 6.69 6.69 6.66 6.66 6.65 6.62 6.01 5.98
5.05 5.00 4.86 4.80 4.61
4.10 3.98
2.42
1.59
0.00
HO
Me
CO2Me
N
O
Bn
6b
2.05 8.16 1.07 2.08 1.02 1.04 1.06 1.00 1.03 3.00 3.05
f1 (ppm)

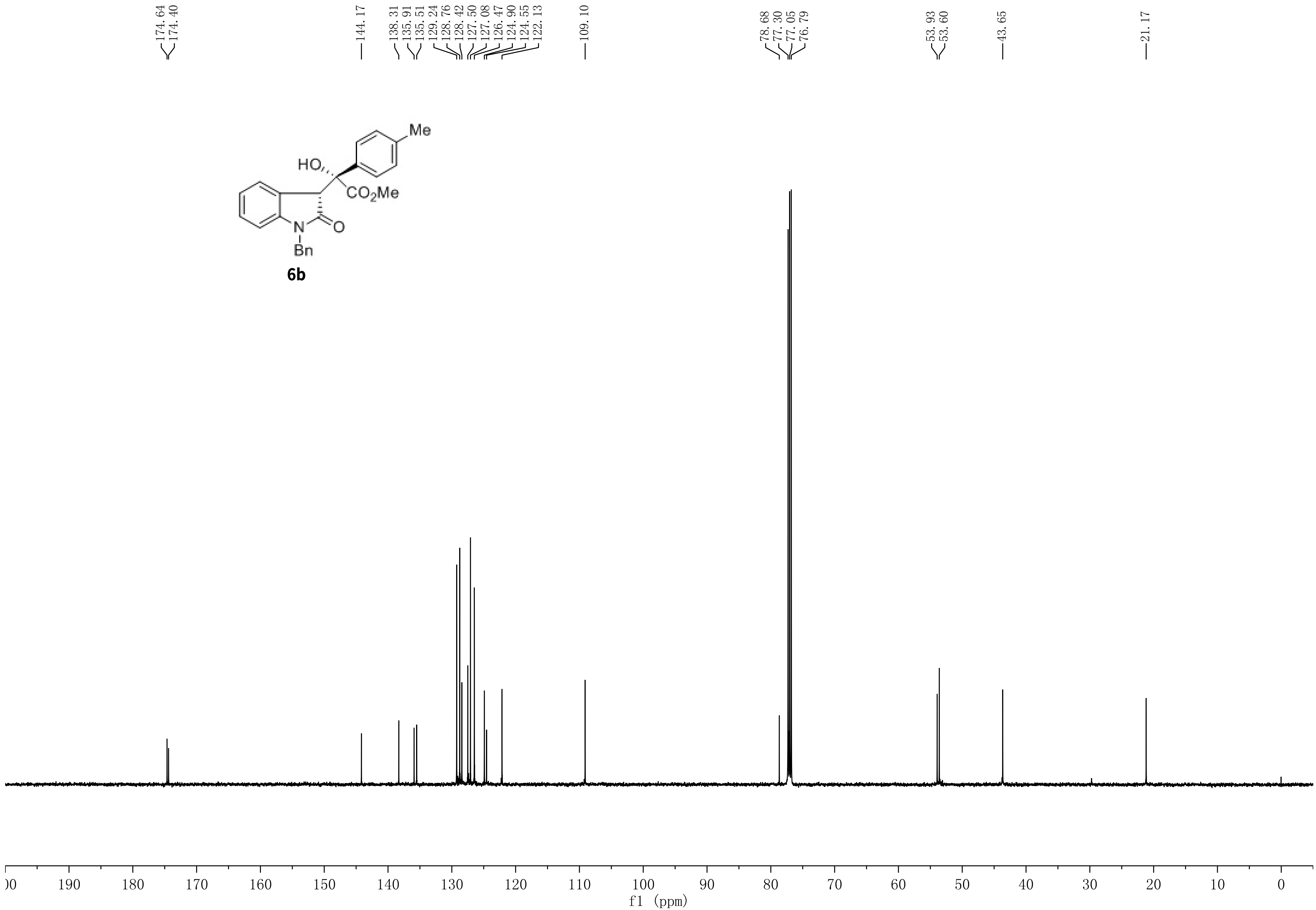
174.64
174.40
144.17
138.31
135.91
135.51
129.24
128.76
128.42
127.50
127.08
126.47
124.90
124.55
122.13
109.10
78.68
77.30
77.05
76.79
53.93
53.60
43.65
21.17
Me
HO
CO_2Me
N
O
Bn
6b
200 190 180 170 160 150 140 130 120 110 100 90 80 70 60 50 40 30 20 10 0
f1 (ppm)

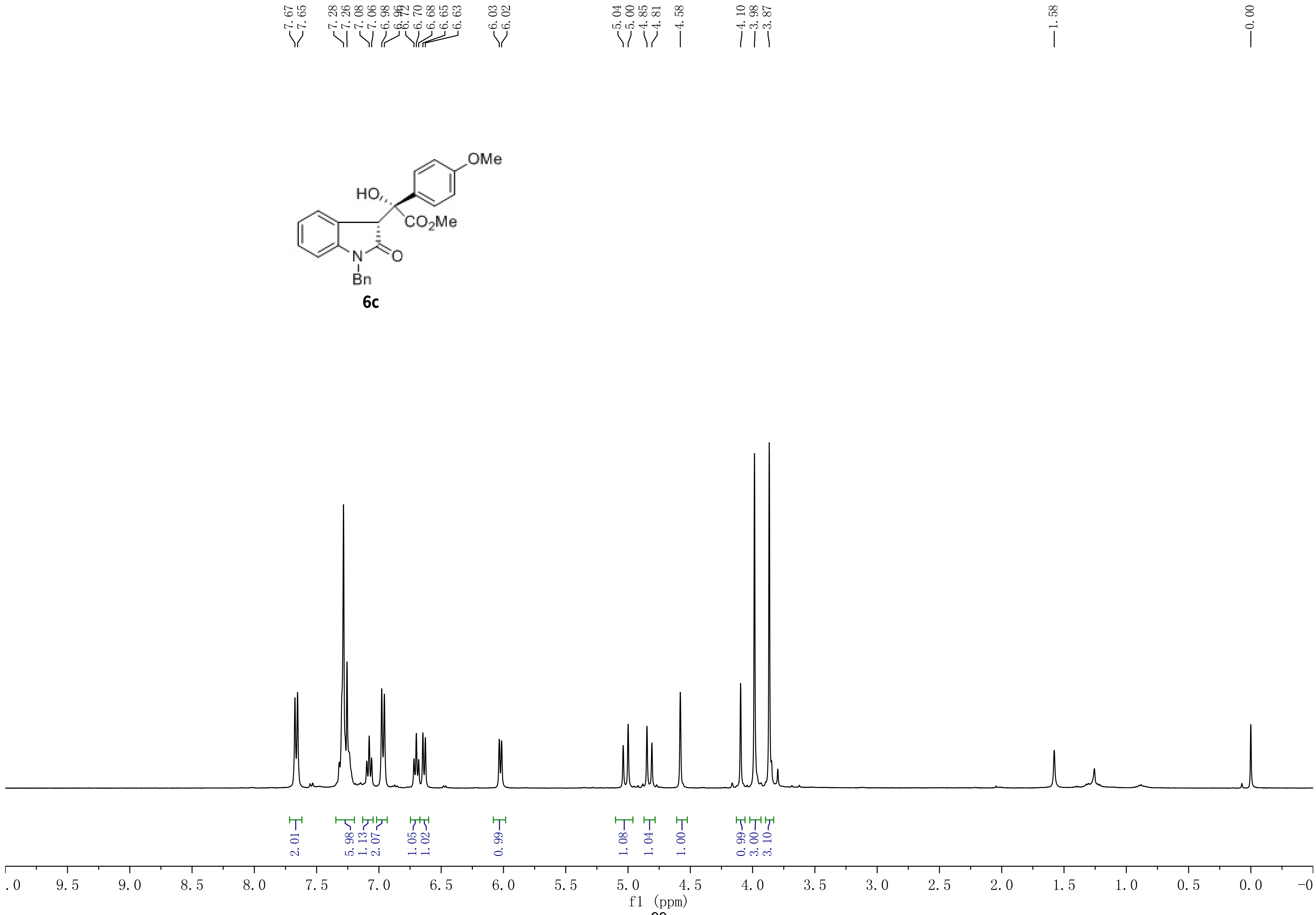
7.67
7.65
7.28
7.26
7.08
7.06
6.98
6.96
6.72
6.70
6.68
6.65
6.63
6.03
6.02
5.04
5.00
4.85
4.81
4.58
4.10
3.98
3.87
1.58
0.00
OMe
HO
CO2Me
N
O
Bn
6c
2.01
5.98
1.13
2.07
1.05
1.02
0.99
1.08
1.04
1.00
0.99
3.00
3.10
9.5
9.0
8.5
8.0
7.5
7.0
6.5
6.0
5.5
5.0
4.5
4.0
3.5
3.0
2.5
2.0
1.5
1.0
0.5
0.0
f1 (ppm)

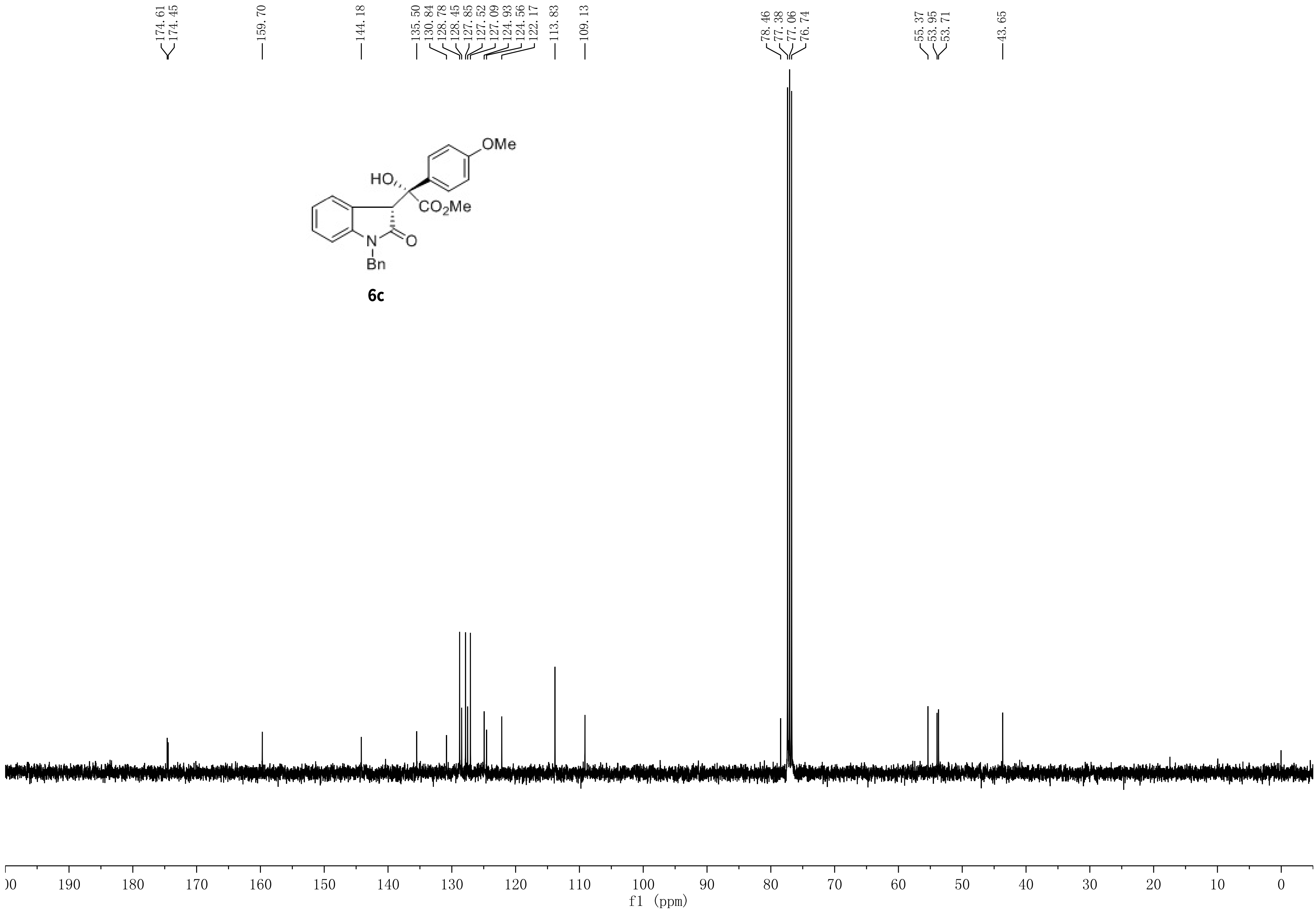

174.61
174.45
159.70
144.18
135.50
130.84
128.78
128.45
127.85
127.52
127.09
124.93
124.56
122.17
113.83
109.13
78.46
77.38
77.06
76.74
55.37
53.95
53.71
43.65
OMe
HO
CO_2Me
N
O
Bn
190
180
170
160
150
140
130
120
110
100
90
80
70
60
50
40
30
20
10
0
f1 (ppm)

6c

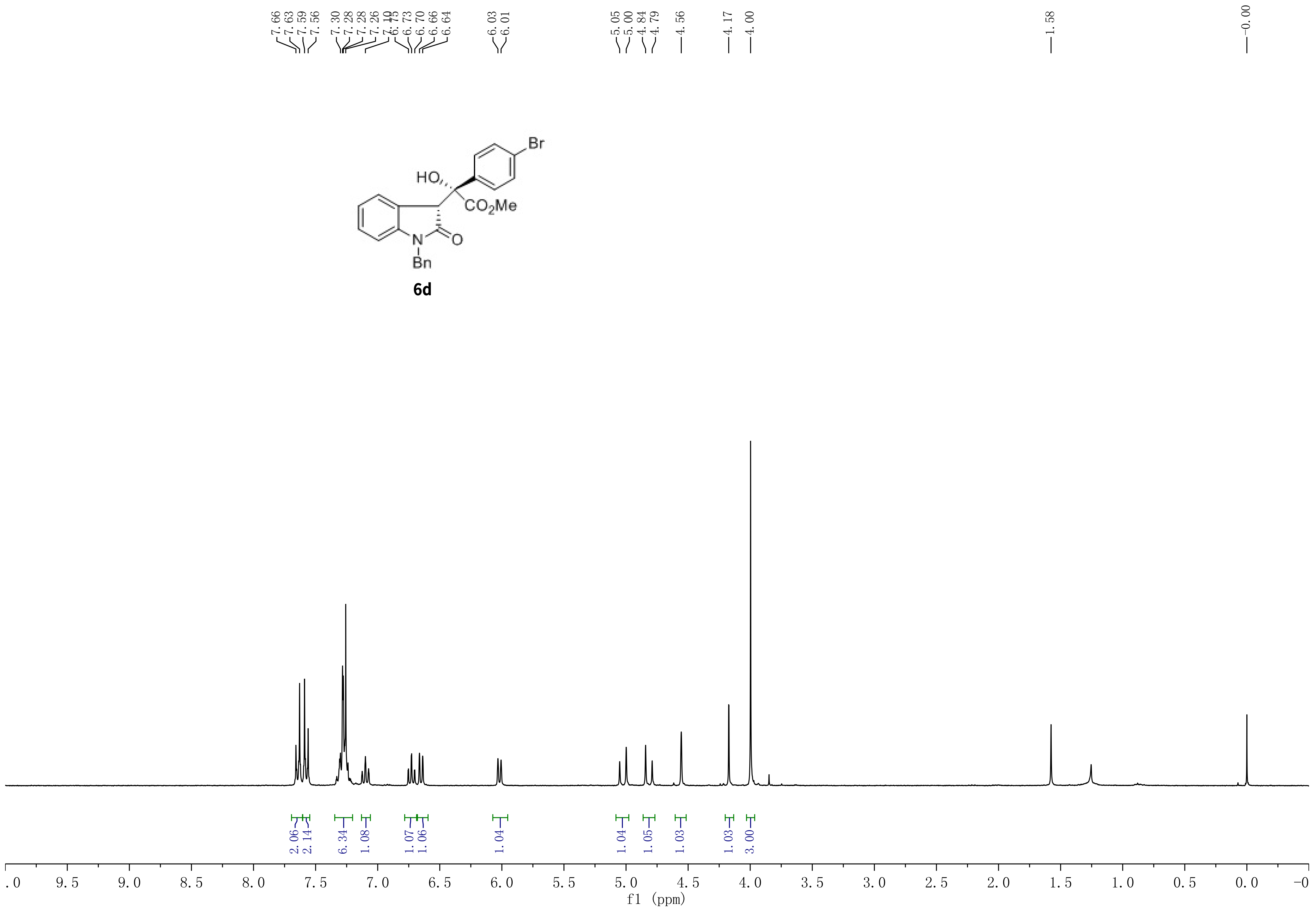
7.66
7.63
7.59
7.56
7.30
7.28
7.28
7.26
7.10
6.75
6.73
6.70
6.66
6.64
6.03
6.01
5.05
5.00
4.84
4.79
4.56
4.17
4.00
1.58
0.00
Br
HO
CO2Me
N
O
Bn
6d
2.06
2.14
6.34
1.08
1.07
1.06
1.04
1.04
1.05
1.03
1.03
3.00
9.5
9.0
8.5
8.0
7.5
7.0
6.5
6.0
5.5
5.0
4.5
4.0
3.5
3.0
2.5
2.0
1.5
1.0
0.5
0.0
f1 (ppm)

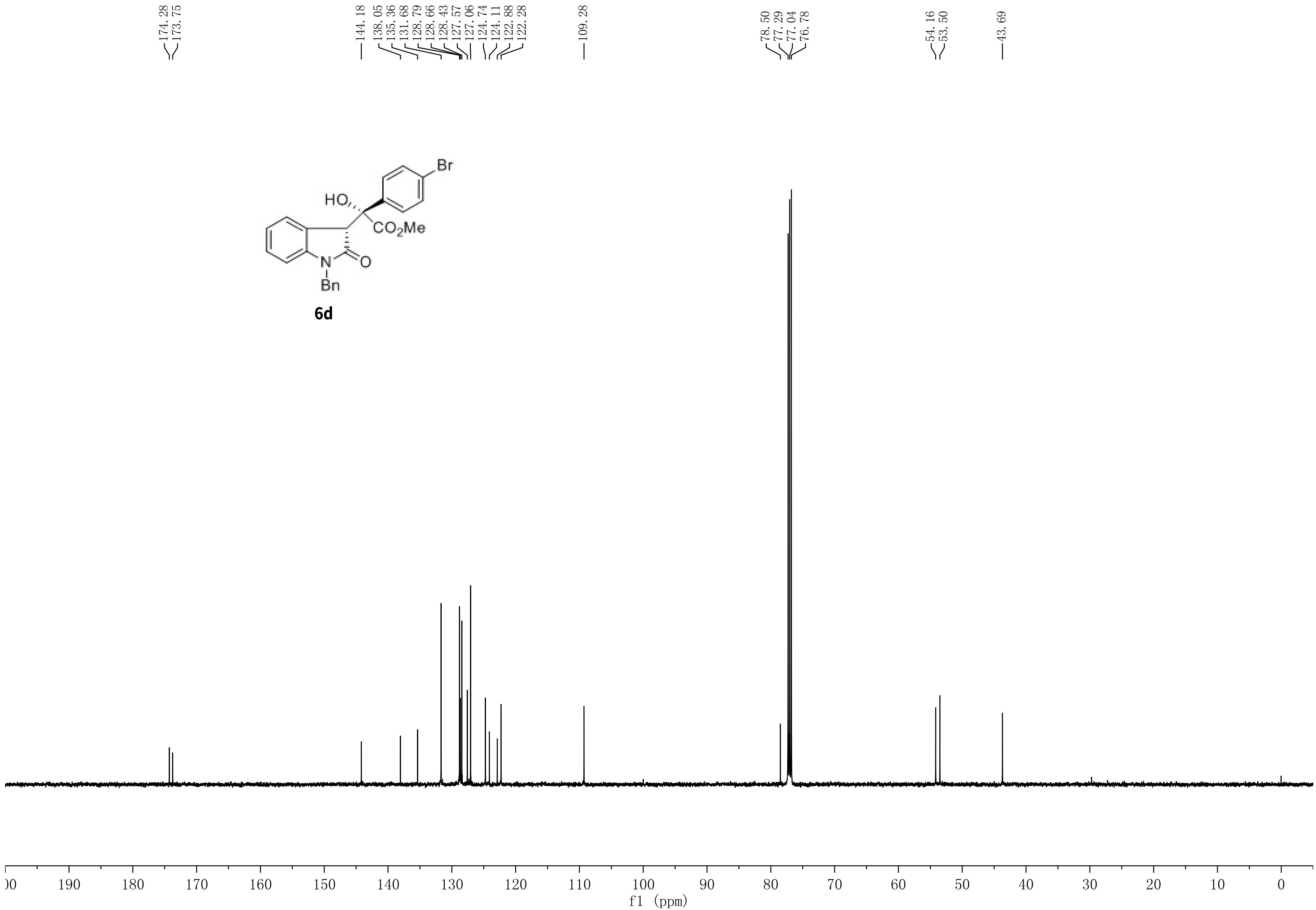
174.28
173.75
144.18
138.05
135.36
131.68
128.79
128.66
128.43
127.57
127.06
124.74
124.11
122.88
122.28
109.28
78.50
77.29
77.04
76.78
54.16
53.50
43.69
Br
HO
CO2Me
N
O
Bn
6d
f1 (ppm)

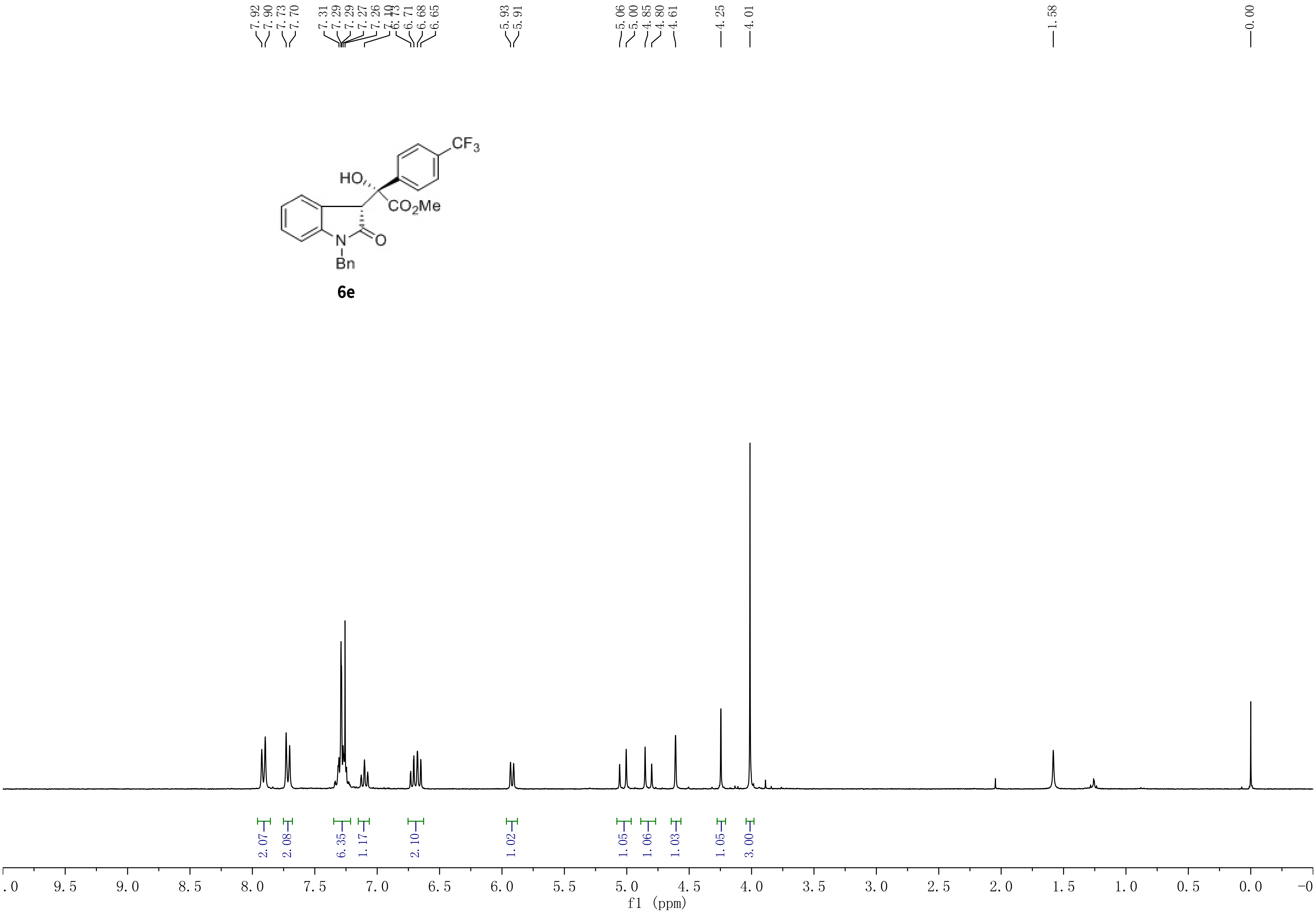
7.92
7.90
7.73
7.70
7.31
7.29
7.29
7.27
7.26
7.10
6.73
6.71
6.68
6.65
5.93
5.91
5.06
5.00
4.85
4.80
4.61
4.25
4.01
1.58
0.00
HO
CF3
CO2Me
N
O
Bn
6e
2.07
2.08
6.35
1.17
2.10
1.02
1.05
1.06
1.03
1.05
3.00
9.5
9.0
8.5
8.0
7.5
7.0
6.5
6.0
5.5
5.0
4.5
4.0
3.5
3.0
2.5
2.0
1.5
1.0
0.5
0.0
f1 (ppm)

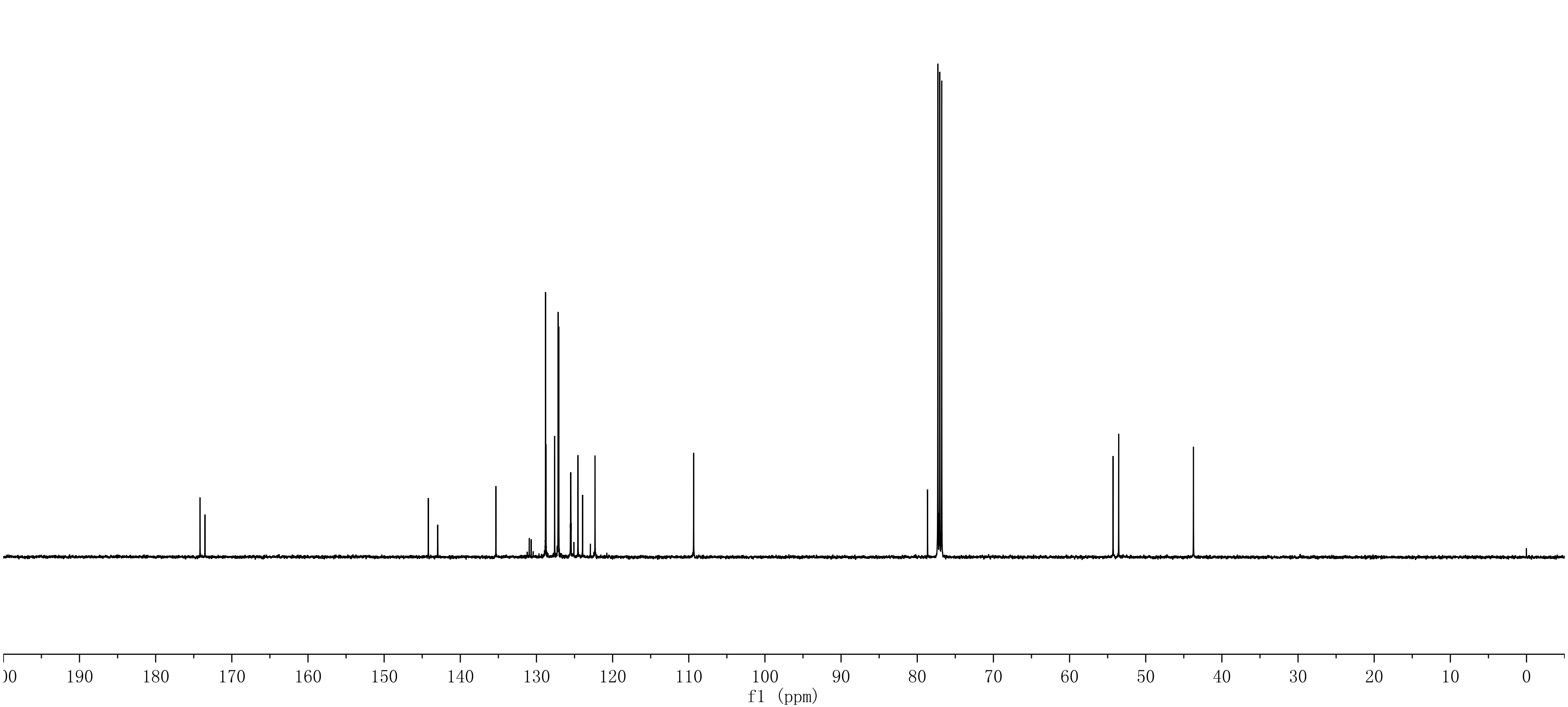
174.17
173.53
144.20
142.96
135.33
130.95
128.81
128.75
127.61
127.16
127.07
125.51
125.48
124.55
123.94
122.31
109.36
78.65
77.30
77.04
76.79
54.29
53.55
43.73
f1 (ppm)

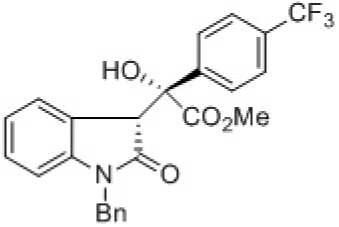
HO
CF3
CO2Me
N
O
Bn

6e

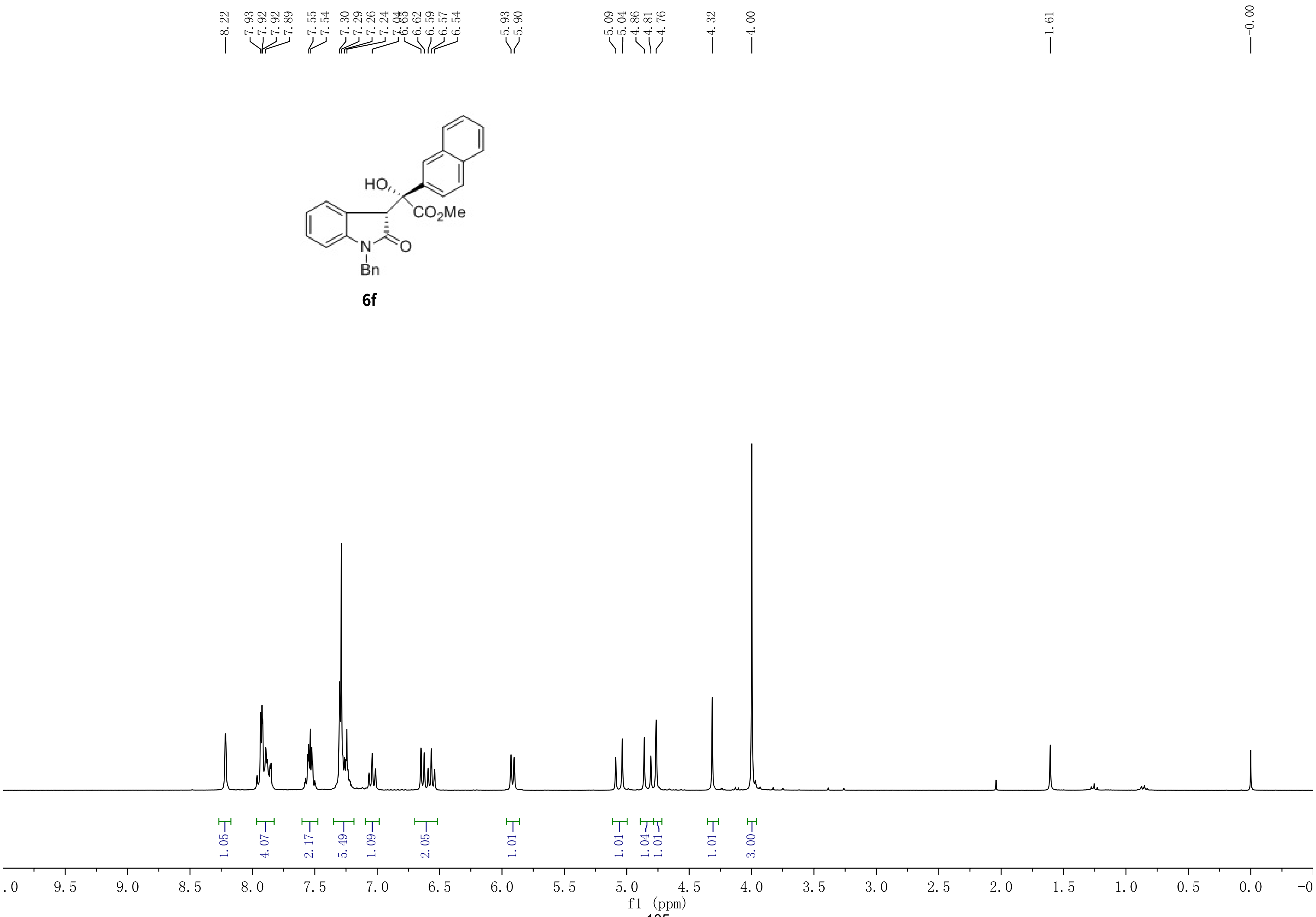
8.22
7.93
7.92
7.92
7.89
7.55
7.54
7.30
7.29
7.26
7.24
6.62
6.59
6.57
6.54
5.93
5.90
5.09
5.04
4.86
4.81
4.76
4.32
4.00
1.61
0.00
HO
CO2Me
N
O
Bn
6f
1.05
4.07
2.17
5.49
1.09
2.05
1.01
1.01
1.04
1.01
1.01
3.00
f1 (ppm)

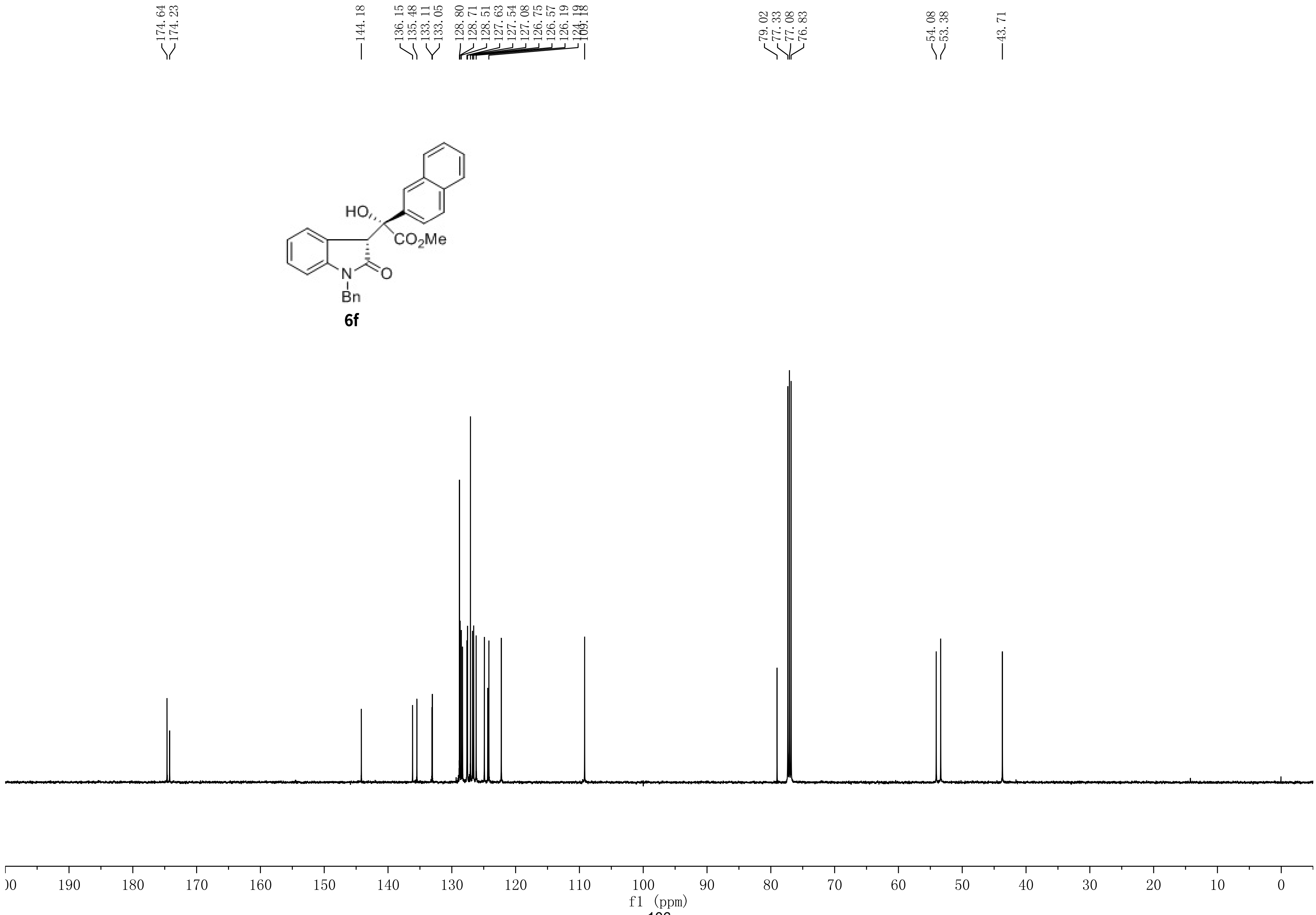

174.64
174.23
144.18
136.15
135.48
133.11
133.05
128.80
128.71
128.51
127.63
127.54
127.08
126.75
126.57
126.19
124.19
109.18
79.02
77.33
77.08
76.83
54.08
53.38
43.71
HO
CO2Me
N
O
Bn
6f
190 180 170 160 150 140 130 120 110 100 90 80 70 60 50 40 30 20 10 0
f1 (ppm)

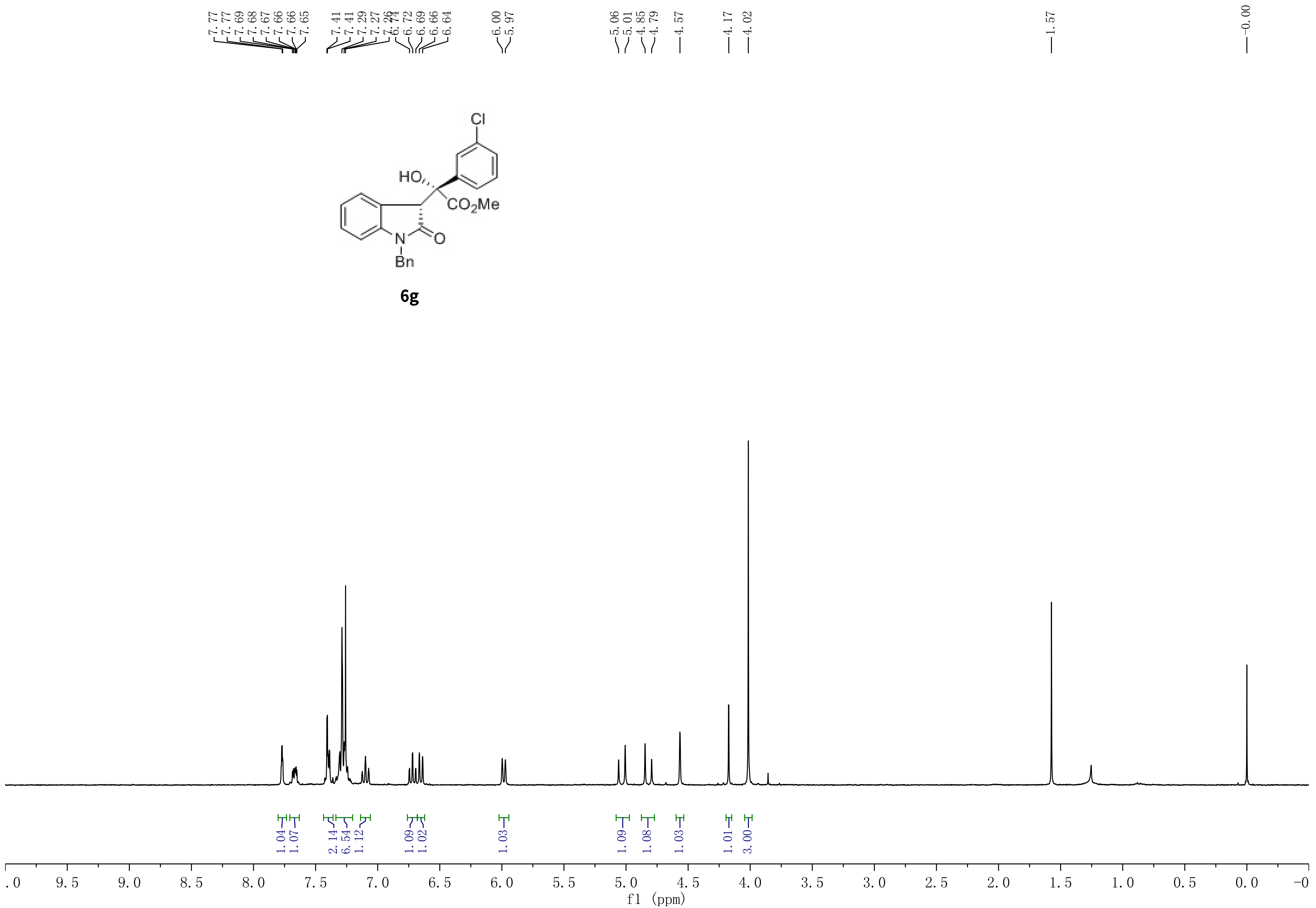
7.77 7.77 7.69 7.68 7.67 7.66 7.66 7.65
7.41 7.41 7.29 7.27 7.26 6.74 6.72 6.69 6.66 6.64
6.00 5.97
5.06 5.01 4.85 4.79
4.57
4.17
4.02
1.57
0.00
Cl
HO
CO_2Me
N
O
Bn
6g
1.04 1.07 2.14 6.54 1.12 1.09 1.02 1.03 1.09 1.08 1.03 1.01 3.00
f1 (ppm)

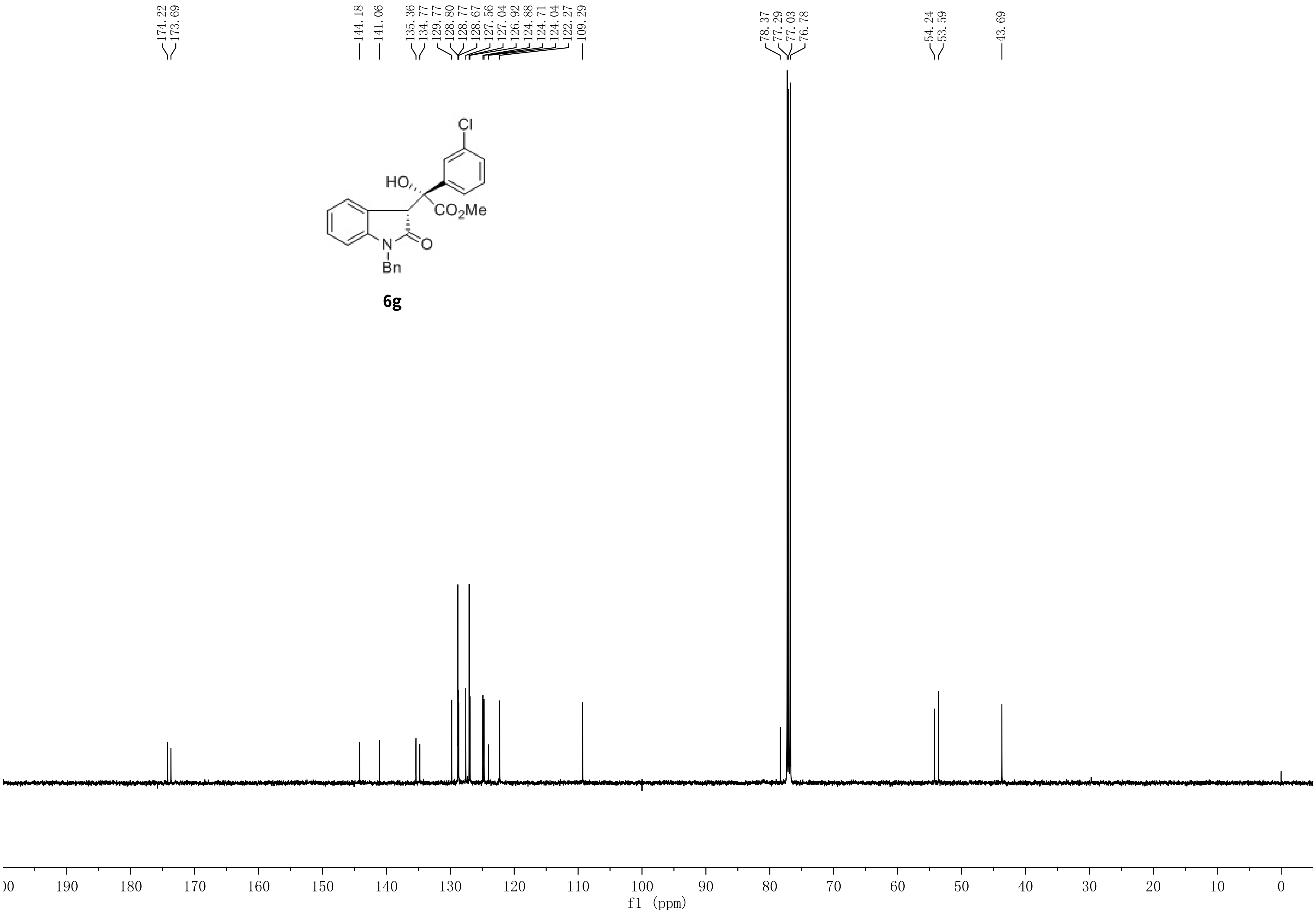
174.22
173.69
144.18
141.06
135.36
134.77
129.77
128.80
128.77
128.67
127.56
127.04
126.92
124.88
124.71
124.04
122.27
109.29
78.37
77.29
77.03
76.78
54.24
53.59
43.69
Cl
HO
CO2Me
N
O
Bn
6g
190
180
170
160
150
140
130
120
110
100
90
80
70
60
50
40
30
20
10
0
f1 (ppm)

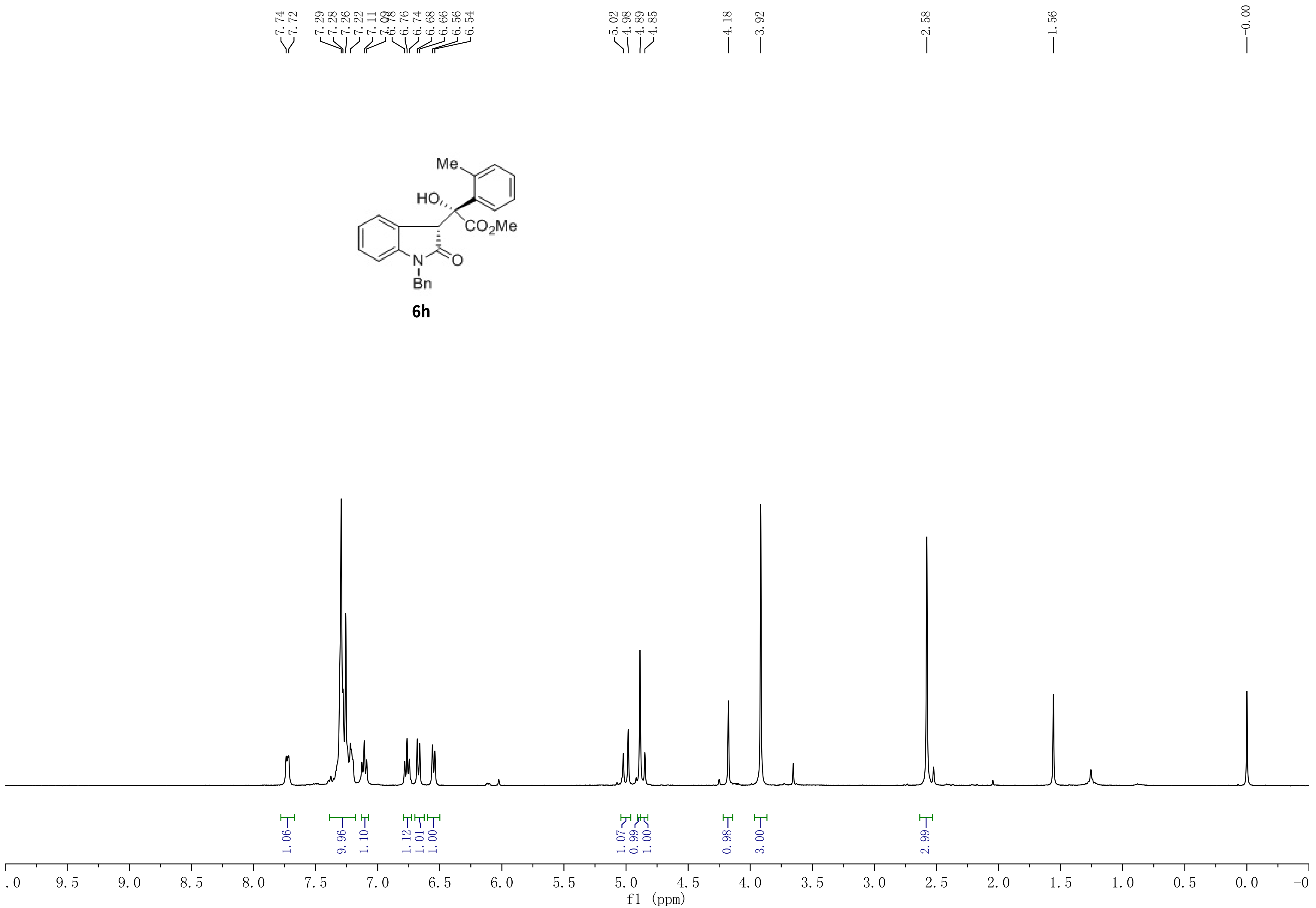
7.74
7.72
7.29
7.28
7.26
7.22
7.11
7.09
6.78
6.76
6.74
6.68
6.66
6.56
6.54
5.02
4.98
4.89
4.85
4.18
3.92
2.58
1.56
-0.00
Me
HO
CO2Me
N
O
Bn
1.06
9.96
1.10
1.12
1.01
1.00
1.07
0.99
1.00
0.98
3.00
2.99
f1 (ppm)

6h

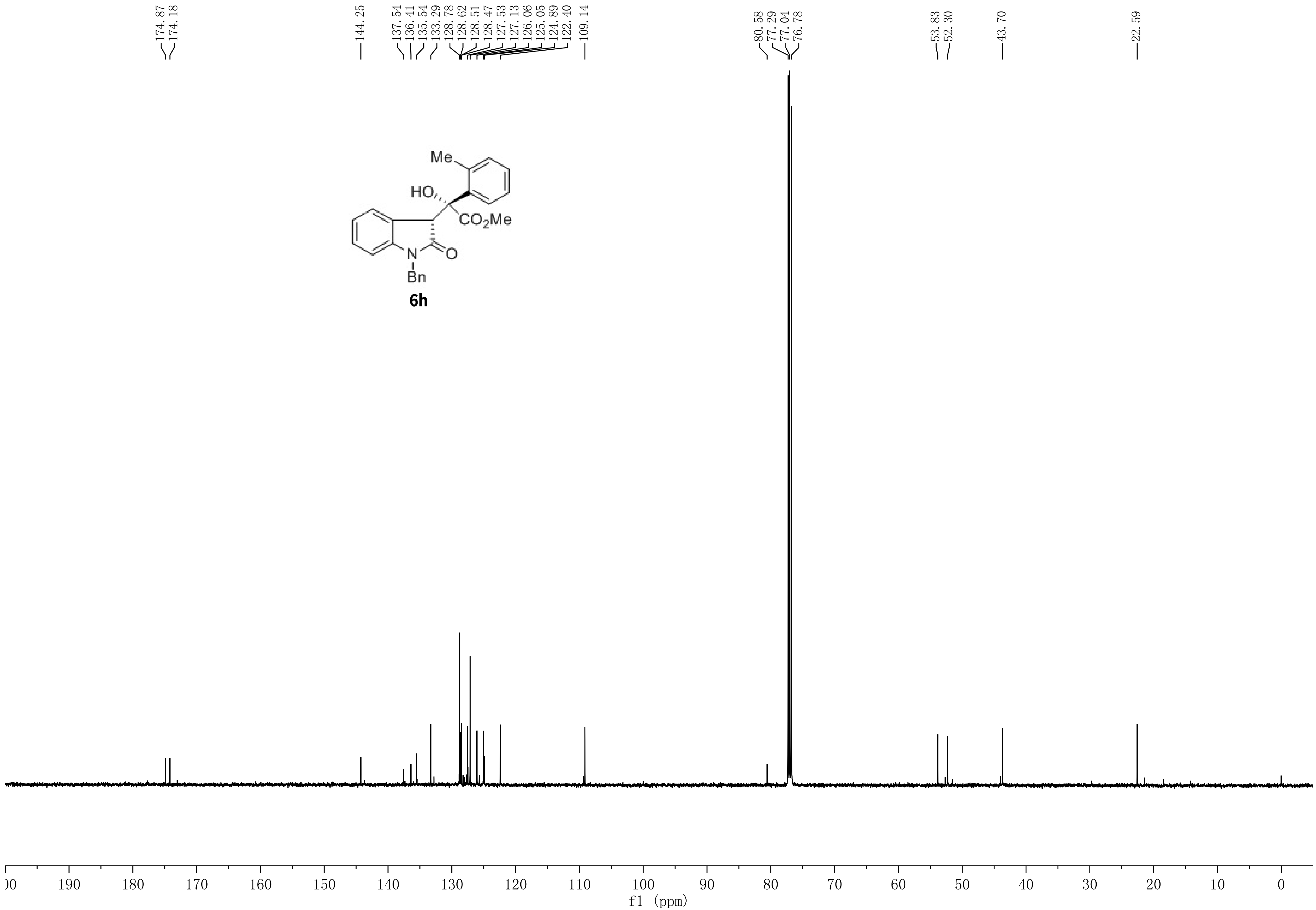
174.87
174.18
144.25
137.54
136.41
135.54
133.29
128.78
128.62
128.51
128.47
127.53
127.13
126.06
125.05
124.89
122.40
109.14
80.58
77.29
77.04
76.78
53.83
52.30
43.70
22.59
Me
HO
CO2Me
N
O
Bn
6h
190
180
170
160
150
140
130
120
110
100
90
80
70
60
50
40
30
20
10
0
f1 (ppm)

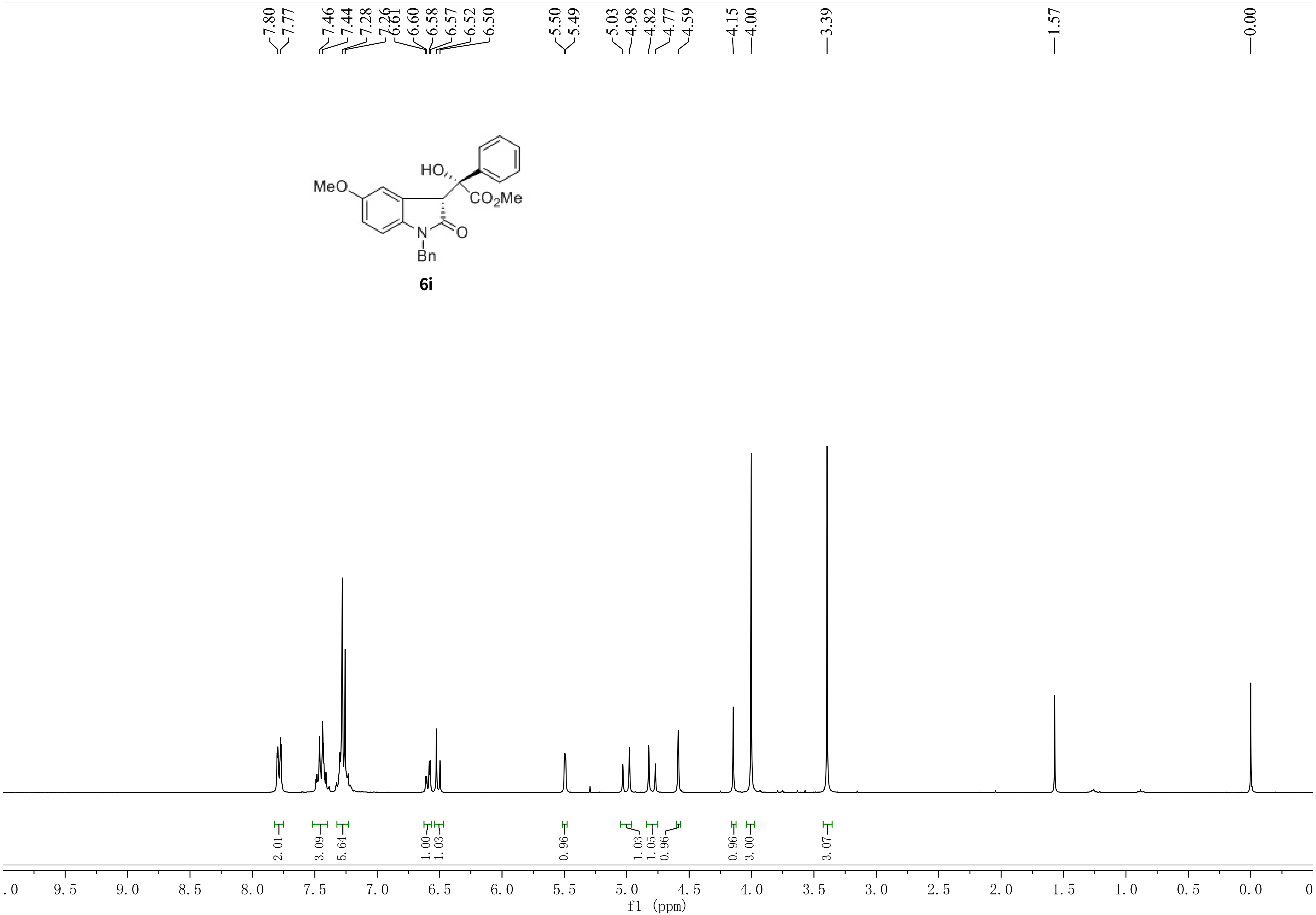
7.80
7.77
7.46
7.44
7.28
7.26
6.61
6.60
6.58
6.57
6.52
6.50
5.50
5.49
5.03
4.98
4.82
4.77
4.59
4.15
4.00
3.39
1.57
0.00
HO
MeO
CO2Me
N
O
Bn
6i
2.01
3.09
5.64
1.00
1.03
0.96
1.03
1.05
0.96
0.96
3.00
3.07
f1 (ppm)

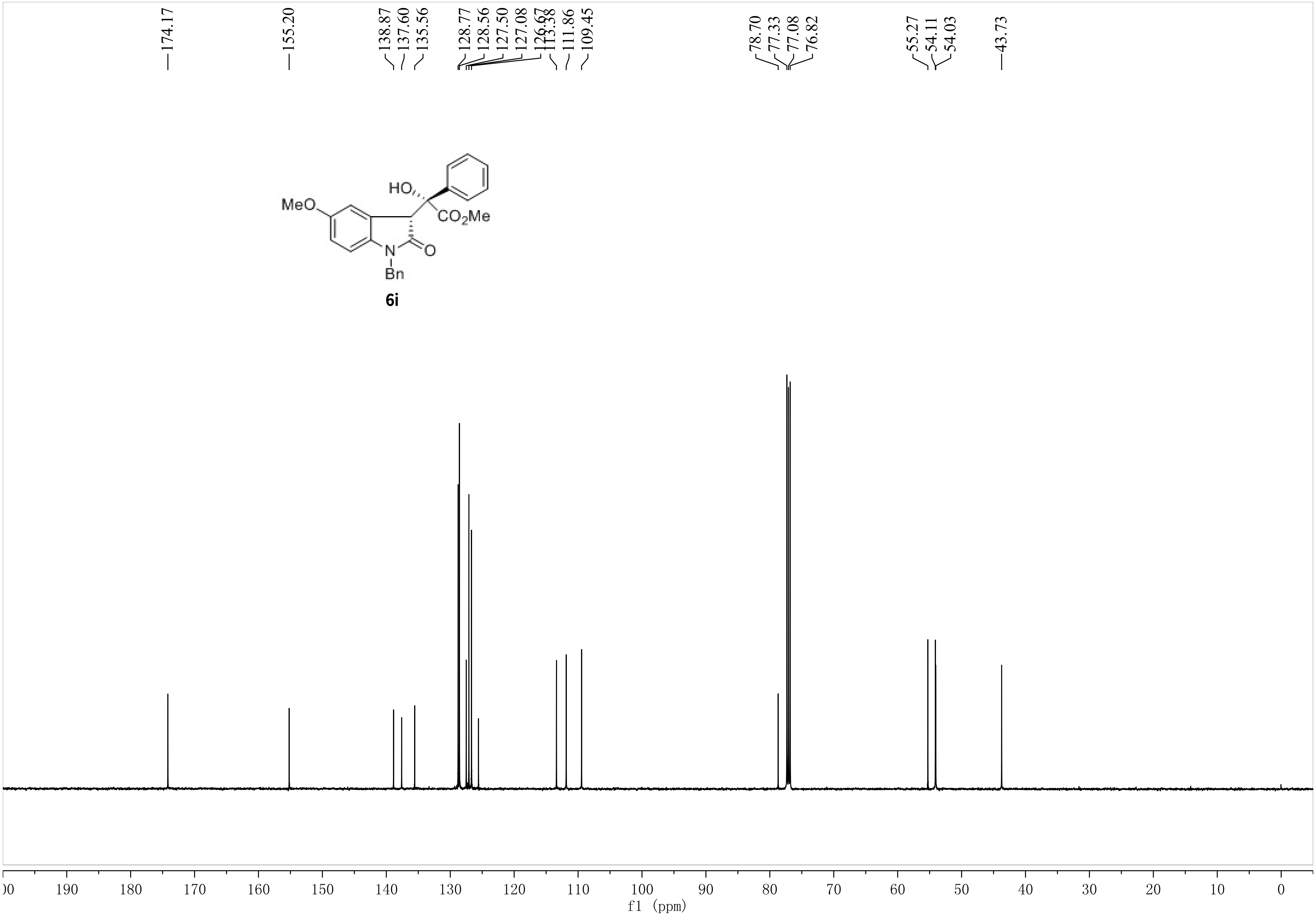
174.17
155.20
138.87
137.60
135.56
128.77
128.56
127.50
127.08
126.67
113.38
111.86
109.45
78.70
77.33
77.08
76.82
55.27
54.11
54.03
43.73
MeO
HO
CO2Me
N
O
Bn
6i
f1 (ppm)

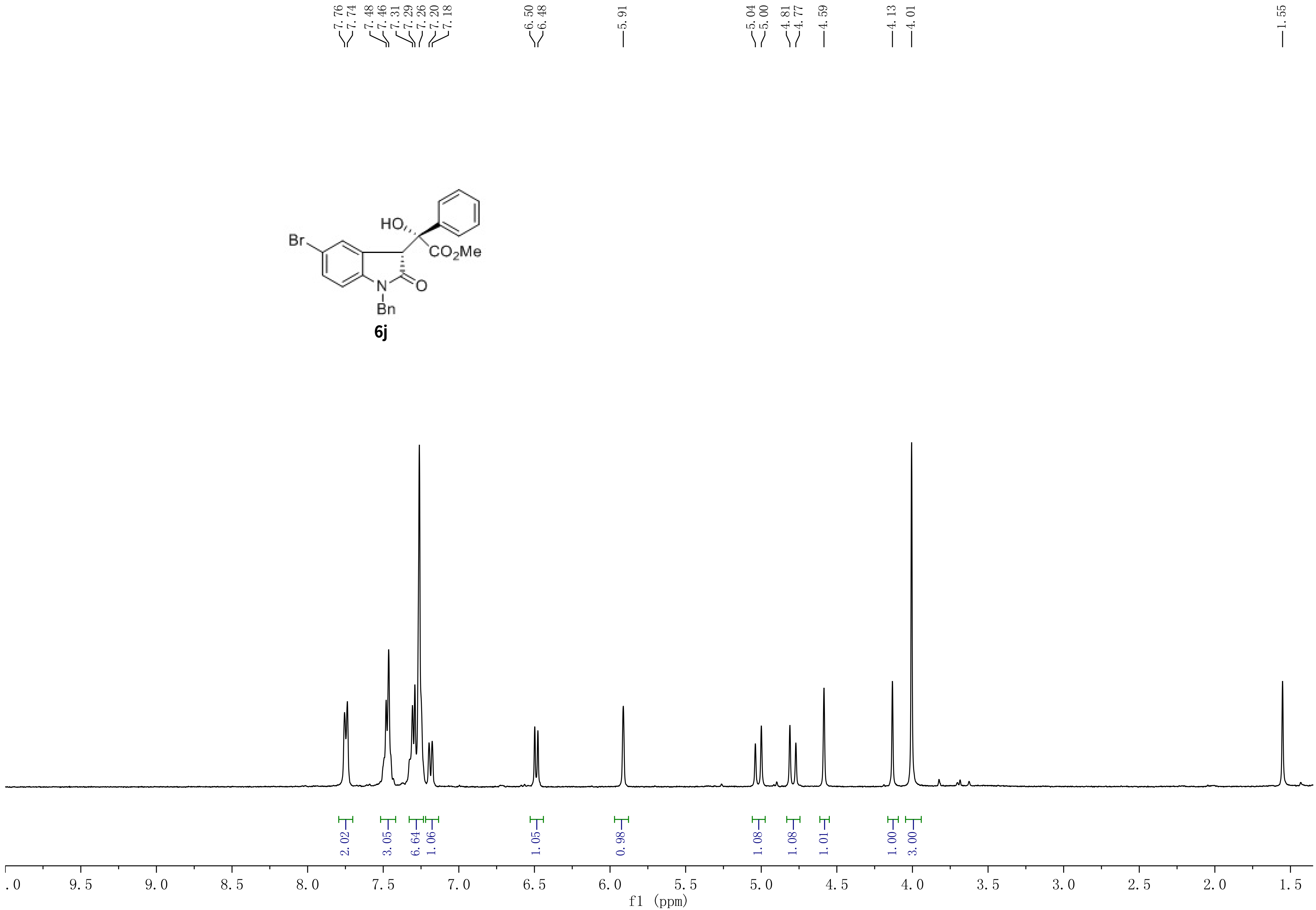
7.76
7.74
7.48
7.46
7.31
7.29
7.26
7.20
7.18
6.50
6.48
5.91
5.04
5.00
4.81
4.77
4.59
4.13
4.01
1.55
Br
HO
CO2Me
N
O
Bn
6j
2.02
3.05
6.64
1.06
1.05
0.98
1.08
1.08
1.01
1.00
3.00
.0
9.5
9.0
8.5
8.0
7.5
7.0
6.5
6.0
5.5
5.0
4.5
4.0
3.5
3.0
2.5
2.0
1.5
f1 (ppm)

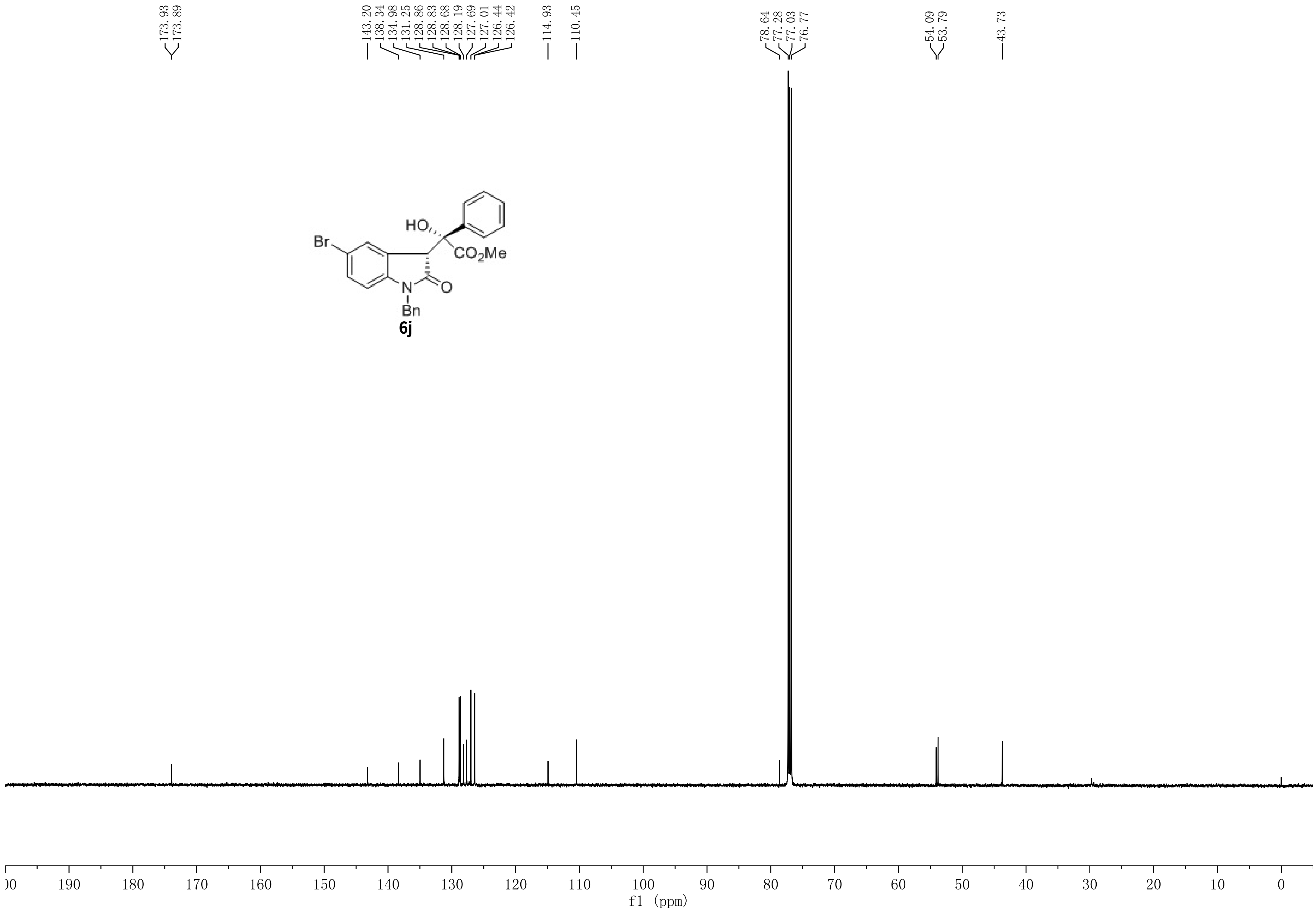
173.93
173.89
143.20
138.34
134.98
131.25
128.86
128.83
128.68
128.19
127.69
127.01
126.44
126.42
114.93
110.45
78.64
77.28
77.03
76.77
54.09
53.79
43.73
Br
HO
CO2Me
N
O
Bn
6j
190 180 170 160 150 140 130 120 110 100 90 80 70 60 50 40 30 20 10 0
f1 (ppm)

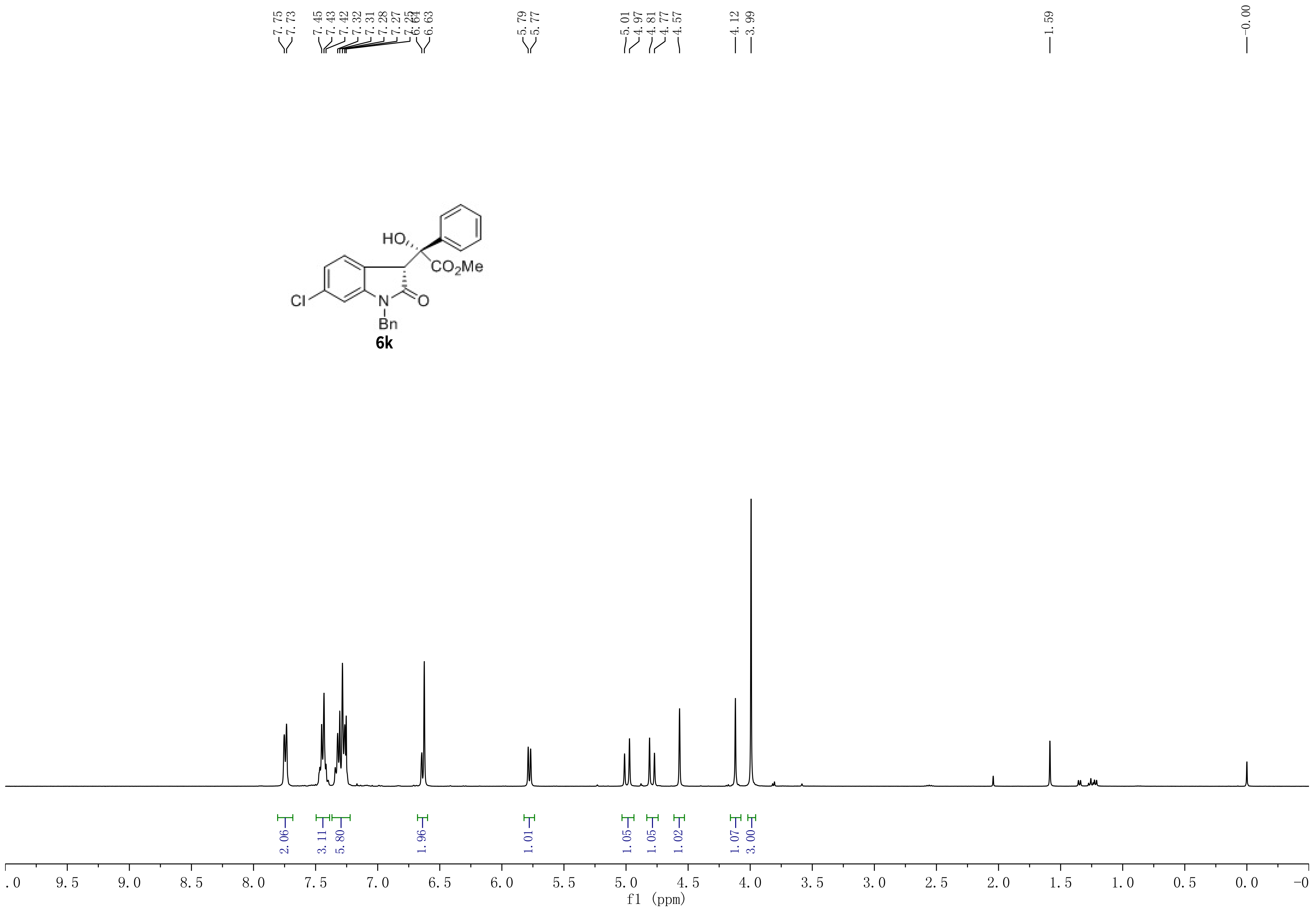
7.75
7.73
7.45
7.43
7.42
7.32
7.31
7.28
7.27
7.25
6.64
6.63
5.79
5.77
5.01
4.97
4.81
4.77
4.57
4.12
3.99
1.59
0.00
HO
CO2Me
Cl
N
O
Bn
6k
2.06
3.11
5.80
1.96
1.01
1.05
1.05
1.02
1.07
3.00
.0
9.5
9.0
8.5
8.0
7.5
7.0
6.5
6.0
5.5
5.0
4.5
4.0
3.5
3.0
2.5
2.0
1.5
1.0
0.5
0.0
-0
f1 (ppm)

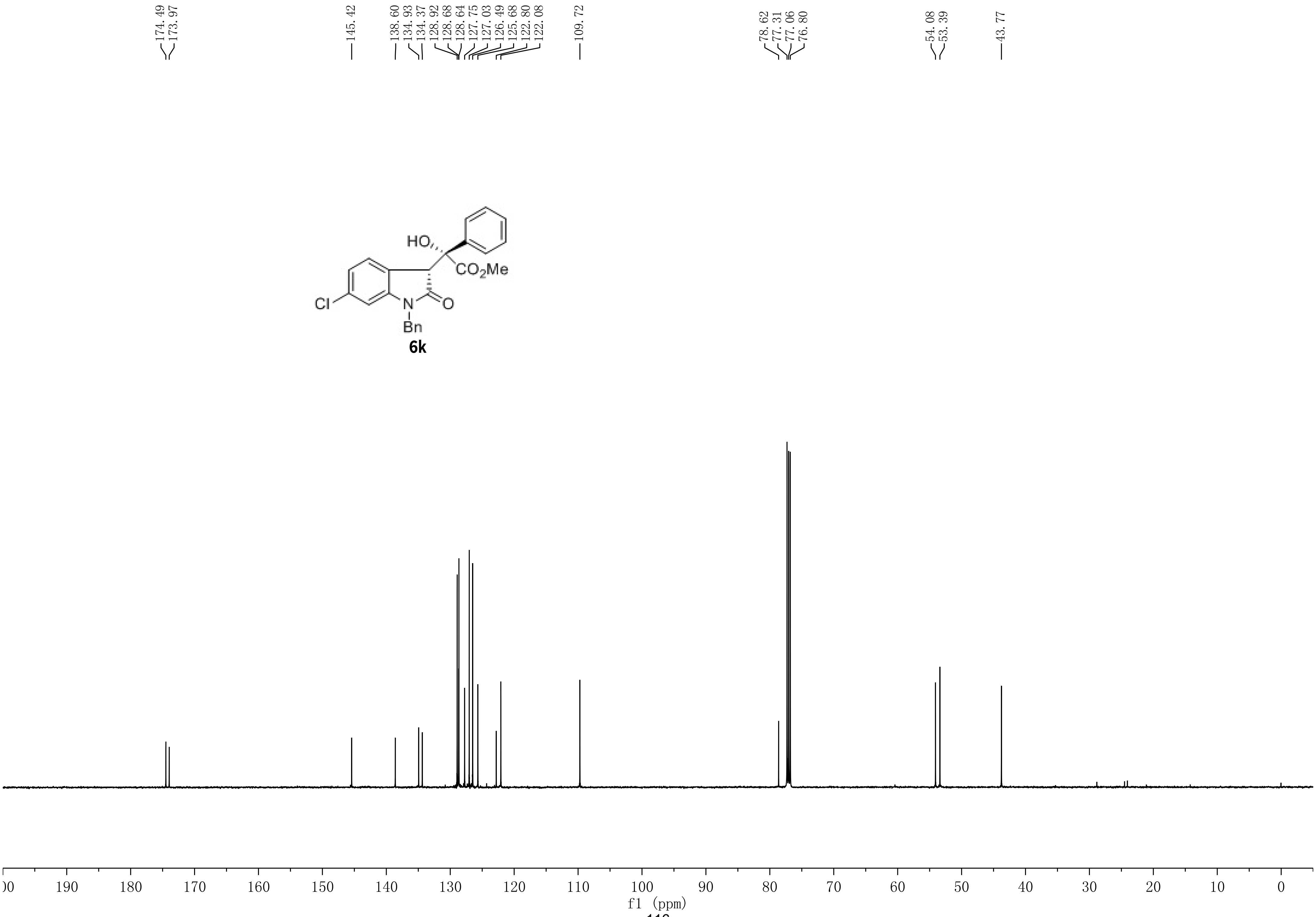
174.49
173.97
145.42
138.60
134.93
134.37
128.92
128.68
128.64
127.75
127.03
126.49
125.68
122.80
122.08
109.72
78.62
77.31
77.06
76.80
54.08
53.39
43.77
HO
CO2Me
Cl
N
O
Bn
6k
f1 (ppm)

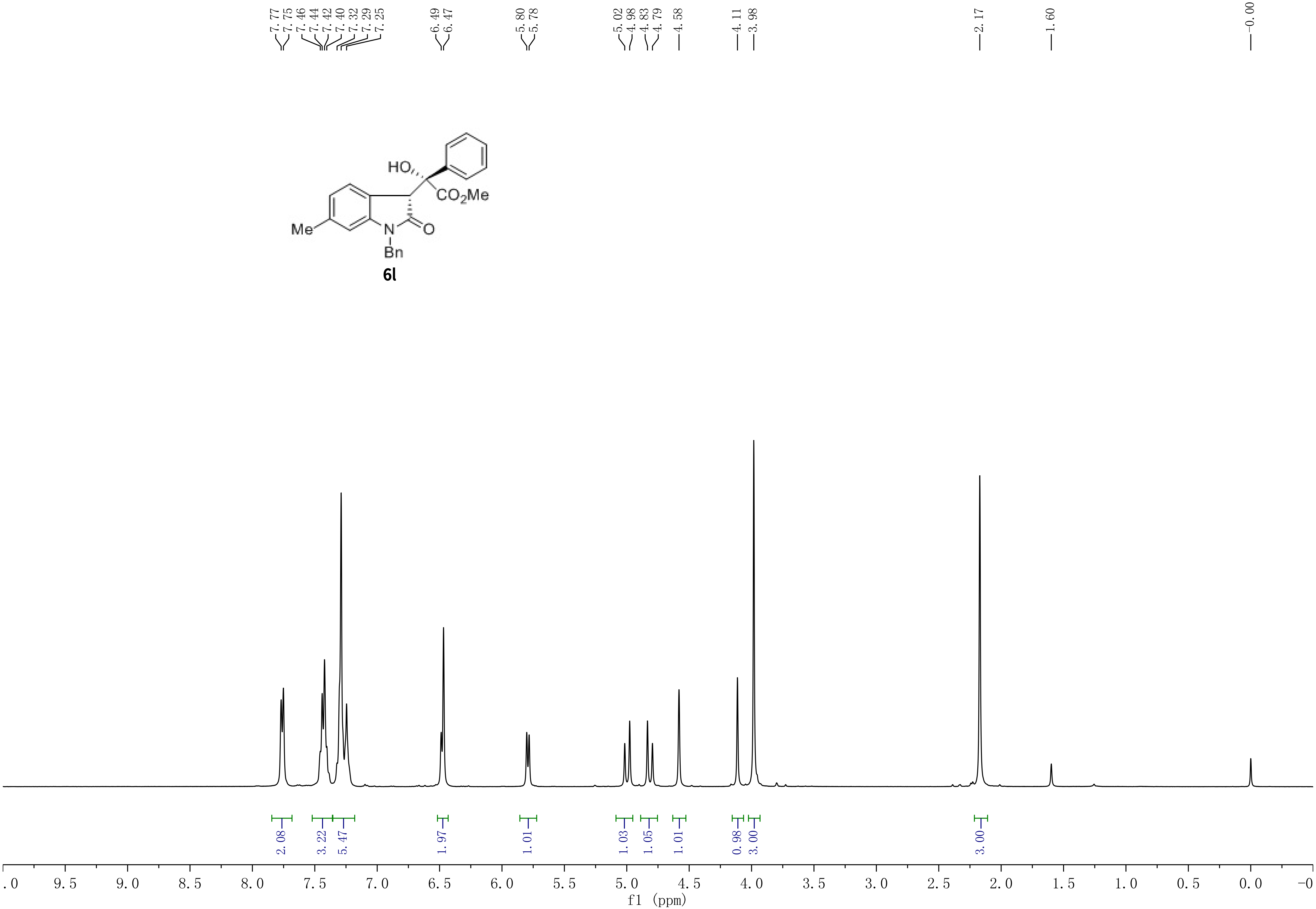
7.77
7.75
7.46
7.44
7.42
7.40
7.32
7.29
7.25
6.49
6.47
5.80
5.78
5.02
4.98
4.83
4.79
4.58
4.11
3.98
2.17
1.60
-0.00
HO
CO2Me
Me
N
O
Bn
6l
2.08
3.22
5.47
1.97
1.01
1.03
1.05
1.01
0.98
3.00
3.00
9.5
9.0
8.5
8.0
7.5
7.0
6.5
6.0
5.5
5.0
4.5
4.0
3.5
3.0
2.5
2.0
1.5
1.0
0.5
0.0
f1 (ppm)

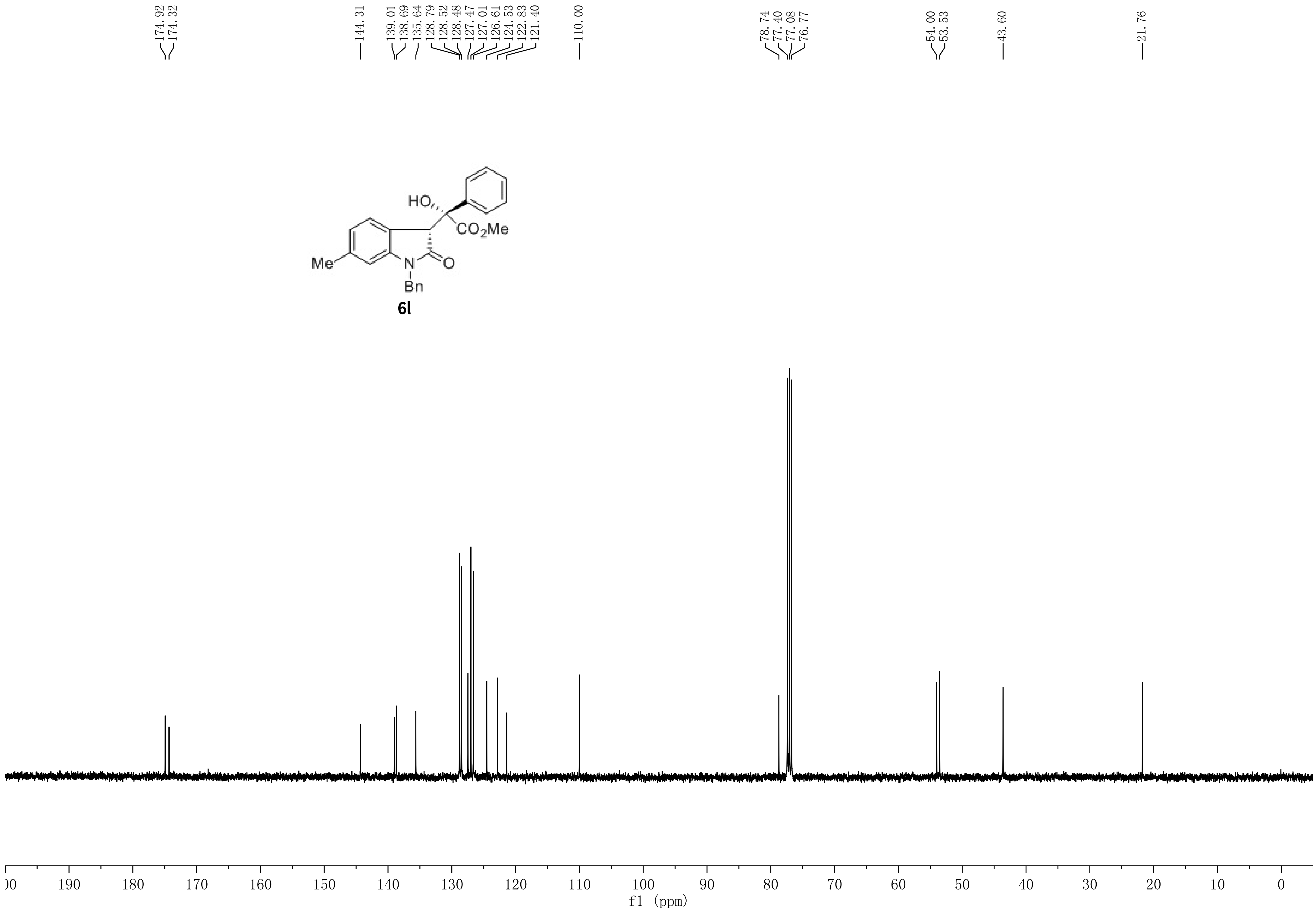
174.92
174.32
144.31
139.01
138.69
135.64
128.79
128.52
128.48
127.47
127.01
126.61
124.53
122.83
121.40
110.00
78.74
77.40
77.08
76.77
54.00
53.53
43.60
21.76
HO
CO2Me
Me
N
O
Bn
6l
190
180
170
160
150
140
130
120
110
100
90
80
70
60
50
40
30
20
10
0
f1 (ppm)

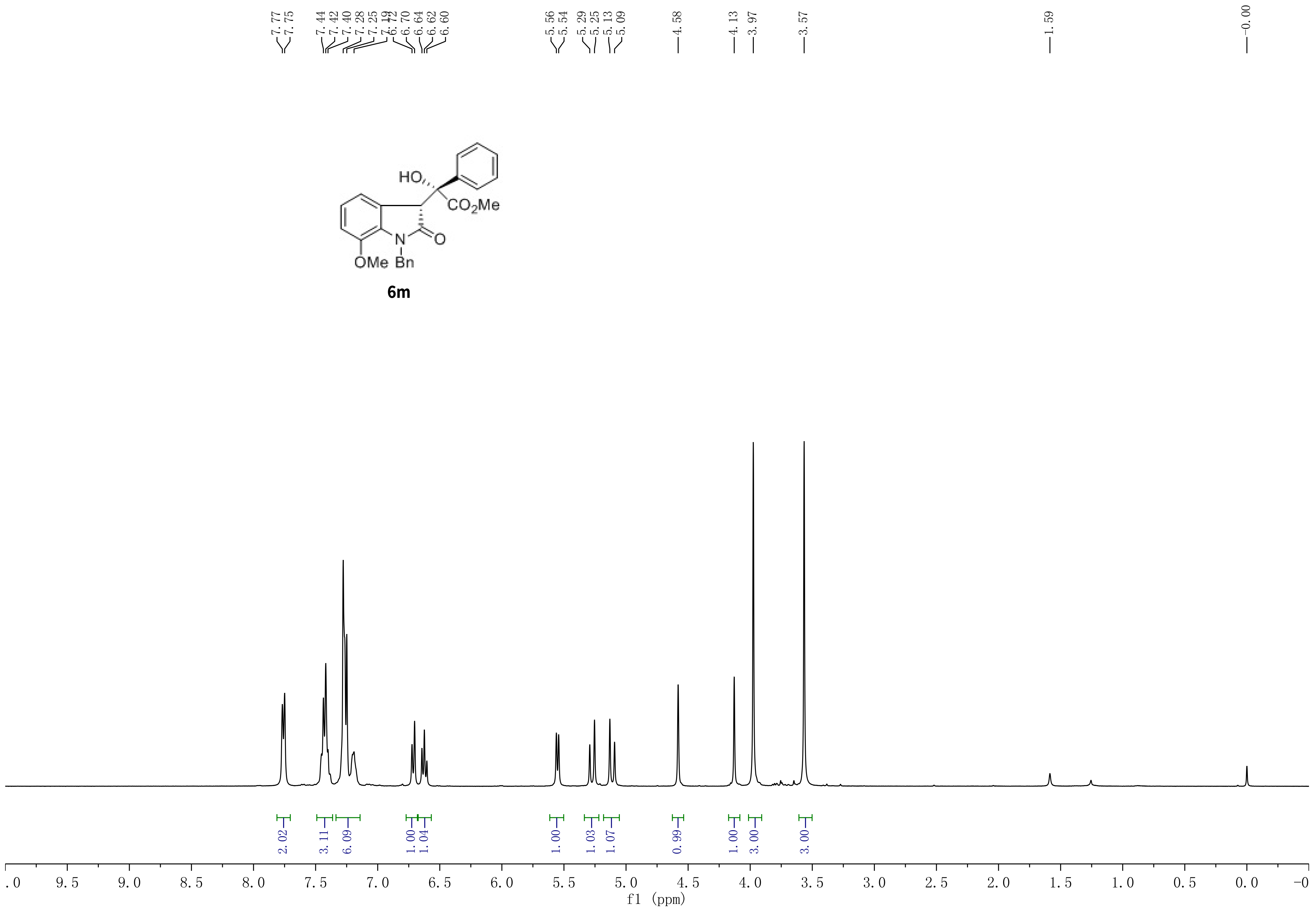
7.77
7.75
7.44
7.42
7.40
7.28
7.25
6.70
6.64
6.62
6.60
5.56
5.54
5.29
5.25
5.13
5.09
4.58
4.13
3.97
3.57
1.59
0.00
HO
CO_2Me
N
O
OMe
Bn
6m
2.02
3.11
6.09
1.00
1.04
1.00
1.03
1.07
0.99
1.00
3.00
3.00
f1 (ppm)

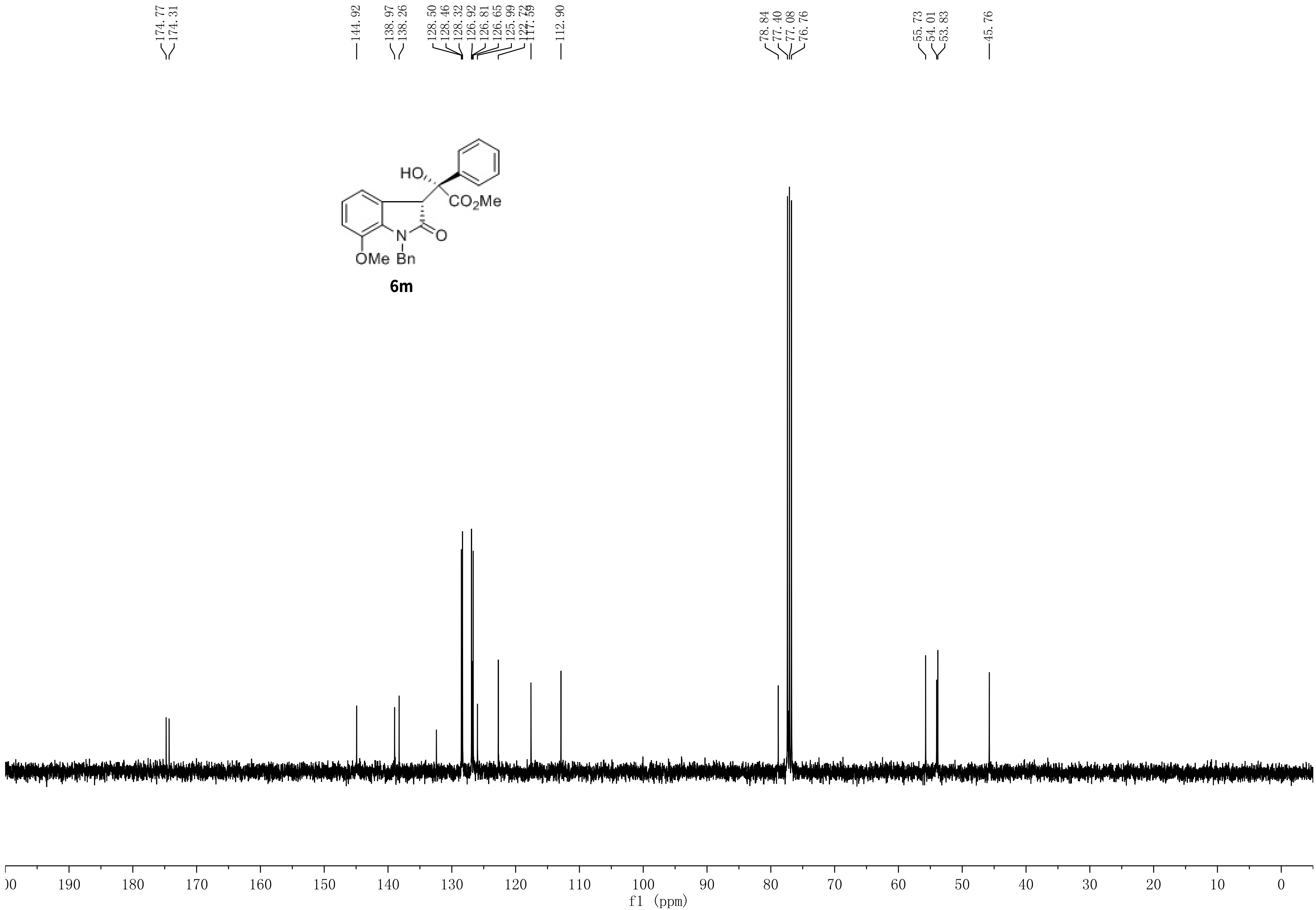
174.77
174.31
144.92
138.97
138.26
128.50
128.46
128.32
126.92
126.81
126.65
125.99
122.72
117.59
112.90
78.84
77.40
77.08
76.76
55.73
54.01
53.83
45.76
HO
CO_2Me
N
O
OMe
Bn
6m
190 180 170 160 150 140 130 120 110 100 90 80 70 60 50 40 30 20 10 0
f1 (ppm)

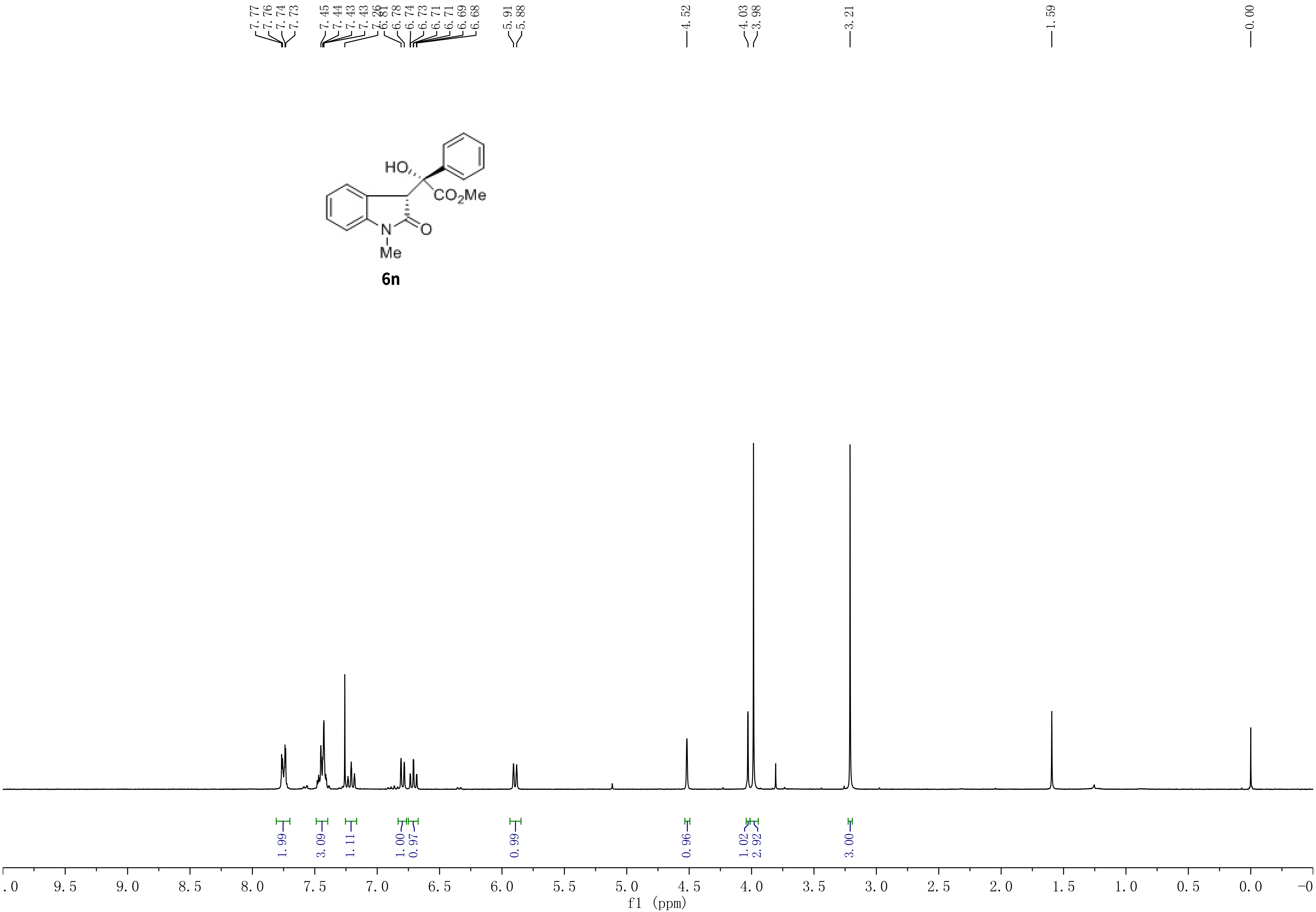
7.77
7.76
7.74
7.73
7.45
7.44
7.43
7.43
7.26
6.81
6.78
6.74
6.73
6.71
6.71
6.69
6.68
5.91
5.88
4.52
4.03
3.98
3.21
1.59
0.00
HO
CO2Me
N
O
Me
6n
1.99
3.09
1.11
1.00
0.97
0.99
0.96
1.02
2.92
3.00
9.5
9.0
8.5
8.0
7.5
7.0
6.5
6.0
5.5
5.0
4.5
4.0
3.5
3.0
2.5
2.0
1.5
1.0
0.5
0.0
f1 (ppm)

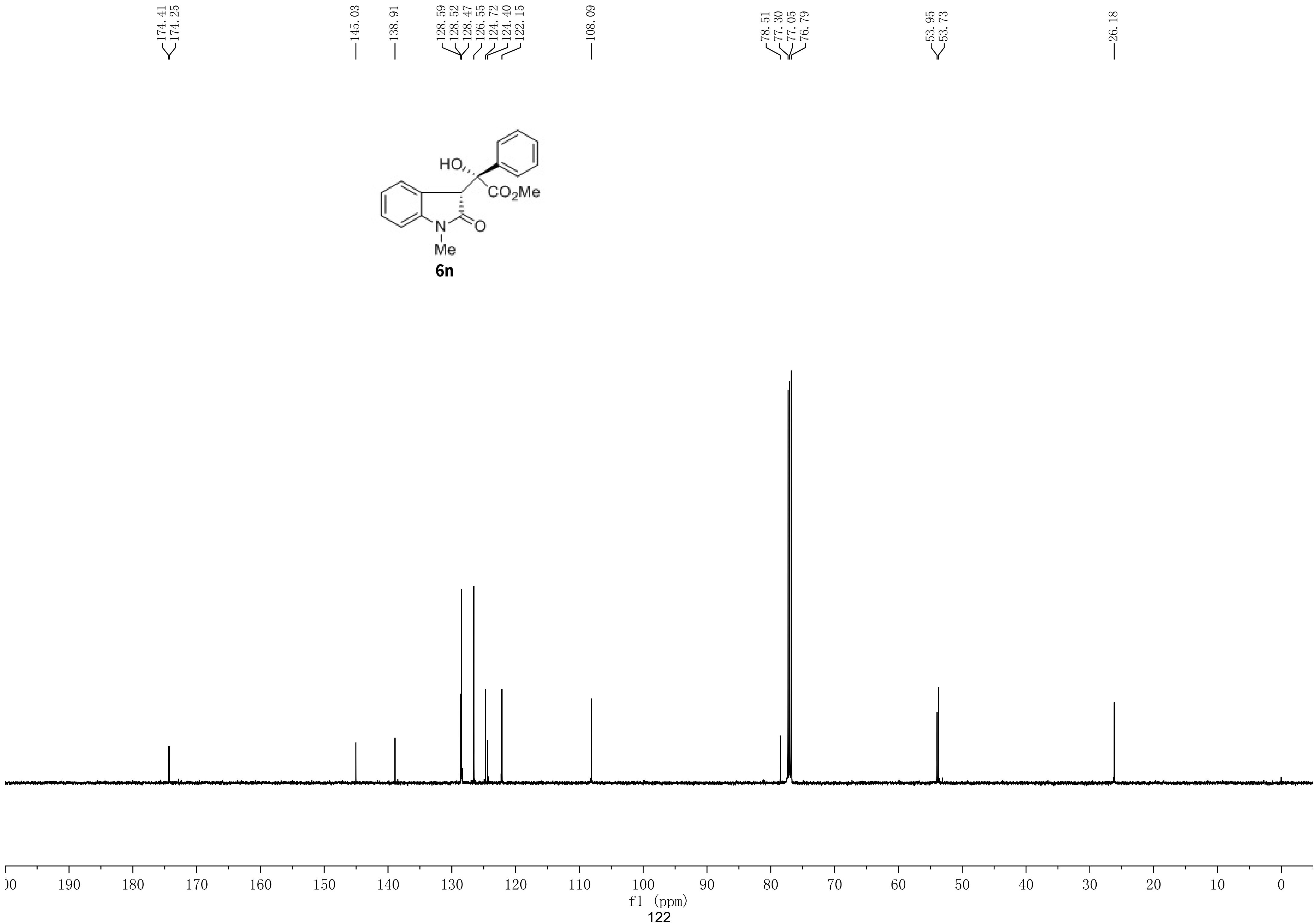
174.41
174.25
145.03
138.91
128.59
128.52
128.47
126.55
124.72
124.40
122.15
108.09
78.51
77.30
77.05
76.79
53.95
53.73
26.18
HO
CO2Me
N
O
Me
6n
190 180 170 160 150 140 130 120 110 100 90 80 70 60 50 40 30 20 10 0
f1 (ppm)

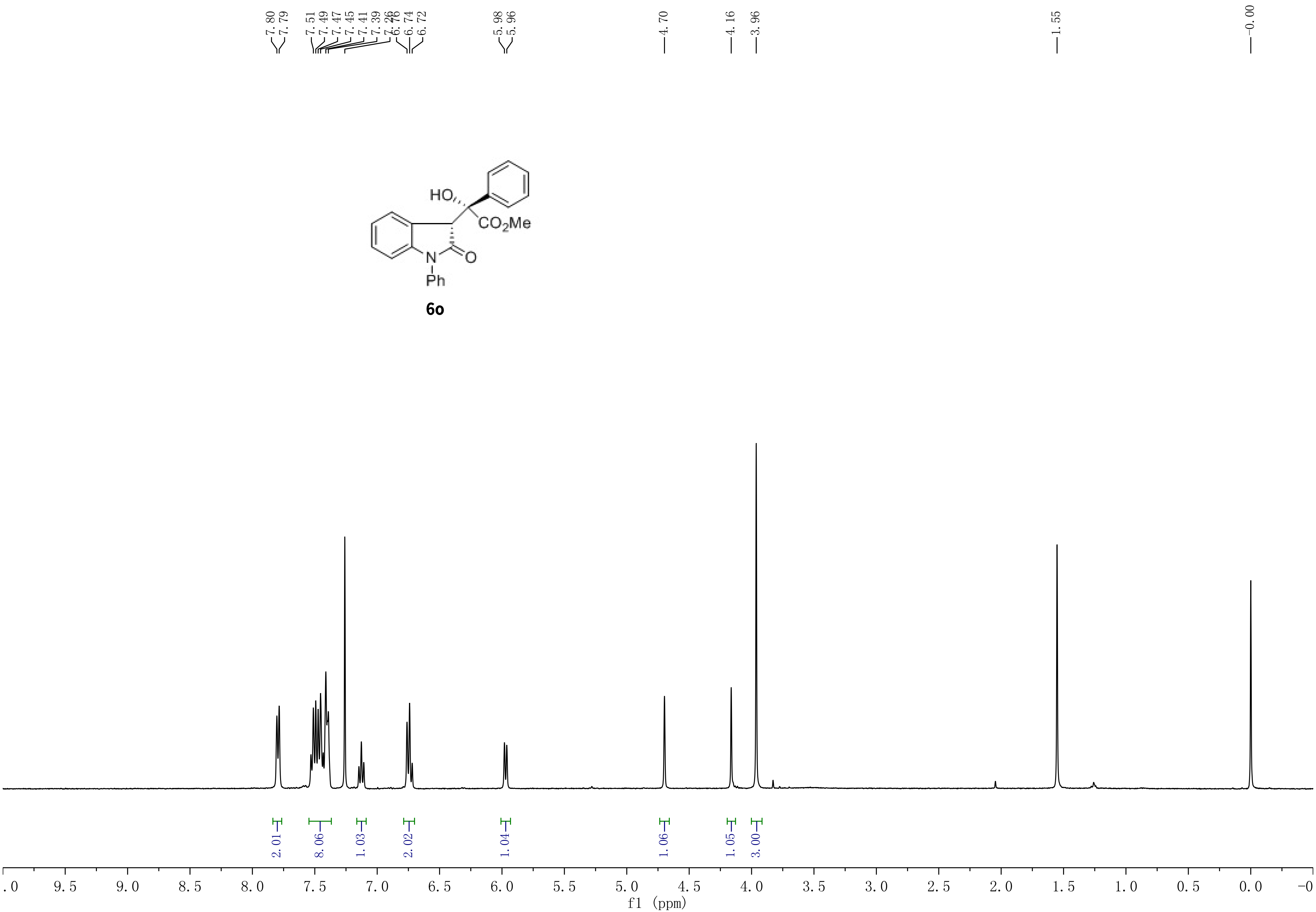
7.80
7.79
7.51
7.49
7.47
7.45
7.41
7.39
7.26
6.76
6.74
6.72
5.98
5.96
4.70
4.16
3.96
1.55
-0.00
HO
CO2Me
N
O
Ph
6o
2.01
8.06
1.03
2.02
1.04
1.06
1.05
3.00
f1 (ppm)

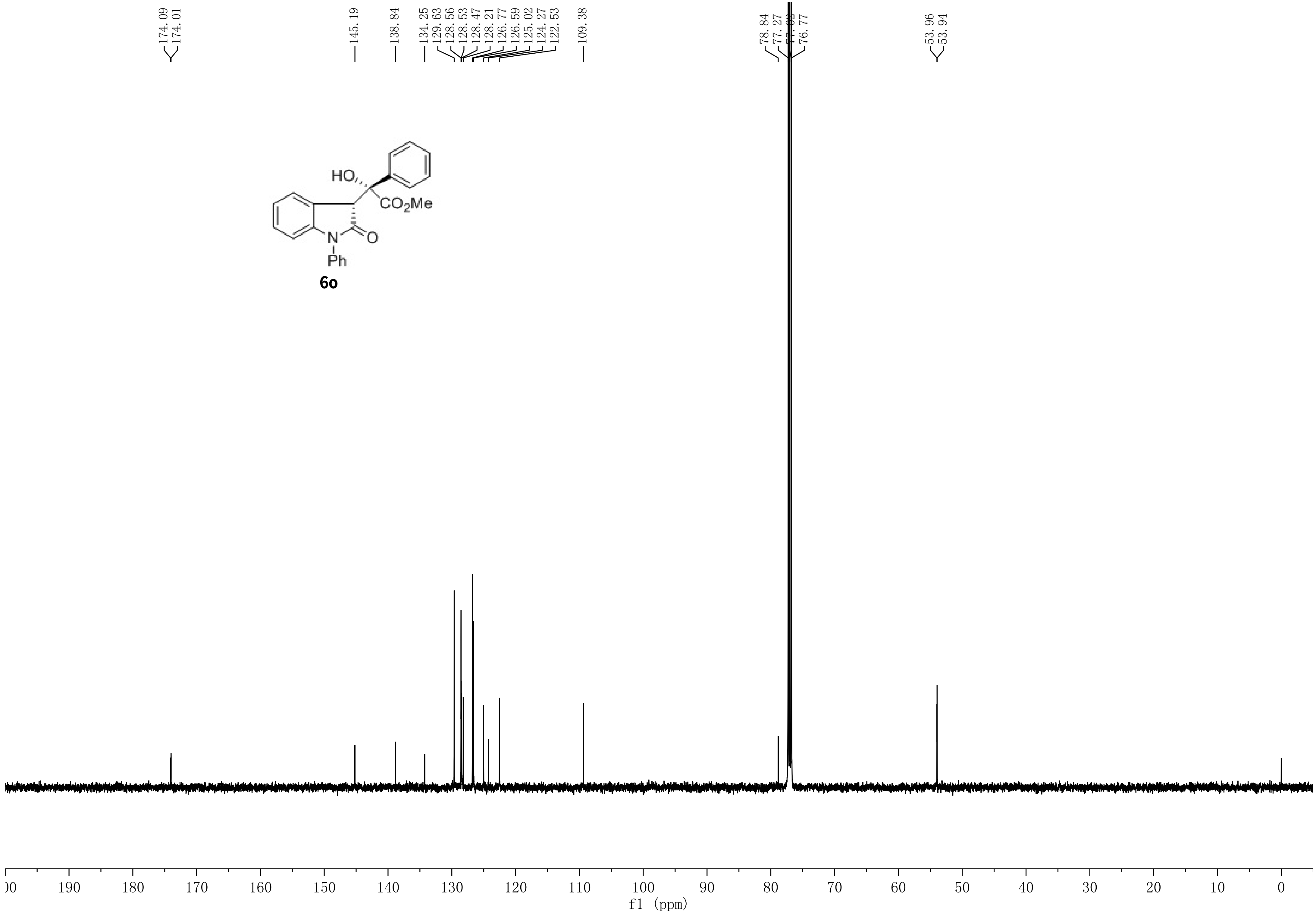
174.09
174.01
145.19
138.84
134.25
129.63
128.56
128.53
128.47
128.21
126.77
126.59
125.02
124.27
122.53
109.38
78.84
77.27
77.02
76.77
53.96
53.94
HO
CO_2Me
N
O
Ph
6o
190 180 170 160 150 140 130 120 110 100 90 80 70 60 50 40 30 20 10 0
f1 (ppm)

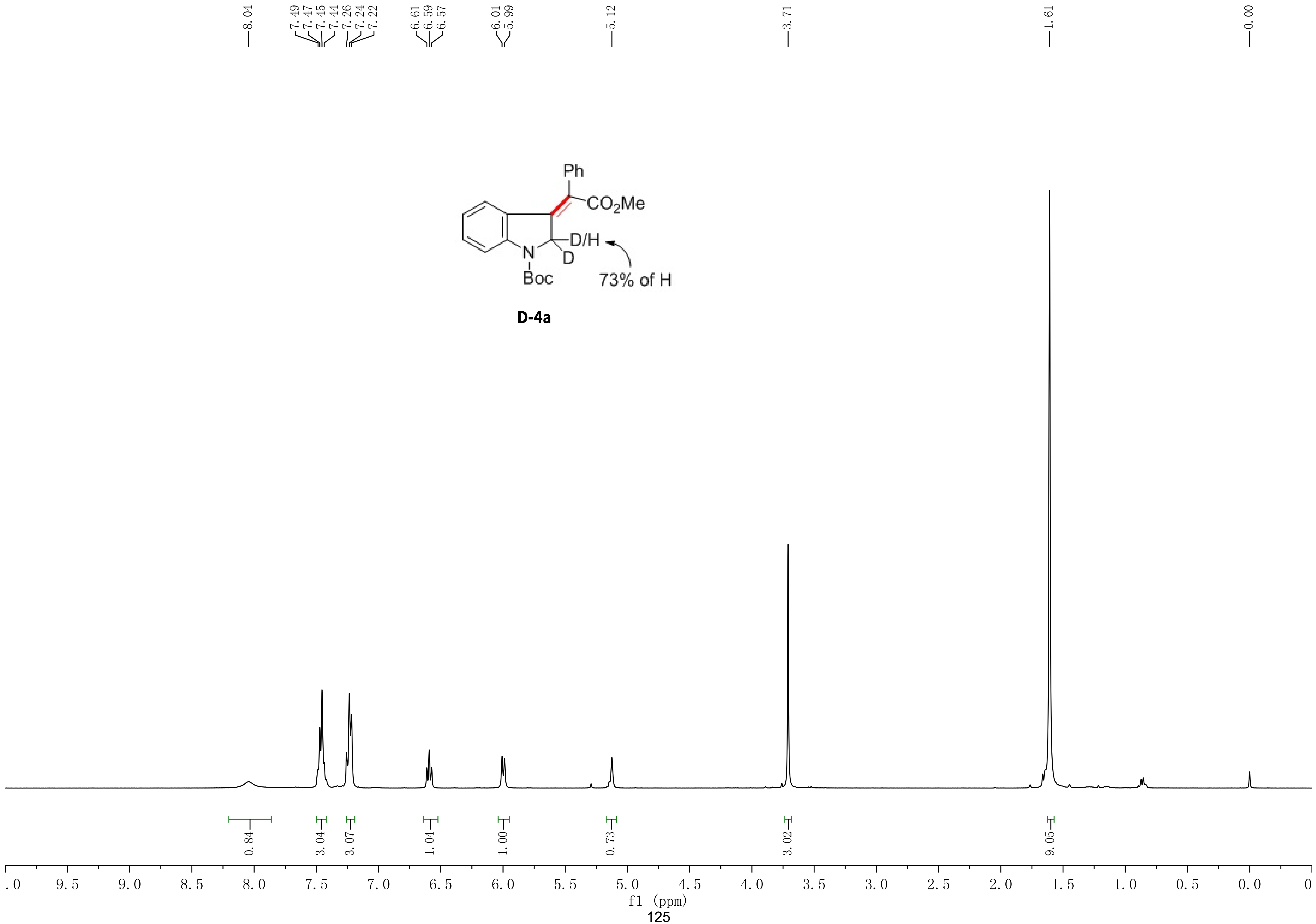
8.04
7.49
7.47
7.45
7.44
7.26
7.24
7.22
6.61
6.59
6.57
6.01
5.99
5.12
3.71
1.61
0.00
Ph
CO2Me
D/H
N
D
Boc
73% of H
D-4a
0.84
3.04
3.07
1.04
1.00
0.73
3.02
9.05
f1 (ppm)

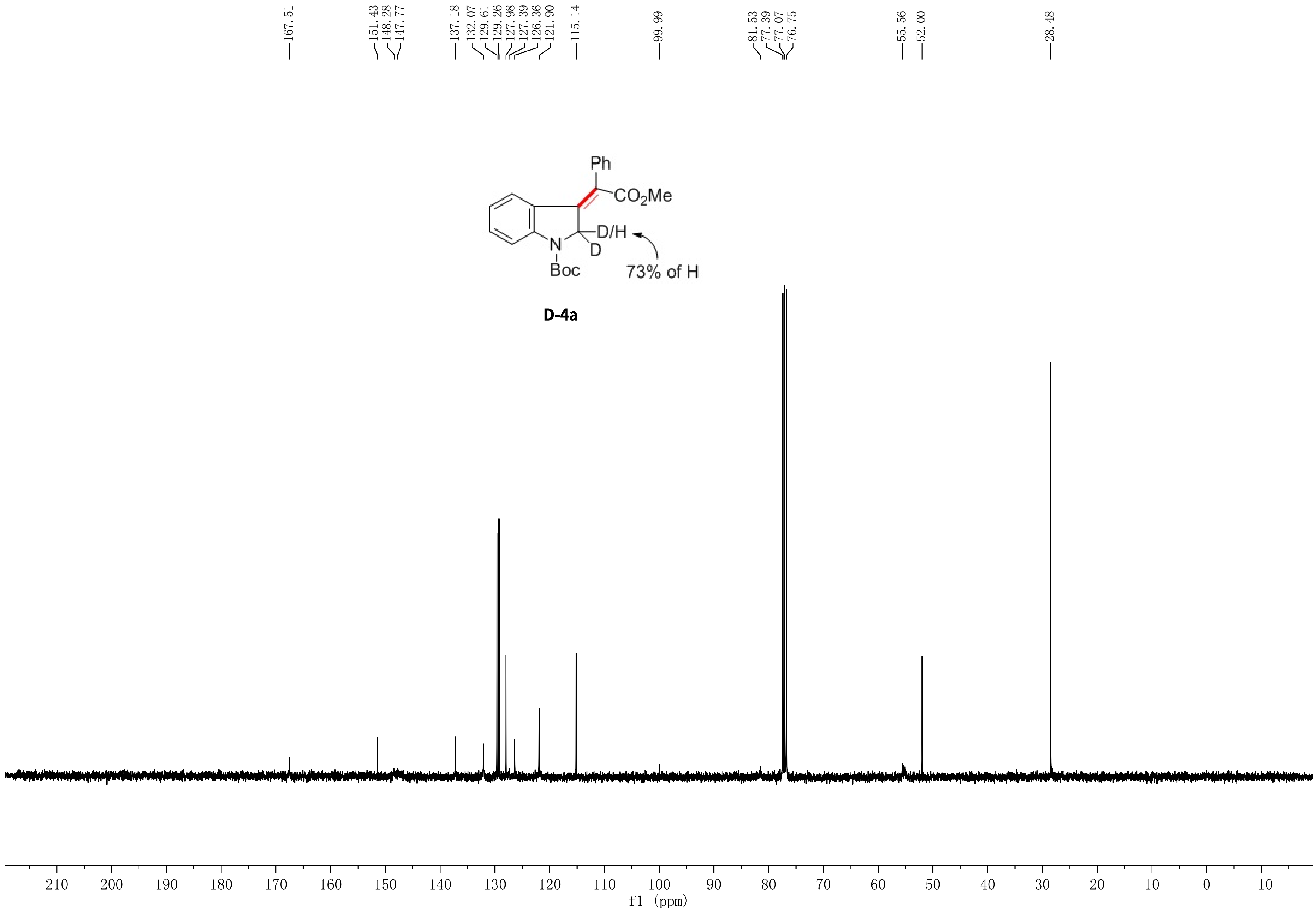
167.51
151.43
148.28
147.77
137.18
132.07
129.61
129.26
127.98
127.39
126.36
121.90
115.14
99.99
81.53
77.39
77.07
76.75
55.56
52.00
28.48
Ph
CO2Me
D/H
N
D
Boc
73% of H
D-4a
210 200 190 180 170 160 150 140 130 120 110 100 90 80 70 60 50 40 30 20 10 0 −10
f1 (ppm)

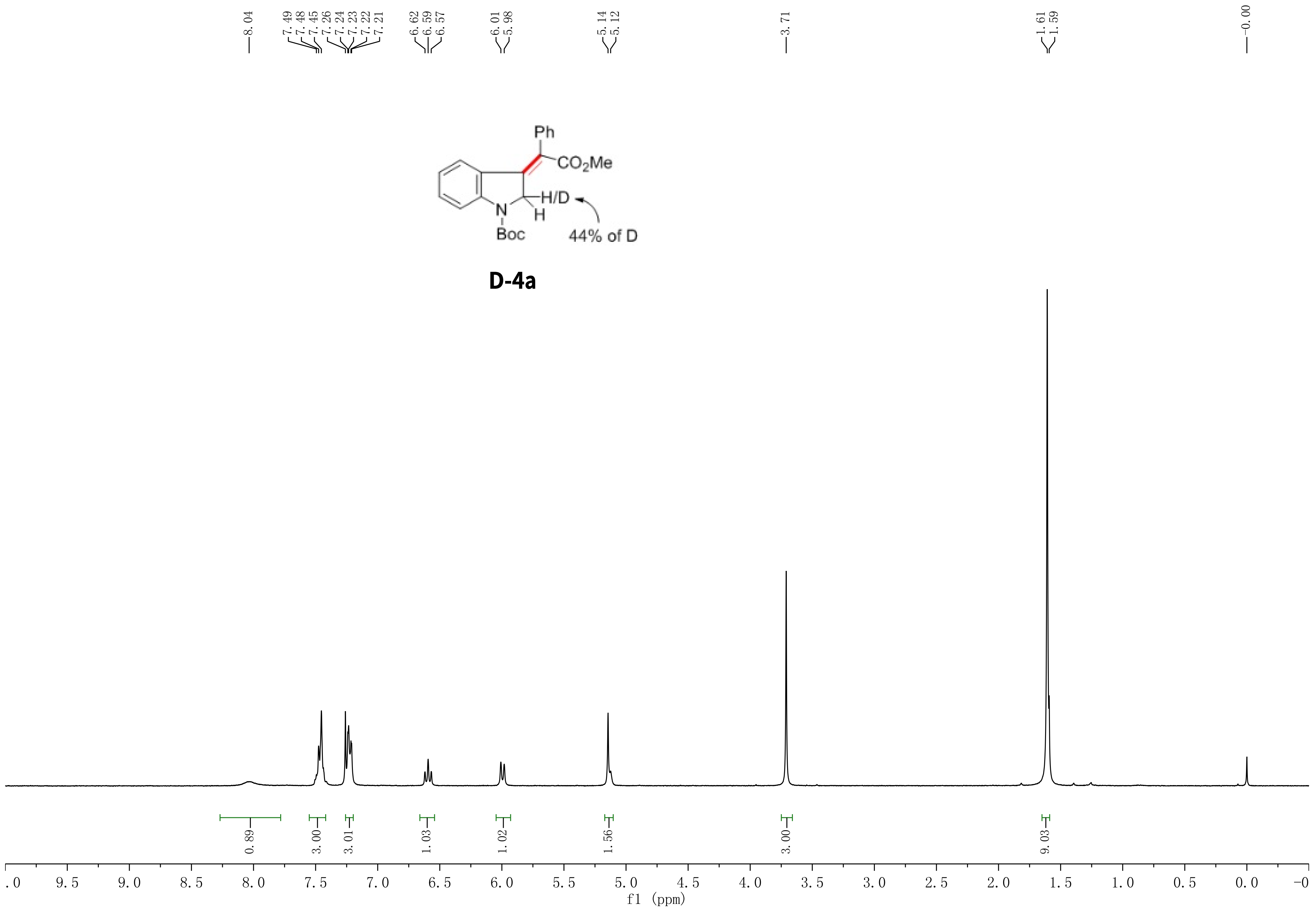

8.04
7.49
7.48
7.45
7.26
7.24
7.23
7.22
7.21
6.62
6.59
6.57
6.01
5.98
5.14
5.12
3.71
1.61
1.59
0.00
Ph
CO2Me
H/D
H
N
Boc
44% of D
D-4a
0.89
3.00
3.01
1.03
1.02
1.56
3.00
9.03
f1 (ppm)

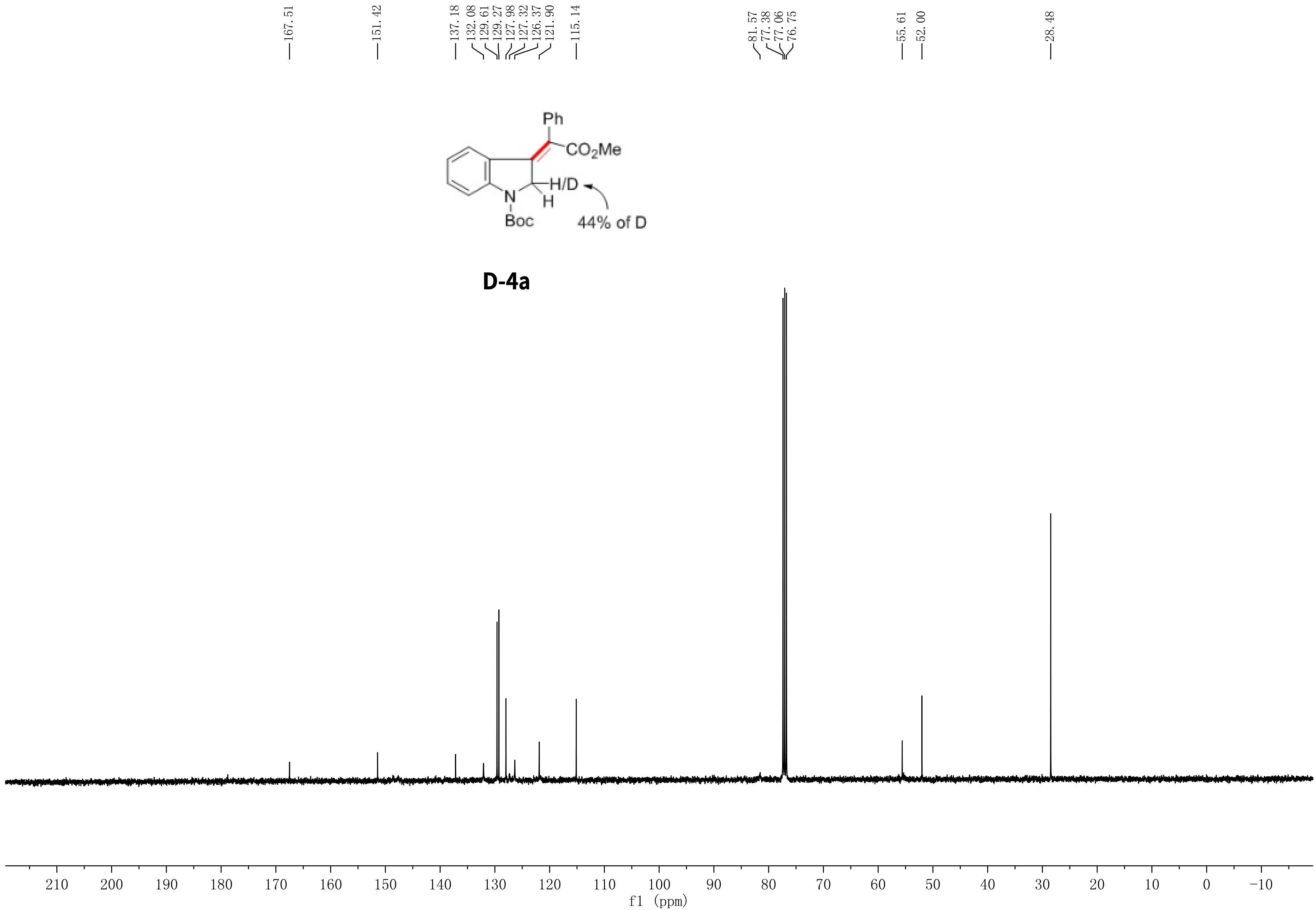

167.51
151.42
137.18
132.08
129.61
129.27
127.98
127.32
126.37
121.90
115.14
81.57
77.38
77.06
76.75
55.61
52.00
28.48
Ph
CO2Me
H/D
H
N
Boc
44% of D
D-4a
210 200 190 180 170 160 150 140 130 120 110 100 90 80 70 60 50 40 30 20 10 0 -10
f1 (ppm)

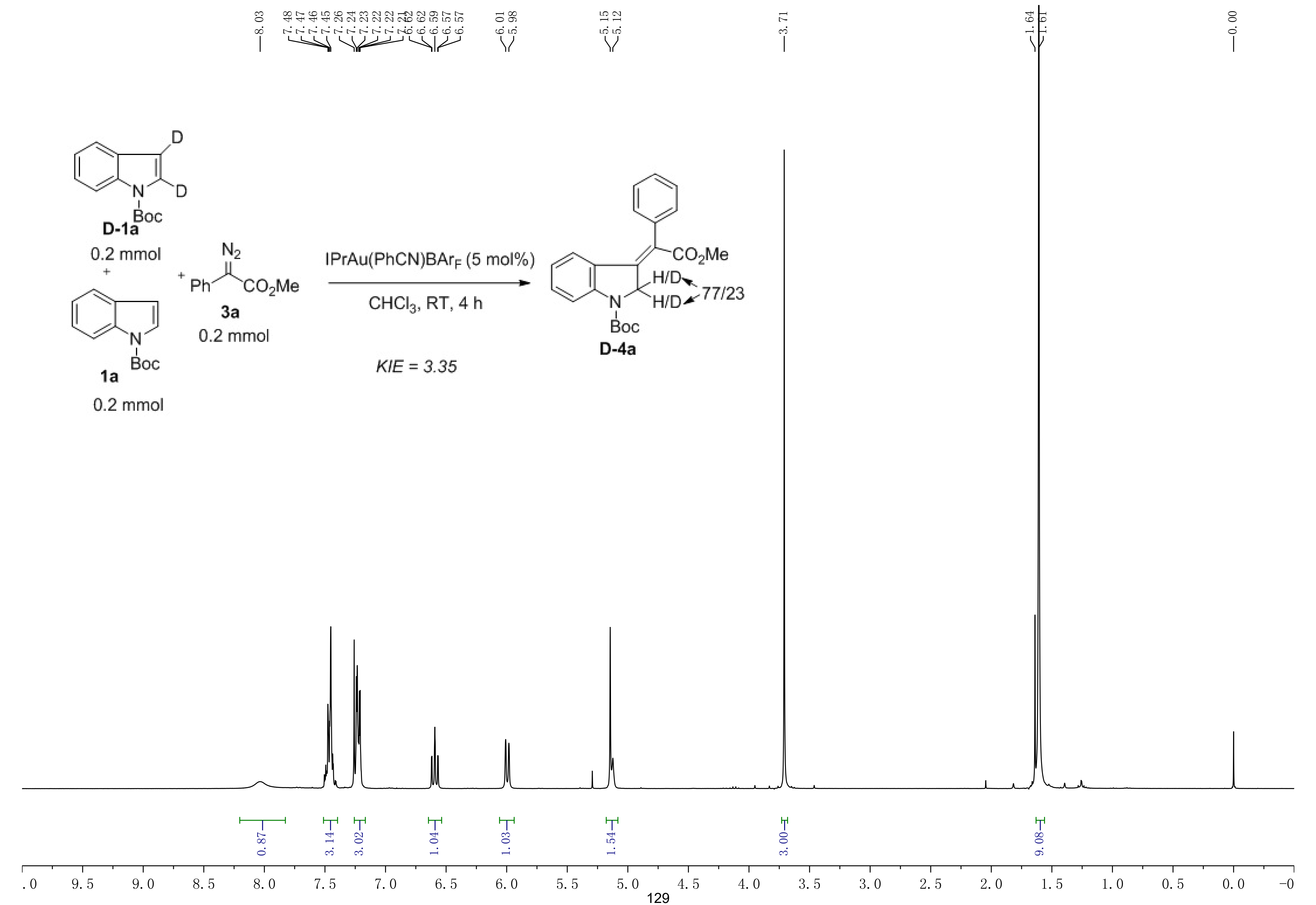
D
D
N
Boc
D-1a
0.2 mmol
+
N
Boc
1a
0.2 mmol
+
N2
Ph
CO2Me
3a
0.2 mmol
IPrAu(PhCN)BArF (5 mol%)
CHCl3, RT, 4 h
KIE = 3.35
CO2Me
H/D
H/D
77/23
N
Boc
D-4a
8.03
7.48
7.47
7.46
7.45
7.26
7.24
7.23
7.22
7.22
7.21
6.62
6.62
6.59
6.57
6.57
6.01
5.98
5.15
5.12
3.71
1.64
1.61
0.00
0.87
3.14
3.02
1.04
1.03
1.54
3.00
9.08

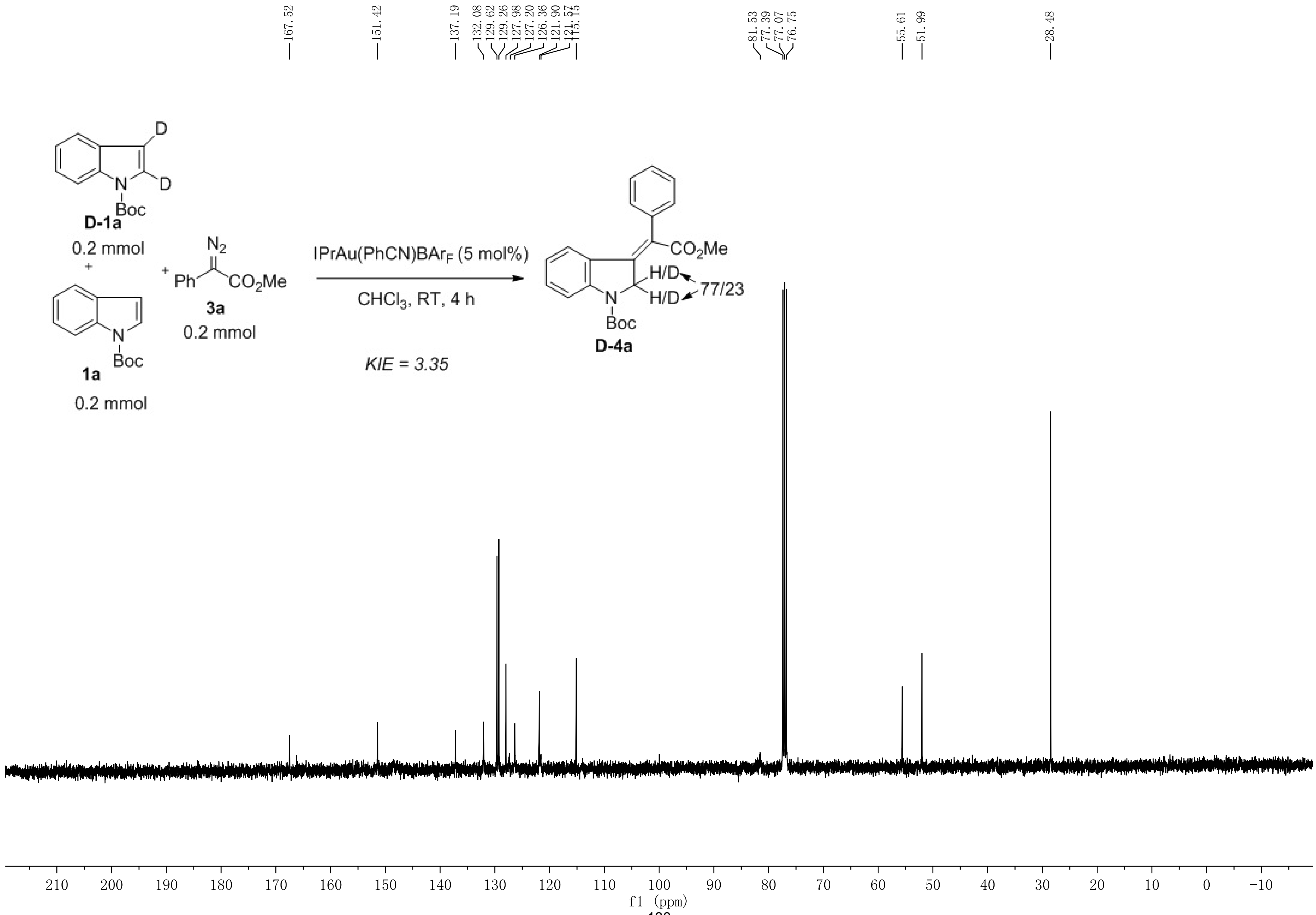

167.52
151.42
137.19
132.08
129.62
129.26
127.98
127.20
126.36
121.90
121.57
115.15
81.53
77.39
77.07
76.75
55.61
51.99
28.48
D
D
N
Boc
D-1a
0.2 mmol
+
N
Boc
1a
0.2 mmol
+
N2
Ph
CO2Me
3a
0.2 mmol
IPrAu(PhCN)BArF (5 mol%)
CHCl3, RT, 4 h
KIE = 3.35
CO2Me
H/D
H/D
77/23
N
Boc
D-4a
210
200
190
180
170
160
150
140
130
120
110
100
90
80
70
60
50
40
30
20
10
0
-10
f1 (ppm)

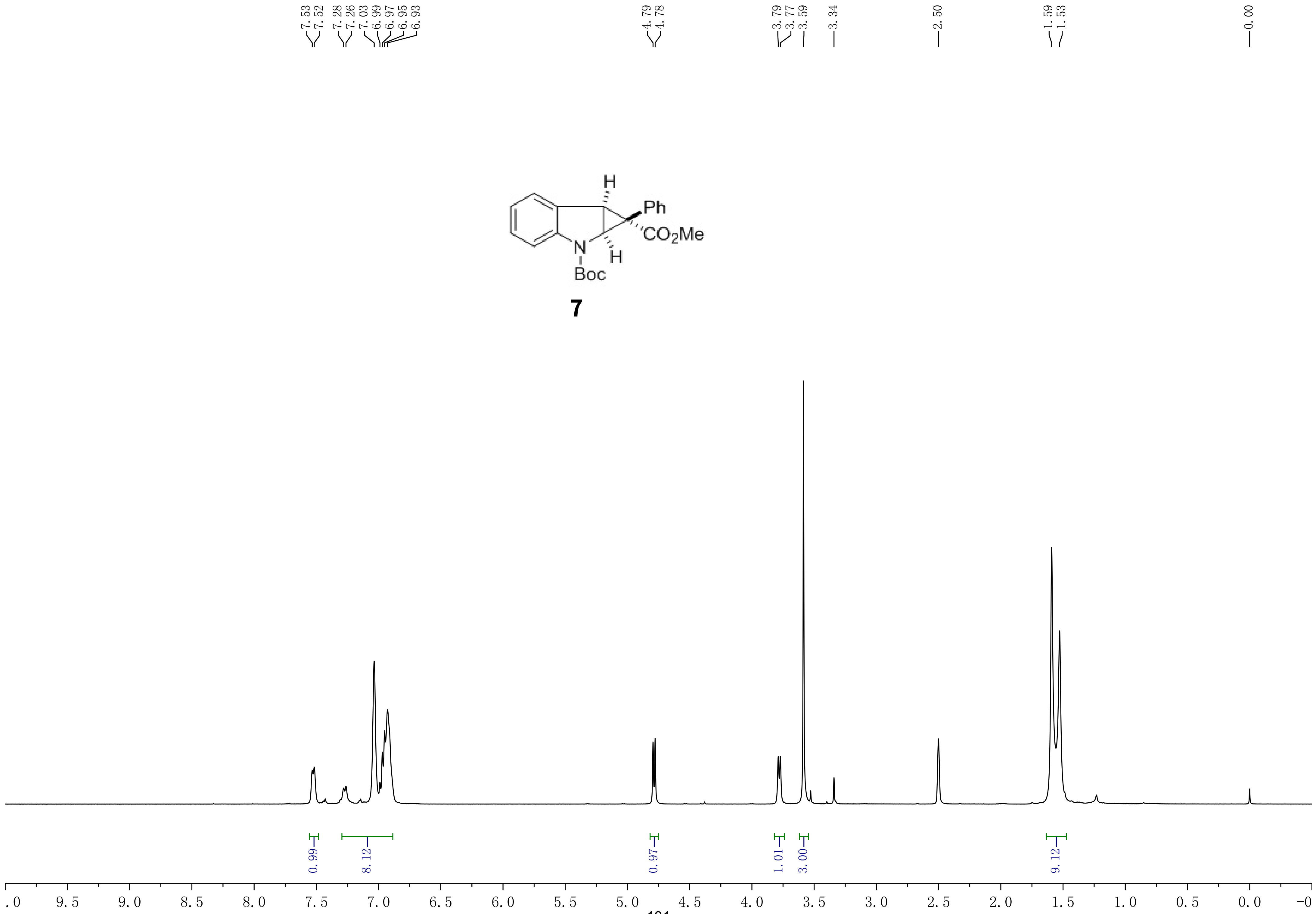
7.53
7.52
7.28
7.26
7.03
6.99
6.97
6.95
6.93
4.79
4.78
3.79
3.77
3.59
3.34
2.50
1.59
1.53
0.00
H
Ph
CO2Me
N
H
Boc
7
0.99
8.12
0.97
1.01
3.00
9.12
.0
9.5
9.0
8.5
8.0
7.5
7.0
6.5
6.0
5.5
5.0
4.5
4.0
3.5
3.0
2.5
2.0
1.5
1.0
0.5
0.0
−0

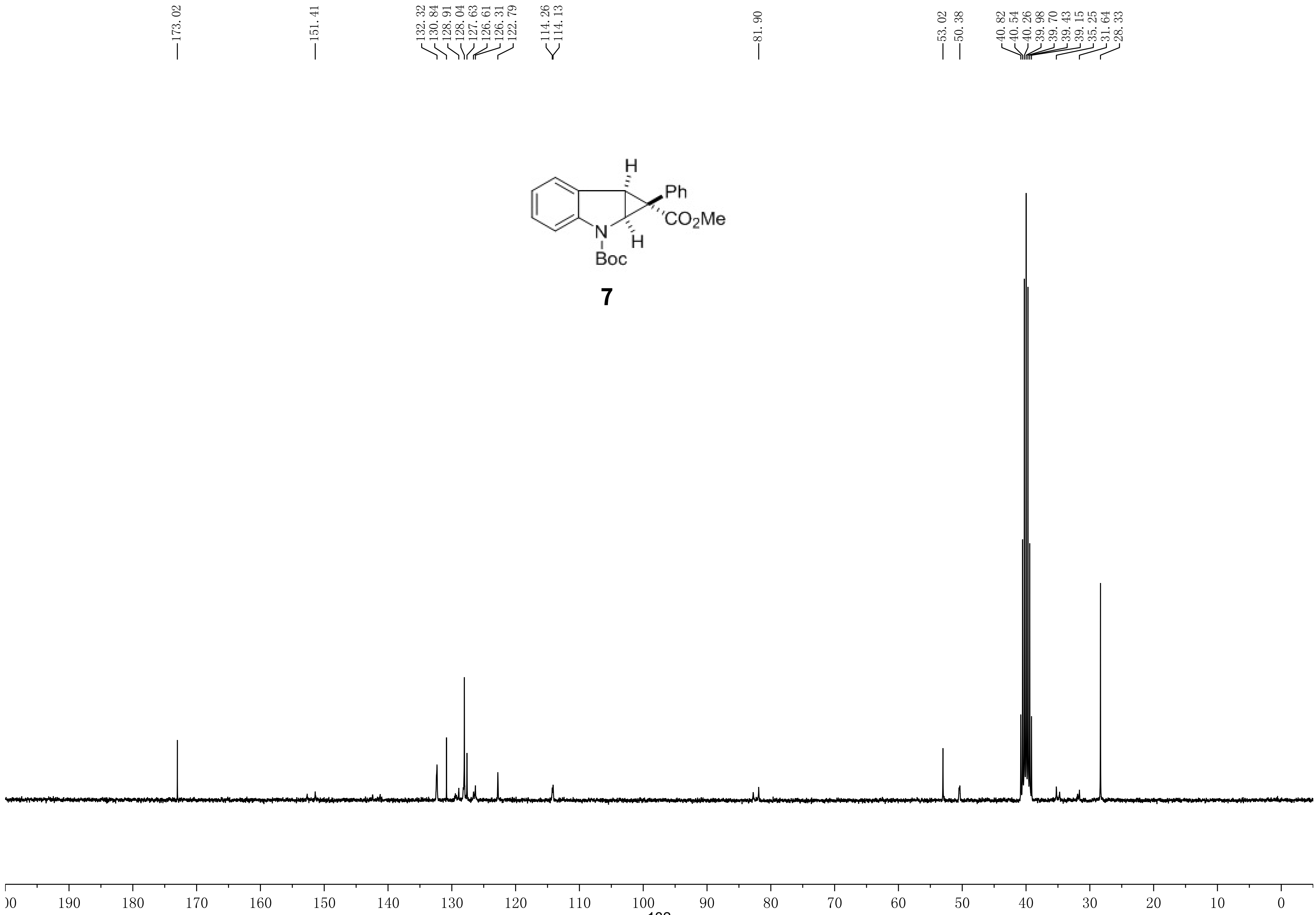
173.02
151.41
132.32
130.84
128.91
128.04
127.63
126.61
126.31
122.79
114.26
114.13
81.90
53.02
50.38
40.82
40.54
40.26
39.98
39.70
39.43
39.15
35.25
31.64
28.33
H
Ph
CO_2Me
N
H
Boc
7
190
180
170
160
150
140
130
120
110
100
90
80
70
60
50
40
30
20
10
0

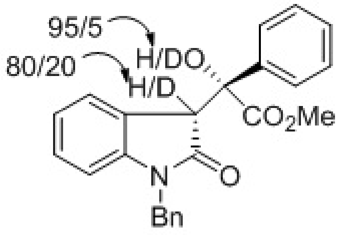

D-6a

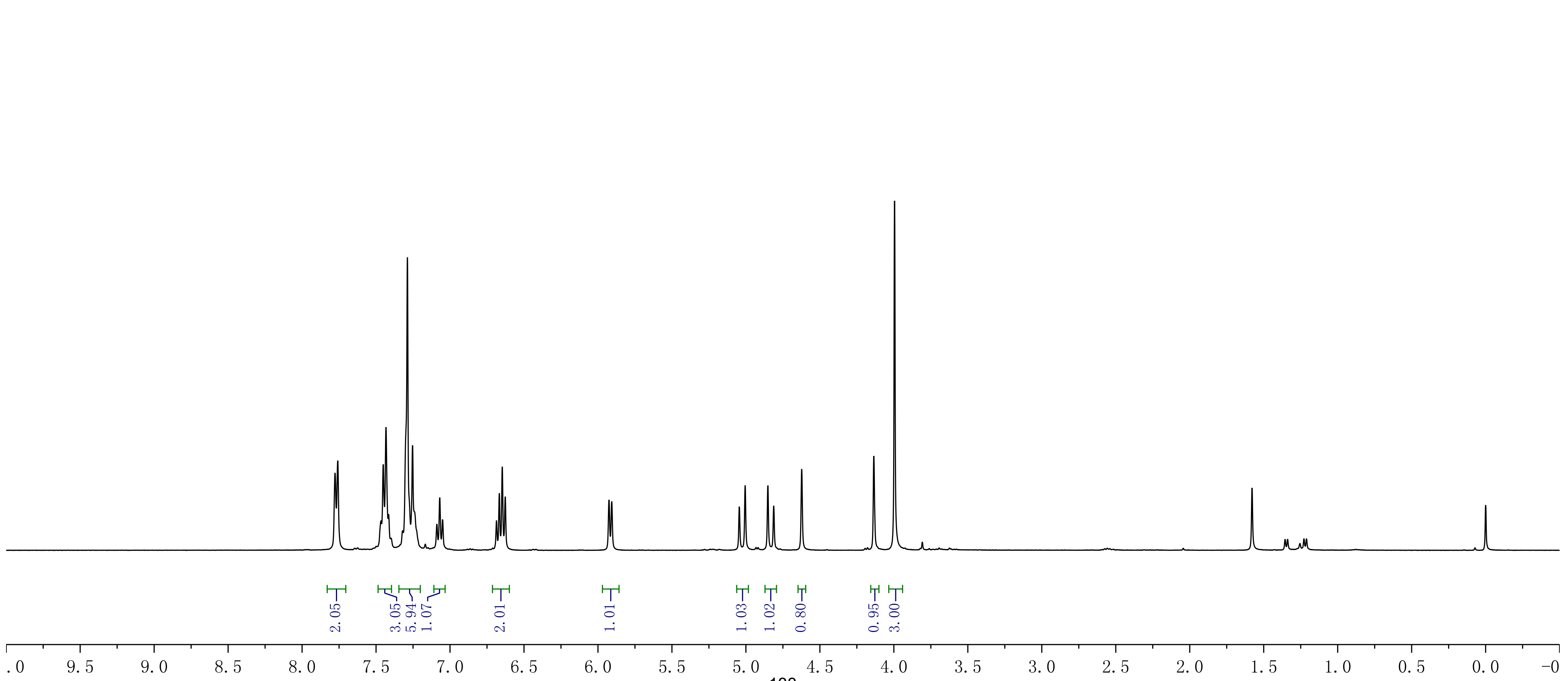

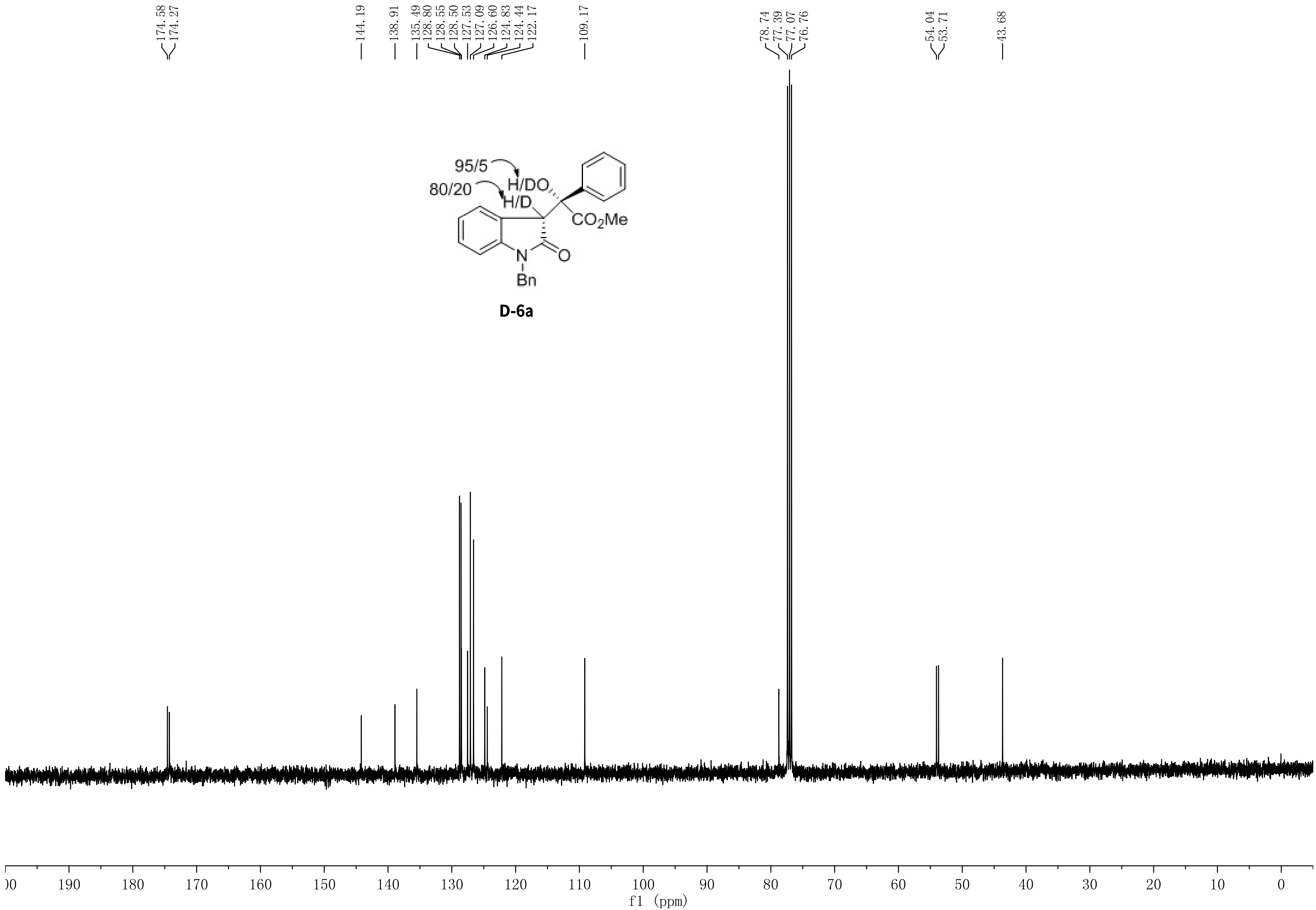
174.58
174.27
144.19
138.91
135.49
128.80
128.55
128.50
127.53
127.09
126.60
124.83
124.44
122.17
109.17
78.74
77.39
77.07
76.76
54.04
53.71
43.68
95/5
80/20
H/DO
H/D
CO2Me
N
O
Bn
D-6a
190
180
170
160
150
140
130
120
110
100
90
80
70
60
50
40
30
20
10
0
f1 (ppm)

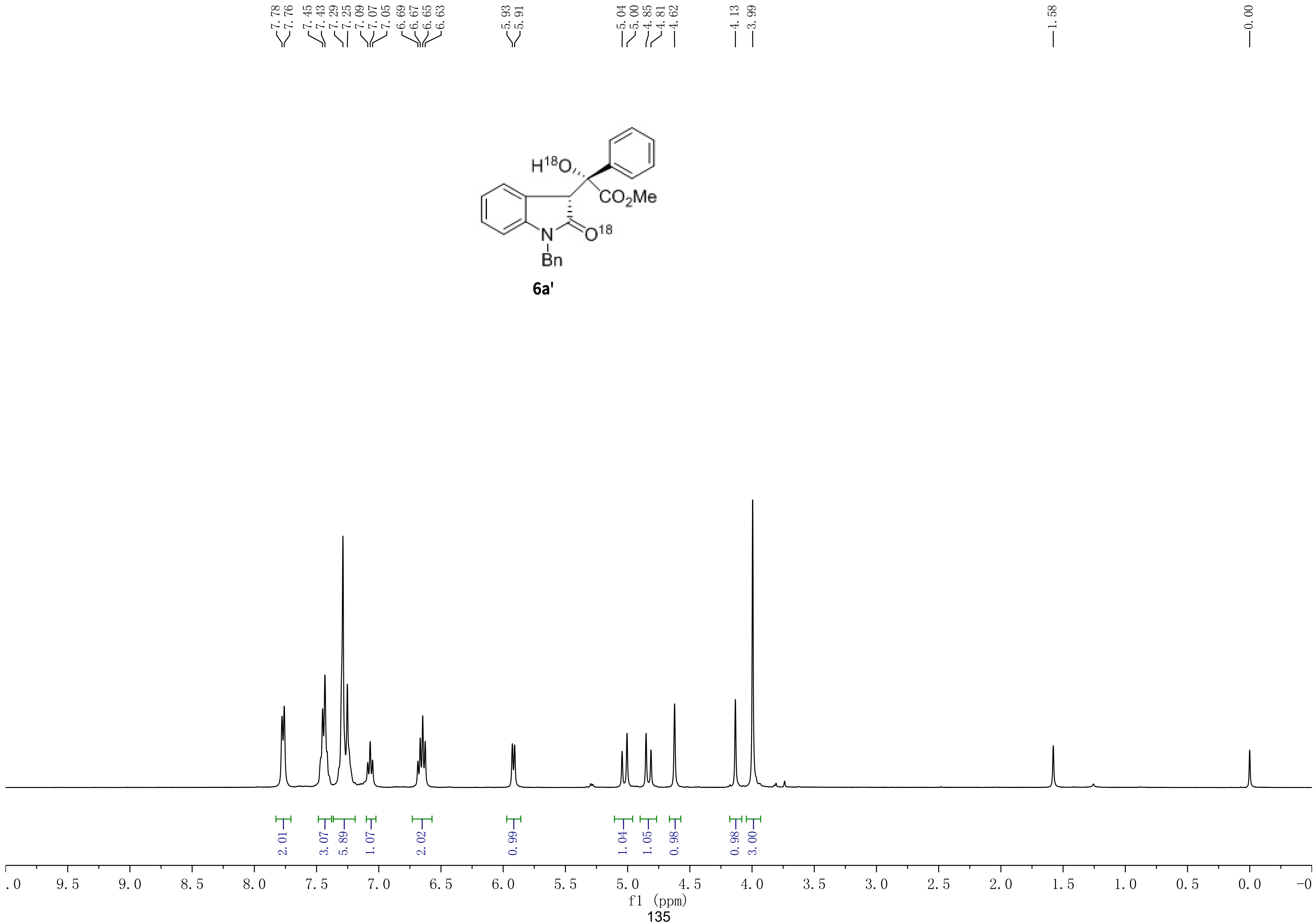
7.78
7.76
7.45
7.43
7.29
7.25
7.09
7.07
7.05
6.69
6.67
6.65
6.63
5.93
5.91
5.04
5.00
4.85
4.81
4.62
4.13
3.99
1.58
0.00
H18O
CO2Me
N
O18
Bn
6a'
2.01
3.07
5.89
1.07
2.02
0.99
1.04
1.05
0.98
0.98
3.00
.0
9.5
9.0
8.5
8.0
7.5
7.0
6.5
6.0
5.5
5.0
4.5
4.0
3.5
3.0
2.5
2.0
1.5
1.0
0.5
0.0
-0
f1 (ppm)

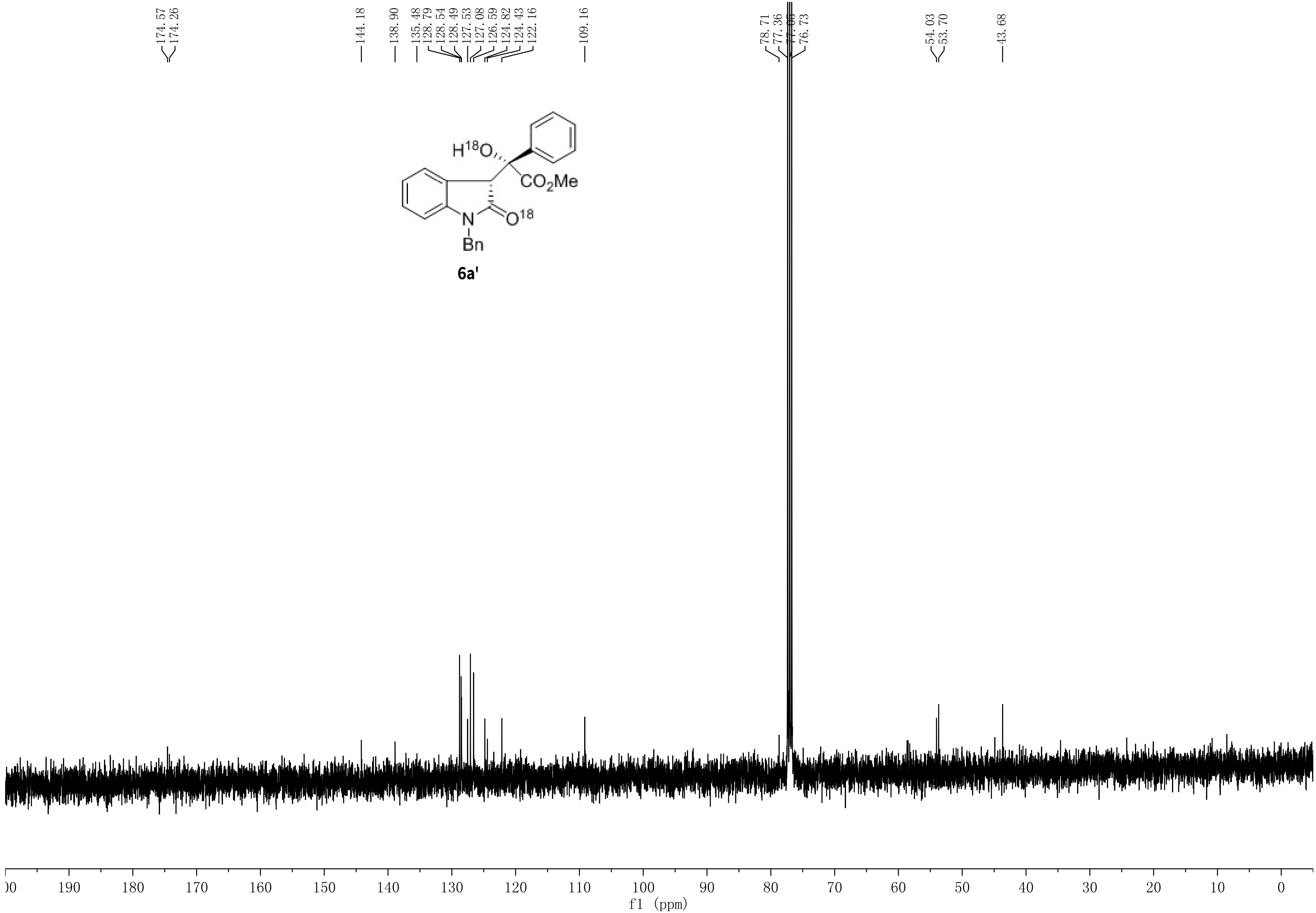
174.57
174.26
144.18
138.90
135.48
128.79
128.54
128.49
127.53
127.08
126.59
124.82
124.43
122.16
109.16
78.71
77.36
76.73
54.03
53.70
43.68
H18O
CO2Me
N
O18
Bn
6a'
f1 (ppm)

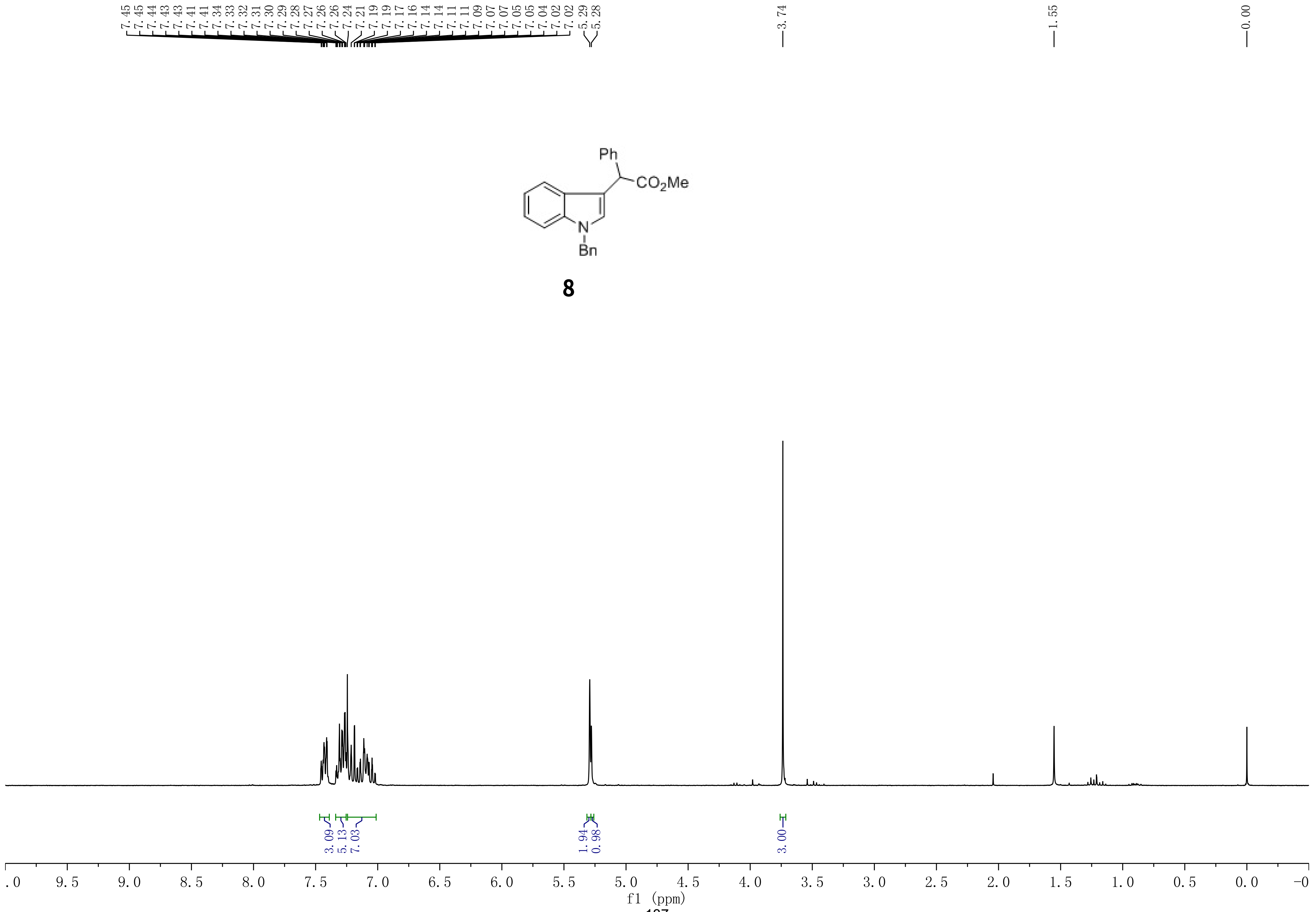

8

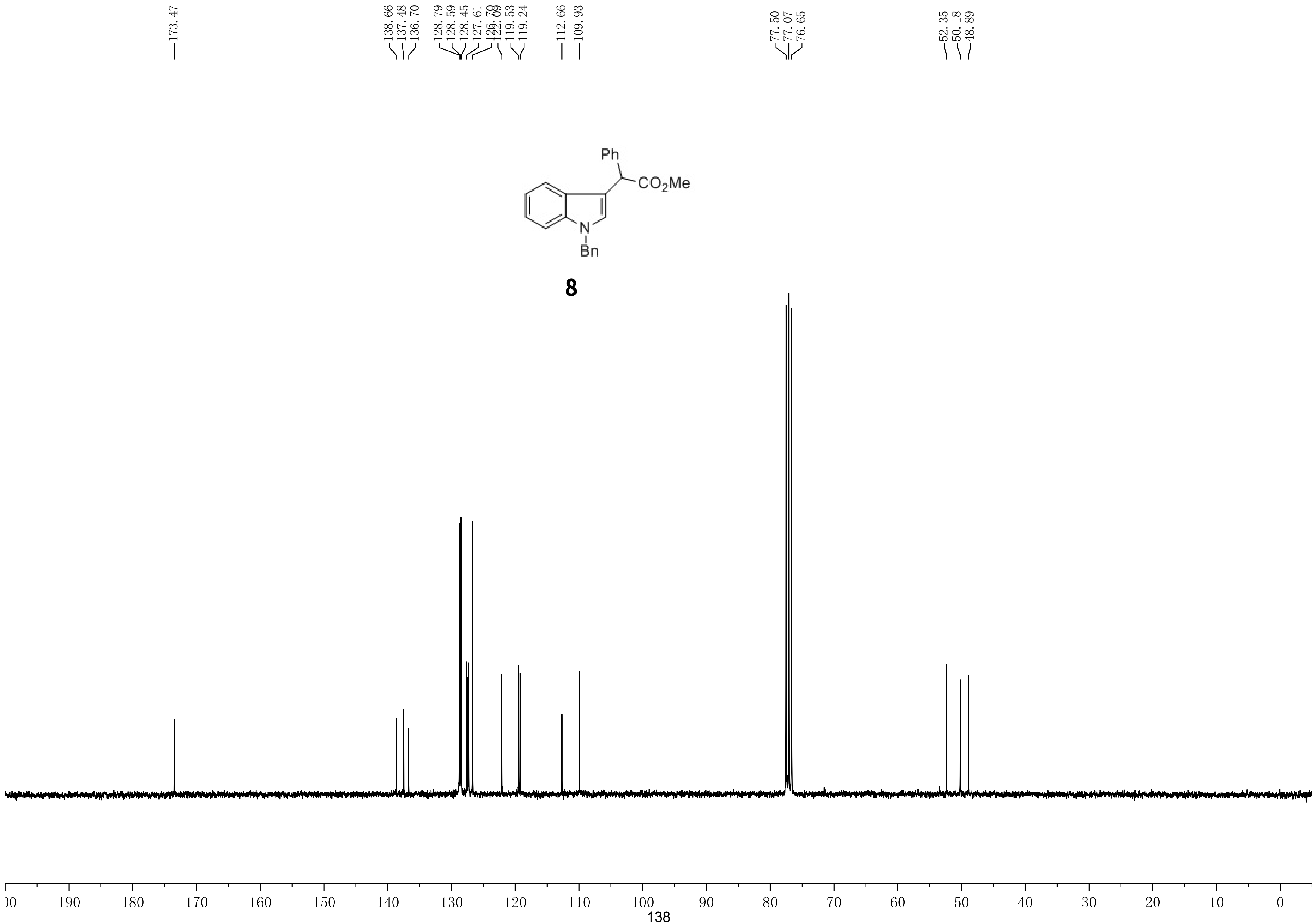
173.47
138.66
137.48
136.70
128.79
128.59
128.45
127.61
126.70
122.09
119.53
119.24
112.66
109.93
77.50
77.07
76.65
52.35
50.18
48.89
Ph
CO_2Me
N
Bn
8
190
180
170
160
150
140
130
120
110
100
90
80
70
60
50
40
30
20
10
0

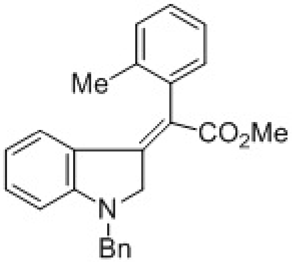
Me
CO2Me
N
Bn

9

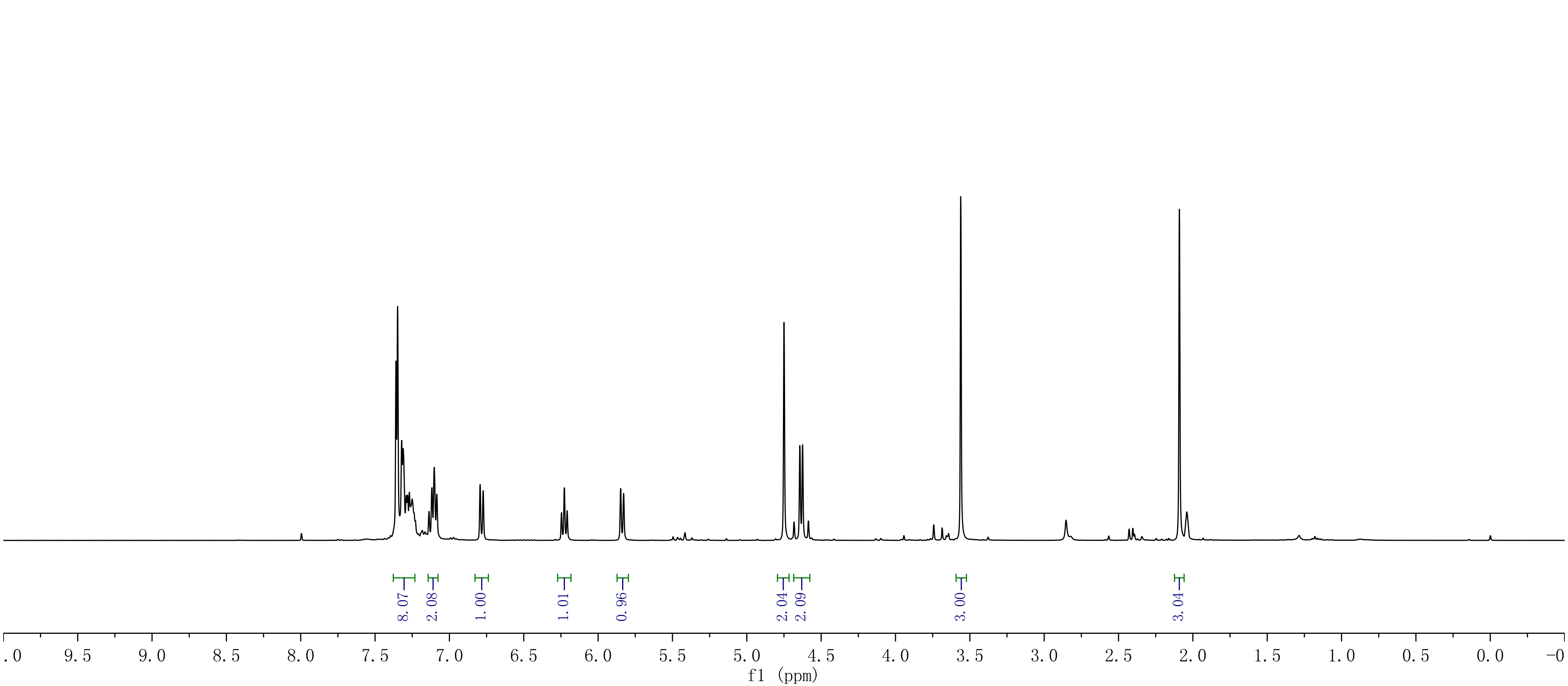
7.36
7.35
7.32
7.31
7.29
7.28
7.27
7.25
7.24
7.23
7.14
7.12
7.10
7.08
6.79
6.77
6.25
6.23
6.21
5.85
5.83
4.75
4.68
4.64
4.62
4.59
3.56
2.85
2.09
2.04
0.00
8.07
2.08
1.00
1.01
0.96
2.04
2.09
3.00
3.04
9.5
9.0
8.5
8.0
7.5
7.0
6.5
6.0
5.5
5.0
4.5
4.0
3.5
3.0
2.5
2.0
1.5
1.0
0.5
0.0
f1 (ppm)

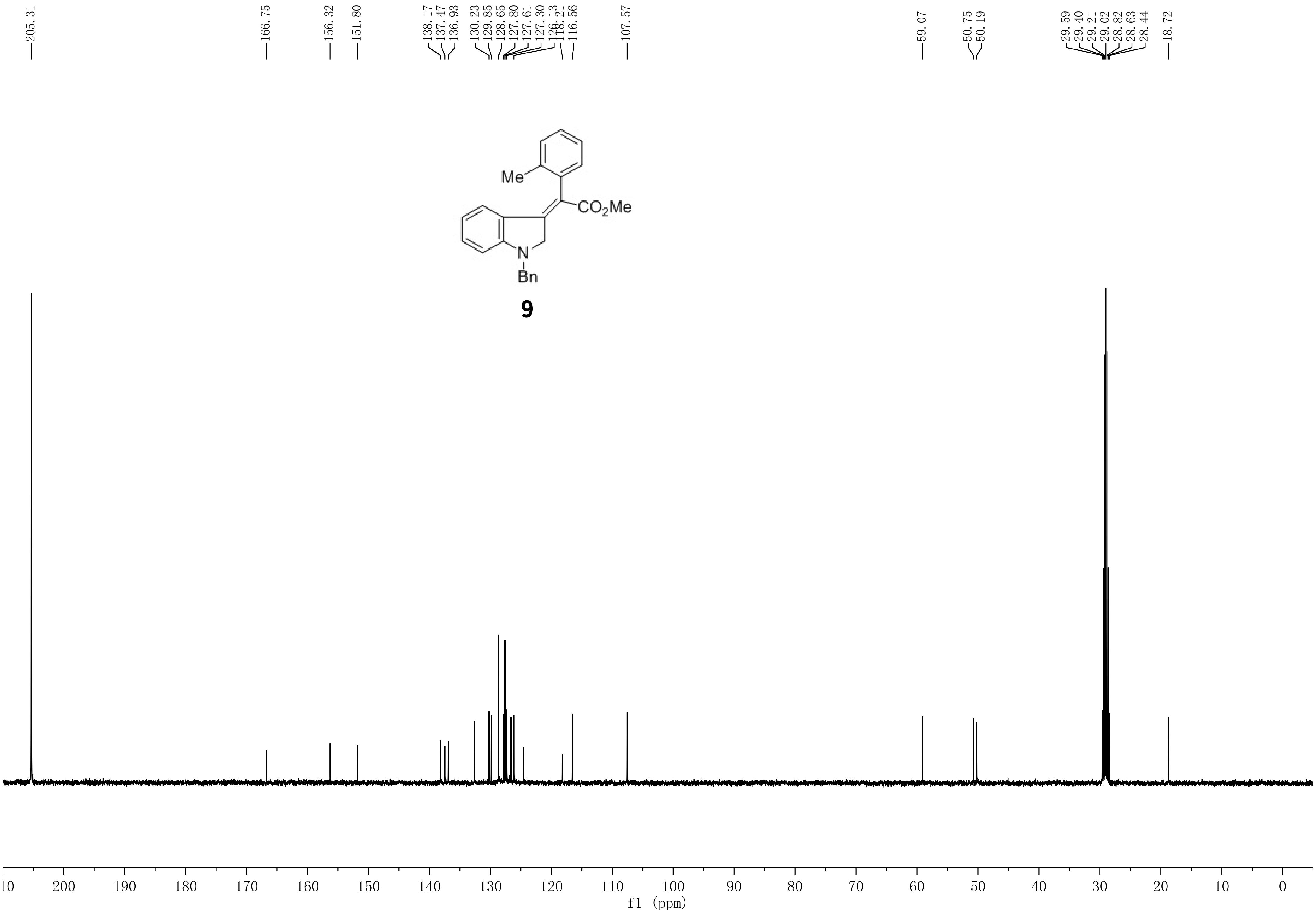
205.31
166.75
156.32
151.80
138.17
137.47
136.93
130.23
129.85
128.65
127.80
127.61
127.30
126.13
118.21
116.56
107.57
59.07
50.75
50.19
29.59
29.40
29.21
29.02
28.82
28.63
28.44
18.72
Me
CO_2Me
N
Bn
10
200
190
180
170
160
150
140
130
120
110
100
90
80
70
60
50
40
30
20
10
0
f1 (ppm)

9
